# Genomic and Transcriptomic Analysis of Mutant *Bacillus subtilis* with Enhanced Nattokinase Production via ARTP Mutagenesis

**DOI:** 10.3390/foods14050898

**Published:** 2025-03-06

**Authors:** Liuyu Guo, Yang Chen, Zhiyong He, Zhaojun Wang, Qiuming Chen, Jie Chen, Fatih Oz, Zhimin Xu, Maomao Zeng

**Affiliations:** 1 State Key Laboratory of Food Science and Resources, Jiangnan University, Wuxi 214122, China; guoliuyu2024@163.com (L.G.); c756542878@163.com (Y.C.); zyhe@jiangnan.edu.cn (Z.H.); zhaojun.wang@jiangnan.edu.cn (Z.W.); chenqm@jiangnan.edu.cn (Q.C.); chenjie@jiangnan.edu.cn (J.C.); 2 School of Food Science and Technology, Jiangnan University, Wuxi 214122, China; 3 Department of Food Engineering, Faculty of Agriculture, Ataturk University, Erzurum 25240, Turkey; fatihoz@atauni.edu.tr; 4 School of Nutrition and Food Sciences, Louisiana State University, Baton Rouge, LA 70803, USA; zxu@agcenter.lsu.edu

**Keywords:** nattokinase, *Bacillus subtilis*, ARTP, genome resequencing, transcriptome

## Abstract

Nattokinase (NK), a serine protease with high thrombolytic activity, has significant potential for application in foods intended for special health benefits. However, the NK production in wild-type *Bacillus subtilis* natto is relatively low. In this study, a high-yielding NK and genetically stable mutant strain (*B. subtilis* JNC002.001, 300.0 ± 4.7 FU/mL) was obtained through atmospheric and room temperature plasma (ARTP) mutagenesis. It increased NK activity by 1.84 times compared to the initial strain SD2, demonstrating significant prospects for NK production and food fermentation applications. Additionally, the *B. subtilis* JNC002.001 exhibited notable alterations in growth characteristics, glucose consumption, and sporulation. This study further elucidated the mechanism of enhanced NK production at the molecular level. Genome resequencing revealed that the mutant genes in JNC002.001 included 10 single nucleotide polymorphisms (SNPs) and one insertion, among which the *kinA* and *gltA* genes were associated with sporulation and NK synthesis, respectively. In terms of the transcriptional level, the NK-coding gene *aprN* was up-regulated 9.4 times relative to the wild-type strain. Most of the genes related to central carbon metabolism and the Sec secretion pathway were up-regulated. In addition, the expression of regulatory factors associated with the transcription of the *aprN* gene and the sporulation process provided evidence for high NK expression and sporulation deficiency in JNC002.001. These results could provide insights into the mechanism of NK production and facilitate the construction of engineered strains with high NK yield.

## 1. Introduction

Nattokinase (NK, EC 3.4.21.62) is a serine protease of the subtilisin family that is secreted by *B. subtilis* natto. It was originally discovered in natto, hence the name, coming from [1,2]. The gene encoding NK is *aprN*, which possesses an open reading frame of 1146 bp and encodes a signal peptide of 29 amino acid residues, a pre-peptide of 77 amino acid residues and a mature peptide of 275 amino acid residues (27.7 kDa) [3]. A number of studies have demonstrated the preventive and palliative effects of NK on cardiovascular diseases, with promising efficacy in antithrombotic treatments [4,5,6], antiplatelet [7], lipid-lowering, and anti-atherosclerotic [8] abilities, and hypertension prevention [9]. Notably, compared with traditional thrombolytic drugs, NK has several advantageous characteristics, including high safety, convenient oral administration, a long half-life, and economical use [10,11]. However, the low production of NK by wild-type *B. subtilis* natto limits the industrial scale of NK production and food application.

Despite the fact there have been many efforts to identify natural high-yielding NK strains and optimize the fermentation conditions, the yield of NK is still not high enough to meet desirable food application [10]. Previous studies have demonstrated that genetic engineering-mediated overexpression of the *aprN* gene significantly enhances NK production [2,10]. Through optimization of the gene’s expression elements, two engineered strains with respective NK activities of 270 and 290 FU/mL were successfully developed [12,13]. However, the utilization of genetically engineered strains in food applications is constrained by safety considerations [14,15], while the instability of enzymatic activity—arising from plasmid loss, metabolic burden, or environmental stress—remains a challenge in scaled production [15,16]. Physical mutagenesis is a straightforward, secure, efficient, and pervasive breeding technique that can enhance the enzyme-producing capabilities of *B. subtilis*. Wang et al. obtained a mutant strain with NK activity of 12,656 IU/mL by ultraviolet treatment combined with ^60^Co-γ ray mutagenesis, which was a 2-fold increase compared to the initial strain [10,17]. Sheng et al. increased the NK activity of the initial strain from 2240.67 U/mL to 5529.56 U/mL by carbon ion beam irradiation [10,18]. Atmospheric and room temperature plasma (ARTP) is a novel physical mutagenesis technique that can generate a stronger DNA damaging ability than traditional methods. The application of ARTP mutagenesis has enhanced the enzyme activity and yield of other strains including the ability of *Aspergillus niger* to produce salt-tolerant proteases [19], the catalytic activity of phospholipase D in *Streptomyces hiroshimensis* [20], and the high production of glutathione or D-arabinitol in yeasts [21,22]. Therefore, the objective of this study was to obtain a food safe and high-yielding NK strain through ARTP mutagenesis.

The analysis of genomics and transcriptomics of the mutated strain was carried out to examine the metabolic pattern of the target and underlying causes of the alterations in strain characteristics [23]. Zhang et al. demonstrated that the mutations in the *alsT* and *ykvZ* genes are responsible for the increased expression of protease and amylase in *Bacillus licheniformis* using genome resequencing [24]. The transcriptomic analysis conducted by Liu et al. elucidated the discrepancies in NK activity across diverse culture media and the interconnection between NK synthesis and substrate metabolism [25]. Moreover, genomic and transcriptomic information establishes the foundation for next-generation chassis engineering, enabling rational design of superior microbial strains with enhanced corresponding phenotypes [22].

This study presents a genetically stable *B. subtilis* JNC002.001 obtained through ARTP mutagenesis of the initial strain SD2, demonstrating enhanced potential for NK production and food fermentation applications. Integrated genomic and transcriptomic analyses subsequently identified key genomic and transcriptional signatures underlying the high-yielding phenotype, providing insights for rational optimization of a NK-producing microbial chassis.

## 2. Materials and Methods

### 2.1. Strain and Culture Conditions

Seven commercially available fermented soybean products, including natto (YJ, BHD, SD, ZW), tempeh (CSDC, JXDC) and Doushen (DS), were used to screen for the wild-type strains with high NK production as initial strains for mutagenesis. Both the selected wild-type strain *B. subtilis* SD2 from commercially available natto and the mutant strain *B. subtilis* JNC002.001 were stored in our laboratory. The mutation was deposited in the China Center for Type Culture Collection (CCTCC; Wuhan, China) under the number CCTCC M-20231585.

LB medium (1% peptone, 0.5% yeast extract, 1% NaCl) was used to obtain fresh seed solution. Ae single-colony, isolated from the strain, was inoculated into 4 mL of LB medium at 37 °C with shaking for 12 h at 200 rpm. Fermentation medium (2% glucose, 1% peptone from soya, 72 mM K_2_HPO_4_, 17 mM KH_2_PO_4_, 0.05% MgCl_2_, 0.02% CaCl_2_) was used to enable *B. subtilis* to express NK [17]. The seed solution was transferred into a 250 mL flask containing 50 mL of fermentation medium at a 2% ratio with the same culture conditions as above. Casein medium (0.3% casein, 0.1% glucose, 0.1% yeast extract, 0.1% K_2_HPO_4_, 0.05% KH_2_PO_4_, 0.01% MgCl_2_, and 2% agar) was used to screen the protease-producing strains.

### 2.2. ARTP Mutagenesis

The ARTP mutagenesis procedure was performed with slight modifications based on the method described by Zhang et al. [24]. In brief, the seed culture was centrifuged (10,000× *g*, 2 min), and the pellet was resuspended in sterile saline to achieve an OD_600_ value of 0.6–0.8. The cell suspension (10 μL) was spread onto a metal carrier plate and subjected to mutagenesis using the ARTP-IIS (Wuxi TMAXTREE Biotechnology Co., Ltd., Wuxi, China). *B. subtilis* SD2 was treated for 0, 15, 30, 45, 60, 90, 120, 150, or 180 s. After treatment, the cells were eluted with sterile saline, serially diluted (10^−1^ to 10^−3^), and plated onto casein medium, followed by incubation at 37 °C for 24 h. The lethality rate was calculated according to Wang et al. [17], and the optimal mutagenesis time was determined accordingly.

### 2.3. Determination of the Enzymatic Activity of Nattokinase (NK)

#### 2.3.1. Fibrin Plate Analysis

Fibrin plate analysis was performed as described by Liu et al. [13], with slight modifications. In brief, 10 mL of 1.5% agarose solution and 10 mL of 1.5 mg/mL bovine fibrinogen solution (0.01 mol/L PBS buffer, pH 7.5) were incubated at 50 °C. After adding and mixing 1 mL of thrombin solution (2 U/mL in 0.9% saline) to the agarose solution and fibrinogen solution system, the plate was allowed to cool to room temperature. Holes (diameter 2 mm) were punched in the fibrin plate followed by the addition of 10 μL of fermentation supernatant in each hole. The NK activity was detected according to the size of the lysis circle after 18 h at 37 °C.

#### 2.3.2. Fibrinolytic Activity Determination

The NK activity, expressed in fibrinolytic units (FU), was determined according to the method established by the Japan Nattokinase Association (https://j-nattokinase.org/jnka_nk_english.html accessed on 29 April 2023). In brief, 1.4 mL Tris-HCl (0.05 M, pH 8.0) was mixed with 0.4 mL fibrinogen solution (0.72%) and incubated at 37 °C for 5 min. After adding 0.1 mL thrombin solution (20 U/mL) and incubating at 37 °C for 10 min, 0.1 mL diluted sample was introduced, followed by a 60-min incubation at 37 °C. Finally, 2 mL trichloroacetic acid solution (0.2 M) was added and the mixture was incubated at 37 °C for 20 min to stop the reaction. After centrifugation (15,000× *g*, 10 min), the absorbance of the supernatant was measured at 275 nm. One unit (1 FU) was defined as the amount of the enzyme that increased the absorbance of the filtrate at 275 nm by 0.01 per minute under the conditions specified in the procedure [10].

### 2.4. Strain Screening Methods

The mutant strains with the largest transparent circles on casein medium were selected for seed liquor culture and fermentation (36 h). Mutant strains with significantly increased NK yield were screened by the fibrin plate analysis and fibrinolytic activity determination. The fibrin plate analysis was used as preliminary screening, while the fibrinolytic activity determination was the final screening to obtain the strains with reliable high NK activity from the initial screened strains.

### 2.5. Determination of the Genetic Stability of Mutant Strain

The strain was streaked onto LB medium and incubated at 37 °C for 24 h as one passage cycle. In this experiment, a total of 30 successive streak passages were conducted for the mutant strain. Flask fermentation was carried out in every 10 generations. The NK activity of the fermentation broth (36 h) was measured to detect a possible decline of the enzyme-producing ability.

### 2.6. Determination of the Indicators of the Fermentation Progress

Cell density was determined at 600 nm using an UV spectrophotometer (UV-5500, METASE, Shanghai, China). The fermentation broth was centrifuged at 10,000× *g* for 10 min at room temperature to collect the supernatant. The glucose content in the supernatant was determined using a M-100 biosensors analyzer (SIEMAN, Shenzhen, China); SDS-PAGE analysis was performed as described by Liu et al. [13] to detect the production of NK in the culture supernatant, with 20 μL loaded into each lane. The sporulation efficiency was determined according to Tian et al. [26]. In brief, 100 μL of each cell suspension was incubated at 80 °C for 20 min, then plated on LB plates for spore CFU counting. Equal volumes of untreated suspensions were similarly plated for the total cell CFU counts. The sporulation efficiency was calculated as the percentage of the spore count to the total number of viable cells.

The fermentation progress of *B. subtilis* SD2 and *B. subtilis* JNC002.001 was monitored and compared. The OD_600_ and residual sugar content were measured every 4 h. After the change in OD_600_ stabilized, the measurement was adjusted to every 12 h. Then, no further measurement of residual sugar was performed when its content dropped below 0.1 g/L. The NK activity and production were measured every 12 h. The sporulation efficiency at 36 h of fermentation was determined.

### 2.7. Whole Genome Sequencing and Functional Annotation

The genomic DNA of *B. subtilis* SD2 was sequenced on the Illumina/PacBio sequencing platform by Genedenovo Biotechnology Co., Ltd. (Guangzhou, China). Genomic DNA was extracted using a commercial DNA isolation kit (TianGen, Beijing, China) from 50 mL of the overnight cultures grown in LB medium inoculated with a single *B. subtilis* SD2 isolate. The DNA quality was detected using a Qubit (Thermo Fisher Scientific, Waltham, MA, USA) and a Nanodrop (Thermo Fisher Scientific, Waltham, MA, USA) accordingly. Qualified genomic DNA was first sonicated randomly, and then end-repaired, A-tailed, and adaptor ligated. DNA fragments with a length of 300–400 bp were enriched by PCR. After the PCR product purification and library validation, the genome was sequenced with Illumina HiSeq. For the PacBio sequencing, the qualified genomic DNA was fragmented with G tubes and end-repaired to prepare SMRTbell DNA template libraries (with a fragment size of >10 Kb). After detecting library quality, the SMRT sequencing was performed. Continuous long reads were attained from the SMRT sequencing runs and used for de novo assembly using Falcon v0.3.0 [27]. The clean reads from the Illumina platform were used to correct the genome sequences to determine the final genome sequences using Pilon v1.23 [28].

The open reading frames were predicted using the Prokka v1.11 [29] or NCBI (the National Center for Biotechnology Information) prokaryotic genome annotation pipeline [30]. Noncoding RNAs, such as rRNAs, were predicted using rRNAmmer v1.2 [31]. The tRNAs were identified by tRNAscan v1.3.1 [32], while sRNAs were identified by cmscan v1.1.2 [33]. The genes were annotated by aligning with the deposited ones in diverse protein databases, including the NCBI non-redundant protein sequence database, UniProt/Swiss-Prot, the Kyoto Encyclopedia of Genes and Genomes (KEGG), Gene Ontology (GO), and Cluster of Orthologous Groups of proteins [34]. The complete genome sequence of *B. subtilis* SD2 was deposited in GenBank with the accession number CP163447 (https://www.ncbi.nlm.nih.gov/nuccore/CP163447.1/ (accessed on 5 August 2024)).

### 2.8. Genome Resequencing

In order to obtain the differences between the *B. subtilis* JNC002.001 and SD2 genome sequences, genome resequencing was performed using the *B. subtilis* SD2 genome sequence as a reference. The genomic DNA of *B. subtilis* JNC002.001 was extracted to perform the genomic sequence analysis using the Illumina sequencing platform (MajorBio Co., Shanghai, China). The filtered high-quality reads were mapped against the *B. subtilis* SD2 genome (GenBank_ CP163447) using BWA-MEM [35]. Then Snippy v4.6.0 (https://github.com/heilaaks/snippy, accessed on 25 August 2024) was used to call single nucleotide polymorphisms (SNPs), insertions/deletions, and other information. It was also used to clean out the sites with low sequencing depth and comparison quality value. The functional annotation of selected polymorphisms was performed using the variant annotation and effect prediction tool SnpEff software (https://pcingola.github.io/SnpEff/, accessed on 25 August 2024) [24]. Primers were designed for the PCR validation of mutations in the resequencing results and are listed in Table S1. The genes that are upstream and downstream of mutation sites in intergenic regions were queried in DBTBS (http://dbtbs.hgc.jp/, accessed on 1 November 2024) for transcription factors and binding sites associated with them.

### 2.9. Transcriptome Sequencing, Annotation and Analysis

The transcriptome sequencing of *B. subtilis* SD2 and JNC002.001 was performed on the Illumina/PacBio sequencing platform by Genedenovo Biotechnology Co., Ltd. (Guangzhou, China). The total bacterial RNA was extracted from *B. subtilis* SD2 and JNC002.001 at 36 h of fermentation. After sample preparation, the sequencing libraries were sequenced on the Illumina NovaSeq X Plus platform. Clean reads were obtained by further filtering using FASTP v0.20.0 [36]. Clean reads were mapped to the reference genome using Bowtie2 v2.2.8 [37]; reads mapped to ribosome RNA were removed. The retained reads were aligned with the reference genome using Bowtie2 v2.2.8 and gene expression was calculated using RSEM v1.2.19 [38]. The correlation coefficient was determined and principal component analysis was performed to reveal the relationship between samples. The gene expression level was normalized by using the FPKM method. Genes with a fold change ≥ 2 and FDR (false discovery rate) less than 0.05 were considered differentially expressed genes (DEGs). The DEGs were analyzed in GO and KEGG functions, with FDR < 0.05 as threshold [39].

### 2.10. Statistical Analysis

All experiments were performed in triplicate and the obtained data are represented as mean ± standard deviation. The data were analyzed by one-way analysis of variance followed by Duncan’s multiple comparisons tests using SPSS Statistics 22.0 with the level of significant difference set to *p* < 0.05.

## 3. Results and Discussion

### 3.1. Strains Screening and ARTP Mutagenesis

**Figure 1:**
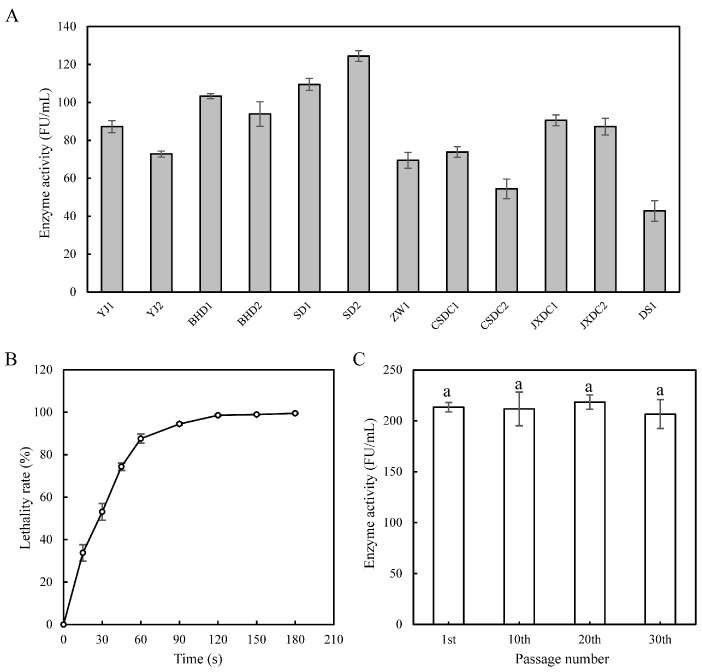
Screening of the initial strains with high NK activity and ARTP mutagenesis correlation maps. (**A**) NK activity of the wild-type strains from different fermented soybean products. (**B**) Lethality rate of *B. subtilis* SD2 by ARTP. (**C**) NK activity of the mutant strain JNC002.001 at various passage numbers, ^a^ *p* < 0.05.

The results of the initial strains’ screening are shown in Figure 1A. These strains were selected based on the formation of transparent zones on casein plates, further cultured for fermentation for 36 h, and then subjected to enzyme activity measurement of the fermentation broth. The strain SD2 is a wild-type strain with high NK-producing ability (124.4 ± 2.8 FU/mL). It exhibited the highest degree of homology with *B. subtilis* through strain identification. As shown in Figure 1B, the lethality rate of *B. subtilis* SD2 (hereafter referred to as SD2) increased with the increase of ARTP mutagenesis duration. It is usually considered more favorable to obtain ideal mutant strains under the condition that the mutagenic lethality rate is about 90% [40]. Thus, the ARTP mutagenesis duration was set to 60 s. In this study, a total of 178 mutant strains were selected on casein medium and inoculated into fermentation medium. Thirty-one mutants with larger diameters of the fibrinolytic ring zone than SD2 were initially screened by fibrin plate analysis and then re-screened by fibrinolytic activity determination. Finally, a mutant strain demonstrating a 71.5% increase in NK activity was obtained and designated *B. subtilis* JNC002.001 (hereafter referred to as JNC002.001). Since the mutant strain may suffer from phenotypic delay phenomenon or undergo self-repair [41], the genetic stability of the strain was verified. Figure 1C shows that JNC002.001 maintained the stability of NK production ability in the 10th, 20th, and 30th generations in flask fermentation. This indicates that JNC002.001 has stable genetic characteristics.

The ARTP mutagenesis strategy employed in this study demonstrated significant effectiveness in enhancing the NK-producing capability of *B. subtilis*. The mutant strain JNC002.001 was successfully obtained, exhibiting a 71.5% increase in NK activity, which highlights the efficiency of ARTP mutagenesis. Moreover, JNC002.001 demonstrated stable NK production over 30 passages of flask fermentation, indicating its great potential for large-scale NK production.

### 3.2. Comparison of the Fermentations of the Initial and Mutant Strains

**Figure 2:**
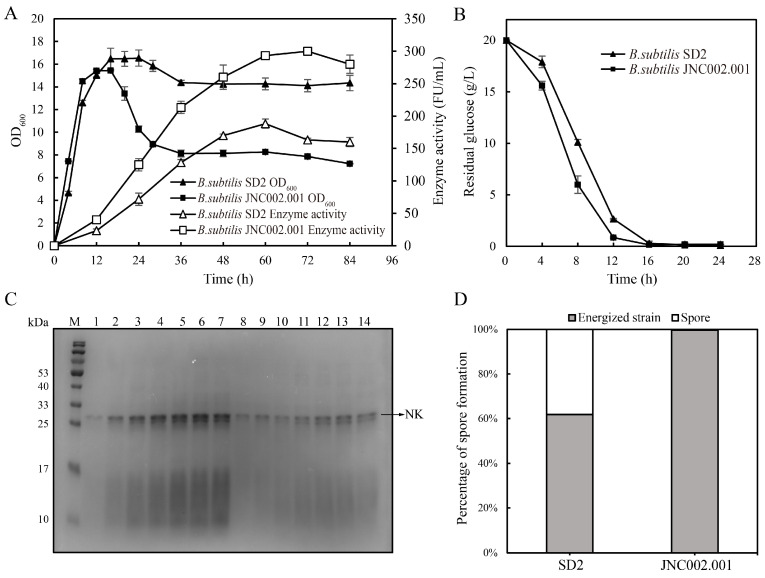
Comparison of the fermentation process between *B. subtilis* SD2 and JNC002.001 in shake flasks. (**A**) Growth curve and enzyme activity. (**B**) Residual glucose. (**C**) SDS-PAGE analysis. Supernatant (20 μL) was loaded into each lane. Lane M denotes protein molecular broad marker. Lane 1–7 and 8–14 denote, respectively, the protein bands of JNC002.001 and SD2 after 12, 24, 36, 48, 60, 72, and 84 h of culture. (**D**) Percentage of spore formation.

Figure 2 shows that the initial and mutant strains were significantly different during flask fermentation. Although the OD600 of SD2 and JNC002.001 increased rapidly from 0 to 8 h, the growth rate and glucose consumption of JNC002.001 were greater than that of SD2 (Figure 2A,B). The OD600 values of the two strains were similar and both reached the peak when the glucose was completely consumed at 16 h. Subsequently, the OD600 of SD2 decreased slightly, while that of JNC002.001 declined sharply before stabilizing at 36 h of fermentation. The NK activity of JNC002.001 was always higher than that of SD2. The maximum NK activity of JNC002.001 was 300.0 ± 4.7 FU/mL at 72 h, which was 1.8 times higher than that of SD2. The NK production of JNC002.001 was significantly higher than that of SD2 after 36 h fermentation (Figure 2C). The sporulation efficiency of SD2 and JNC002.001 was 38.13% and 0.33%, respectively (Figure 2D), indicating that JNC002.001 exhibited impaired sporulation following ARTP mutagenesis.

The differences in growth curves and glucose consumption between SD2 and JNC002.001 suggest that ARTP mutagenesis may have altered the metabolic pathways of the mutant strain. The higher growth rate and glucose consumption of JNC002.001 could be attributed to enhanced metabolic efficiency, potentially through modifications in glycolysis or the TCA cycle. The sharp decline in OD600 of JNC002.001 after glucose depletion, compared to the gradual decrease observed in SD2, may be linked to its impaired sporulation efficiency. Sporulation is a survival strategy for *B. subtilis* under nutrient-limited conditions [26], and the inability of JNC002.001 to form spores effectively likely resulted in cell death and a rapid reduction in OD600. This phenomenon is consistent with the findings of Zhou et al. [42]. In their study, the sporulation-deficient strains exhibited a sharp decline in OD600 during the late fermentation phase, accompanied by a prolonged stable phase of enzyme production and increased protease activity, which aligns well with our results. Therefore, although the reduced sporulation efficiency of JNC002.001 may compromise its survival in harsh environments [26], it could be advantageous for NK production. The impaired sporulation in JNC002.001 may have diverted cellular resources from sporulation to secondary metabolite production, thereby enhancing NK synthesis [43]. Moreover, lower sporulation rates can prevent the loss of metabolic activity during prolonged fermentation, thereby maintaining higher NK production.

### 3.3. Genomic Analysis

#### 3.3.1. *B. subtilis* SD2 Genome Characterization and Functional Classification

The genome of SD2 consisted of a circular chromosome with a length of 4,120,963 bp and a GC content of 43.47%. The genome contained 4051 protein-coding sequences (CDSs), *87 tRNA* genes, and *30 rRNA* genes. The functional categories of Cluster of Orthologous Groups of proteins, location of CDSs, and the number of each functional gene are shown in Figure S1A,B.

#### 3.3.2. Resequencing Results of *B. subtilis* JNC002.001

All reads were aligned with the reference genome of the initial strain SD2. The PCR-verified correct variants found in the genome of JNC002.001 are listed in Table 1. Eight SNPs were generated in seven CDSs, including five missense mutations (*acoA, kinA, gltA, comC, yvyF*) and three synonymous mutations (*gltA, ganP, mdxK*): two SNPs and one insertion (Ins) generated in intergenic region. Through a search in the DBTBS database, the Ins was found in to be located in the promoter of the *mmgA* gene.

The missense mutations lead to the changes in amino acids that may affect the transcription and translation processes, alter the functional activity of the encoded proteins, and thus change life activities [43,44]. Among the CDSs with missense mutations, the *acoA* gene is involved in energy production and conversion, which promotes the degradation of acetoin. Acetoin is a key metabolic product in *B. subtilis* [45]. Therefore, the *acoA* gene mutation might alter metabolic or energy pathways in JNC002.001, indirectly modulating the NK expression. The *kinA* gene, associated with signal transduction, encodes sporulation kinase A. This kinase phosphorylates Spo0A, a key regulatory protein that directly controls spore formation and indirectly affects the transcription of the *aprN* gene [46]. The *gltA* gene is involved in amino acid transport and metabolism. GltA (the protein encoded by *gltA*) and GltB form glutamate synthase, which catalyzes the production of glutamate from α-ketoglutarate and L-glutamine. Glutamate has been reported to promote the NK synthesis [47]. The *comC* gene, encoding the pseudopilin signal peptidase (ComC), is an important gene in the Com secretion pathway, but is not associated with the NK secretion [48]. There is less information on the *yvyF* gene to speculate whether it has an effect on the NK expression. Because of the low codon preference of *B. subtilis*, the effect of synonymous mutations was negligible in this study [49]. The *mmgA* gene is associated with sporulation. Mutations that occur in intergenic regions may have an effect on transcription if they are located at the promoters or terminators. In summary, mutations in the *acoA*, *kinA*, and *gltA* genes in JNC002.001 may affect NK expression, while mutations in the *kinA* and *mmgA* genes may be responsible for the reduced sporulation efficiency.

### 3.4. Transcriptomic Analysis

#### 3.4.1. Quality Evaluation of the RNA Sequencing and Assembling

**Figure 3:**
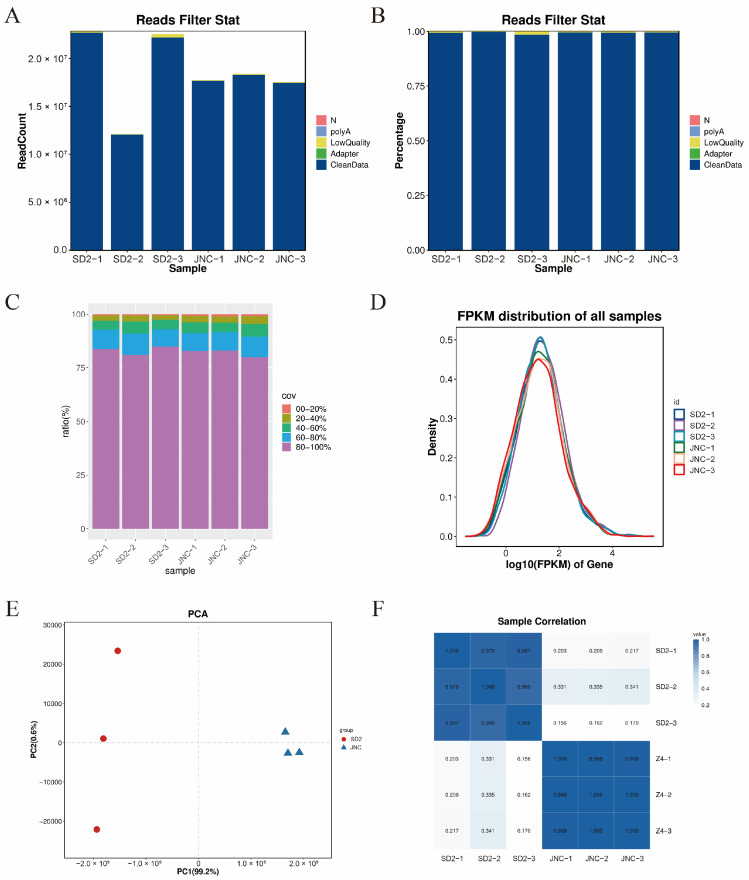
Sequence statistics of the transcriptome data. (**A**) Reads count filtering. (**B**) Read filtering (%). (**C**) Distribution of the known gene coverage (%). (**D**) Density plot of FPKM. (**E**) Principal component analysis. (**F**) Sample correlation heatmap analysis.

Transcriptome changes in the initial and mutant strains were analyzed by RNA sequencing. Samples used to produce the raw and clean data are shown in Table S2 (SD2-1, SD2-2, SD2-3, JNC002.001-1, JNC002.001-2, JNC002.001-3), where the Q20 and Q30 of each group were higher than 90%. The number and percentage of raw and clean reads, as represented (Figure 3A,B) show that a larger proportion of the reads were identified as clean reads, with a very small proportion of polyA, low quality, N, and adapter reads. The gene coverage statistics (Figure 3C) show the sequencing depth and homogeneity of the sample data. It revealed that more than 80% of the genes had a coverage of 80% to 100%, while most of the transcribed genes could be detected. Figure 3D reflects the similar distribution of gene expression abundance across the samples. The principal component analysis (Figure 3E) demonstrates a significant separation of the two transcriptomes, with 99.2% and 0.6% variation for PC1 and PC2, respectively. The correlation analysis (Figure 3F) reveals a significant positive correlation between the parallel groups of the strains SD2 and JNC002.001.

#### 3.4.2. Statistics Analysis of the DEGs

**Figure 4:**
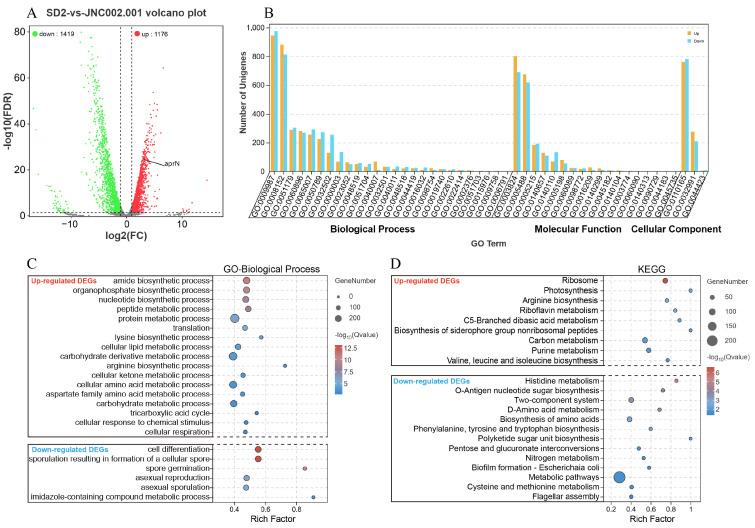
The DEGs between *B. subtilis* SD2 and JNC002.001, with GO and KEGG analysis of the DEGs. (**A**) DEGs volcano plot. (**B**) GO classification of the differential genes. The name and details of a GO term can be queried in the GO database according to the GO ID. (**C**) GO enrichment analysis statistics chart of the up-regulated and down-regulated genes. (**D**) KEGG enrichment analysis statistics chart of the up-regulated and down-regulated genes. The bubble color indicates the significance of enrichment, as demonstrated in Figure 4 (**C**,**D**).

The results of the transcriptome sequencing showed that there were 2595 DEGs in the mutant strain JNC002.001 compared to the initial strain SD2, of which 1419 genes were significantly down-regulated and 1176 genes were significantly up-regulated. Visual analysis of the DEGs is shown in Figure 4A (see Table S3 for detailed transcriptional data). Table 2 shows that the *aprN* gene was up-regulated 9.4 times and other extracellular protease genes (*nprE*, *vpr*, *bpr*, *wprA*) were also significantly up-regulated. Meanwhile, the intracellular protease genes (*aprX*, *isp*) were significantly down-regulated [50]. The gene with the highest expression in SD2 was *cgeB* (spore maturation protein), followed by the *aprX* gene, whereas the gene with the highest expression in JNC002.001 was *aprN*, indicating that JNC002.001 is an excellent strain for NK production.

#### 3.4.3. GO and KEGG Analysis of the DEGs

GO and KEGG function annotation analyses were conducted to obtain information on the biological functions of the DEGs. Level 2 GO terms (Figure 4B) containing more DEGs included cellular process (GO:0009987), metabolic process (GO:0008152), catalytic activity (GO:0003824), binding (GO:0005488), and cellular anatomical entity (GO:0110165). The partial GO and KEGG enrichment for up-regulated genes and down-regulated genes are shown in Figure 4C and Figure 4D, respectively. There were 201 significantly enriched GO terms (Q < 0.05, the same below) in the up-regulated genes (Table S4), including the metabolic processes of carbohydrates and derivatives, nucleotides, proteins, cellular lipid, ketones, the biosynthetic processes of organophosphate and amides, respiration, translation and cell envelope Sec protein transport complex, etc. The down-regulated genes produced 14 significantly enriched GO terms, which were mainly associated with sporulation. The KEGG enrichment showed that the up-regulated genes were enriched significantly only in ribosome pathway, while the down-regulated genes produced six significantly enriched KEGG pathways, including histidine metabolism, O-antigen ribose biosynthesis, two-component system, D-amino acid metabolism, biosynthesis of amino acid, and phenylalanine, tyrosine, and tryptophan biosynthesis. The GO analysis showed accelerated substance metabolism and down-regulation of the sporulation genes in the mutant strain, which is consistent with increased glucose consumption and reduced sporulation efficiency (Figure 2B,D).

### 3.5. Pathway Analysis of the Key DEGs

Based on the changes in the characteristics of the mutant strain, genome resequencing, and the GO and KEGG enrichment, the DEGs in central carbon metabolism, the *aprN* gene transcription process, sporulation, and NK transport and folding were further analyzed and discussed as follows.

#### 3.5.1. Analysis of the DEGs in Central Carbon Metabolism

**Figure 5:**
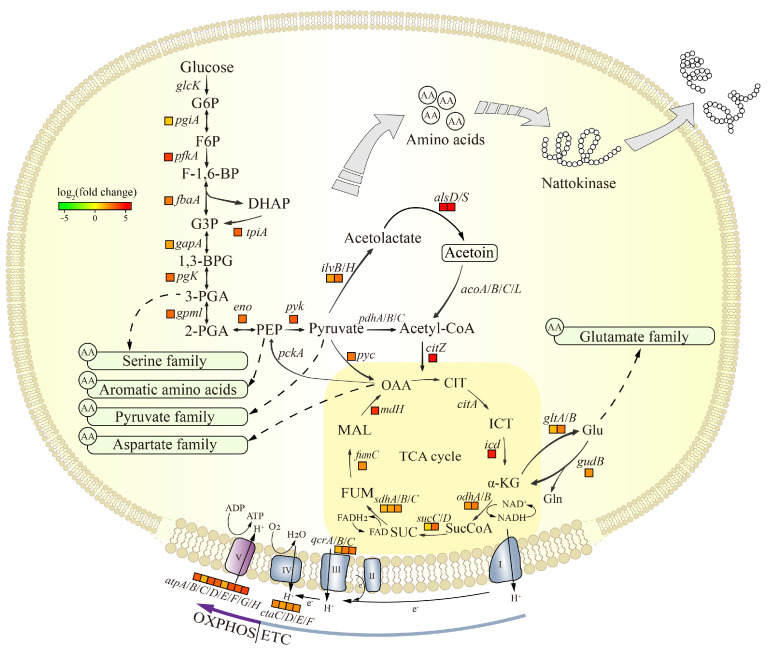
Overview of the key genes regulatory map and related pathways associated with central carbon metabolism. The small squares next to gene names refer to the comparison of *B. subtilis* JNC002.001 with *B. subtilis* SD2 gene expression. The color of the small squares changes from green to red with the variation of gene expression from down- to up-regulation. The absence of small squares indicates that there was no significant change in the gene expression. G6P: glucose-6-phosphate; F6P: fructose-6-bisphosphate; F-1,6-BP: fructose-1,6-bisphosphate; G3P: glyceraldehyde-3-phosphate; DHAP: dihydroxyacetone phosphate; 1,3-BPG: 1,3-bisphosphoglycerate; 3-PGA: 3-phosphoglycerate; 2-PGA: 2-phosphoglycerate; PEP: phosphoenolpyruvate; CIT: citrate; ICT: isocitrate; α-KG: α-ketoglutarate; SucCoA: succinyl CoA; SUC: succinate; FUM: fumarate; MAL: malate; OAA: oxaloacetate; Glu: glutamic; Gln: glutamine; ETC: electron transport chain; OXPHOS: oxidative phosphorylation; I: NADH dehydrogenase; II: succinate dehydrogenase; III: cytochrome bc1 complex; IV: cytochrome C oxidase; and V: ATP synthase.

Glycolysis, the TCA cycle, the electron transport chain (ETC) and oxidative phosphorylation (OXPHOS) in central carbon metabolism were considered. Figure 5 and Table S3 show the change in expression of the genes involved in these processes. The *pfkA* gene was up-regulated 14 times in JNC002.001, encoding phosphofructokinase, the rate-limiting enzyme of glycolysis. The expression of genes involved in glycolysis such as *fbaA*, *tpiA*, *gapA*, *pgk*, *gpml*, *eno*, and *pyk* were also significantly up-regulated. The transcript levels of citrate synthase (*citZ*), isocitrate dehydrogenase (*icd*), and malate dehydrogenase (*mdh*) in the TCA cycle were up-regulated 28.4, 20.9, and 16.8 times, respectively. In addition, the α-ketoglutarate dehydrogenase complex (*sucA*, *odhB*), succinyl-CoA synthase (*sucD*), succinate dehydrogenase (*sdhA*/*B*/*C*), and fumarase (*fumC*) were also up-regulated. Large amounts of NADH are generated in the TCA cycle and subsequently utilized for ATP production. The ETC and OXPHOS are the metabolic pathways of NADH. As shown in Figure 5, the genes encoding cytochrome C reductase (*qcrA*/*B*), cytochrome C (*qcrC*), cytochrome C oxidase (*ctaC*/*D*/*E*/*F*), and the F0F1 ATP synthase complex (*atpA*/*B*/*C*/*D*/*E*/*F*/*G*/*H*) in the ETC and OXPHOS were up-regulated. As a result, central carbon metabolism in JNC002.001 was enhanced, and more energy was available for vital activities compared to SD2.

Central carbon metabolism is not only the main source of energy required by organisms, but also provides precursors for the synthesis of amino acids. Glycolysis and the TCA cycle intermediates such as glyceraldehyde-3-phosphate, pyruvate, phosphoenolpyruvate, α-ketoglutarate, and oxaloacetate are precursors for the synthesis of a variety of amino acids. Chen et al. reported that the addition of aspartate and glutamate to the culture medium increased the production of NK, where glutamate was the limiting factor for NK production in *B. subtilis* [47]. Oxaloacetate is the precursor of the aspartate family amino acids. The up-regulated expression of the *pyc* gene in JNC002.001 indicated an increased conversion of pyruvate to oxaloacetate, which coincided with the significant enrichment of aspartate family amino acids metabolic process in the GO analysis (Figure 4C and Figure 5). The glutamate is converted from α-ketoglutarate by glutamate synthase (*gltA*/*B*), which is the only pathway for glutamate synthesis in *B. subtilis*. Notably, citrate synthase, isocitrate dehydrogenase, and α-ketoglutarate dehydrogenase (*odhA*/*B*) are the key enzymes in TCA cycle. The up-regulated levels of the first two were much greater than α-ketoglutarate dehydrogenase in JNC002.001 (Figure 5), which may imply that more α-ketoglutarate was involved in the metabolism process of other substances. Meanwhile, the up-regulation of glutamate synthase (*gltA*/*B*) in the mutant strain indicated an increased metabolic flux from α-ketoglutarate to glutamate. It shows that the enhancement of the central carbon metabolism in JNC002.001 provides a suitable material basis for the NK synthesis.

Genome resequencing revealed a missense mutation and a synonymous mutation in the *gltA* gene. In a previous study, *B. subtilis* responded to any mutation that interfered with glutamate metabolism in order to bring glutamate supply into balance [51]. Therefore, whether the mutations are associated with changes in the expression of glutamate synthase and glutamate dehydrogenase (*gudB*) is unclear and needs to be further investigated.

#### 3.5.2. Analysis of the DEGs in the aprN Gene Transcription Process

**Figure 6:**
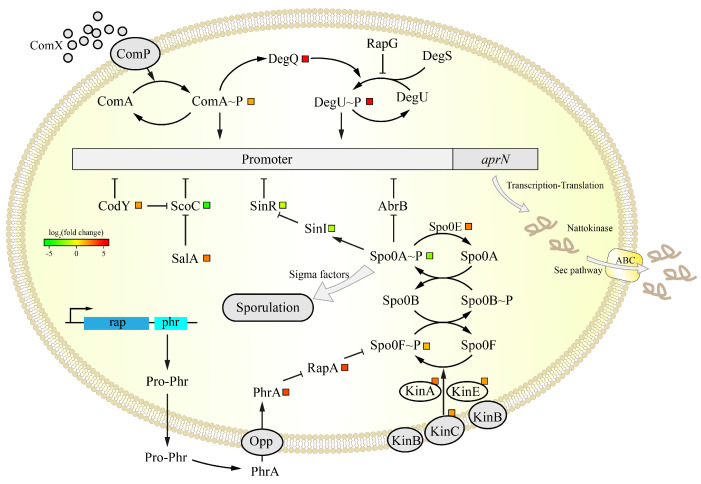
Overview of the *aprN* regulatory factors map and related pathways associated with signal transduction in transcriptional analysis. The color scheme and interpretation of the small squares follow the conventions established in Figure 5.

The transcription of the *aprN* gene is co-regulated by multiple transcription factors in *B. subtilis*, which also interact with each other, resulting in a complex regulatory process [46]. The transcription process of the *aprN* gene is shown in Figure 6. The global transcriptional regulators AbrB and CodY are important inhibitors, as well as key elements indispensable for *aprN* gene transcription [52,53]. ComA, DegU, and Spo0A are master transcriptional regulators and are active after being phosphorylated by the corresponding specific kinases [54]. ComA–P and DegU–P can directly promote the transcription of the *aprN* gene, while Spo0A–P exerts and indirect regulatory effect by inhibiting *abrB* [25,46]. Moreover, SinR and ScoC are also able to inhibit NK expression, with SinR being indirectly inhibited by Spo0A–P [55] and ScoC being inhibited by CodY and SalA [53,56].

The specific gene expression values are shown in Table 3. In JNC002.001, the change in expression of the *abrB* and *comP* genes was not obvious. The *comA*, *degQ* and *degU* genes showed significant up-regulation, while the *sinR* and *scoC* genes showed the opposite change. This favored the expression of NK. Unexpectedly, the *codY* gene was up-regulated. This may be attributed to the fact that the ScoC is one of the primary inhibitors of *aprN* gene transcription at this stage. Consequently, the up-regulation of the *codY* and *salA* genes would inhibit the ScoC synthesis and thus promote *aprN* gene transcription. The changes in the expression of these transcription factors resulted in significant up-regulation of the *aprN* gene.

#### 3.5.3. Analysis of the DEGs in Sporulation

As shown in Figure 6, Spo0A is a key regulatory protein for sporulation, which is phosphorylated and activated by a combination of sporulation kinases (KinA/B/C/D/E) and phosphotransferases (Spo0F, Spo0B). Spo0E and RapA specifically dephosphorylate Spo0A–P and Spo0F–P, respectively, and negatively regulate the sporulation initiation pathway [57]. PhrA is transcribed with RapA and can inhibit the action of RapA. The different stages of the sporulation process are also regulated by some sigma factors, such as SigE/SigK in the mother cell and SigF/SigG in the forespore [58]. The expressions of genes related to sporulation are shown in Table 3. In JNC002.001, *spo0A* was significantly down-regulated compared to the wild-type strain SD2. Though *spo0F*, *kinA*/*C*/*E*, and *phrA*, which promote phosphorylation of Spo0A, were up-regulated, *spo0E* and *rapA*, which have the opposite effect, were also up-regulated. It is interesting to note that the PhrA peptides are imported back into the cell and bind to their cognate RapA phosphatase only at high cell density [54]. According to Figure 2A, the cell density of JNC002.001 was lower than that of SD2 during fermentation, so this relatively limited the inhibitory effect of the PhrA peptide. The *sigE*, *sigK*, and *sigG* genes were all down-regulated. The change in *spo0B*, *kinA*/*D*, and *sigF* expressions were not obvious. As for the other spore protein genes, *cegB*, *cotG*, and *cotX* were down-regulated significantly. The transcriptomic data showed impaired sporulation in JNC002.001, which was consistent with the assay results (Figure 2D).

The correlation between gene mutations and reduced sporulation efficiency in this study requires further validation. In combination with genome resequencing, it can be inferred that the mutation in the *kinA* gene may be responsible for the impaired sporulation process in JNC002.001. In previous studies, mutations in the *kinA* gene were detrimental to sporulation and were accompanied by up-regulated expression of the *aprN* gene. A *Bacillus pumilus* strain with high expression of the *aprE* gene was obtained by mutagenesis, in which the *kinA* gene was mutated and up-regulated and the *sigG*/*K* genes related to sporulation were significantly down-regulated [55]; a high-yielding NK mutant obtained by Sheng et al. also had mutations in genes related to sporulation (*kinA, oppA*, *appA*, *spoIIP*) [18]. It should be noted that the *mmgA* gene is also associated with sporulation, and was observed to be down-regulated (Table 4). However, the mutation in the promoter of the *mmgA* gene was not identified as the primary factor, as the gene is expressed at a late stage of sporulation and the SigE protein, which regulates this gene, was also down-regulated.

#### 3.5.4. Analysis of the DEGs in NK Transport and Folding

NK is an extracellular protease that is secreted extracellularly via the Sec pathway in post-translational translocation [48]. In the Sec secretion pathway, the SecYEG, SecDF-YajC, and YidC proteins constitute a complete protein transporter structure on the membrane. SecA not only guides proteins into the SecYEG channel, but also acts as an ATPase to energize protein translocation [59]. As shown in Table 4, the *secA*, *secY*, *secG*, *secDF*, and *yrbF* (YajC) genes were all significantly up-regulated in JNC002.001, which facilitated the translocation of NK. In addition, the pro-folding factor PrsA, which enables rapid folding of proteins secreted into the extracellular space to reduce degradation by protein hydrolases, was also significantly up-regulated [60]. The up-regulation of these genes facilitated the secretion and folding of NK and improved its stability in the fermentation broth.

## 4. Conclusions

A wild-type *B. subtilis* SD2 isolated from natto was successfully mutated by ARTP mutagenesis to obtain the strain named as *B. subtilis* JNC002.001 with high-yielding NK. The strain JNC002.001 showed reliable genetic stability and a significant reduction in sporulation efficiency. The NK activity of JNC002.001 was 1.84 times higher than that of the initial strain SD2. Genome resequencing revealed that the mutant strain JNC002.001 exhibited 10 SNPs and one Ins, in which the *kinA and gltA* genes were associated with sporulation and NK synthesis, respectively. Transcriptomic analysis revealed 2595 DEGs at the transcriptional level, with the *aprN* gene displaying a 9.7-fold increase in expression. GO enrichment analysis showed accelerated substance metabolism and down-regulation of sporulation genes in JNC002.001. The data of the DEGs elucidated the mechanism of the increased expression and secretion of NK in terms of the central carbon metabolism, the regulation of the *aprN* gene, sporulation, and NK transport and folding. This study demonstrated that ARTP mutagenesis is an effective method for modifying strain characteristics. The JNC002.001 strain is promising for the large-scale production of NK and high value-added food applications. In light of these findings, future research could focus on validating the effects of these mutation sites by introducing or knocking out certain genes in the strain. The results of this study can also provide a novel way to construct engineered strains with high-yielding NK.

## Figures and Tables

**Table 1:** Gene mutation summary.

CDS Region Variants					
Gene ID	Gene Name	Annotation	Codon	Amino Acid Mutate	Type
AB5991_04395	*acoA*	2,6-dichlorophenolindophenol oxidoreductase subunit alpha	CGT→CAT	R297H	missense
AB5991_07820	*kinA*	Sporulation kinase A	ATC→GTC	I578V	missense
AB5991_10430	*gltA*	Glutamate synthase [NADPH] large chain	GAA→GAG	E102E	silent
			AAA→GAA	K101E	missense
AB5991_14300	*comC*	Prepilin leader peptidase	GAT→TAT	D172Y	missense
AB5991_18005	*ganP*	Galacto-oligosaccharides transport system permease protein GanP	AAA→AAG	K215K	silent
AB5991_18470	*mdxK*	Maltose phosphorylase	TTA→TTG	L271L	silent
AB5991_18900	*yvyF*	Uncharacterized	CAT→TAT	H78Y	missense
**Intergenic Region Variants**					
**Pos**	**Intergenic region**		**Variant**	**Comment**	
339112	*ycgL–putB*		G to T	No feature found	
2331408	*mmgA–glpQB*		C to CATGA	SigE-binding site	
4120715	*rpmH–dnaA*		T to A	No feature found	

**Table 2:** Transcript-level expression of some of the DEGs in *B. subtilis* SD2 and JNC002.001.

Gene Name	Annotation	SD2_fpkm	JNC002.001_fpkm	log_2_FC (JNC002.001/SD2)
*aprN*	Nattokinase, subtilisin NAT	35,684.56	335,260.20	3.23
*nprE*	Bacillolysin, neutral protease	1049.24	13,915.68	3.73
*vpr*	Minor extracellular protease	108.57	466.81	2.10
*bpr*	Bacillopeptidase F	381.31	2016.95	2.40
*wprA*	Cell wall-associated protease	22.78	587.41	4.69
*aprX*	Serine protease	81,411.65	1665.46	−5.61
*isp*	Intracellular serine protease	47,239.65	19,476.40	−1.28
*cgeB*	Spore maturation protein	97,503.94	3462.53	−4.82

**Table 3:** Changes in the expression of the DEGs in the aprN gene transcription process and sporulation.

Gene Name	Annotation	log_2_FC (JNC002.001/SD2)
*abrB*	Transition state regulatory protein	0.80
*codY*	Global transcriptional regulator	1.98
*comA*	Two-component system response regulator	1.73
*comP*	Sensor histidine kinase	−0.26
*degQ*	Pleiotropic regulator	5.51
*degU*	Two-component system response regulator	5.41
*sinR*	Master regulator of biofilm formation	−1.46
*sinI*	Antagonist of SinR	−1.15
*scoC*	DNA-binding transcriptional repressor	−4.31
*salA*	phosphorylation-dependent transcriptional regulator	2.62
*spo0A*	Stage 0 sporulation protein A	−2.48
*spo0B*	Sporulation initiation phosphotransferase B	−0.22
*spo0F*	Sporulation initiation phosphotransferase F	1.34
*kinA*	Sporulation kinase A	2.79
*kinB*	Sporulation kinase B	0.16
*kinC*	Sporulation kinase C	2.02
*kinD*	Sporulation kinase D	−0.11
*kinE*	Sporulation kinase E	1.87
*spo0E*	Aspartyl-phosphate phosphatase	2.46
*rapA*	Response regulator aspartate phosphatase A	3.71
*phrA*	Phosphatase RapA inhibitor	3.69
*sigF*	Sporulation sigma factor	0.26
*sigE*	Sporulation sigma factor	−1.96
*sigG*	Sporulation sigma factor	−3.53
*sigK*	Sporulation sigma factor	−3.33
*cgeB*	Spore maturation protein	−4.82
*cotG*	Spore coat protein G	−3.36
*cotX*	Spore coat protein X	−1.13
*mmgA*	Acetyl-CoA acetyltransferase	−4.57

**Table 4:** Changes in the expression of the DEGs in NK transport and folding.

Gene Name	Annotation	log_2_FC (JNC002.001/SD2)
*secY*	Protein translocase subunit SecY	1.51
*secA*	Protein translocase subunit SecA	1.94
*secDF*	Protein translocase subunit SecDF	1.71
*secG*	Probable protein-export membrane protein SecG	2.09
*yrbF*	Sec translocon accessory complex subunit YrbF	2.63
*prsA*	Foldase protein PrsA	2.79

## Data Availability

The original contributions presented in this study are included in the article/Supplementary Materials. Further inquiries can be directed to the corresponding author.

## Supplementary Materials

The following supporting information can be downloaded at: https://www.mdpi.com/article/10.3390/foods14050898/s1, Figure S1: Genome sequencing information (A) and gene function classification (B) of *B. subtilis* SD2; Table S1: Primers for confirmation of variants in coding regions of functional genes and intergenic regions; Table S2: Statistics of the base information before and after filtering; Table S3: Transcriptome sequencing results of *B. subtilis* SD2 and JNC002.001; Table S4: GO terms significantly enriched in up-regulated genes.

## Abbreviations

The following abbreviations are used in this manuscript:

ARTP	atmospheric and room temperature plasma
DEG	differentially expressed gene
ETC	electron transport chain
GO	Gene Ontology
Ins	insertion
KEGG	Kyoto Encyclopedia of Genes and Genomes
LB	Luria–Bertani
NK	nattokinase
OXPHOS	oxidative phosphorylation
SNP	single nucleotide polymorphism
