# Supplementary material for: Genomic and Transcriptomic Analysis of Mutant Bacillus subtilis with Enhanced Nattokinase Production via ARTP Mutagenesis
Source: Foods. 2025 Mar 6;14(5):898. doi: 10.3390/foods14050898 (PMC11899143; doi:10.3390/foods14050898)
Supplement: Supplementary file 1 [file foods-14-00898-s001.zip › Table S1.pdf]

Table S1. Primers for confirmation of variants in coding regions of functional genes and intergenic regions.

Gene ID/Pos	Name	Primer	Sequence (5′ to 3′)
AB5991_04395	*acoA*	RE1-1	CGGCTGTATGGAACCTTCCT
		RE1-2	CCTGGTCAAAGCACGTGC
AB5991_07820	*kinA*	RE2-1	CCGCAACCCTCTTACAGCGA
		RE2-2	CACCGAAAGACATTGCAAAGCACAT
AB5991_10430	*gltA*	RE3-1	CTCACGTGCTCTCATCCAG
		RE3-2	GCGGCTTCTGAAACGTG
AB5991_14300	*comC*	RE4-1	CAGCCCAATGCCATTTTG
		RE4-2	CTTCGGGCTTATCCTTGG
AB5991_18005	*ganP*	RE5-1	GCCATTGCAATGAACACCA
		RE5-2	GAGGGCTATCCTTATGTCGTC
AB5991_18470	*mdxK*	RE6-1	CCAAATACATCGGAACGGC
		RE6-2	GCTGTCATTACGCTTGTTCC
AB5991_18900	*yvyF*	RE7-1	GGATTAACGGATTGTGTTCCA
		RE7-2	GAATGTTTAGACCGGCCTTC
339112	Null	RE8-1	GGATTGAAAAGCACGGAACC
		RE8-2	GTTTCTCGGCCGTCTGAA
2331408	Null	RE9-1	CAAGTATGTCCGCATCCTGC
		RE9-2	GCGTGATCCGCTATGAGG
4120715	Null	RE10-1	GCGACGGCGTGCTAAAAC
		RE10-2	CCGGCACGTCCCTCCTTTTC
