# Supplementary material for: Genomic and Transcriptomic Analysis of Mutant Bacillus subtilis with Enhanced Nattokinase Production via ARTP Mutagenesis
Source: Foods. 2025 Mar 6;14(5):898. doi: 10.3390/foods14050898 (PMC11899143; doi:10.3390/foods14050898)
Supplement: Supplementary file 1 [file foods-14-00898-s001.zip › Table S2.pdf]

Table S2. Statistics of base information before and after filtering.

Sample	Raw Data(bp)	Before Filter				Clean Data(bp)	After Filter			
		Q20	Q30	N	GC		Q20	Q30	N	GC
SD2-1	3425345100	3341878473 (97.56%)	3211274922 (93.75%)	118145 (0.0%)	1563396317 (45.64%)	3086287771	3036888154 (98.4%)	2937291684 (95.17%)	88489 (0.0%)	1384688761 (44.86%)
SD2-2	1814779800	1782837683 (98.24%)	1719339368 (94.74%)	7117 (0.0%)	824894668 (45.46%)	1670256774	1644222080 (98.44%)	1589040041 (95.14%)	6513 (0.0%)	749428898 (44.87%)
SD2-3	3379618500	3276363637 (96.94%)	3141512668 (92.95%)	194217 (0.0%)	1554630817 (46.0%)	2978024732	2936707661 (98.61%)	2852515935 (95.79%)	150684 (0.0%)	1341973836 (45.06%)
JNC002.001-1	2660652600	2583001252 (97.08%)	2456363073 (92.32%)	164699 (0.0%)	1242581675 (46.7%)	2455094393	2391261655 (97.4%)	2280091301 (92.87%)	114995 (0.0%)	1135349352 (46.24%)
JNC002.001-2	2756733600	2674179117 (97.01%)	2547331022 (92.4%)	237325 (0.01%)	1286761589 (46.68%)	2534761809	2474644815 (97.63%)	2368513599 (93.44%)	177652 (0.01%)	1168200585 (46.08%)
JNC002.001-3	2629986000	2546042131 (96.81%)	2413439478 (91.77%)	218261 (0.01%)	1220332720 (46.4%)	2457729164	2387939086 (97.16%)	2269048546 (92.32%)	152543 (0.01%)	1129514288 (45.96%)
