# Supplementary material for: Genomic and Transcriptomic Analysis of Mutant Bacillus subtilis with Enhanced Nattokinase Production via ARTP Mutagenesis
Source: Foods. 2025 Mar 6;14(5):898. doi: 10.3390/foods14050898 (PMC11899143; doi:10.3390/foods14050898)
Supplement: Supplementary file 1 [file foods-14-00898-s001.zip › Table S3.pdf]

Table S3. Transcriptome sequencing results of *B. subtilis* SD2 and JNC002.001.

id	SD2-1_fpkm	SD2-2_fpkm	SD2-3_fpkm	Z4-1_fpkm	Z4-2_fpkm	Z4-3_fpkm	SD2_mean	Z4_mean	log2(fc)	PValue	FDR	Symbol
AB5991_00005	169.16	128.94	173.69	34.74	31.28	21.59	157.26	29.20	-2.4290	1.74E-16	8.80E-16	dnaA
AB5991_00010	60.57	36.49	70.89	41.15	49.19	28.74	55.98	39.69	-0.4961	0.12807111	0.164213877	dnaN
AB5991_00015	8.37	1.54	4.47	9.1	6.81	4.53	4.79	6.81	0.5073	0.606025134	0.662407323	rlbA
AB5991_00020	32.64	41.75	38.21	118.87	100.49	67.33	37.53	95.56	1.3483	4.72E-06	1.01E-05	recF
AB5991_00025	16.16	14.84	20.43	30.37	39.63	22.27	17.14	30.76	0.8433	0.026869745	0.037877088	remB
AB5991_00030	76.66	72.37	86.11	251.85	247.77	170.04	78.38	223.22	1.5099	5.26E-08	1.33E-07	gyrB
AB5991_00035	60.62	56.66	57.06	174.02	177.92	136.87	58.11	162.94	1.4874	1.62E-08	4.31E-08	gyrA
AB5991_00065	104.87	107.83	95.02	1.24	2.33	2.27	102.57	1.95	-5.7195	5.38E-52	3.56E-50	yaaC
AB5991_00070	82.8	74.66	78.93	275.51	207.41	157.52	78.80	213.48	1.4379	5.24E-07	1.23E-06	guaB
AB5991_00075	26.33	18.44	26.27	29.37	25.69	10.87	23.68	21.98	-0.1077	0.758699938	0.807504008	dacA
AB5991_00080	26.35	35.25	26.21	109.06	87.09	59.25	29.27	85.13	1.5403	1.04E-06	2.37E-06	pdxS
AB5991_00085	31.2	29.2	30.74	186.93	152.52	140.04	30.38	159.83	2.3953	1.19E-16	6.12E-16	pdxT
AB5991_00090	13.58	10.13	9.23	64.76	85.36	78.08	10.98	76.07	2.7924	1.18E-19	7.74E-19	serS
AB5991_00100	3.59	11.67	7.98	14.13	17.72	4.49	7.75	12.11	0.6449	0.218274869	0.266125117	dck
AB5991_00105	2.61	3.72	4.34	2.52	2.36	1.25	3.56	2.04	-0.7996	0.132439108	0.169213546	dgk
AB5991_00110	1220.06	2181.84	1358.33	92.63	59.89	66.44	1586.74	72.99	-4.4423	2.78E-38	6.90E-37	sleL
AB5991_00115	29.79	23.71	24.78	22.32	15.16	24.01	26.09	20.50	-0.3483	0.265803504	0.317063897	yaaI
AB5991_00120	6.32	9.56	7.95	2.02	0.38	1.21	7.94	1.20	-2.7227	1.57E-06	3.51E-06	tadA
AB5991_00130	18.16	22.17	21.82	16.27	14.79	12.26	20.72	14.44	-0.5207	0.050937077	0.06884654	dnaX
AB5991_00135	20.08	37.9	24.46	20.63	36.34	24.76	27.48	27.24	-0.0125	1	1	yaaK
AB5991_00140	17.56	20.01	17.81	14.49	18.49	7.54	18.46	13.51	-0.4507	0.22127224	0.269531308	recR
AB5991_00145	7.23	7.38	7.73	7.86	6.54	5.22	7.45	6.54	-0.1873	0.798378425	0.843854332	yaaL
AB5991_00150	2.05	7.54	2.2	0	0.7	2.22	3.93	0.97	-2.0135	0.077875199	0.103046457	bofA

AB5991_00180	25.95	34.04	30.73	21.17	10.38	18.06	30.24	16.54	-0.8708	0.029079541	0.04081826	csfB
AB5991_00185	63.19	97.68	58.77	9.91	9.57	9.23	73.21	9.57	-2.9355	1.50E-18	8.91E-18	xpaC
AB5991_00190	76.13	101.2	67.59	18.79	15.84	16.52	81.64	17.05	-2.2595	2.73E-14	1.16E-13	yaaN
AB5991_00195	31.44	39.56	31.21	11.58	9.43	4.2	34.07	8.40	-2.0195	6.45E-09	1.77E-08	yaaO
AB5991_00200	43	47.26	37.81	7.69	7.77	10.1	42.69	8.52	-2.3250	1.18E-13	4.75E-13	tmk
AB5991_00205	84.9	83.48	82.59	23.83	19.51	15.42	83.66	19.59	-2.0946	8.50E-12	2.99E-11	darA
AB5991_00210	74.19	79.77	66.62	30.76	32.95	21.74	73.53	28.48	-1.3681	2.46E-06	5.41E-06	yaaR
AB5991_00215	102.42	127.06	99.38	29.19	24.71	17.39	109.62	23.76	-2.2057	1.03E-13	4.18E-13	holB
AB5991_00220	117.88	123.86	96.18	23.03	24.66	19.38	112.64	22.36	-2.3329	8.78E-16	4.17E-15	yaaT
AB5991_00225	78.32	106.94	84.83	26.21	26.57	26.09	90.03	26.29	-1.7759	1.04E-09	3.05E-09	yabA
AB5991_00230	11.66	8.92	10.39	4.49	3.46	1.58	10.32	3.18	-1.7003	2.50E-05	5.02E-05	yabB
AB5991_00235	9.04	7.74	5.8	3.28	1.84	2.61	7.53	2.58	-1.5465	0.008627884	0.013022169	yazA
AB5991_00240	32.08	33.23	29.91	19.23	17.79	10.02	31.74	15.68	-1.0174	0.001046949	0.001782013	rsmI
AB5991_00245	18.63	55.89	26.57	75.66	56.26	43.7	33.70	58.54	0.7968	0.047533221	0.064487332	abrB
AB5991_00250	20.66	19.13	22.77	48.28	49.52	39.23	20.85	45.68	1.1312	1.95E-05	3.92E-05	metG
AB5991_00255	10.83	16.85	15.86	15.61	16.53	10.19	14.51	14.11	-0.0407	0.933506403	0.970660887	yabD
AB5991_00260	15.13	15.41	13.24	42.34	46.48	30.08	14.59	39.63	1.4414	4.50E-07	1.06E-06	yabE
AB5991_00265	13.85	13.61	17.23	6.66	5.57	5.23	14.90	5.82	-1.3559	0.000106434	0.000198988	rnmV
AB5991_00270	83.49	115.92	78.29	23.48	18.42	9.35	92.57	17.08	-2.4379	1.24E-11	4.28E-11	rsmA
AB5991_00275	172.67	241.41	158.97	14.19	19.18	23.76	191.02	19.04	-3.3263	2.18E-25	2.20E-24	yabG
AB5991_00280	243.77	498.47	302.9	1873.17	1704.26	1417.61	348.38	1665.01	2.2568	2.66E-12	9.74E-12	veg
AB5991_00285	156.45	173.08	208.88	21.14	23.74	22.09	179.47	22.32	-3.0071	1.78E-20	1.25E-19	sspF
AB5991_00290	9.35	9.92	9.11	32.09	27.7	18.89	9.46	26.23	1.4711	4.89E-06	1.05E-05	ispE
AB5991_00295	15.38	12.38	13.52	49.03	54.89	36.26	13.76	46.73	1.7638	2.93E-09	8.29E-09	purR
AB5991_00300	33.47	47.41	35.8	147.7	161.57	115.95	38.89	141.74	1.8657	1.79E-10	5.58E-10	ridA
AB5991_00305	90.37	160.3	90.07	885.95	672.01	513.11	113.58	690.36	2.6036	7.28E-15	3.25E-14	spoVG

AB5991_00310	26.89	32.68	23.54	153.85	137.13	116.31	27.70	135.76	2.2930	2.47E-16	1.23E-15	glmU
AB5991_00315	42.63	49.4	42.35	175.77	181.64	158.74	44.79	172.05	1.9415	1.35E-13	5.42E-13	prs
AB5991_00320	1011.02	1374.51	769.4	5388.55	4830.49	5414.57	1051.64	5211.20	2.3090	7.72E-16	3.67E-15	ctc
AB5991_00325	40.17	71.41	32.05	293.66	138.21	187.03	47.88	206.30	2.1073	1.59E-08	4.22E-08	spoVC
AB5991_00330	48.51	50.29	51.04	139.56	73.27	91.48	49.95	101.44	1.0221	0.001694044	0.002799438	fin
AB5991_00335	45.37	42.64	43.76	95.18	83.65	99.27	43.92	92.70	1.0776	2.80E-05	5.58E-05	mfd
AB5991_00340	35.34	33.37	32.04	32.58	27.75	37.53	33.58	32.62	-0.0420	0.88751018	0.926472904	spoVT
AB5991_00345	2.37	2.08	1.33	2.7	2.42	1.35	1.93	2.16	0.1627	0.72500782	0.775594303	yabM
AB5991_00350	6.89	7.68	7.1	13.64	17.52	9.85	7.22	13.67	0.9203	0.003834212	0.006058757	yabN
AB5991_00355	15.93	12.72	19.26	21.84	29.6	11.99	15.97	21.14	0.4048	0.368932588	0.426376981	yabO
AB5991_00360	8.35	29.57	18.5	21.41	13.36	20.02	18.81	18.26	-0.0423	1	1	yabP
AB5991_00365	72.75	75.67	64.74	8.96	10.41	12	71.05	10.46	-2.7645	1.27E-20	9.08E-20	yabQ
AB5991_00370	343.81	449.54	329.83	44.72	46.72	28.99	374.39	40.14	-3.2213	5.29E-25	5.10E-24	divIC
AB5991_00375	2132.56	3125.94	2255.59	140.71	147.36	123.36	2504.70	137.14	-4.1909	9.80E-43	3.17E-41	yabR
AB5991_00390	17.97	26.72	10.43	22.87	16.81	16.94	18.37	18.87	0.0387	0.904873896	0.942369326	spoIIE
AB5991_00395	102.87	148.41	77.53	80.71	51.1	47.19	109.60	59.67	-0.8773	0.007744472	0.011760344	yabS
AB5991_00400	23.28	31.66	21.67	13.92	12.84	10.58	25.54	12.45	-1.0368	0.000567432	0.000992194	yabT
AB5991_00405	112.22	213.07	122.73	52.37	55.36	38.75	149.34	48.83	-1.6129	3.16E-07	7.55E-07	tilS
AB5991_00410	8.65	11	8.19	22.08	24.39	11.89	9.28	19.45	1.0678	0.004514833	0.007061206	hprT
AB5991_00415	45.42	57.57	53.12	203.15	159.64	117.15	52.04	159.98	1.6203	2.45E-08	6.41E-08	ftsH
AB5991_00420	73.28	96.96	63.19	7.08	10.18	3.78	77.81	7.01	-3.4718	2.11E-21	1.55E-20	coaX
AB5991_00425	77.38	62.51	65.31	35.91	29.4	23.68	68.40	29.66	-1.2053	2.04E-05	4.11E-05	hslO
AB5991_00430	8.69	8.54	4.54	6.6	7.2	5.91	7.26	6.57	-0.1434	0.722536838	0.773784731	yacD
AB5991_00435	797.68	1318.97	771.92	406.52	256.19	206.85	962.86	289.85	-1.7320	2.20E-07	5.31E-07	cysK
AB5991_00440	125.36	143.75	109.44	24.21	20.44	15.37	126.18	20.01	-2.6570	1.04E-19	6.86E-19	pabB
AB5991_00445	138.42	204.24	147.7	89.05	102.51	68.57	163.45	86.71	-0.9146	0.001422134	0.00237462	pabA

AB5991_00450	33.4	36.88	29.37	17.61	10.64	11.09	33.22	13.11	-1.3409	1.20E-05	2.46E-05	pabC
AB5991_00455	58.99	37.52	40.55	35.97	33.02	31.24	45.69	33.41	-0.4515	0.107454174	0.139581461	sul
AB5991_00460	34.36	17.37	21.83	34.12	27.87	18.33	24.52	26.77	0.1268	0.769105937	0.817212198	folB
AB5991_00465	38.01	28.32	34.52	37.05	32.12	33.77	33.62	34.31	0.0296	0.950848862	0.986369078	folK
AB5991_00470	7.75	6.32	3.68	3.74	3.5	3.73	5.92	3.66	-0.6943	0.33129238	0.387043848	yazB
AB5991_00475	43.83	44.38	38.39	97.51	109.79	82.21	42.20	96.50	1.1933	7.78E-06	1.64E-05	dus1
AB5991_00480	51.45	46.02	41.62	149.66	144.71	103.44	46.36	132.60	1.5161	3.07E-08	7.96E-08	lysS
AB5991_00560	137.6	231.97	132.19	156.42	160.62	205.34	167.25	174.13	0.0581	0.840914753	0.883405419	ctsR
AB5991_00565	114.99	82.68	107.39	153.95	167.8	230.02	101.69	183.92	0.8550	0.002322829	0.003786517	mcsA
AB5991_00570	190.51	183.27	188.51	236.73	253.87	289.91	187.43	260.17	0.4731	0.064360855	0.086139857	mcsB
AB5991_00575	803.82	891.18	720.1	1239.52	1253.08	1372.6	805.03	1288.40	0.6785	0.005315904	0.008239443	clpC
AB5991_00580	442.75	485.66	411.57	306.79	261.04	272.96	446.66	280.26	-0.6724	0.007561423	0.011499965	radA
AB5991_00585	346.14	309.83	316.98	111.63	97.16	92.68	324.32	100.49	-1.6904	1.04E-10	3.32E-10	disA
AB5991_00590	148.24	130.53	125.53	69.28	74.52	76.24	134.77	73.35	-0.8777	0.000524232	0.000919897	yacL
AB5991_00595	95.42	97.34	84.34	69.18	78.95	71.38	92.37	73.17	-0.3361	0.204094153	0.250141322	ispD
AB5991_00600	3.03	5.57	2.84	8.65	9.64	3.69	3.81	7.33	0.9421	0.06458753	0.086414111	ispF
AB5991_00605	190.09	161.37	180.91	184.8	193.84	151.87	177.46	176.84	-0.0050	0.984895889	1	gltX
AB5991_00610	4.42	3.04	5.62	14.73	19.13	12.86	4.36	15.57	1.8367	1.87E-06	4.15E-06	cysE
AB5991_00615	25.54	21.56	20.14	35.36	41.76	40.22	22.41	39.11	0.8033	0.002857003	0.004619364	cysS
AB5991_00620	8.79	4.61	2.24	16.84	18.74	8.61	5.21	14.73	1.4985	0.00386648	0.006104887	mrnC
AB5991_00625	6.51	2.21	4.12	25.69	36.06	22.7	4.28	28.15	2.7175	6.04E-12	2.15E-11	yacO
AB5991_00630	2.47	1.94	3.39	7.66	10.76	6.87	2.60	8.43	1.6970	0.000429045	0.00075789	yacP
AB5991_00635	10.73	13.13	10	75.4	60.76	39.31	11.29	58.49	2.3736	3.14E-12	1.15E-11	sigH
AB5991_00640	2.41	0	2.58	6.55	2.45	0	1.66	3.00	0.8509	0.604915335	0.661570389	rpmGB
AB5991_00645	4.02	3.69	6.44	6.55	4.09	3.26	4.72	4.63	-0.0257	1	1	secE
AB5991_00650	38.59	34.18	44.16	45.65	46.85	17.95	38.98	36.82	-0.0823	0.824709447	0.867758668	nusG

AB5991_00655	538	536.01	568.08	1065.04	967.73	873.13	547.36	968.63	0.8235	0.001209283	0.002045172	rplK
AB5991_00660	572.76	538.91	534.25	822.32	753.99	652.48	548.64	742.93	0.4374	0.082593681	0.108963291	rplA
AB5991_00665	663.47	367	587.98	274.66	393.62	292.91	539.48	320.40	-0.7517	0.012137049	0.017976957	rplJ
AB5991_00670	694.33	385.42	580.4	327.63	487.1	434.98	553.38	416.57	-0.4097	0.165274208	0.205619713	rplL
AB5991_00675	9.25	5.48	11.16	34.71	56.16	38.1	8.63	42.99	2.3166	1.67E-10	5.24E-10	ybxB
AB5991_00680	680.31	584.93	680.04	1023.74	1105.29	1033.69	648.43	1054.24	0.7012	0.003720986	0.005891561	rpoB
AB5991_00685	1426.21	991.25	1315.36	2258.28	2801.54	2755.84	1244.27	2605.22	1.0661	2.96E-05	5.88E-05	rpoC
AB5991_00690	202.53	98.63	164.57	176.84	234.2	175.23	155.24	195.42	0.3321	0.295280619	0.34856104	rplGB
AB5991_00695	472.03	251.5	460.29	668	915.82	711.33	394.61	765.05	0.9551	0.001901232	0.003122329	rpsL
AB5991_00700	337.71	219.85	304.51	627.72	822.93	719.92	287.36	723.52	1.3322	2.14E-06	4.72E-06	rpsG
AB5991_00705	911.83	590.19	845.32	1349.31	1636.96	1436.36	782.45	1474.21	0.9139	0.000525291	0.000921348	fusA
AB5991_00710	1103.31	933.53	963.06	4204.45	4949.75	4637.55	999.97	4597.25	2.2008	4.90E-18	2.83E-17	tuf
AB5991_00715	23.99	18.03	19.39	8.63	9.61	7.97	20.47	8.74	-1.2284	4.82E-05	9.33E-05	ybaC
AB5991_00720	88.33	51.56	65.68	476.5	597.12	474.91	68.52	516.18	2.9132	7.98E-22	6.11E-21	rpsJ
AB5991_00725	265.1	217.57	218.45	1225.04	1431.38	1348.82	233.71	1335.08	2.5142	4.79E-21	3.50E-20	rplC
AB5991_00730	235.21	168.07	201.04	1279.04	1526.8	1498.5	201.44	1434.78	2.8324	2.93E-24	2.67E-23	rplD
AB5991_00735	67.15	58.77	67.79	379.51	470.12	414.42	64.57	421.35	2.7061	6.46E-22	5.01E-21	rplW
AB5991_00740	448.19	304.43	362.25	1924.8	2274.78	2174.8	371.62	2124.79	2.5154	2.58E-20	1.79E-19	rplB
AB5991_00745	105.6	63.05	96.99	678.52	752.32	694.28	88.55	708.37	3.0000	3.87E-24	3.49E-23	rpsS
AB5991_00750	211.4	183.41	189.34	1277.78	1562.04	1431.99	194.72	1423.94	2.8704	1.38E-25	1.42E-24	rplV
AB5991_00755	275.11	186.4	221.24	1373.37	1653.67	1630.51	227.58	1552.52	2.7701	1.51E-22	1.22E-21	rpsC
AB5991_00760	179.09	122.07	174.63	946.3	1181.99	1142.03	158.60	1090.11	2.7810	8.46E-22	6.46E-21	rplP
AB5991_00765	28.78	3.3	23.08	132.03	142.77	145.04	18.39	139.95	2.9281	1.03E-11	3.58E-11	rpmC
AB5991_00770	80.79	66.63	65.9	439.33	623.65	581.8	71.11	548.26	2.9468	1.04E-23	9.08E-23	rpsQ
AB5991_00775	165.56	113.33	144.58	905.12	1149.62	1103.45	141.16	1052.73	2.8988	2.62E-23	2.21E-22	rplN
AB5991_00780	177.27	125.52	191.44	834.21	1089.6	1005.27	164.74	976.36	2.5672	8.42E-19	5.11E-18	rplX

AB5991_00785	292.21	183.15	238.75	1141.99	1416.14	1408.76	238.04	1322.30	2.4738	1.01E-17	5.65E-17	rplE
AB5991_00790	95.23	74.94	83.14	414.3	518.25	491.26	84.44	474.60	2.4908	1.56E-17	8.55E-17	rpsZ
AB5991_00795	265.46	158.87	181.18	823.27	978.35	999.39	201.84	933.67	2.2097	6.22E-14	2.57E-13	rpsH
AB5991_00800	459.57	310.38	405.2	1211.88	1409.67	1368.54	391.72	1330.03	1.7636	8.25E-11	2.65E-10	rplF
AB5991_00805	378.93	250.52	301.39	1006.73	1179.77	1087.73	310.28	1091.41	1.8146	6.95E-11	2.25E-10	rplR
AB5991_00810	422.11	272.93	335.66	1193.22	1404.11	1254.42	343.57	1283.92	1.9019	7.08E-12	2.50E-11	rpsE
AB5991_00815	49.2	23.97	36.51	97.2	132.86	106.53	36.56	112.20	1.6177	2.27E-06	4.99E-06	rpmD
AB5991_00820	495.52	307.05	418.58	1276.66	1531.74	1540.44	407.05	1449.61	1.8324	8.49E-11	2.72E-10	rplO
AB5991_00825	580.32	386.95	490.1	1173.72	1518.37	1451.91	485.79	1381.33	1.5077	2.26E-08	5.93E-08	secY
AB5991_00830	410.69	219.74	323.34	628.52	866.63	773.97	317.92	756.37	1.2504	2.66E-05	5.31E-05	adk
AB5991_00835	417.39	237.25	366.4	691.59	940.23	888.46	340.35	840.09	1.3035	8.18E-06	1.71E-05	map
AB5991_00845	70.98	19.7	61.78	134.64	176.4	165.28	50.82	158.77	1.6435	1.96E-05	3.95E-05	infA
AB5991_00850	17.44	8.73	25.43	41.39	72.62	44.62	17.20	52.88	1.6202	0.000557856	0.00097631	rpmJ
AB5991_00855	217.29	156.88	189.07	590.82	811.73	787.99	187.75	730.18	1.9595	2.27E-12	8.38E-12	rpsM
AB5991_00860	439.55	290.82	350.96	1179.98	1601.28	1548.49	360.44	1443.25	2.0015	1.88E-12	7.00E-12	rpsK
AB5991_00865	779.61	466.75	649.02	1897.16	2558.49	2452.29	631.79	2302.65	1.8658	4.96E-11	1.63E-10	rpoA
AB5991_00870	387.39	245.03	326.95	1061.43	1338.39	1258.59	319.79	1219.47	1.9311	1.12E-11	3.89E-11	rplQ
AB5991_00875	26.49	20.79	23.99	23	32.18	31.22	23.76	28.80	0.2777	0.33459879	0.3906768	ecfA
AB5991_00880	43.63	37.77	44.88	27.11	42.29	37.56	42.09	35.65	-0.2396	0.390897848	0.448240068	ecfAB
AB5991_00885	28.99	22.46	31.49	18.72	25.13	18.63	27.65	20.83	-0.4087	0.161087246	0.200777606	ecfT
AB5991_00890	69.97	55.32	78.46	30.12	26.95	29.19	67.92	28.75	-1.2400	5.06E-06	1.08E-05	truA
AB5991_00895	263.28	255.36	337.6	1095.11	1092.83	867.07	285.41	1018.34	1.8351	2.33E-11	7.88E-11	rplM
AB5991_00900	332.52	310.78	339.37	1575.15	1490.39	1290.97	327.56	1452.17	2.1484	2.91E-16	1.44E-15	rpsI
AB5991_00905	1.65	7.35	6.8	27.39	23.71	20.13	5.27	23.74	2.1726	2.72E-07	6.55E-07	ybaJ
AB5991_00910	0.81	0	2.61	2.66	2.07	4.41	1.14	3.05	1.4182	0.097285188	0.127288133	ybaK
AB5991_00915	15.19	31.61	18.14	6.06	2.58	1.37	21.65	3.34	-2.6977	3.97E-08	1.02E-07	cwlD

AB5991_00920	170.68	191.17	140.73	971.21	1051.47	1073.09	167.53	1031.92	2.6229	7.51E-22	5.78E-21	salA
AB5991_00925	11.34	8.92	14.55	1.06	0	0.7	11.60	0.59	-4.3059	2.54E-13	1.01E-12	gerD
AB5991_00930	6.06	7.23	6.48	27.66	36.67	32.12	6.59	32.15	2.2865	3.71E-12	1.34E-11	kbaA
AB5991_00935	19.61	37.74	24.26	4.37	2.16	0.51	27.20	2.35	-3.5351	4.92E-13	1.92E-12	pdaB
AB5991_01020	213.58	190.77	231.9	5.47	9.22	5.17	212.08	6.62	-5.0017	4.89E-49	2.66E-47	ybaR
AB5991_01025	50.04	68.83	52.05	7.26	7.79	8.5	56.97	7.85	-2.8595	1.70E-21	1.26E-20	ybaS
AB5991_01030	12.24	14.54	8.21	16.19	13.44	13.51	11.66	14.38	0.3021	0.355096943	0.411945651	ybbA
AB5991_01035	5.36	4.6	3.63	11.86	13.1	19.55	4.53	14.84	1.7116	1.70E-06	3.79E-06	feuC
AB5991_01040	19.78	14.2	20.96	34.82	43.75	43.61	18.31	40.73	1.1531	3.81E-05	7.48E-05	feuB
AB5991_01045	91.7	64.01	74.36	129.82	174.12	181.3	76.69	161.75	1.0766	9.12E-05	0.000171786	feuA
AB5991_01050	45.7	29.64	48.75	23.37	35.63	33.72	41.36	30.91	-0.4204	0.163155804	0.203164534	btr
AB5991_01055	227.35	301.77	224.34	386.06	491.89	509.19	251.15	462.38	0.8805	0.001013156	0.001726714	ybbC
AB5991_01060	139.43	136.09	130.17	282.59	369.06	406.13	135.23	352.59	1.3826	1.81E-07	4.39E-07	nagZ
AB5991_01065	157.99	175.46	163.99	190.21	228.91	261.47	165.81	226.86	0.4523	0.085206428	0.112089139	amiE
AB5991_01070	149.57	147.99	150.2	116.97	128.69	138.02	149.25	127.89	-0.2228	0.374140779	0.431893323	ybbF
AB5991_01075	150.42	150.41	139.67	61.71	59.85	61.42	146.83	60.99	-1.2675	5.19E-07	1.22E-06	ybbH
AB5991_01080	173.64	170.84	166.68	97.32	79.61	84.68	170.39	87.20	-0.9664	0.000139457	0.000257454	murQ
AB5991_01085	16.47	12.37	22.01	3.66	1.9	1.62	16.95	2.39	-2.8242	1.16E-09	3.38E-09	ybbJ
AB5991_01090	22.2	32.02	26.28	3.45	1.61	1.29	26.83	2.12	-3.6642	4.19E-16	2.04E-15	ybbK
AB5991_01120	3740.29	3579	3441.59	2012.86	1790.66	1756.1	3586.96	1853.21	-0.9527	8.68E-05	0.000164277	sigW
AB5991_01125	4535.16	4599.88	4136.55	2661.52	2339.24	2239.96	4423.86	2413.57	-0.8741	0.000344417	0.00061386	rsiW
AB5991_01130	15.17	31.49	17.17	33.48	35.36	15.71	21.28	28.18	0.4056	0.282456702	0.334916562	cdaA
AB5991_01135	37.14	72.99	33.08	55.53	51.46	34.69	47.74	47.23	-0.0155	0.974698083	1	cdaR
AB5991_01140	7.51	13.31	11.05	123.76	109.8	87.44	10.62	107.00	3.3323	1.55E-25	1.58E-24	glmM
AB5991_01145	422.05	373.12	416.18	923.27	935.4	873.37	403.78	910.68	1.1734	1.64E-06	3.68E-06	glmS
AB5991_01150	0	0	0	0	0	0	0.00	0.00	0.0000	1	1	--

AB5991_01160	102.88	72.54	91.48	1.24	1.45	2.46	88.97	1.72	-5.6956	1.40E-46	6.03E-45	adaA
AB5991_01165	79.66	74.98	71.95	0.73	1.7	2.54	75.53	1.66	-5.5107	6.48E-42	1.96E-40	adaB
AB5991_01170	2.02	1.53	1.78	0	0.24	0	1.78	0.08	-4.4730	1.24E-08	3.32E-08	dabB
AB5991_01175	12.3	9.77	9.61	0.45	0.49	1.27	10.56	0.74	-3.8415	1.22E-21	9.17E-21	dabA
AB5991_01180	16.77	8.8	12.45	0	0.35	0.37	12.67	0.24	-5.7226	6.93E-16	3.33E-15	ybcF
AB5991_01185	4.35	12.55	7.97	1.35	0	0.67	8.29	0.67	-3.6220	4.08E-05	7.99E-05	ybcH
AB5991_01190	15.91	11.51	10.31	1.57	0.49	1.57	12.58	1.21	-3.3777	2.34E-09	6.64E-09	ybcI
AB5991_01200	44.87	46.07	39.56	0.6	0.19	0.79	43.50	0.53	-6.3680	1.38E-47	6.38E-46	ybdK
AB5991_01210	25.32	14.64	28.83	4.08	4.06	2.79	22.93	3.64	-2.6539	1.05E-11	3.65E-11	ybdM
AB5991_01215	97.12	111.4	92.37	553.77	674.73	601.12	100.30	609.87	2.6042	1.97E-21	1.45E-20	ybdN
AB5991_01220	16.02	15.4	15.17	2.32	3.42	1.98	15.53	2.57	-2.5933	2.45E-14	1.05E-13	ybdO
AB5991_01225	2.34	1.67	1.11	11.75	10.33	7.47	1.71	9.85	2.5289	1.62E-10	5.09E-10	ybxG
AB5991_01230	0	0	0	0	0.74	0	0.00	0.25	7.9464	1	1	csgA
AB5991_01235	1.88	0	1.01	0	0	0	0.96	0.00	-9.9119	0.264561319	0.316391271	ybxH
AB5991_01240	36.64	50.77	40.63	5.62	7.09	6.81	42.68	6.51	-2.7136	1.36E-17	7.51E-17	ybxI
AB5991_01245	3.6	4.23	3.08	30.57	29.19	27.46	3.64	29.07	2.9990	1.97E-21	1.45E-20	cypC
AB5991_01255	26.89	24.17	25.18	15.78	15.22	13.89	25.41	14.96	-0.7642	0.002921963	0.004712882	ybeC
AB5991_01260	13.12	11.67	9.64	45.69	62.15	59.01	11.48	55.62	2.2768	8.57E-15	3.80E-14	glpQ
AB5991_01265	3.79	1.74	5.07	6.04	6.75	4.69	3.53	5.83	0.7216	0.084350748	0.111059953	glpT
AB5991_01270	5.81	1.33	3.11	0	0.74	1.57	3.42	0.77	-2.1497	0.044539752	0.060695729	ybeF
AB5991_01275	18.31	13.02	14.53	8.78	11.02	5.12	15.29	8.31	-0.8799	0.011010408	0.016344796	ybfA
AB5991_01285	0	0.36	0.21	0.22	0.81	1.29	0.19	0.77	2.0251	0.137939203	0.175226032	ybfF
AB5991_01290	25.97	22.79	23.56	3.4	3.51	5.16	24.11	4.02	-2.5830	1.56E-18	9.24E-18	ybfG
AB5991_01295	2.55	2.16	4.2	3.63	2.8	1.7	2.97	2.71	-0.1322	0.80600246	0.850409745	ybfH
AB5991_01300	1.31	3.21	3.27	9.5	7.55	4.25	2.60	7.10	1.4512	0.00247731	0.004033374	ybfI
AB5991_01305	0	0	0	0	0	0	0.00	0.00	0.0000	1	1	--

AB5991_01310	1.49	9.56	7.16	0	0	0	6.07	0.00	-12.5675	0.000104009	0.000194545	xhlB
AB5991_01315	73.12	62.17	67.79	3.53	3.08	2.81	67.69	3.14	-4.4302	2.56E-42	8.07E-41	sle1
AB5991_01320	119.35	147.65	121.27	171.67	174.17	133.42	129.42	159.75	0.3037	0.244978427	0.295327667	ydeJ
AB5991_01325	8.5	7.38	10.08	14.79	9.12	6.86	8.65	10.26	0.2452	0.500560273	0.560712227	purT
AB5991_01330	123.57	176.51	118.6	200.55	168.73	160.77	139.56	176.68	0.3403	0.210036974	0.25686998	mpr
AB5991_01335	80.63	143.9	93.01	100.51	93.59	70.12	105.85	88.07	-0.2652	0.401020397	0.458391478	ybfJ
AB5991_01340	102.04	130.37	124.52	2.87	2.06	3.73	118.98	2.89	-5.3651	1.66E-47	7.59E-46	ybfK
AB5991_01345	400.42	446.87	464.05	27.24	23.08	23.45	437.11	24.59	-4.1519	1.52E-44	5.59E-43	pssA
AB5991_01350	479.78	583.01	511.5	27.74	30.1	29.61	524.76	29.15	-4.1701	6.85E-45	2.59E-43	ybfM
AB5991_01355	606.83	740.05	680.68	37.73	33.91	36.56	675.85	36.07	-4.2280	2.17E-46	9.17E-45	psd
AB5991_01360	23.97	24.98	28.41	2.11	1.98	0	25.79	1.36	-4.2414	6.66E-15	2.99E-14	ybfN
AB5991_01365	59.85	55.93	48.72	50.43	42.39	39.25	54.83	44.02	-0.3168	0.227228229	0.275434462	ybfO
AB5991_01370	10.58	10.09	8.27	15.94	16.37	22.92	9.65	18.41	0.9324	0.002893197	0.004670279	ybfP
AB5991_01375	32.46	65.42	26.13	56.4	61.32	52.9	41.34	56.87	0.4603	0.180443998	0.22350066	trhO
AB5991_01380	27.73	28.79	28.26	25.11	20.24	23.26	28.26	22.87	-0.3053	0.238938734	0.288660089	gltP
AB5991_01385	3.53	4.03	4.08	1.45	1.16	1.34	3.88	1.32	-1.5592	1.86E-05	3.76E-05	gamP
AB5991_01390	12.77	12.39	9.02	6.81	8.83	8.35	11.39	8.00	-0.5107	0.137328487	0.17461781	gamA
AB5991_01395	9.19	17.81	12.83	5.83	7.79	3.87	13.28	5.83	-1.1873	0.003501633	0.005561995	gamR
AB5991_01400	14.41	16.84	13.31	2.14	2.67	2.84	14.85	2.55	-2.5422	8.76E-07	2.01E-06	ybgB
AB5991_01405	46.75	63.84	49.09	51.58	44.32	43.85	53.23	46.58	-0.1923	0.492830241	0.552676896	ilvE
AB5991_01410	19.44	17.62	19.01	9.18	3.91	3.46	18.69	5.52	-1.7604	3.01E-06	6.55E-06	ybgF
AB5991_01415	16.59	21.71	16.11	4.15	1.94	3.92	18.14	3.34	-2.4424	3.05E-11	1.02E-10	ybgG
AB5991_01425	2.39	1.35	1.96	0	0	0	1.90	0.00	-10.8918	3.50E-07	8.32E-07	glsA1
AB5991_01430	3.96	4.85	4.39	4.78	4.48	2.06	4.40	3.77	-0.2217	0.610032974	0.666054699	glnK
AB5991_01435	15.88	17.21	13.3	9.15	9.93	3.11	15.46	7.40	-1.0639	0.0071307	0.010890773	glnL
AB5991_01440	7.02	3.58	5.63	2.76	1.59	2.74	5.41	2.36	-1.1948	0.005592244	0.008644143	ycbC

AB5991_01445	9.12	13.8	12.78	13.94	10.78	13.07	11.90	12.60	0.0821	0.775148709	0.822626461	gucD
AB5991_01450	11.23	9.7	9.89	10.63	14.39	13.02	10.27	12.68	0.3037	0.306482793	0.360819203	gudP
AB5991_01455	12.55	12.62	8.9	15.81	22.05	23.03	11.36	20.30	0.8377	0.004893457	0.007623349	gudD
AB5991_01460	81.88	111.58	82.88	45.08	48.74	34	92.11	42.61	-1.1123	0.000138484	0.000255777	ycbG
AB5991_01465	11.91	19.27	10.84	36.67	31.68	35.23	14.01	34.53	1.3016	9.45E-06	1.96E-05	garD
AB5991_01470	158.38	171.17	154.47	161.58	165.58	111.11	161.34	146.09	-0.1432	0.593937924	0.65044884	ycbJ
AB5991_01475	2.23	0	0	2.43	4.54	7.25	0.74	4.74	2.6728	0.039708085	0.054579719	rtpA
AB5991_01480	29.45	39.23	30.67	95.67	71.9	67.51	33.12	78.36	1.2426	1.63E-05	3.32E-05	ycbK
AB5991_01485	0	0.97	0.28	0.58	2.97	0.86	0.42	1.47	1.8189	0.072451231	0.09631866	ycbL
AB5991_01490	2.32	2.13	2.27	1.68	1.97	1.05	2.24	1.57	-0.5158	0.347963128	0.40449695	ycbM
AB5991_01495	3.91	3.95	1.88	3.62	8.36	1.48	3.25	4.49	0.4667	0.439139386	0.497098775	ycbN
AB5991_01500	2.63	1.93	1.69	2.86	3.21	1.14	2.08	2.40	0.2061	0.81373404	0.857118799	ycbO
AB5991_01505	0	0	0	2.91	2.73	0	0.00	1.88	10.8765	0.139958323	0.177280543	--
AB5991_01510	36.43	84.04	36.46	718.76	696.38	550.58	52.31	655.24	3.6469	3.72E-23	3.12E-22	ycbP
AB5991_01515	96.06	187.99	127.96	8.25	11.58	18.7	137.34	12.84	-3.4186	4.44E-19	2.75E-18	cwlJ
AB5991_01520	10.12	26.75	10.3	0.54	1.76	1.07	15.72	1.12	-3.8070	8.60E-11	2.76E-10	ycbR
AB5991_01525	0	0	0	0	0	0	0.00	0.00	0.0000	1	1	yczK
AB5991_01530	20.32	10.61	18.87	12.23	15.54	11.73	16.60	13.17	-0.3343	0.277709967	0.329484996	phoD
AB5991_01535	17.82	6.23	19.06	10.15	9.5	18.37	14.37	12.67	-0.1813	0.726205558	0.776457262	tatAd
AB5991_01540	0.74	0.46	0.8	4.31	2.27	1.61	0.67	2.73	2.0339	0.003992912	0.006292005	tatC1
AB5991_01545	31.8	51.73	37.29	94.95	103.05	87.87	40.27	95.29	1.2425	1.43E-05	2.90E-05	pcp
AB5991_01550	1.62	4.17	2.08	15.72	10.58	8.09	2.62	11.46	2.1276	5.31E-07	1.25E-06	ycbU
AB5991_01555	7.78	4.38	8.32	9.69	5.37	3.94	6.83	6.33	-0.1082	0.78908585	0.835532966	lmrB
AB5991_01560	17.21	15.22	16.02	13.87	15.57	12.42	16.15	13.95	-0.2109	0.510234435	0.568501948	lmrA
AB5991_01565	13.46	14.42	12.51	4.88	1.47	2.08	13.46	2.81	-2.2604	2.33E-08	6.10E-08	ansZ
AB5991_01570	2.55	1.56	2.42	7.69	11.52	1.84	2.18	7.02	1.6887	0.00794194	0.0120326	estA

AB5991_01575	0.94	3.46	2.52	13.31	6.23	2.55	2.31	7.36	1.6745	0.021228974	0.030488338	yczC
AB5991_01580	24	33.9	30.15	11.68	12.64	2.91	29.35	9.08	-1.6931	7.25E-05	0.000137726	yccF
AB5991_01590	1.29	2.36	1.65	4.48	3.14	1.67	1.77	3.10	0.8097	0.185461869	0.2292154	natR
AB5991_01600	1.87	5.43	3.5	0.85	1.43	1.85	3.60	1.38	-1.3868	0.013538362	0.019970592	natB
AB5991_01605	25.77	29.53	26.93	28.87	26.03	30.2	27.41	28.37	0.0495	0.858936033	0.899956461	yccK
AB5991_01610	1.42	2.6	0.95	0	0	0	1.66	0.00	-10.694	7.36E-06	1.55E-05	ycdA
AB5991_01615	0	0	0	0	0	0	0.00	0.00	0.0000	1	1	--
AB5991_01620	33.18	31.51	28.95	17.83	19.53	15.69	31.21	17.68	-0.8198	0.001814138	0.002989187	ycdB
AB5991_01625	46.32	45.08	38.18	25.11	32.01	20.54	43.19	25.89	-0.7386	0.009929638	0.014845855	ycdC
AB5991_01630	15.06	18.44	18.41	3.12	1.82	2.33	17.30	2.42	-2.8360	1.40E-11	4.79E-11	cwlK
AB5991_01640	0.47	1.28	2.49	49.59	59.19	58.42	1.41	55.73	5.3014	1.78E-37	4.20E-36	ycdF
AB5991_01645	7.61	8.86	7.11	73.92	76.7	92.26	7.86	80.96	3.3646	5.31E-30	7.40E-29	ycdG
AB5991_01650	86.42	112.7	96.24	4.5	5.75	5.71	98.45	5.32	-4.2099	7.09E-37	1.60E-35	znuA
AB5991_01655	1.04	5.72	2.5	1.98	2.11	1.41	3.09	1.83	-0.7516	0.318432846	0.373448562	znuC
AB5991_01660	14.37	22.05	13.76	1.4	1.09	0.7	16.73	1.06	-3.9755	5.12E-18	2.93E-17	znuB
AB5991_01670	71.09	162.07	76.03	60.61	49.67	35.22	103.06	48.50	-1.0875	0.002938329	0.004731591	yceC
AB5991_01675	62.73	84.4	58.45	174.63	164.68	122.71	68.53	154.01	1.1683	3.90E-05	7.65E-05	yceD
AB5991_01680	83.04	91.71	73.44	275.69	254.49	252.43	82.73	260.87	1.6568	1.96E-10	6.10E-10	yceE
AB5991_01685	125.64	134.64	121.12	275.06	316.58	295.01	127.13	295.55	1.2171	1.61E-06	3.61E-06	yceF
AB5991_01690	7.28	6.79	6.59	30.81	26.1	19.03	6.89	25.31	1.8780	3.95E-10	1.19E-09	yceG
AB5991_01695	23.67	20.67	19.65	36.54	37.9	35.84	21.33	36.76	0.7853	0.002633985	0.004269206	yceH
AB5991_01700	0.45	0.62	0.61	1.39	0.97	0.74	0.56	1.03	0.8838	0.222489982	0.270825006	insK
AB5991_01705	17.58	12.41	19.76	5.39	5.35	3.58	16.58	4.77	-1.7967	6.92E-08	1.74E-07	naiP
AB5991_01710	25.12	16.69	30.16	1.17	2.04	0.67	23.99	1.29	-4.2133	2.07E-22	1.67E-21	yceJ
AB5991_01715	113.34	111.73	96.97	23.36	26.11	12.27	107.35	20.58	-2.3830	6.55E-13	2.53E-12	yceK
AB5991_01720	3.16	4.49	4.46	43.95	36.29	23.5	4.04	34.58	3.0987	4.28E-18	2.47E-17	opuAA

AB5991_01725	18.73	25.8	26.41	155.37	107.47	89.19	23.65	117.34	2.3110	4.98E-13	1.94E-12	opuAB
AB5991_01730	36.89	34.24	41.42	234.69	226.3	183.02	37.52	214.67	2.5165	6.53E-20	4.38E-19	opuAC
AB5991_01735	1.73	3.17	1.51	15.7	5.75	4.25	2.14	8.57	2.0034	0.000327737	0.000584919	amhX
AB5991_01740	4.54	6.94	3.64	10.69	10.39	9.82	5.04	10.30	1.0311	0.001461711	0.002437822	ycgA
AB5991_01755	17.59	31.61	21.61	22.38	28.95	29.77	23.60	27.03	0.1957	0.513772507	0.569904577	ldh
AB5991_01770	10.2	10.89	9.13	3.35	4.35	5.39	10.07	4.36	-1.2070	0.000843409	0.001445481	ycgG
AB5991_01785	33.1	61.19	24.07	523.74	530.31	649.1	39.45	567.72	3.8470	3.26E-25	3.20E-24	nadE
AB5991_01790	2.13	4.47	3.9	6.95	8.36	12.85	3.50	9.39	1.4233	0.001500604	0.002495351	tmrB
AB5991_01795	694.97	1051.87	722.18	5.61	5.9	9.42	823.01	6.98	-6.8822	2.21E-68	5.49E-66	aroK
AB5991_01805	20.64	25.26	19.79	23.73	29.45	26.96	21.90	26.71	0.2868	0.311690842	0.36640744	ycgJ
AB5991_01810	2.41	3.06	3.37	5.65	3.77	2.21	2.95	3.88	0.3957	0.41588224	0.473471438	ycgK
AB5991_01815	8.12	11.44	6.46	60.6	32.29	14.31	8.67	35.73	2.0426	1.03E-05	2.13E-05	cah
AB5991_01820	11.54	11.44	10.86	26.86	18.09	6	11.28	16.98	0.5904	0.187669391	0.231788732	ycgL
AB5991_01825	16.65	18.2	12.93	17.89	9.48	6.22	15.93	11.20	-0.5084	0.199151402	0.244839077	putB
AB5991_01830	91.31	80.4	50.57	75.43	61.32	45.12	74.09	60.62	-0.2895	0.34526566	0.401714016	putC
AB5991_01835	14.49	13.77	9.24	20.6	17.21	12.8	12.50	16.87	0.4325	0.166853077	0.207443196	putP
AB5991_01840	11.26	20.41	12.04	9.22	9.82	4.59	14.57	7.88	-0.8873	0.020053355	0.028914987	putR
AB5991_01845	98.8	94.38	98.22	8.48	9.86	6.84	97.13	8.39	-3.5327	2.66E-30	3.73E-29	ycgQ
AB5991_01850	51.67	58.88	52.64	3.55	2.91	3.32	54.40	3.26	-4.0606	1.83E-34	3.46E-33	ycgR
AB5991_01855	308.39	272.89	302.35	8.74	12.95	20.53	294.54	14.07	-4.3874	2.39E-36	5.21E-35	ycgS
AB5991_01860	6.08	5.91	7.07	25.28	24.38	25.35	6.35	25.00	1.9765	5.83E-11	1.90E-10	ycgT
AB5991_01865	26.02	26.74	26.89	63.9	60.05	61.45	26.55	61.80	1.2189	1.93E-06	4.28E-06	nasF
AB5991_01870	18.02	27.92	10.24	28.78	20.63	21.33	18.73	23.58	0.3325	0.383783763	0.440973762	nasE
AB5991_01875	23.4	29.37	23.42	22.44	12.93	10.11	25.40	15.16	-0.7444	0.024046576	0.034249983	nasD
AB5991_01880	5.85	6.85	6.25	1.84	0.78	0.73	6.32	1.12	-2.5000	2.33E-10	7.21E-10	nasC
AB5991_01885	5.7	5.88	3.92	1.19	0.4	0.25	5.17	0.61	-3.0745	9.22E-11	2.95E-10	nasB

AB5991_01890	5.55	4.13	5.93	1.96	2.29	0	5.20	1.42	-1.8769	0.001103499	0.001876657	nasA
AB5991_01900	5.25	6.81	5.29	1.01	2.83	0.33	5.78	1.39	-2.0568	0.000266847	0.000479047	yciB
AB5991_01905	0	0	1.02	0	0	0	0.34	0.00	-8.4094	1	1	yczL
AB5991_01915	23.89	31.19	22.14	7.22	3.24	4.88	25.74	5.11	-2.3317	1.74E-10	5.45E-10	yckA
AB5991_01920	109.41	137.52	106.49	8.42	7.88	6.11	117.81	7.47	-3.9792	7.20E-34	1.27E-32	yckB
AB5991_01925	3.65	1.68	1.95	0	0.93	0.99	2.43	0.64	-1.9228	0.208950532	0.255698787	--
AB5991_01930	5.95	13.1	11.45	12.5	4.84	6.87	10.17	8.07	-0.3332	0.530695772	0.587508478	yckC
AB5991_01935	80.33	89.7	83.01	2.95	3.31	2.94	84.35	3.07	-4.7816	9.94E-33	1.60E-31	yckD
AB5991_01940	90.59	98.86	104.19	40.43	28.27	26.37	97.88	31.69	-1.6270	1.04E-08	2.81E-08	--
AB5991_01945	37.16	49.63	37.94	25.72	18.34	10.36	41.58	18.14	-1.1966	0.001106244	0.00188052	--
AB5991_01955	110.67	120.58	124.68	179.99	162.02	123.48	118.64	155.16	0.3872	0.143599718	0.18119939	bglC
AB5991_01960	17.67	18.3	21.8	8.87	11.07	12.75	19.26	10.90	-0.8215	0.018312468	0.026597955	nin
AB5991_01965	0.8	1.48	1.29	1.75	2.04	0.43	1.19	1.41	0.2413	1	1	nucA
AB5991_01975	20.08	23.79	17.32	4.23	2.97	6.31	20.40	4.50	-2.1793	1.17E-08	3.14E-08	hxlB
AB5991_01980	13.42	22.02	21.68	1.24	2.32	3.4	19.04	2.32	-3.0368	4.70E-12	1.69E-11	hxlA
AB5991_01985	30.37	24.69	30.88	15.7	19.76	13.48	28.65	16.31	-0.8123	0.012181009	0.018035341	hxlR
AB5991_01990	84.18	87.23	54.77	346.26	370.5	373.67	75.39	363.48	2.2694	1.70E-16	8.61E-16	srfAA
AB5991_02000	3.85	4.71	8.23	8.37	10.44	13.88	5.60	10.90	0.9612	0.185827277	0.229595556	comS
AB5991_02010	140.28	129.1	107.05	437.58	440.39	485.78	125.48	454.58	1.8571	4.74E-13	1.85E-12	srfAC
AB5991_02015	141.82	130.66	98.9	488.62	491.31	523.11	123.79	501.01	2.0169	1.93E-13	7.71E-13	srfAD
AB5991_02020	4.86	2.98	2.68	6.25	2.55	3.35	3.51	4.05	0.2078	0.695772132	0.749162456	ycxA
AB5991_02025	13.6	15.46	19.05	6.69	5.6	4.56	16.04	5.62	-1.5136	4.09E-05	8.00E-05	ycxB
AB5991_02030	33.88	30.75	31.29	12.35	16.85	10.42	31.97	13.21	-1.2756	1.39E-05	2.82E-05	ycxC
AB5991_02035	0.27	2.73	1.3	4.12	2.2	1.61	1.43	2.64	0.8830	0.163071998	0.203123872	ycxD
AB5991_02040	13.92	8.85	15.75	8.45	7.09	7.25	12.84	7.60	-0.7572	0.027730588	0.039035153	sfp
AB5991_02045	7.25	4.61	8.05	9.4	5.11	5.74	6.64	6.75	0.0244	1	1	yczE

AB5991_02050	21.86	19.63	20.26	14	17.31	20.25	20.58	17.19	-0.2602	0.381966287	0.43939401	tcyC
AB5991_02055	17.43	18.36	26.32	12.55	14.87	15.54	20.70	14.32	-0.5318	0.087099015	0.114412897	tcyB
AB5991_02060	48.6	55.11	48.86	36.54	41.94	56.49	50.86	44.99	-0.1768	0.532391735	0.589221734	tcyA
AB5991_02065	1.86	0.38	0.66	4.5	3.37	2.91	0.97	3.59	1.8942	0.00114169	0.001937457	bsdA
AB5991_02070	9.11	18.35	8.8	96.53	111.87	132.99	12.09	113.80	3.2350	1.38E-20	9.81E-20	bsdB
AB5991_02075	16.52	17.74	11.83	138.1	186.68	187.54	15.36	170.77	3.4745	9.57E-30	1.31E-28	bsdC
AB5991_02080	4.76	10.19	8.48	57.77	75.84	78.95	7.81	70.85	3.1814	7.75E-16	3.69E-15	bsdD
AB5991_02090	5.55	5.1	4.8	40.2	38.27	44.41	5.15	40.96	2.9916	3.30E-21	2.42E-20	yclE
AB5991_02095	84.45	64.63	90.31	59.28	55.6	42.47	79.80	52.45	-0.6054	0.025936716	0.036665966	yclF
AB5991_02100	1465.77	1905.86	1353.39	24.42	35.74	50.06	1575.01	36.74	-5.4219	1.03E-51	6.69E-50	yclG
AB5991_02105	8.14	2.99	14.8	0	0	0	8.64	0.00	-13.0774	3.05E-06	6.64E-06	yczF
AB5991_02110	29.41	33.29	27.19	0.72	0.9	2.03	29.96	1.22	-4.6222	5.40E-34	9.74E-33	gerKA
AB5991_02115	30.57	26.3	32.53	3.53	4.21	4.64	29.80	4.13	-2.8523	2.84E-20	1.96E-19	gerKC
AB5991_02120	33.19	17.75	29.46	2.63	3.61	3.14	26.80	3.13	-3.0995	7.69E-17	4.00E-16	gerKB
AB5991_02125	2595.25	1544.43	2684.26	6.64	7.56	10.34	2274.65	8.18	-8.1193	1.66E-83	2.20E-80	yclH
AB5991_02130	1712.11	858.23	1633.93	4.17	3.4	7.63	1401.42	5.07	-8.1116	1.33E-68	4.07E-66	yclI
AB5991_02135	10.31	4.37	9.04	14.94	29.85	25.74	7.91	23.51	1.5721	6.57E-05	0.000125348	yclJ
AB5991_02140	10.93	9.34	13.32	22.95	30.01	24.63	11.20	25.86	1.2078	2.05E-05	4.11E-05	yclK
AB5991_02145	23.75	25.13	25.57	87.08	115.59	73.73	24.82	92.13	1.8924	3.18E-11	1.06E-10	rapC
AB5991_02150	8.82	10.79	14.14	68.72	37.39	31.81	11.25	45.97	2.0309	4.65E-05	9.03E-05	phrC
AB5991_02155	202.84	202.82	315.71	6.55	10.22	13.04	240.46	9.94	-4.5969	5.74E-25	5.52E-24	yczM
AB5991_02160	1550.64	1077.19	2568.77	31.04	50.02	49.77	1732.20	43.61	-5.3118	4.62E-37	1.07E-35	yczN
AB5991_02165	6.36	7.29	5.81	11.67	9.57	4.16	6.49	8.47	0.3843	0.32641264	0.381904712	yclM
AB5991_02170	4.18	7.68	5.49	8.48	6.58	7	5.78	7.35	0.3465	0.318208706	0.373295944	yclN
AB5991_02175	2.1	0.7	1.22	1.66	4.66	3.3	1.34	3.21	1.2588	0.035499732	0.049186824	yclO
AB5991_02180	3.81	1.75	3.06	6.99	8.24	4.9	2.87	6.71	1.2236	0.005628827	0.008697304	yclP

AB5991_02185	20.08	13.92	18.84	51.51	65.75	55.79	17.61	57.68	1.7115	1.65E-09	4.76E-09	yclQ
AB5991_02190	0.58	4.98	1.45	4.21	4.34	1.05	2.34	3.20	0.4536	0.540752179	0.597974632	--
AB5991_02195	47.89	40.46	56.39	174.42	130.94	95.97	48.25	133.78	1.4713	7.58E-07	1.75E-06	ycnB
AB5991_02200	8.02	11.33	7.48	60.38	49.18	21.59	8.94	43.72	2.2893	9.56E-09	2.60E-08	ycnC
AB5991_02205	23.62	34.52	23.2	78.11	100.56	82.44	27.11	87.04	1.6826	9.37E-09	2.55E-08	nfrA2
AB5991_02210	6.28	8.07	5.37	27.3	13.41	19.02	6.57	19.91	1.5988	0.000495331	0.00086995	ycnE
AB5991_02215	26.4	38.98	22.09	28.71	28.62	11.8	29.16	23.04	-0.3395	0.419699072	0.477542984	yczG
AB5991_02220	7.41	7.84	7.25	5.19	3.7	3.26	7.50	4.05	-0.8890	0.006464417	0.009938134	gabR
AB5991_02225	5.79	6.33	5.01	2.4	2.53	3.58	5.71	2.84	-1.0093	0.005038287	0.007836678	gabT
AB5991_02230	147.96	339.78	140	211.3	246.87	232.43	209.25	230.20	0.1377	0.687592008	0.741762528	gabD
AB5991_02235	4.81	9.6	8.95	1.14	0.85	2.72	7.79	1.57	-2.3102	6.41E-06	1.36E-05	glcU
AB5991_02240	20.93	23.65	29.51	7.25	10.3	11.7	24.70	9.75	-1.3408	3.69E-05	7.26E-05	gdh
AB5991_02245	124.32	152.72	123.21	192.75	183.06	161.94	133.42	179.25	0.4260	0.097382829	0.127373918	ycnI
AB5991_02250	61.58	66.13	70.14	101.07	84.51	76.89	65.95	87.49	0.4077	0.112721909	0.145946756	ycnJ
AB5991_02255	41.95	57.34	48.58	56.61	44.63	41.32	49.29	47.52	-0.0528	0.873702578	0.913019194	ycnK
AB5991_02260	1.02	2.81	1.64	1.67	3.12	0	1.82	1.60	-0.1915	1	1	ycnL
AB5991_02265	1.51	2.31	1.08	0.41	1.66	0.27	1.63	0.78	-1.0663	0.130728273	0.167242903	mtlA
AB5991_02270	1.26	0.77	1.34	1.37	0.85	1.81	1.12	1.34	0.2580	1	1	mtlF
AB5991_02275	3.06	1.18	2.24	2.63	4.26	3.66	2.16	3.52	0.7032	0.150666845	0.189394759	mtlD
AB5991_02280	4.24	4.99	5.63	7.57	7.77	6.25	4.95	7.20	0.5389	0.109876961	0.142588697	ycsA
AB5991_02285	0	1.77	0.34	0.35	0.33	0	0.70	0.23	-1.6336	0.498321036	0.558361409	sipU
AB5991_02295	5.52	6.76	3.93	25.51	19.66	11.95	5.40	19.04	1.8171	4.43E-05	8.64E-05	ycsD
AB5991_02300	85.45	164.94	120.66	229.94	292.1	157.71	123.68	226.58	0.8734	0.007190725	0.010974008	yczO
AB5991_02305	6.02	10.18	3.09	17.04	12.75	5.74	6.43	11.84	0.8812	0.07142166	0.095109126	ycsE
AB5991_02310	8.64	10.72	7.74	6.1	4.28	2.78	9.03	4.39	-1.0421	0.008175416	0.01237217	pxpA
AB5991_02315	14.28	8.74	14.48	6.47	5.75	3.22	12.50	5.15	-1.2802	0.00038774	0.00068922	ycsG

AB5991_02320	13.08	8.15	4	6.35	3.09	3.29	8.41	4.24	-0.9869	0.048360651	0.065542711	ycsI
AB5991_02325	10.25	4.59	8.29	3.53	5.34	2.71	7.71	3.86	-0.9981	0.022521779	0.032216854	pxpB
AB5991_02330	14.52	6.26	5.56	7.61	6.39	2.14	8.78	5.38	-0.7066	0.160846417	0.200540383	pxpC
AB5991_02335	5.28	1.32	5.39	5.22	6.11	1.3	4.00	4.21	0.0750	0.95671187	0.992191913	kipR
AB5991_02340	480.59	640	580.78	10.41	22.35	31.39	567.12	21.38	-4.7291	2.30E-33	3.93E-32	lipC
AB5991_02345	52.17	49.92	47.14	8.79	5.23	4.77	49.74	6.26	-2.9895	1.51E-14	6.60E-14	yczI
AB5991_02350	5.02	6.91	7.38	17.75	11.5	15.63	6.44	14.96	1.2167	0.006673281	0.010235457	yczJ
AB5991_02355	144.09	129.32	122.8	94.23	94.96	81.89	132.07	90.36	-0.5475	0.032475039	0.045232683	pbpC
AB5991_02360	166.34	167.97	122.87	228.58	211.66	196.53	152.39	212.26	0.4780	0.067941317	0.090687385	ycsN
AB5991_02365	38.92	49.5	34.77	14.61	12.35	14.92	41.06	13.96	-1.5566	4.30E-08	1.10E-07	mtlR
AB5991_02370	17.93	16.24	15.6	59.55	72.03	40.25	16.59	57.28	1.7876	5.97E-09	1.64E-08	ydaB
AB5991_02375	6.66	8.89	4.53	44.45	36.36	31.79	6.69	37.53	2.4874	2.26E-12	8.37E-12	ydaC
AB5991_02380	1.89	8.87	4.49	47.03	52.77	54.31	5.08	51.37	3.3371	7.23E-17	3.78E-16	ydaD
AB5991_02385	4.3	8.56	6.52	57.73	77.74	77.26	6.46	70.91	3.4564	1.41E-22	1.15E-21	ydaE
AB5991_02390	1.96	4.21	1.4	6.05	8	7.8	2.52	7.28	1.5293	0.001597392	0.002646326	ydaF
AB5991_02395	94.01	156.92	96.42	319.27	369.22	389.47	115.78	359.32	1.6338	1.93E-08	5.09E-08	ydaG
AB5991_02400	27.67	60.23	26.97	2.43	0.91	0.72	38.29	1.35	-4.8224	8.62E-21	6.21E-20	amj
AB5991_02405	26.71	34.22	25.91	18.24	12.01	14.12	28.95	14.79	-0.9688	0.006156248	0.009471702	ydzA
AB5991_02410	8.73	12.97	7.55	3.16	3.38	3.15	9.75	3.23	-1.5939	0.00098403	0.001679236	lrpC
AB5991_02415	62.32	81.15	59.3	77.05	78.08	61.64	67.59	72.26	0.0963	0.715467012	0.766626957	topB
AB5991_02420	1.61	0.74	0.43	0.44	2.04	3.48	0.93	1.99	1.1002	0.261946662	0.3141587	mutT
AB5991_02425	44.11	84.85	47.29	799.31	899.43	943.14	58.75	880.63	3.9059	3.87E-31	5.73E-30	ydaP
AB5991_02430	561.01	550.43	368.1	1950.23	1727.5	1555.13	493.18	1744.29	1.8225	9.86E-11	3.14E-10	--
AB5991_02435	0.61	0.56	0	0.99	1.55	2.64	0.39	1.73	2.1464	0.019137816	0.027685344	--
AB5991_02440	0.56	0.34	1.2	0	0.96	0.81	0.70	0.59	-0.2466	0.846411399	0.888709589	--
AB5991_02445	85.3	220.02	102.08	19.88	8.27	7.33	135.80	11.83	-3.5214	2.46E-13	9.77E-13	ydzK

AB5991_02450	423.45	417.59	433.02	411.31	395.12	308.04	424.69	371.49	-0.1931	0.448918896	0.507301347	mntH
AB5991_02455	43.44	79.76	46.45	401.54	414.26	304.87	56.55	373.56	2.7237	1.55E-16	7.90E-16	--
AB5991_02460	63.44	101.84	50.78	233.03	285.89	269.52	72.02	262.81	1.8676	1.64E-09	4.72E-09	ydaT
AB5991_02465	2.87	3.24	2.83	3.12	2.92	0.96	2.98	2.33	-0.3529	0.506915492	0.566552608	ydbA
AB5991_02470	71.43	134.72	70.15	916.32	986.55	715.32	92.10	872.73	3.2443	4.73E-22	3.74E-21	--
AB5991_02475	0	0	0.57	0	0	4.58	0.19	1.53	3.0063	0.136988082	0.174305942	ydbB
AB5991_02480	0	0	0.54	8.74	8.18	1.09	0.18	6.00	5.0597	6.95E-05	0.000132334	ydbC
AB5991_02485	56.73	121.53	71.49	325.24	322.49	306.35	83.25	318.03	1.9336	7.38E-10	2.18E-09	ydbD
AB5991_02490	2.23	4.1	2.57	16.62	16.07	14.49	2.97	15.73	2.4063	5.39E-12	1.93E-11	dctB
AB5991_02495	4.72	7.84	3.85	1.47	2.63	2.56	5.47	2.22	-1.3010	0.0020793	0.003404908	dctS
AB5991_02500	3.98	4.39	2.84	4.04	2.16	2.3	3.74	2.83	-0.3993	0.497809697	0.558094564	dctR
AB5991_02505	42.83	24.64	25.04	182.92	80.65	22.1	30.84	95.22	1.6267	0.002622601	0.00425249	dctA
AB5991_02510	38.96	61.15	49.93	19.98	18.17	11.71	50.01	16.62	-1.5894	3.12E-07	7.47E-07	ydbI
AB5991_02515	0	0	0	0	0	0	0.00	0.00	0.0000	1	1	--
AB5991_02520	0.78	1.07	3.54	15.27	12.9	10.13	1.80	12.77	2.8290	3.16E-09	8.91E-09	ydbJ
AB5991_02525	2.35	4.32	4.28	18.43	19.64	12.23	3.65	16.77	2.1996	1.54E-08	4.11E-08	ydbK
AB5991_02530	10.22	37.54	28.76	45.05	58.03	57.07	25.51	53.38	1.0655	0.007952809	0.012044472	ydbL
AB5991_02535	50.31	82.25	57.18	198.64	136.44	129.59	63.25	154.89	1.2922	1.43E-05	2.91E-05	ydbM
AB5991_02540	0	9.22	2.15	8.74	2.04	1.09	3.79	3.96	0.0621	1	1	ydbN
AB5991_02545	1.1	0	0	1.19	0	3.56	0.37	1.58	2.1104	0.403466913	0.460790081	--
AB5991_02550	12.22	22.05	16.38	36.03	22.76	24.65	16.88	27.81	0.7202	0.025998562	0.036740316	ydbO
AB5991_02555	27.03	33.09	21.08	20.21	28.08	15.85	27.07	21.38	-0.3403	0.372454722	0.430197121	ydbP
AB5991_02560	205.38	249.38	189.2	106.8	76.9	76.57	214.65	86.76	-1.3070	1.89E-06	4.18E-06	ddl
AB5991_02565	160.62	182.85	138.99	52.08	38.29	33.46	160.82	41.28	-1.9620	5.83E-12	2.08E-11	murF
AB5991_02570	552.35	521.64	536.8	301.95	236.85	161.54	536.93	233.45	-1.2016	2.86E-05	5.69E-05	cshA
AB5991_02575	3.01	7.61	2.82	7.37	2.3	4.48	4.48	4.72	0.0743	0.871592753	0.911294055	ydbS

AB5991_02580	45.86	61.59	46.04	10.88	8.07	6.21	51.16	8.39	-2.6089	1.35E-16	6.91E-16	ydbT
AB5991_02585	98.21	82.97	97.61	6.55	4.6	3.91	92.93	5.02	-4.2104	1.04E-36	2.28E-35	ydcA
AB5991_02590	19.75	26.3	23.24	5.91	2.01	4.28	23.10	4.07	-2.5058	5.03E-09	1.39E-08	acpS
AB5991_02595	586.32	1116.42	592.62	44.07	39.25	28.28	765.12	37.20	-4.3623	1.76E-32	2.80E-31	ydcC
AB5991_02600	29.51	36.31	24.62	21.51	28.77	19.57	30.15	23.28	-0.3727	0.199906702	0.245539596	alr1
AB5991_02605	113.45	120.05	153.54	130.36	154.6	53.43	129.01	112.80	-0.1938	0.594740258	0.651044667	ndoAI
AB5991_02610	148.31	165.47	177.32	225.7	237.42	115.95	163.70	193.02	0.2377	0.450080088	0.508468856	ndoA
AB5991_02615	11.39	31.38	13.35	39.32	30.55	18.02	18.71	29.30	0.6472	0.112659465	0.145913482	rsbRA
AB5991_02620	6.91	10.88	7.39	46.73	32.67	18.71	8.39	32.70	1.9621	5.43E-06	1.16E-05	rsbS
AB5991_02625	6.74	15.69	5.77	52.32	39.81	34.56	9.40	42.23	2.1675	4.15E-08	1.06E-07	rsbT
AB5991_02630	3.59	5.27	3.07	38.81	43.25	22.13	3.98	34.73	3.1266	2.30E-16	1.15E-15	rsbU
AB5991_02635	98.59	217.24	94.89	629.06	711.31	741.73	136.91	694.03	2.3418	1.16E-11	4.02E-11	rsbV
AB5991_02640	312.48	585.45	313.75	2756.2	3145.2	3245.63	403.89	3049.01	2.9163	2.25E-19	1.43E-18	rsbW
AB5991_02645	320.95	546.42	318.48	3120.13	3539.05	3976.46	395.28	3545.21	3.1649	6.86E-25	6.54E-24	sigB
AB5991_02650	184.06	245.6	187.82	1398.35	1667.89	1901.83	205.83	1656.02	3.0082	9.06E-26	9.37E-25	rsbX
AB5991_02655	0	0	0	1.07	0	0	0.00	0.36	8.4784	1	1	--
AB5991_02665	1.64	3.76	2.19	10.7	10.43	10.2	2.53	10.44	2.0454	9.96E-06	2.05E-05	ydcG
AB5991_02670	1.63	1.5	0.44	12.4	5.8	9.25	1.19	9.15	2.9428	1.72E-06	3.82E-06	ydcH
AB5991_02675	4.85	10.45	5.19	43.14	39.18	28.72	6.83	37.01	2.4381	5.93E-12	2.11E-11	ydcI
AB5991_02680	0	0	0	0	0	0	0.00	0.00	0.0000	1	1	cmpA
AB5991_02685	2	0	1.28	9.11	19.09	5.62	1.09	11.27	3.3661	1.87E-06	4.15E-06	ydcK
AB5991_02725	0.82	0	0	0.71	0.33	0.35	0.27	0.46	0.7614	0.808605086	0.852621029	int
AB5991_02730	0.35	0	0	0	1.44	0	0.12	0.48	2.0406	0.423051277	0.48053107	immA
AB5991_02735	0	0	0	0	0	0	0.00	0.00	0.0000	1	1	immR
AB5991_02740	1.85	0	0.99	0	0.94	0	0.95	0.31	-1.5952	0.638627904	0.694601865	xis
AB5991_02745	0.69	0	0	0.75	0	0	0.23	0.25	0.1203	1	1	ydzL

AB5991_02750	1.39	1.27	0.74	0	0	0	1.13	0.00	-10.1464	0.136704139	0.174085232	ydcO
AB5991_02755	0	0	0	0	0	0	0.00	0.00	0.0000	1	1	--
AB5991_02760	6.17	6.1	8.12	0	0	0	6.80	0.00	-12.7306	2.21E-09	6.31E-09	ydcP
AB5991_02765	3.76	2.3	2.95	0.14	0	0.14	3.00	0.09	-5.0080	1.75E-12	6.54E-12	ydcQ
AB5991_02770	7.17	7.21	6.21	0.37	0.35	0.55	6.86	0.42	-4.0190	8.60E-15	3.81E-14	nicK
AB5991_02775	2.15	2.63	1.53	0	0	0	2.10	0.00	-11.0385	0.020538224	0.029560452	--
AB5991_02780	4.74	1.24	2.9	0.74	0	1.47	2.96	0.74	-2.0065	0.061089456	0.081871829	ydcT
AB5991_02785	10.8	3.13	7.9	0	0	0.62	7.28	0.21	-5.1379	1.44E-06	3.23E-06	--
AB5991_02790	2.34	5.37	3.75	1.91	0.6	0.63	3.82	1.05	-1.8678	0.02648055	0.037381537	yddA
AB5991_02795	1.53	0.93	1.09	0.18	0	0.18	1.18	0.12	-3.3018	0.001403239	0.002346216	yddB
AB5991_02800	2.18	1.33	1.55	0	0	0	1.69	0.00	-10.7200	0.038250378	0.052703766	yddC
AB5991_02805	1.72	0	0.37	0	0.35	0	0.70	0.12	-2.5781	0.168198099	0.20904997	yddD
AB5991_02810	4.92	4.52	5.03	0.47	0.29	0.39	4.82	0.38	-3.6534	4.92E-18	2.83E-17	yddE
AB5991_02815	2.76	3.04	1.18	0.6	0	0.6	2.33	0.40	-2.5402	0.036443248	0.050388627	yddF
AB5991_02820	2.05	2.44	2.92	0.24	0.53	0.72	2.47	0.50	-2.3142	5.86E-07	1.36E-06	yddG
AB5991_02825	31.23	40.55	36.77	20.58	16.05	12.63	36.18	16.42	-1.1399	0.0002865	0.000513398	--
AB5991_02830	4.2	4.36	5.27	0	0.19	0.2	4.61	0.13	-5.1482	1.85E-13	7.37E-13	yddH
AB5991_02835	5.35	9.16	6.48	0	0.36	0	7.00	0.12	-5.8656	4.49E-10	1.35E-09	yddI
AB5991_02840	12.33	14.81	7.1	75.85	91.74	69.84	11.41	79.14	2.7937	6.67E-16	3.21E-15	yddJ
AB5991_02845	5.53	7.9	4.77	8.19	4.07	1.83	6.07	4.70	-0.3693	0.46679384	0.526449388	rapI
AB5991_02850	0	0	0	1.96	0	0	0.00	0.65	9.3517	0.512550498	0.569081258	--
AB5991_02855	11.86	10.01	7.06	2.24	3.01	0.9	9.64	2.05	-2.2339	3.21E-07	7.67E-07	--
AB5991_02865	0	0	0	0	0.84	0	0.00	0.28	8.1293	1	1	--
AB5991_02870	5.54	10.34	5.82	1.66	1.75	0.93	7.23	1.45	-2.3219	2.38E-08	6.23E-08	--
AB5991_02875	12.05	9.75	10.74	2.18	4.09	2.02	10.85	2.76	-1.9728	4.16E-08	1.07E-07	--
AB5991_02880	30.53	28.4	27.28	1.97	2.04	0.87	28.74	1.63	-4.1429	5.74E-27	6.46E-26	ywqM

AB5991_02885	1.28	1.77	2.74	2.09	3.91	2.43	1.93	2.81	0.5420	0.429087059	0.486690863	ydaF
AB5991_02890	1.39	1.92	1.49	2.27	5.67	0.38	1.60	2.77	0.7935	0.355898275	0.412754688	--
AB5991_02895	0.94	4.43	2.69	2.51	4.38	0.8	2.69	2.56	-0.0678	0.971252513	1	yobL
AB5991_02900	0	0	0	0	0	0.84	0.00	0.28	8.1293	1	1	MJ0272
AB5991_02905	4.08	4.69	3.28	0.56	3.12	0.55	4.02	1.41	-1.5103	0.048688123	0.065964018	--
AB5991_02910	0	0	0	3.33	2.08	1.11	0.00	2.17	11.0857	0.039318927	0.054082251	ydzM
AB5991_02915	10	10.15	12.38	78.98	85.42	78.61	10.84	81.00	2.9012	4.77E-23	3.97E-22	yddT
AB5991_02920	0	3.57	3.12	0	0	0	2.23	0.00	-11.1228	0.042818977	0.058551708	ydzN
AB5991_02925	1.83	0	1.95	3.31	1.86	1.98	1.26	2.38	0.9196	0.25143094	0.302463576	ydeA
AB5991_02930	43.16	64.4	100.01	44.01	37.52	15.58	69.19	32.37	-1.0959	0.01085719	0.016129405	cspC
AB5991_02935	46.56	43.82	53.55	91.06	79.24	54.63	47.98	74.98	0.6441	0.024842865	0.035245093	ydeB
AB5991_02940	53.53	50.83	63.78	6.64	4.76	5.07	56.05	5.49	-3.3518	8.75E-27	9.70E-26	ydzE
AB5991_02945	31.98	26.52	30.67	2.47	2.1	2.01	29.72	2.19	-3.7604	2.23E-25	2.24E-24	ydeC
AB5991_02955	59.84	58.88	61.81	16.44	14.97	20.34	60.18	17.25	-1.8026	5.22E-11	1.71E-10	ydeE
AB5991_02960	23.42	20.79	22.13	2.26	3.44	1.41	22.11	2.37	-3.2220	7.79E-20	5.20E-19	ydeF
AB5991_02970	105.56	180.55	127.83	6.81	4.91	4.7	137.98	5.47	-4.6559	1.20E-33	2.10E-32	ydeH
AB5991_02975	15.52	20.11	17.9	4.63	1.86	3.62	17.84	3.37	-2.4046	2.10E-09	5.99E-09	ydeI
AB5991_02980	55.32	66.38	45.1	16.98	18.4	12.45	55.60	15.94	-1.8021	3.13E-09	8.81E-09	ydeJ
AB5991_02985	22.09	25.81	15.57	2.18	0	1.09	21.16	1.09	-4.2787	7.69E-14	3.16E-13	--
AB5991_02990	0.65	0	0	0	0	0	0.22	0.00	-7.7593	1	1	ptsH
AB5991_02995	11.59	6.69	9.56	1.08	0.67	2.15	9.28	1.30	-2.8356	1.11E-07	2.75E-07	yxbF
AB5991_03000	7.53	6.91	8.5	2.28	2.55	3.4	7.65	2.74	-1.4789	0.000115226	0.000214469	ydeK
AB5991_03005	9.87	10.25	10.28	4.66	3.17	1.83	10.13	3.22	-1.6540	4.19E-06	9.01E-06	ydeL
AB5991_03010	11.46	7.79	5.9	2.77	3.45	0.46	8.38	2.23	-1.9126	0.000730207	0.001259076	ydeM
AB5991_03015	11.67	24.33	19.23	41.17	43.66	43.71	18.41	42.85	1.2187	0.000135285	0.000250218	ydeN
AB5991_03025	8.7	3.8	6.64	4.05	5.06	1.79	6.38	3.63	-0.8123	0.073200981	0.097217757	ydeO

AB5991_03030	2.15	4.94	2.88	0	0	1.16	3.32	0.39	-3.1035	0.006864317	0.010507614	aseR
AB5991_03035	105.99	136.26	104.77	4.21	5.49	4.94	115.67	4.88	-4.5670	2.24E-45	8.80E-44	ydfA
AB5991_03045	5.98	8.45	7.62	6	7.26	4.23	7.35	5.83	-0.3342	0.421564152	0.479343128	ydfB
AB5991_03050	14.13	10.45	14.06	0	0.2	0	12.88	0.07	-7.5940	4.80E-27	5.44E-26	ydfC
AB5991_03055	6.99	7.1	7.07	0.27	0.13	1.08	7.05	0.49	-3.8377	2.87E-15	1.32E-14	ydfD
AB5991_03060	91.06	117.3	99.13	10.22	5.35	5.83	102.50	7.13	-3.8449	1.51E-30	2.15E-29	GAD1
AB5991_03065	3.77	7.45	8.05	7.88	5.6	3.14	6.42	5.54	-0.2134	0.726019467	0.776457262	ydfE
AB5991_03070	3087.6	2861.76	2990.8	29.16	45.65	47.69	2980.05	40.83	-6.1894	6.80E-77	3.86E-74	ydfF
AB5991_03075	116.02	140.53	112.32	279.38	270.14	226.51	122.96	258.68	1.0730	5.05E-05	9.76E-05	ydfG
AB5991_03080	4.02	0	2.15	0	0	0	2.06	0.00	-11.0061	0.042532635	0.058227037	ydzP
AB5991_03085	5.02	6.91	6.71	0	0	0	6.21	0.00	-12.6012	0.000569022	0.000994536	ydzQ
AB5991_03090	1.03	0.81	0.32	3.21	1.8	1.12	0.72	2.04	1.5049	0.021298585	0.030577252	ydfH
AB5991_03095	5.63	5.69	2.41	3.67	1.15	0.3	4.58	1.71	-1.4231	0.045662097	0.062139886	ydfI
AB5991_03100	2.33	3.97	2.75	0.36	0.25	0.36	3.02	0.32	-3.2219	6.14E-10	1.82E-09	ydfJ
AB5991_03105	83.87	109.16	106.6	26.56	52.15	24.7	99.88	34.47	-1.5348	4.29E-06	9.21E-06	nap
AB5991_03110	0.52	2.89	2.8	9.69	16.26	6.52	2.07	10.82	2.3864	1.66E-05	3.36E-05	ydfK
AB5991_03115	2.89	5.72	3.8	1.21	2.49	0.24	4.14	1.31	-1.6552	0.007199412	0.010983045	ydfL
AB5991_03120	1.01	1.11	0.65	0.44	0.21	0.44	0.92	0.36	-1.3456	0.183476569	0.226937905	mneP
AB5991_03125	0	0	0.62	0.32	0	0.32	0.21	0.21	0.0458	1	1	mhqN
AB5991_03130	1.35	1.41	0.82	13.19	11.36	8.75	1.19	11.10	3.2175	5.40E-13	2.10E-12	mhqO
AB5991_03135	86.2	45.1	69.39	11.09	19.34	33.11	66.90	21.18	-1.6592	3.60E-05	7.09E-05	mhqP
AB5991_03140	0	0.98	0	0	0	0	0.33	0.00	-8.3517	1	1	ydfQ
AB5991_03145	1.46	2.49	2.28	9.07	7.18	5.16	2.08	7.14	1.7810	8.79E-06	1.83E-05	--
AB5991_03150	2558.71	3408.53	3291.7	51.43	43.47	61.09	3086.31	52.00	-5.8913	2.12E-68	5.49E-66	ydzH
AB5991_03155	18631.3	26553.47	24049.95	442.64	602.56	684.15	23078.24	576.45	-5.3232	1.21E-60	1.41E-58	--
AB5991_03160	4882.57	5065.99	5261.61	76.59	136.18	215.99	5070.06	142.92	-5.1487	6.34E-39	1.64E-37	ydzR

AB5991_03170	5.62	6.09	5.46	0	0.26	0	5.72	0.09	-6.0452	1.08E-13	4.39E-13	ydfS
AB5991_03175	114.22	76.06	100.23	6.37	8.94	4.98	96.84	6.76	-3.8397	8.19E-27	9.14E-26	cotP
AB5991_03180	60.99	34.15	78.75	2.43	3.03	12.08	57.96	5.85	-3.3095	3.27E-10	9.99E-10	ydgA
AB5991_03185	76.14	52.28	97	4.32	5.39	3.58	75.14	4.43	-4.0842	4.31E-21	3.15E-20	ydgB
AB5991_03190	4.22	6.64	6.44	2.95	7.05	3.26	5.77	4.42	-0.3837	0.475833818	0.535734645	ydgC
AB5991_03195	4.72	4.81	2.24	2.85	2.67	1.7	3.92	2.41	-0.7050	0.356008179	0.412761599	ydgD
AB5991_03200	3.81	2.1	1.22	16.17	12.03	14.03	2.38	14.08	2.5663	4.21E-08	1.08E-07	ydgE
AB5991_03205	782.48	667.21	779.51	5.73	7.04	8.08	743.07	6.95	-6.7403	3.47E-86	7.43E-83	vmlR
AB5991_03210	48.7	60.5	53.48	17.99	13.63	8.53	54.23	13.38	-2.0186	4.41E-10	1.33E-09	ydgF
AB5991_03215	65.13	72.9	61.82	106.81	94.99	103.67	66.62	101.82	0.6121	0.019282055	0.027863551	dinB
AB5991_03220	0.79	0	0	0.86	0.4	0.85	0.26	0.70	1.4173	0.480280021	0.539822237	ydgG
AB5991_03225	5.44	6.99	5.09	5.32	5.12	3.9	5.84	4.78	-0.2890	0.368236551	0.425820427	ydgH
AB5991_03230	0.57	1.05	2.45	3.12	6.42	5.28	1.36	4.94	1.8644	0.002273822	0.003709674	ydgI
AB5991_03235	0.73	0.67	0	3.97	1.49	1.58	0.47	2.35	2.3301	0.010785328	0.016046661	ydgJ
AB5991_03240	2.69	1.65	1.92	1.3	3.35	2.59	2.09	2.41	0.2098	0.755972166	0.804816481	ydgK
AB5991_03245	0.49	0.9	1.57	0	0.5	0.8	0.99	0.43	-1.1871	0.365989838	0.423345658	ydhB
AB5991_03250	37.76	57.04	41.52	7.28	4.36	2.9	45.44	4.85	-3.2289	1.81E-17	9.84E-17	ydhC
AB5991_03255	37.35	51.77	34.59	4.05	3.35	6.04	41.24	4.48	-3.2024	7.62E-22	5.84E-21	ydhD
AB5991_03260	61.16	100.29	68.82	167.95	232.43	201.59	76.76	200.66	1.3864	1.50E-06	3.36E-06	ydhE
AB5991_03265	8.39	8.87	7.88	3.59	2.07	4.13	8.38	3.26	-1.3606	0.000671662	0.001163179	ydhF
AB5991_03270	47.5	45.4	41.05	8.63	7.42	6.06	44.65	7.37	-2.5989	2.08E-19	1.33E-18	phoB
AB5991_03275	7.77	3.57	3.12	1.59	2.47	2.63	4.82	2.23	-1.1120	0.091655611	0.120199615	fra
AB5991_03280	13.43	18.66	14.36	1.58	2.22	0.39	15.48	1.40	-3.4707	1.57E-12	5.87E-12	ydhH
AB5991_03285	0.75	2.07	3.62	0.41	0	0.41	2.15	0.27	-2.9734	0.009792024	0.014678795	ydhI
AB5991_03290	29.02	36.31	28.66	3.62	3.39	1.6	31.33	2.87	-3.4484	1.49E-21	1.11E-20	ydhJ
AB5991_03295	16.01	13.36	11.52	113.64	145.15	148.4	13.63	135.73	3.3159	2.47E-25	2.45E-24	ydhK

AB5991_03300	9.6	9.1	11.59	15.16	22.07	14.25	10.10	17.16	0.7652	0.015014194	0.022033024	pbuE
AB5991_03305	3.48	9.57	6.81	61.12	97.88	102.85	6.62	87.28	3.7208	3.48E-19	2.18E-18	gmuB
AB5991_03320	14.48	15.67	13.96	35.01	58.42	58.78	14.70	50.74	1.7869	5.30E-09	1.47E-08	gmuD
AB5991_03325	10.89	16.27	11.37	29.46	51.79	54.26	12.84	45.17	1.8143	9.54E-08	2.37E-07	gmuR
AB5991_03330	10.04	11.43	13.75	18.35	39.86	50	11.74	36.07	1.6194	1.77E-05	3.59E-05	gmuE
AB5991_03340	15.77	25.6	18.81	55.24	112.67	132.6	20.06	100.17	2.3201	1.38E-10	4.36E-10	gmuG
AB5991_03385	2.96	3.39	4.15	4.02	3.39	0.8	3.50	2.74	-0.3549	0.517592714	0.573802532	thiL
AB5991_03390	2.27	1.39	1.62	5.36	2.31	1.23	1.76	2.97	0.7533	0.33996659	0.396477923	tsaE
AB5991_03395	10.22	5.29	8.12	5.7	6.4	0.85	7.88	4.32	-0.8677	0.117083231	0.151100913	tsaB
AB5991_03400	11.1	8.01	16.11	6.9	4.44	5.15	11.74	5.50	-1.0948	0.009796578	0.01468008	rimI
AB5991_03405	37.5	35.07	40.85	15.48	16.61	11.65	37.81	14.58	-1.3746	5.82E-07	1.36E-06	tsaD
AB5991_03410	68.21	62.28	60.82	31.18	34.05	26.07	63.77	30.43	-1.0672	3.82E-05	7.49E-05	ydiF
AB5991_03415	85.62	100.93	78	32.96	34.43	22.12	88.18	29.84	-1.5634	4.69E-08	1.20E-07	moaC
AB5991_03420	93.72	98.85	82.03	37.92	44.57	35.93	91.53	39.47	-1.2134	6.80E-06	1.44E-05	rex
AB5991_03425	135.04	62.94	97.76	127.66	163.87	127.07	98.58	139.53	0.5012	0.129667346	0.166099687	tatAy
AB5991_03430	255.18	208.24	243.58	269.82	261.39	241.96	235.67	257.72	0.1291	0.612721667	0.66843894	tatC2
AB5991_03435	4.71	8.64	3.02	11.26	3.83	4.08	5.46	6.39	0.2278	0.789243051	0.835532966	ydiK
AB5991_03440	4.67	14.9	7.63	13.11	10.51	5.32	9.07	9.65	0.0895	0.850518377	0.892549808	ydiL
AB5991_03445	304.42	293.46	341.15	289.01	359.53	346.7	313.01	331.75	0.0839	0.74545999	0.795117276	groES
AB5991_03450	1302.79	1629.56	1259.49	1499.29	1666.9	1632.55	1397.28	1599.58	0.1951	0.427983774	0.485716938	groEL
AB5991_03455	0.9	0.41	0	0.73	0	0	0.44	0.24	-0.8436	0.760740204	0.809458561	--
AB5991_03460	48.77	46.01	40.91	89.24	54.9	66.88	45.23	70.34	0.6371	0.027843531	0.039166369	--
AB5991_03465	40.67	35.15	35.15	38.81	41.84	40.74	36.99	40.46	0.1295	0.609455963	0.665607709	gutR
AB5991_03470	6.13	8.75	5.82	3.33	2.43	2.4	6.90	2.72	-1.3430	0.000555084	0.000971886	gutB
AB5991_03475	12.34	13.35	9.86	3.67	1.19	2.25	11.85	2.37	-2.3219	2.45E-09	6.97E-09	gutA
AB5991_03480	78.83	116.49	83.1	248.84	282.72	317.57	92.81	283.04	1.6087	6.71E-09	1.84E-08	ydjE

AB5991_03490	4.23	8.73	3.39	2.11	0.72	0.76	5.45	1.20	-2.1872	0.000138036	0.000255069	ydjG
AB5991_03495	0.47	4.34	0.51	0.26	0.24	0	1.77	0.17	-3.4114	0.014286936	0.020989058	ydjH
AB5991_03500	53.18	88.44	44.15	1.42	1.89	2.42	61.92	1.91	-5.0188	4.72E-33	7.82E-32	ydjI
AB5991_03505	16.03	32.99	16.01	197.54	239.36	188.03	21.68	208.31	3.2645	1.37E-21	1.03E-20	ydjJ
AB5991_03510	17.67	35.01	13.59	5.25	6.6	4.13	22.09	5.33	-2.0521	2.32E-07	5.59E-07	iolT
AB5991_03515	275.38	221.9	188.84	918.51	1162.95	1201.03	228.71	1094.16	2.2583	1.03E-15	4.85E-15	bdhA
AB5991_03520	0.97	2.68	1.04	2.64	8.9	1.58	1.56	4.37	1.4841	0.065408571	0.087424246	ydjM
AB5991_03525	14.33	20.29	14.4	8.64	10.37	6.73	16.34	8.58	-0.9294	0.003875164	0.006116167	ydjN
AB5991_03530	0.45	0.62	0.61	1.39	0.97	0.74	0.56	1.03	0.8838	0.222382897	0.270801129	insK
AB5991_03535	54.57	54.27	80.24	2.47	2.31	6.15	63.03	3.64	-4.1126	4.01E-16	1.96E-15	ydzJ
AB5991_03540	150.62	279.74	207.1	123.57	112.13	63.36	212.49	99.69	-1.0919	0.001194456	0.002021751	ydjO
AB5991_03545	214.2	253.8	231.67	8.91	10.14	9.59	233.22	9.55	-4.6106	3.52E-50	2.05E-48	ydjP
AB5991_03555	965.99	993.73	1041.05	74.71	194.58	362.52	1000.26	210.60	-2.2478	2.36E-07	5.70E-07	cotA
AB5991_03560	88.2	80.74	89.93	26.77	20.22	26.78	86.29	24.59	-1.8111	1.78E-11	6.08E-11	gabP
AB5991_03565	3.77	0	8.05	0	0	0	3.94	0.00	-11.9440	0.000806012	0.001384375	ydzX
AB5991_03570	130.02	133.06	118.68	10.81	5.27	5.6	127.25	7.23	-4.1382	3.01E-32	4.68E-31	mneS
AB5991_03575	35.47	34.12	33.52	13.06	10.7	8.94	34.37	10.90	-1.6568	6.41E-09	1.76E-08	yeaC
AB5991_03580	25.82	13.31	19.38	15.27	13.06	9.32	19.50	12.55	-0.6360	0.053910252	0.072691888	yeaD
AB5991_03585	35.59	23.69	24.53	14.38	14.87	9.46	27.94	12.90	-1.1144	0.000263998	0.00047479	yebA
AB5991_03590	24.03	17.86	21.56	96.38	96.27	64.46	21.15	85.70	2.0187	3.35E-12	1.22E-11	guaA
AB5991_03595	0.93	0.85	2.97	1.76	0.71	0.5	1.58	0.99	-0.6775	0.404268639	0.461572963	--
AB5991_03600	4.24	7.02	4.24	7.43	3.75	3.11	5.17	4.76	-0.1173	0.853641715	0.895354266	pbuG
AB5991_03605	78.23	92.47	80.78	73.35	68.87	73.98	83.83	72.07	-0.2181	0.406007076	0.463291408	yebC
AB5991_03610	1.08	9.88	2.3	10.53	6.57	0	4.42	5.70	0.3669	0.701291153	0.753878497	yebD
AB5991_03615	338.7	469.43	311.36	44.98	65.63	60.64	373.16	57.08	-2.7087	2.02E-19	1.30E-18	yebE
AB5991_03620	85.81	100.57	73.22	11.91	9.29	1.98	86.53	7.73	-3.4853	1.63E-16	8.29E-16	yebG

AB5991_03625	8.5	21.04	13.44	34.17	19.56	28.01	14.33	27.25	0.9274	0.013877759	0.020433289	purE
AB5991_03630	29.73	29.91	22.49	97.52	50.54	64.19	27.38	70.75	1.3698	1.78E-05	3.59E-05	purK
AB5991_03635	42.26	35.08	32.66	222.67	118.81	121.84	36.67	154.44	2.0745	7.82E-11	2.53E-10	purB
AB5991_03640	50.29	36.11	45.26	245.32	131	131.25	43.89	169.19	1.9468	2.23E-09	6.35E-09	purC
AB5991_03645	18.43	9.11	21.22	84.8	65.65	67.52	16.25	72.66	2.1604	9.77E-10	2.86E-09	purS
AB5991_03650	55.49	42.7	51.71	233.94	154.91	138.45	49.97	175.77	1.8146	1.30E-09	3.78E-09	purQ
AB5991_03655	53.28	43.33	49	257.43	155.73	128.07	48.54	180.41	1.8941	2.02E-09	5.77E-09	purL
AB5991_03660	65.3	68.42	59.7	376.54	242.19	198.26	64.47	272.33	2.0786	1.34E-11	4.62E-11	purF
AB5991_03665	29.86	28.69	22.28	159.57	118.57	96.61	26.94	124.92	2.2130	1.51E-13	6.04E-13	purM
AB5991_03670	23.98	24.27	25.97	161.81	113.88	99.83	24.74	125.17	2.3390	1.67E-14	7.27E-14	purN
AB5991_03675	43.92	41.41	29.64	208.84	183.24	152.82	38.32	181.63	2.2447	5.63E-15	2.54E-14	purH
AB5991_03680	58.26	49.69	29.7	215.79	226.87	191.34	45.88	211.33	2.2035	5.98E-13	2.32E-12	purD
AB5991_03685	1.28	0	1.83	8.83	11.74	5.09	1.04	8.55	3.0445	6.46E-06	1.37E-05	yezC
AB5991_03690	0.43	0	0	1.85	3.17	0.77	0.14	1.93	3.7512	9.96E-05	0.000186773	yecA
AB5991_03700	36.92	86.45	63.1	66.88	55.09	40.19	62.16	54.05	-0.2015	0.550086539	0.607282082	yerA
AB5991_03705	69.69	112.96	96.45	126.12	137.23	102.55	93.03	121.97	0.3907	0.159257709	0.198778312	yerB
AB5991_03710	53.36	65.32	57.68	93.61	68.91	72.05	58.79	78.19	0.4115	0.139429347	0.176723248	yerC
AB5991_03715	31.5	54.26	33.2	557.73	618.68	633.98	39.65	603.46	3.9278	2.06E-35	4.28E-34	yerD
AB5991_03720	33.15	41.06	35.73	2.86	2.14	1.42	36.65	2.14	-4.0980	2.98E-26	3.17E-25	pcrB
AB5991_03725	66.44	64.58	61.73	25.15	25.94	20.98	64.25	24.02	-1.4193	3.92E-08	1.01E-07	pcrA
AB5991_03730	234.96	264.26	211.11	69.35	50.6	52.64	236.78	57.53	-2.0411	1.75E-13	7.00E-13	ligA
AB5991_03735	408.85	423.57	382.53	63.38	50.2	55.03	404.98	56.20	-2.8491	2.41E-25	2.40E-24	yerH
AB5991_03740	0.45	0.62	0.61	1.39	0.97	0.74	0.56	1.03	0.8838	0.222599938	0.270825006	insK
AB5991_03745	4189.23	4467.22	4372.91	458.88	556.06	583.11	4343.12	532.68	-3.0274	1.04E-30	1.52E-29	amiN
AB5991_03750	61.54	103.98	74.94	9.28	6.58	11.76	80.15	9.21	-3.1220	9.32E-20	6.19E-19	sapB
AB5991_03755	3.06	4.04	3.79	21.4	18.78	27.65	3.63	22.61	2.6389	6.30E-16	3.04E-15	opuE

AB5991_03760	1.86	2.28	2.66	1.35	6.95	1.34	2.27	3.21	0.5035	0.641790257	0.69689612	gatC
AB5991_03765	17.11	15.93	13.12	79.95	72.93	53.41	15.39	68.76	2.1600	3.38E-13	1.33E-12	gatA
AB5991_03770	40.29	45.69	36.74	202.49	216.23	196.48	40.91	205.07	2.3257	3.32E-18	1.94E-17	gatB
AB5991_03780	46.12	41.71	38.98	203.55	165.62	110.56	42.27	159.91	1.9196	1.06E-10	3.38E-10	swrC
AB5991_03785	18.83	21.83	17.38	67.9	56.68	46.77	19.35	57.12	1.5618	3.65E-08	9.41E-08	dagK
AB5991_03790	9.71	8.92	9.55	30.27	26.18	22.17	9.39	26.21	1.4802	1.68E-07	4.10E-07	rlmCD
AB5991_03795	6.44	7.6	6.39	0	0	0	6.81	0.00	-12.7334	9.96E-10	2.91E-09	yefB
AB5991_03800	0	3.16	0	0	0	0	1.05	0.00	-10.0407	0.273731401	0.325347926	--
AB5991_03805	1.74	1.43	2.11	4.3	4.39	4.41	1.76	4.37	1.3110	4.49E-05	8.74E-05	--
AB5991_03810	22.82	8.38	26.36	1.99	1.86	0	19.19	1.28	-3.9021	6.69E-08	1.69E-07	--
AB5991_03815	71.31	84.02	64.02	13.27	5.82	6.19	73.12	8.43	-3.1172	1.22E-17	6.76E-17	yezG
AB5991_03820	46.76	57.79	41.45	10.17	4.85	3.31	48.67	6.11	-2.9937	2.68E-14	1.14E-13	yeeF
AB5991_03830	551.68	468.64	436.32	650.92	711.76	740.61	485.55	701.10	0.5300	0.037048682	0.051207907	rapH
AB5991_03835	99.72	57.22	63.32	109.59	123.7	140.56	73.42	124.62	0.7633	0.016323993	0.023814319	phrH
AB5991_03840	553.8	537.48	409.68	860.04	919.28	912.81	500.32	897.38	0.8429	0.001098019	0.001868137	yeeI
AB5991_03845	491.94	245.96	443.2	81.11	148.83	212.12	393.70	147.35	-1.4178	0.000128743	0.000238451	yeeK
AB5991_03850	8.03	14.18	8.92	33.6	19.5	21.41	10.38	24.84	1.2591	0.000453259	0.000798887	yezE
AB5991_03855	59.52	90.64	73.75	15.79	13.3	5.5	74.64	11.53	-2.6945	6.22E-12	2.21E-11	cotJA
AB5991_03860	21.22	32.69	39.54	10.42	5.57	2.22	31.15	6.07	-2.3595	2.06E-06	4.56E-06	cotJB
AB5991_03865	90.06	206.7	152.6	37.94	25.17	26.43	149.79	29.85	-2.3273	8.51E-11	2.73E-10	cotJC
AB5991_03870	50.26	94.13	74.4	75.3	100.62	89	72.93	88.31	0.2760	0.357720311	0.414504627	yesJ
AB5991_03880	5.45	3.16	5.83	20.28	21.32	18.01	4.81	19.87	2.0455	1.09E-08	2.93E-08	yesL
AB5991_03885	4.48	7.08	4.68	14.51	16.66	15.35	5.41	15.51	1.5183	5.63E-07	1.31E-06	yesM
AB5991_03895	6.19	6.2	4.37	4.59	7.31	4.72	5.59	5.54	-0.0121	1	1	yesO
AB5991_03900	2.14	1.43	2.29	3.38	2.37	2.1	1.95	2.62	0.4218	0.477769143	0.537608746	yesP
AB5991_03905	1.22	1.12	1.52	1.99	2.68	5.27	1.29	3.31	1.3646	0.01835679	0.026642842	yesQ

AB5991_03910	5.94	5.13	5.04	11.4	14.75	17.01	5.37	14.39	1.4217	1.39E-05	2.83E-05	yesR
AB5991_03915	5.85	7.69	5.75	15.74	20.6	22.25	6.43	19.53	1.6028	2.04E-08	5.38E-08	yesS
AB5991_03920	4.4	7.12	4.7	19.97	22.9	21.55	5.41	21.47	1.9897	2.89E-09	8.18E-09	rhgT
AB5991_03925	7.91	7.51	7.58	20.16	26.64	23.02	7.67	23.27	1.6020	4.67E-07	1.10E-06	yesU
AB5991_03930	4.32	6.88	4.62	10.97	15.84	12.48	5.27	13.10	1.3124	0.000441451	0.000778421	yesV
AB5991_03935	5.63	8.19	3.42	11.61	11.75	17.85	5.75	13.74	1.2572	0.000402421	0.000713399	yesW
AB5991_03940	3.34	4.69	3.26	12.61	12.1	12.45	3.76	12.39	1.7187	1.99E-08	5.25E-08	yesX
AB5991_03945	6.63	4.57	5.32	24.05	17.44	25.13	5.51	22.21	2.0117	1.08E-08	2.92E-08	yesY
AB5991_03960	3.11	2.86	1.67	8.47	10.24	10.76	2.55	9.82	1.9476	2.21E-08	5.82E-08	lipO
AB5991_03965	1.7	1.04	0.81	3.08	4.04	4.29	1.18	3.80	1.6844	0.000784959	0.001349383	lplB
AB5991_03970	2.65	2.62	3.48	0	1.86	3.31	2.92	1.72	-0.7591	0.299057889	0.352600617	lplC
AB5991_03975	21.11	29.14	22.15	6.58	5.47	6.26	24.13	6.10	-1.9834	3.92E-11	1.30E-10	lplD
AB5991_03980	30.64	42.92	26.94	3.39	1.85	4.78	33.50	3.34	-3.3262	4.81E-17	2.54E-16	yetF
AB5991_03985	15.48	27.4	11.23	53.5	71.45	54.45	18.04	59.80	1.7292	1.31E-06	2.95E-06	hmoA
AB5991_03990	0	0	0.53	3.25	5.07	4.31	0.18	4.21	4.5747	4.12E-05	8.06E-05	yetH
AB5991_03995	423.98	245.84	426.13	51.66	79.16	120.09	365.32	83.64	-2.1269	1.74E-09	4.99E-09	--
AB5991_04000	1695.9	1778.06	1565.61	18.46	20.86	17.03	1679.86	18.78	-6.4827	3.74E-86	7.43E-83	yezB
AB5991_04005	0	0	1.15	0	0	0	0.38	0.00	-8.5825	1	1	yezD
AB5991_04010	104.81	183.18	100.09	20.72	11.41	12.13	129.36	14.75	-3.1323	4.88E-17	2.57E-16	yetJ
AB5991_04020	23.56	39.43	23.62	0.8	1.25	0.8	28.87	0.95	-4.9255	7.85E-32	1.20E-30	--
AB5991_04025	59.31	62.66	52.41	44.95	47.02	43.51	58.13	45.16	-0.3642	0.155156099	0.194545269	NGR_a01970
AB5991_04030	32.99	32.14	27.54	2.2	1.71	1.28	30.89	1.73	-4.1583	2.50E-29	3.23E-28	yetN
AB5991_04035	37.16	54.38	48.35	63.12	70.27	80.94	46.63	71.44	0.6155	0.022155581	0.031738749	cypD
AB5991_04040	155.33	220.91	147.59	55.29	55.67	42.09	174.61	51.02	-1.7751	6.05E-10	1.80E-09	ltaS1
AB5991_04045	1472.46	1518.02	1401.06	101.76	162.56	271.11	1463.85	178.48	-3.0360	1.81E-17	9.84E-17	yfnH
AB5991_04050	1646.51	1694.39	1625.94	135.72	187.76	298.24	1655.61	207.24	-2.9980	1.14E-19	7.53E-19	yfnG

AB5991_04055	1553.41	1474.22	1411.13	144.85	219.66	338.55	1479.59	234.35	-2.6584	1.53E-15	7.14E-15	yfnF
AB5991_04060	2760.14	2740.13	2595.28	213.09	335	509.65	2698.52	352.58	-2.9361	7.83E-19	4.77E-18	yfnE
AB5991_04065	2711.24	2614.7	2564.03	192.38	306.21	502.53	2629.99	333.71	-2.9784	9.13E-18	5.15E-17	yfnD
AB5991_04070	9.7	11.33	10.69	13.11	14.51	10.34	10.57	12.65	0.2591	0.388325708	0.445676702	yfnC
AB5991_04075	7.66	19.22	12.56	8.89	3.12	2.21	13.15	4.74	-1.4717	0.006143068	0.009455086	yfnB
AB5991_04080	104.85	106.56	114.5	5.25	4.25	7.62	108.64	5.71	-4.2507	1.02E-39	2.79E-38	mtrA
AB5991_04085	1.86	1.82	1.99	2.16	2.9	1.88	1.89	2.31	0.2916	0.521727024	0.578063062	yfmT
AB5991_04090	7.56	7.32	3.59	3.88	3.2	2.95	6.16	3.34	-0.8809	0.044840572	0.061084704	yfmS
AB5991_04095	22.28	37.75	23.62	49.72	47.01	34.16	27.88	43.63	0.6459	0.030656771	0.042910834	yfmR
AB5991_04100	6.07	15.59	10.81	27.27	28.81	19.26	10.82	25.11	1.2143	0.001336225	0.002245514	yfmQ
AB5991_04105	7.26	21.97	9.14	19.98	29.14	13.88	12.79	21.00	0.7154	0.111009096	0.143869817	yfmP
AB5991_04110	1.97	2.79	1.79	2.81	3.4	2.3	2.18	2.84	0.3777	0.373298137	0.431045916	yfmO
AB5991_04125	0.45	0.62	0.61	1.39	0.97	0.74	0.56	1.03	0.8838	0.222607106	0.270825006	insK
AB5991_04130	33.67	44.12	38.11	17.55	16.07	12.19	38.63	15.27	-1.3391	2.31E-06	5.08E-06	yfmM
AB5991_04135	5.11	10.27	8.03	9.39	5.86	5.19	7.80	6.81	-0.1957	0.648235701	0.703125913	yfmL
AB5991_04145	60.68	75.42	59.99	215.26	291.45	248.33	65.36	251.68	1.9450	1.19E-12	4.48E-12	pel
AB5991_04150	1.26	3	2.56	7.11	8.19	9.67	2.27	8.32	1.8724	7.67E-07	1.77E-06	yflS
AB5991_04155	4.77	6.32	3.32	10.02	11.29	9.61	4.80	10.31	1.1015	0.000617358	0.00107382	citS
AB5991_04160	4.78	9.75	5.11	17.9	12.97	14.08	6.55	14.98	1.1945	0.00127033	0.00214385	citT
AB5991_04165	13.18	15.21	8.46	28.87	36.41	31.59	12.28	32.29	1.3944	5.11E-06	1.09E-05	yflP
AB5991_04170	0	0	0	0	0	0	0.00	0.00	0.0000	1	1	--
AB5991_04175	4.16	5.35	2.82	27.93	15.4	11.42	4.11	18.25	2.1507	1.90E-07	4.61E-07	citM
AB5991_04180	19.1	28.39	18.48	38.82	20.83	16.98	21.99	25.54	0.2161	0.551515044	0.608633468	yflN
AB5991_04185	2.15	3.34	2.3	16.2	15.67	6.99	2.60	12.95	2.3186	9.13E-08	2.27E-07	nos
AB5991_04190	2.62	8.42	9.1	0.71	2.67	1.42	6.71	1.60	-2.0690	0.007176592	0.01095665	acyP
AB5991_04195	8.96	7.48	10.45	48.11	43.64	37.9	8.96	43.22	2.2695	4.73E-13	1.85E-12	yflK

AB5991_04200	2.62	2.41	4.2	1.42	0	1.42	3.08	0.95	-1.7004	0.316285201	0.371274817	yflJ
AB5991_04210	65.41	35.82	52.16	48.05	43.22	29.82	51.13	40.36	-0.3411	0.305444528	0.359810211	yflH
AB5991_04215	94.47	59.3	106.44	71.29	81.68	67.31	86.74	73.43	-0.2403	0.409548062	0.467063571	mapB
AB5991_04220	23.14	20.76	18.92	41.95	30.86	28.08	20.94	33.63	0.6835	0.013808028	0.020338159	nagP
AB5991_04225	56.54	47.32	68.3	96.78	93.49	70.94	57.39	87.07	0.6015	0.027317057	0.038466679	ltaS2
AB5991_04230	0	0	0	0	0	0	0.00	0.00	0.0000	1	1	yflD
AB5991_04235	19.78	44.76	19.67	10	14.51	4.48	28.07	9.66	-1.5384	0.001467312	0.002446136	yflB
AB5991_04240	17.1	32.35	15.7	308.89	193.05	287.83	21.72	263.26	3.5996	1.96E-23	1.66E-22	yflA
AB5991_04255	22.26	57.78	22.98	50.78	50.51	72.28	34.34	57.86	0.7526	0.041350563	0.056739145	treP
AB5991_04260	23.59	31.69	26.83	59.35	66.67	66.38	27.37	64.13	1.2285	3.35E-06	7.27E-06	treA
AB5991_04265	18.4	19.44	19.68	44.69	50.8	40.11	19.17	45.20	1.2372	7.89E-06	1.66E-05	treR
AB5991_04270	45.59	58.8	49.92	61.69	89.22	78.15	51.44	76.35	0.5699	0.040510293	0.05562461	yfkO
AB5991_04275	17.17	13.46	13.3	202.27	307.39	321.6	14.64	277.09	4.2420	2.15E-40	6.14E-39	yfkN
AB5991_04280	117.37	221.9	151.95	1493.86	1492.23	1521.56	163.74	1502.55	3.1979	4.62E-25	4.48E-24	yfkM
AB5991_04285	0.45	0.62	0.61	1.39	0.97	0.74	0.56	1.03	0.8838	0.222913269	0.270880578	insK
AB5991_04290	24.59	20.9	25.64	0.83	1.39	1.15	23.71	1.12	-4.3996	9.79E-30	1.33E-28	yfkL
AB5991_04295	15.06	6.15	8.05	3.64	0.85	0	9.75	1.50	-2.7041	0.000289525	0.000518585	yfkK
AB5991_04300	14.97	50.73	20.52	342.66	346.44	347.7	28.74	345.60	3.5880	3.52E-19	2.21E-18	yfkJ
AB5991_04305	16.33	40.32	16.86	293.34	327.23	301.11	24.50	307.23	3.6483	8.00E-23	6.58E-22	yfkI
AB5991_04310	28.16	52.91	24.98	268.52	316.15	312.87	35.35	299.18	3.0812	2.54E-20	1.76E-19	yfkH
AB5991_04315	5.88	0	1.57	0	1.5	3.18	2.48	1.56	-0.6707	0.751565085	0.800768703	--
AB5991_04320	14.76	13.83	14.96	39.12	39.11	30.45	14.52	36.23	1.3193	8.65E-07	1.98E-06	yfkF
AB5991_04325	2.57	10.69	6.04	23.83	29.61	37.43	6.43	30.29	2.2352	6.93E-08	1.74E-07	chaA
AB5991_04330	11.14	20.04	22.37	38.57	49.06	60.54	17.85	49.39	1.4683	7.28E-06	1.54E-05	yfkD
AB5991_04335	4.29	3.94	5.04	20.29	22.48	16.25	4.42	19.67	2.1530	2.23E-10	6.91E-10	yfkC
AB5991_04340	5.16	5.03	5	21.2	21.48	13.6	5.06	18.76	1.8895	1.91E-08	5.06E-08	yfkA

AB5991_04345	12.63	26.77	18.71	7.4	2.97	2.1	19.37	4.16	-2.2203	0.000202593	0.000368359	yfjT
AB5991_04350	10.27	17.6	13.42	3.47	3.25	1.48	13.76	2.73	-2.3321	4.36E-08	1.12E-07	pdaA
AB5991_04355	14.49	35.46	14.82	32.19	22.22	22.95	21.59	25.79	0.2563	0.480135377	0.539822237	yfjR
AB5991_04360	33.14	35.61	32.01	23.34	17.44	18.34	33.59	19.71	-0.7692	0.004842721	0.007550233	yfjQ
AB5991_04365	3.56	7.3	3.13	29.81	19.38	21.29	4.66	23.49	2.3328	8.57E-10	2.52E-09	yfjP
AB5991_04370	13.16	17.53	14.35	31.71	25.08	25.42	15.01	27.40	0.8681	0.001300653	0.002191299	yfjO
AB5991_04375	5.71	25.62	12.89	4.14	1.94	2.06	14.74	2.71	-2.4416	0.000397331	0.000705316	yfzA
AB5991_04380	16.26	20.02	17	5.43	3.76	3.2	17.76	4.13	-2.1044	3.58E-10	1.09E-09	dus2
AB5991_04385	1.2	0.73	0	3.91	2.44	1.3	0.64	2.55	1.9869	0.022912968	0.032752843	yfjM
AB5991_04390	0.52	0	1.12	4.84	3.2	0.28	0.55	2.77	2.3429	0.024791624	0.035197547	yfjL
AB5991_04395	2336.03	2814.77	1817.39	2611.07	3194.68	3325.85	2322.73	3043.87	0.3901	0.138589407	0.175939429	acoA
AB5991_04400	2186.73	2134.55	1624.31	1556.41	1968.49	2158.74	1981.86	1894.55	-0.0650	0.80322999	0.848079311	acoB
AB5991_04405	4473.73	4470.4	3287.93	2806.33	3217.36	3700.4	4077.35	3241.36	-0.3310	0.198872135	0.244571461	acoC
AB5991_04410	4788.08	4484.98	3394.5	2690.75	3075.33	3514.94	4222.52	3093.67	-0.4488	0.082105081	0.108426763	acoL
AB5991_04415	115.23	125.78	91.97	172.47	228.08	234.51	110.99	211.69	0.9315	0.000648826	0.001126088	acoR
AB5991_04420	76.32	62.69	66.58	48.05	65.41	57.61	68.53	57.02	-0.2652	0.384044391	0.44103725	sspH
AB5991_04425	27.64	31.46	28.96	50.5	39.94	41.88	29.35	44.11	0.5875	0.043711606	0.059649068	yfjF
AB5991_04430	30.96	36.67	38.45	14.48	11.86	11.53	35.36	12.62	-1.4860	9.02E-07	2.06E-06	yfjE
AB5991_04435	38.22	31.52	42.95	9.51	11.87	7.36	37.56	9.58	-1.9712	1.83E-09	5.26E-09	yfjD
AB5991_04440	16.24	14.69	12.84	8.19	6.71	6.62	14.59	7.17	-1.0243	0.001326351	0.002230808	yfjC
AB5991_04445	1.62	5.15	3.79	5.46	3.91	4.16	3.52	4.51	0.3576	0.410309836	0.467797978	yfjB
AB5991_04450	1.72	4.21	3.07	1.87	2.34	3.11	3.00	2.44	-0.2981	0.857702401	0.899138394	yfjA
AB5991_04455	77.39	99.81	66.01	205.75	181.1	191.17	81.07	192.67	1.2489	3.76E-06	8.12E-06	glvA
AB5991_04460	17.25	26.46	18.45	15.93	25.97	21.23	20.72	21.04	0.0223	0.936921237	0.973446947	glvR
AB5991_04465	60.82	57.83	52.23	18	19.98	19.52	56.96	19.17	-1.5713	4.69E-09	1.31E-08	malP
AB5991_04470	13.94	13.51	14.58	3.03	6.54	4.28	14.01	4.62	-1.6015	5.73E-07	1.34E-06	yobO

AB5991_04475	1.26	1.54	1.8	13.24	11.75	12.39	1.53	12.46	3.0226	3.23E-18	1.89E-17	yfiB
AB5991_04480	11.95	10.06	7.35	75.6	78.25	75.46	9.79	76.44	2.9654	8.67E-24	7.61E-23	yfiC
AB5991_04485	83.9	79.49	115.98	1.94	2.27	3.86	93.12	2.69	-5.1135	1.16E-34	2.21E-33	catD
AB5991_04490	236.36	196.89	261.33	10.77	12.86	18.47	231.53	14.03	-4.0442	2.44E-36	5.26E-35	catE
AB5991_04495	0.38	0.7	1.23	1.66	0.39	0.83	0.77	0.96	0.3182	0.868956374	0.908776867	yfiF
AB5991_04500	5.61	6.41	2.53	5.56	7.49	6.21	4.85	6.42	0.4046	0.290331432	0.343228972	yfiG
AB5991_04505	9.59	13.74	5.75	9.81	11.91	12.25	9.69	11.32	0.2242	0.507941374	0.567539448	yfiH
AB5991_04515	1.95	2.76	2.25	14.38	13.3	8.62	2.32	12.10	2.3828	3.27E-10	9.99E-10	lnrJ
AB5991_04520	4.36	3	6.12	28.17	24.14	15.35	4.49	22.55	2.3275	4.81E-09	1.34E-08	lnrK
AB5991_04525	0	0.71	0	0.84	0.2	0	0.24	0.35	0.5507	0.765725205	0.814326403	lnrL
AB5991_04530	0.46	0	0.16	0.17	0.15	0.66	0.21	0.33	0.6605	0.779415746	0.826451249	lnrM
AB5991_04535	3.12	9.17	4.17	4.92	4.77	5.24	5.49	4.98	-0.1408	0.842386418	0.884717393	lnrN
AB5991_04540	4.94	12.7	12.32	4.3	2.68	1.07	9.99	2.68	-1.8960	0.000871431	0.001490931	padR
AB5991_04545	4	6.82	4.58	5.28	0.58	2.16	5.13	2.67	-0.9413	0.136488194	0.17388342	estB
AB5991_04550	20.42	22.25	22.72	70.94	94.26	58.39	21.80	74.53	1.7737	2.26E-09	6.43E-09	yfiQ
AB5991_04555	7.02	5.37	7.51	7	7.44	5.7	6.63	6.71	0.0173	1	1	yfiR
AB5991_04560	2.02	2.65	3.55	2.04	2.93	0.94	2.74	1.97	-0.4760	0.394512145	0.451732333	yfiS
AB5991_04565	15.48	12.36	12.96	22.7	30.49	23.68	13.60	25.62	0.9139	0.003415132	0.005435466	yfiT
AB5991_04575	10.48	12.37	18.81	8.14	4.95	5.27	13.89	6.12	-1.1821	0.004063156	0.006392549	yfiV
AB5991_04580	22.22	22.46	22.4	39.84	34.92	38.13	22.36	37.63	0.7510	0.002978625	0.004789553	mprF
AB5991_04585	18.11	20.36	17.79	29.15	25.21	23.41	18.75	25.92	0.4671	0.08594285	0.11300631	yfiY
AB5991_04590	3.25	2.65	2.12	10.01	9	9.96	2.67	9.66	1.8529	5.64E-07	1.32E-06	yfiZ
AB5991_04595	5.25	5.47	7.49	4	4.81	3.6	6.07	4.14	-0.5532	0.128603387	0.164789948	yfhA
AB5991_04600	49.18	59.45	39.45	94.28	94.69	93.39	49.36	94.12	0.9312	0.000581342	0.001013838	yfhB
AB5991_04605	172.41	228.64	171.16	213.38	273.89	256.87	190.74	248.05	0.3790	0.15160984	0.190339131	yfhC
AB5991_04610	877.39	992.21	824.52	41.98	29.7	60.13	898.04	43.94	-4.3533	6.53E-39	1.68E-37	yfhD

AB5991_04615	0	0	0	0	0	0	0.00	0.00	0.0000	1	1	yfhE
AB5991_04620	5.95	10.92	7.63	63.8	64.75	60.5	8.17	63.02	2.9479	1.70E-21	1.26E-20	yfhF
AB5991_04625	35.47	39.66	35.5	14.09	9.26	5.66	36.88	9.67	-1.9311	1.79E-08	4.74E-08	recX
AB5991_04630	30.41	31.61	36.82	12.48	9.34	3.73	32.95	8.52	-1.9518	1.42E-06	3.20E-06	yfhH
AB5991_04635	9.23	6.39	5.99	3.95	2.31	3.11	7.20	3.12	-1.2056	0.001327965	0.002232578	yfhI
AB5991_04640	8.27	13.02	8.84	2.57	2.4	1.28	10.04	2.08	-2.2693	0.002979148	0.004789553	sspK
AB5991_04645	10.04	27.04	11.45	46.6	40.88	14.49	16.18	33.99	1.0712	0.031568363	0.044109068	yfhJ
AB5991_04650	222.19	642.04	291.62	4138.42	4296.98	4270.24	385.28	4235.21	3.4584	2.26E-19	1.44E-18	yfhK
AB5991_04655	105.84	214.28	136.41	1260.95	1003.21	1078.78	152.18	1114.31	2.8723	6.13E-19	3.76E-18	yfhL
AB5991_04660	270.39	593.24	317.44	2259.65	1766.73	1984.34	393.69	2003.57	2.3474	7.97E-13	3.06E-12	yfhM
AB5991_04665	26.47	43.58	21.87	127.28	124.13	148.03	30.64	133.15	2.1195	9.41E-12	3.30E-11	csbB
AB5991_04670	6.29	9.11	7.77	7.83	8.18	9.31	7.72	8.44	0.1280	0.654940941	0.70981727	yfhO
AB5991_04675	31.59	42.5	39.48	12.59	17.95	20.28	37.86	16.94	-1.1601	6.87E-05	0.000130769	yfhP
AB5991_04680	28.82	26.01	26.29	6.2	9.45	7.76	27.04	7.80	-1.7929	1.46E-09	4.24E-09	mutY
AB5991_04685	4.02	2.95	6.01	2.62	0	0	4.33	0.87	-2.3087	0.028559372	0.040130668	yfhS
AB5991_04690	9.84	9.7	6.67	32.89	31.76	25.46	8.74	30.04	1.7816	2.54E-08	6.62E-08	fabL
AB5991_04695	7433.4	7430.44	9794.31	1093.91	1075.62	1419.5	8219.38	1196.34	-2.7804	1.81E-23	1.55E-22	sspE
AB5991_04700	9	10.17	12.59	6.78	7.05	4.5	10.59	6.11	-0.7930	0.101220577	0.131958933	ygaB
AB5991_04705	15.66	36.88	20.39	39.24	47.12	17.69	24.31	34.68	0.5127	0.216937907	0.264576298	ygaC
AB5991_04710	88.15	65.5	78.63	19.62	15.73	13.13	77.43	16.16	-2.2604	3.21E-15	1.48E-14	ygaD
AB5991_04715	14.3	15.63	16.2	7.59	7.97	8.47	15.38	8.01	-0.9409	0.001166709	0.00197738	ygaE
AB5991_04720	107.33	128.13	118.67	69.79	66.03	42.17	118.04	59.33	-0.9925	0.000406802	0.00071988	gsaB
AB5991_04725	4.58	19.61	8.56	16.59	15.91	7.43	10.92	13.31	0.2860	0.555901302	0.612509453	ygaF
AB5991_04730	241.41	719.1	274.49	223.06	184.8	149.2	411.67	185.69	-1.1486	0.0031363	0.005023899	perR
AB5991_04735	59.74	68.44	45.32	149.38	113.81	106.12	57.83	123.10	1.0899	0.000153917	0.000282573	ygzB
AB5991_04740	21.85	36.75	22.71	33.54	35.96	39.57	27.10	36.36	0.4237	0.140416391	0.177747366	ygxA

AB5991_04840	50.01	94.4	46.77	5.31	5.45	6.04	63.73	5.60	-3.5084	2.07E-20	1.45E-19	spo0M
AB5991_04845	5.32	0	14.21	3.85	0.9	0	6.51	1.58	-2.0397	0.092301724	0.121006981	ygzA
AB5991_04850	71.51	81.45	78.59	0	0	0	77.18	0.00	-16.2360	1.78E-39	4.84E-38	ygzC
AB5991_04855	16.43	20.59	17.85	32.05	32.65	23.15	18.29	29.28	0.6790	0.022346544	0.032000767	ygaJ
AB5991_04860	2.96	6.74	3.05	15.41	23.14	25.93	4.25	21.49	2.3384	3.41E-10	1.04E-09	thiC
AB5991_04870	0	0	0	0	0	0	0.00	0.00	0.0000	1	1	senN
AB5991_04875	3519.5	5362.13	4071.02	247.22	361.08	327.58	4317.55	311.96	-3.7908	7.64E-36	1.61E-34	katA
AB5991_04880	0	0.43	0.5	0	0.24	0	0.31	0.08	-1.9542	0.639204593	0.694848464	ssuB
AB5991_04885	0	0	0	0.39	0	0.2	0.00	0.20	7.6196	0.265002104	0.316457856	ssuA
AB5991_04890	0.65	1.2	0.7	0.47	0.22	0	0.85	0.23	-1.8858	0.122673554	0.15766775	ssuC
AB5991_04895	1.28	1.76	1.03	2.26	1.3	1.73	1.36	1.76	0.3782	0.492530953	0.552497293	ssuD
AB5991_04900	22.21	37.08	35.64	6.59	6.51	5.1	31.64	6.07	-2.3829	7.90E-11	2.54E-10	ygaN
AB5991_04905	8.03	25.81	8.59	0.73	2.73	6.52	14.14	3.33	-2.0880	0.004435083	0.006950164	rpsN2
AB5991_04910	3.43	11.2	4.49	39.4	35.71	33.44	6.37	36.18	2.5052	1.81E-10	5.64E-10	ygaO
AB5991_04915	0	0	1.92	2.93	7.32	6.81	0.64	5.69	3.1514	0.003183725	0.005089603	ygzD
AB5991_04925	2.03	0.53	1.55	9.45	6.19	3.14	1.37	6.26	2.1920	0.000399749	0.000708978	yhzB
AB5991_04930	10.28	11.43	10.99	24.04	15.37	9.1	10.90	16.17	0.5690	0.120105349	0.154649268	queG
AB5991_04935	18.54	33.33	21.27	3.99	3.14	2.3	24.38	3.14	-2.9553	2.46E-14	1.05E-13	yhbB
AB5991_04940	16.09	23.36	17.21	12.62	11.81	9.32	18.89	11.25	-0.7474	0.031644146	0.044183862	cspR
AB5991_04945	61.09	63.07	56.9	0.92	0.29	0.61	60.35	0.61	-6.6364	1.11E-47	5.30E-46	yhbD
AB5991_04950	74.17	79.95	77.16	2.2	4.64	3.56	77.09	3.47	-4.4750	1.93E-39	5.15E-38	yhbE
AB5991_04955	69.95	76.88	75.35	9.72	5.72	3.04	74.06	6.16	-3.5877	1.19E-23	1.02E-22	yhbF
AB5991_04960	136.61	233.69	143.95	15.34	14.75	19.61	171.42	16.57	-3.3712	1.75E-25	1.78E-24	prkA
AB5991_04965	1354.93	2157.99	1358.47	52.35	74.74	90.78	1623.80	72.62	-4.4828	2.56E-39	6.73E-38	yhbH
AB5991_04970	14.77	11.42	7.9	0	0.79	0.42	11.36	0.40	-4.8163	2.35E-12	8.67E-12	yhbI
AB5991_04975	30.94	21.43	32.8	16.53	19.61	17.92	28.39	18.02	-0.6558	0.028024987	0.039407656	yhbJ

AB5991_04980	45.44	34.66	45.94	20.16	23.01	27.29	42.01	23.49	-0.8390	0.002746323	0.00444222	yhcA
AB5991_04985	42.55	40.63	41.13	12.22	9.01	12.9	41.44	11.38	-1.8648	1.67E-09	4.82E-09	yhcB
AB5991_04990	19.28	9.74	15.98	2.1	3.43	4.17	15.00	3.23	-2.2139	3.38E-06	7.34E-06	yhcC
AB5991_04995	0	0	0	0	0	0	0.00	0.00	0.0000	1	1	yhcD
AB5991_05000	0.71	0	0.25	2.84	1.45	1.54	0.32	1.94	2.6024	0.004546218	0.007104696	yhcE
AB5991_05005	0	0	0	2.15	5.03	0.53	0.00	2.57	11.3276	0.001300634	0.002191299	yhcF
AB5991_05010	0.52	1.42	0.28	2.53	2.37	3.08	0.74	2.66	1.8458	0.004035151	0.006353522	yhcG
AB5991_05015	0.2	0.72	0.42	3.85	4.41	2.77	0.45	3.68	3.0411	8.03E-07	1.85E-06	yhcH
AB5991_05020	6.91	7.4	5.13	8.35	10.94	8.52	6.48	9.27	0.5166	0.125546253	0.161236796	yhcI
AB5991_05025	58.48	107.38	141.18	86.73	96.49	36.45	102.35	73.22	-0.4831	0.24580077	0.296049396	cspB
AB5991_05030	15.66	28.76	17.68	5.91	8.41	5.42	20.70	6.58	-1.6535	5.63E-06	1.20E-05	yhcJ
AB5991_05035	12.22	8.6	16.82	1.46	2.21	0.54	12.55	1.40	-3.1604	4.61E-12	1.66E-11	yhcK
AB5991_05040	16.88	12.4	13.47	41.52	20.09	14.76	14.25	25.46	0.8371	0.024834613	0.035245093	tcyP
AB5991_05045	8.37	17.41	7.64	13.35	12.15	4.59	11.14	10.03	-0.1514	0.753872541	0.802796423	yqcG
AB5991_05050	2.6	11.94	6.95	19.33	17.2	13.14	7.16	16.56	1.2087	0.008706621	0.013131026	--
AB5991_05055	34.25	47.17	54.95	0.67	0.31	0.33	45.46	0.44	-6.7018	3.64E-35	7.37E-34	--
AB5991_05060	2.99	1.37	2.8	0.81	0.76	0	2.39	0.52	-2.1892	0.007412881	0.011291351	--
AB5991_05065	11.19	7.9	11.05	4.68	5.26	3.73	10.05	4.56	-1.1407	0.040004735	0.05494943	--
AB5991_05070	9.74	14.59	13.16	2.51	0.78	0.28	12.50	1.19	-3.3925	5.68E-11	1.86E-10	int
AB5991_05080	6.25	7.77	7.97	4.38	7.18	7.42	7.33	6.33	-0.2124	0.611044586	0.666975825	--
AB5991_05085	17.47	14.38	19.97	0.66	1.84	0	17.27	0.83	-4.3735	1.51E-12	5.68E-12	yddA
AB5991_05090	11.09	41.6	28.35	5.77	4.42	3.13	27.01	4.44	-2.6050	2.05E-06	4.53E-06	--
AB5991_05095	3.58	9.86	3.83	0.65	3.04	1.29	5.76	1.66	-1.7941	0.022086073	0.031650595	--
AB5991_05105	0	0	0	0	0	0	0.00	0.00	0.0000	1	1	--
AB5991_05110	0	0.31	0	0	0	0.18	0.10	0.06	-0.7843	1	1	yddB
AB5991_05115	0	0	0	0.79	0	0	0.00	0.26	8.0407	1	1	yddC

AB5991_05120	0	1.26	0	0	0.35	0	0.42	0.12	-1.8480	0.64594931	0.70102889	yddD
AB5991_05130	0	0	2.36	0	0.56	0	0.79	0.19	-2.0753	0.422031378	0.479509757	yddF
AB5991_05135	1.56	2.3	0.95	0.16	0.08	0	1.60	0.08	-4.3249	6.91E-09	1.89E-08	yddG
AB5991_05140	2.74	2.35	2.73	0	0.19	0	2.61	0.06	-5.3631	2.24E-08	5.88E-08	yddH
AB5991_05145	10.7	4.58	8.01	0	0	0	7.76	0.00	-12.9225	1.13E-11	3.91E-11	yddI
AB5991_05150	20.71	46.67	17.11	58.36	73.78	56.05	28.16	62.73	1.1553	0.001896293	0.003115507	yddJ
AB5991_05155	3.21	2.94	3.18	0.25	0.23	0.5	3.11	0.33	-3.2510	1.79E-06	3.99E-06	--
AB5991_05160	40.53	40.18	34.61	8.21	4.86	5.5	38.44	6.19	-2.6346	1.32E-17	7.30E-17	rapI
AB5991_05165	143.95	148.5	168.65	98.51	109.47	121.25	153.70	109.74	-0.4860	0.0679196	0.090687385	yhcM
AB5991_05170	157.21	185.54	150.34	20.48	21.08	15.63	164.36	19.06	-3.1080	2.23E-24	2.05E-23	yhcN
AB5991_05180	181.89	127.8	166.1	268.73	324.87	336.97	158.60	310.19	0.9678	0.000321514	0.000574071	yhcR
AB5991_05185	168.94	127.31	153.47	360.56	492.71	469.65	149.91	440.97	1.5566	1.15E-08	3.09E-08	srtD
AB5991_05190	17.7	24.1	19.14	6.49	3.04	2.15	20.31	3.89	-2.3833	1.42E-09	4.10E-09	yhcT
AB5991_05195	0	0	0.49	0	0.93	0	0.16	0.31	0.9244	1	1	yhcU
AB5991_05200	1623.32	1503.31	1670.19	187.75	191.79	258.57	1598.94	212.70	-2.9102	2.41E-25	2.40E-24	yhcV
AB5991_05205	2.73	4.51	3.79	13.64	18.59	9.15	3.68	13.79	1.9075	8.82E-06	1.84E-05	yhcW
AB5991_05210	20.4	24.54	18.05	66.04	58.1	33.5	21.00	52.55	1.3234	3.53E-05	6.96E-05	yhcX
AB5991_05215	24.18	26	20.29	131.2	138.68	136.37	23.49	135.42	2.5273	8.62E-21	6.21E-20	yhxA
AB5991_05220	54.32	40.7	43.4	209.48	269.43	251.76	46.14	243.56	2.4002	1.41E-17	7.75E-17	glpP
AB5991_05225	60.47	44.25	61.39	90.07	76.93	75.89	55.37	80.96	0.5482	0.048124535	0.065244975	glpF
AB5991_05230	178.2	194.33	186.42	554.41	483.52	412.19	186.32	483.37	1.3754	1.14E-07	2.81E-07	glpK
AB5991_05235	132.42	169.92	131.99	622.39	586.4	490.44	144.78	566.41	1.9680	3.39E-13	1.33E-12	glpD
AB5991_05240	35.4	41.25	35.76	131.39	112.31	104.11	37.47	115.94	1.6295	5.31E-10	1.59E-09	pgcA
AB5991_05245	31.55	27.66	36.96	35.01	40.34	38.79	32.06	38.05	0.2471	0.363466256	0.42067167	yhcY
AB5991_05250	42.96	43.02	44.15	54.91	61.6	60.99	43.38	59.17	0.4479	0.082439098	0.1087955	yhcZ
AB5991_05255	51.99	48.68	39.4	78.26	85.85	70.44	46.69	78.18	0.7437	0.005391509	0.008346854	azr

AB5991_05260	241	279.99	195.68	12.94	23.47	24.16	238.89	20.19	-3.5646	3.22E-26	3.42E-25	yhdB
AB5991_05265	0	2.05	4.77	4.85	1.14	1.21	2.27	2.40	0.0782	1	1	yhdC
AB5991_05270	0.12	0	0.13	1.75	1.39	0.54	0.08	1.23	3.8797	0.000124875	0.00023172	lytF
AB5991_05275	350.84	598.31	394.48	83.8	56.31	47.92	447.88	62.68	-2.8371	9.78E-18	5.49E-17	nsrR
AB5991_05280	19.72	16.4	16.54	32.35	35.26	36.09	17.55	34.57	0.9776	0.000247011	0.000445855	ygxB
AB5991_05285	48.17	95.3	54.68	6.71	7.84	10.85	66.05	8.47	-2.9637	2.57E-16	1.28E-15	spoVR
AB5991_05290	397.36	458.09	375.43	16.45	8.49	11.72	410.29	12.22	-5.0693	1.38E-48	7.19E-47	phoA
AB5991_05295	7.91	17.17	9.62	22.1	26.18	11.49	11.57	19.92	0.7845	0.043875879	0.059852667	lytE
AB5991_05300	1.65	1.14	2.65	1.12	1.47	1.34	1.81	1.31	-0.4691	0.421699749	0.479343128	citR
AB5991_05305	13.46	7.23	7.02	5.71	9.86	9.6	9.24	8.39	-0.1387	0.717072652	0.768140141	citA
AB5991_05310	21.81	28.61	21.55	107.78	112.91	114.7	23.99	111.80	2.2204	4.28E-15	1.95E-14	yhdF
AB5991_05315	92.83	99.23	92.36	13.92	7.5	11.76	94.81	11.06	-3.0996	9.93E-24	8.65E-23	yhdG
AB5991_05320	2.4	1.22	2.14	7.54	8.82	5.48	1.92	7.28	1.9228	1.85E-06	4.11E-06	yhdH
AB5991_05325	30	43.08	33.45	16.45	15.92	16.93	35.51	16.43	-1.1116	4.94E-05	9.56E-05	yhdI
AB5991_05330	0.84	1.55	0	0.46	2.57	0	0.80	1.01	0.3423	1	1	yhdJ
AB5991_05335	14.29	22.81	18.6	30.4	28.45	31.6	18.57	30.15	0.6994	0.036376702	0.050314136	yhdK
AB5991_05340	38.26	80.43	38.05	69.36	66.27	62.86	52.25	66.16	0.3407	0.287543082	0.34043935	yhdL
AB5991_05345	7.35	20.91	11.39	1.6	3.37	1.19	13.22	2.05	-2.6863	3.20E-06	6.96E-06	sigM
AB5991_05350	12.7	54.65	16.69	166.38	120.42	156.37	28.01	147.72	2.3987	1.35E-07	3.30E-07	yhdN
AB5991_05355	3.01	6.64	3.87	45.87	38.32	23.48	4.51	35.89	2.9934	3.08E-13	1.21E-12	plsC
AB5991_05360	30.33	46.74	25.19	57.43	46.99	29.46	34.09	44.63	0.3887	0.245193771	0.295407908	yhdP
AB5991_05365	25.52	45.33	37.14	60.07	49.82	31.25	36.00	47.05	0.3862	0.250852442	0.301859105	cueR
AB5991_05375	117.89	199.47	113.38	204.52	179.84	129.45	143.58	171.27	0.2544	0.408547354	0.466056174	yhdT
AB5991_05380	25.82	23.24	23.82	50.66	32.98	31.79	24.29	38.48	0.6634	0.042680331	0.058382223	crcB1
AB5991_05385	12.78	14.25	13.67	19.86	17.65	17.29	13.57	18.27	0.4291	0.199224656	0.244853331	crcB2
AB5991_05390	24.69	22.67	29.84	23.9	18.6	17.11	25.73	19.87	-0.3730	0.209913985	0.256798655	yhdW

AB5991_05395	51.88	39.95	42.95	242.09	281.05	148.56	44.93	223.90	2.3172	1.74E-10	5.45E-10	yhdX
AB5991_05400	924.15	1020.06	906.19	41.22	43.85	52.77	950.13	45.95	-4.3701	4.42E-50	2.55E-48	yhdY
AB5991_05405	14.33	32.12	20.78	22.46	22.01	13.68	22.41	19.38	-0.2093	0.586567186	0.643619314	cobB
AB5991_05410	3.83	8.6	7.29	40.29	33.15	12.91	6.57	28.78	2.1305	2.72E-06	5.95E-06	yheN
AB5991_05415	230.78	431.57	186.24	1304.29	1343.61	1160.37	282.86	1269.42	2.1660	9.39E-11	3.00E-10	dat
AB5991_05420	10.48	14.13	11.64	41.86	22.96	26.15	12.08	30.32	1.3274	1.89E-05	3.81E-05	nhaC
AB5991_05425	458.91	894.98	439.83	5103.65	5267.25	5646.84	597.91	5339.25	3.1586	6.78E-22	5.24E-21	nhaX
AB5991_05430	2.23	2.05	2.39	3.64	0	1.21	2.22	1.62	-0.4597	1	1	yheJ
AB5991_05435	7.3	9.06	7.15	35.11	44.68	47.52	7.84	42.44	2.4370	1.18E-16	6.07E-16	yheI
AB5991_05440	34.68	32.01	31.36	53.96	63.14	70.54	32.68	62.55	0.9364	0.000294303	0.000526669	yheH
AB5991_05445	0.58	1.07	1.56	10.45	10.37	4.41	1.07	8.41	2.9745	6.68E-07	1.55E-06	yheG
AB5991_05450	1.43	0	1.53	0	0	0	0.99	0.00	-9.9464	0.509606204	0.568309168	yheF
AB5991_05455	147.97	94.36	135.5	16.38	9.02	8.63	125.94	11.34	-3.4729	2.03E-19	1.30E-18	sspB
AB5991_05460	1.46	2.49	2.28	9.07	7.18	5.16	2.08	7.14	1.7810	8.85E-06	1.84E-05	--
AB5991_05465	29.71	22.73	31.77	7.18	8.4	3.57	28.07	6.38	-2.1366	1.22E-06	2.75E-06	yheE
AB5991_05470	141.73	203.72	181.51	29.44	28.09	33.18	175.65	30.24	-2.5384	1.70E-19	1.10E-18	yheD
AB5991_05475	90.87	153.79	132.05	16.74	20.89	16.84	125.57	18.16	-2.7899	1.17E-19	7.70E-19	yheC
AB5991_05480	15.78	16.97	15.17	7.28	11.84	14.49	15.97	11.20	-0.5117	0.118574445	0.152876339	yheB
AB5991_05485	78.63	86.25	56.79	122.72	120.56	93.41	73.89	112.23	0.6030	0.032186707	0.044875632	yheA
AB5991_05495	802.84	999.5	785.88	24.03	26.73	23.92	862.74	24.89	-5.1151	7.78E-61	9.36E-59	yhaX
AB5991_05500	81.25	153.16	88.95	9.01	10.02	8.96	107.79	9.33	-3.5302	1.27E-25	1.30E-24	hemZ
AB5991_05505	30.57	28.61	32.22	6.94	9.52	9	30.47	8.49	-1.8440	9.58E-11	3.06E-10	khtU
AB5991_05510	28.31	23.99	29.89	10.26	11.45	15.72	27.40	12.48	-1.1348	0.000389921	0.000692785	khtT
AB5991_05515	79.01	57.4	54.71	63.67	60.16	61.53	63.71	61.79	-0.0441	0.886339481	0.925494105	khtS
AB5991_05520	80.02	128.78	60.66	363.47	392.11	374.51	89.82	376.70	2.0683	3.49E-11	1.16E-10	yhaR
AB5991_05525	9.72	14.27	4.16	31.71	19.78	12.62	9.38	21.37	1.1874	0.029461705	0.041325479	yhzD

AB5991_05530	3.02	2.22	3.23	21.7	23.79	17.23	2.82	20.91	2.8885	1.75E-15	8.14E-15	yhaQ
AB5991_05535	6.74	11.33	8.9	51.17	46.87	46.28	8.99	48.11	2.4198	3.09E-16	1.53E-15	yhaP
AB5991_05540	0.15	0	0.47	3.52	4.05	1.59	0.21	3.05	3.8850	2.63E-07	6.33E-07	yhaO
AB5991_05545	14.44	18.59	10.76	14.68	16.67	17.79	14.60	16.38	0.1663	0.561137894	0.618107788	yhaN
AB5991_05550	43.04	61.46	36.41	47.22	40.69	27.33	46.97	38.41	-0.2901	0.360059323	0.416971587	yhaM
AB5991_05555	10.18	18.7	8.17	0.92	0.86	2.76	12.35	1.51	-3.0287	2.83E-05	5.64E-05	yhaL
AB5991_05560	16.13	32.63	20.75	210.35	187.7	82.24	23.17	160.10	2.7886	1.72E-12	6.41E-12	prsA
AB5991_05565	430.06	179.3	435.47	29.37	42.29	33.73	348.28	35.13	-3.3095	4.33E-15	1.96E-14	--
AB5991_05570	2.08	0	6.67	9.04	4.23	0	2.92	4.42	0.6008	0.78409284	0.830966819	sscB
AB5991_05575	0	0	1.06	1.07	1.01	0	0.35	0.69	0.9725	1	1	--
AB5991_05580	1.04	3.2	2.98	4.55	1.77	1.88	2.41	2.73	0.1836	0.903123309	0.941286788	yhaJ
AB5991_05585	34.88	27.17	35.04	5.75	9.68	2.86	32.36	6.10	-2.4083	4.79E-09	1.33E-08	yhaI
AB5991_05590	1109.6	1738.63	1269.67	77.09	72.44	57.87	1372.63	69.13	-4.3114	1.10E-41	3.28E-40	hpr
AB5991_05595	62.78	119	44.94	93.61	78.84	51.52	75.57	74.66	-0.0176	0.991919224	1	yhaH
AB5991_05600	137.44	124.46	111.75	10.24	1.92	5.1	124.55	5.75	-4.4362	2.69E-26	2.90E-25	yhzF
AB5991_05605	26.47	51.8	37.99	50.38	22.33	19.23	38.75	30.65	-0.3386	0.414287847	0.471926862	trpP
AB5991_05610	3.68	9.83	6.09	24.75	14.31	15.22	6.53	18.09	1.4696	0.000179989	0.000328312	serC
AB5991_05615	11.97	21.97	18.09	35.46	37.8	23.23	17.34	32.16	0.8910	0.009920985	0.014844096	hit
AB5991_05620	28.18	44.61	31.44	8.98	10.38	2.63	34.74	7.33	-2.2449	1.16E-07	2.87E-07	ecsA
AB5991_05625	120.65	76.55	105.86	35.89	33.73	27.91	101.02	32.51	-1.6357	8.09E-09	2.21E-08	ecsB
AB5991_05630	131.94	116.23	155.23	51.7	42.95	38.25	134.47	44.30	-1.6019	6.90E-09	1.89E-08	ecsC
AB5991_05635	147.06	183.64	161	138.65	106.88	118.28	163.90	121.27	-0.4346	0.09491437	0.124227081	yhaA
AB5991_05640	29.09	15.26	36.1	13.13	6.61	9.84	26.82	9.86	-1.4435	8.88E-05	0.000167717	yhfA
AB5991_05650	42.93	70.22	55.94	199.33	190.57	153.87	56.36	181.26	1.6852	1.09E-08	2.93E-08	hmoB
AB5991_05655	51.49	39.15	54.79	9.35	12.95	11.04	48.48	11.11	-2.1250	1.38E-13	5.56E-13	pbpF
AB5991_05660	79.48	72.5	76.99	49.61	42.79	42.56	76.32	44.99	-0.7626	0.0028897	0.00466653	hemE

AB5991_05665	167.19	251.85	177.96	124.52	111.99	100.66	199.00	112.39	-0.8243	0.002570131	0.004172522	cpfC
AB5991_05670	137.26	171.7	129.41	142.18	137.74	126.43	146.12	135.45	-0.1094	0.675210111	0.728999279	cgoX
AB5991_05675	19.46	24.78	30.54	49.15	32.26	23.44	24.93	34.95	0.4876	0.160502835	0.200174861	yhgD
AB5991_05680	27.1	14.83	31.3	22.29	16.52	10.93	24.41	16.58	-0.5580	0.112948538	0.146192518	yhgE
AB5991_05685	71.71	48.19	64.83	40.4	90.29	41.21	61.58	57.30	-0.1038	0.76692676	0.815385854	fabHB
AB5991_05690	9.54	11.96	20.15	14.17	27.46	18.89	13.88	20.17	0.5391	0.1582514	0.197739556	yhfC
AB5991_05695	62491.2	50837.97	64507.77	502.71	772.24	980.34	59278.98	751.76	-6.3011	5.25E-71	1.74E-68	--
AB5991_05700	22.57	17.54	22.1	100.84	113.63	124.24	20.74	112.90	2.4448	3.53E-18	2.06E-17	yhfE
AB5991_05705	68.22	71.58	58.75	137.8	171.33	162.09	66.18	157.07	1.2469	5.36E-06	1.14E-05	yhfF
AB5991_05710	62.21	52.23	76.87	240.01	226.31	99.65	63.77	188.66	1.5648	5.71E-06	1.21E-05	gltT
AB5991_05715	1.34	0	0	21.84	28.62	14.49	0.45	21.65	5.5990	1.01E-08	2.74E-08	yhfH
AB5991_05720	0	0.9	0.79	2.67	1.75	0.53	0.56	1.65	1.5504	0.07618454	0.101045026	yhfI
AB5991_05725	33.39	35.32	32.22	7.5	6.46	10.22	33.64	8.06	-2.0615	5.15E-12	1.84E-11	lplJ
AB5991_05730	49.32	51.46	37.46	90.82	91.55	81.3	46.08	87.89	0.9316	0.000615628	0.001071278	yhfK
AB5991_05735	126.36	205.98	108.05	557.23	575.14	540.16	146.80	557.51	1.9252	1.40E-10	4.43E-10	lcfB
AB5991_05740	23.28	22.63	27.33	13.4	16.26	9.39	24.41	13.02	-0.9073	0.00805624	0.012196466	yhfM
AB5991_05745	52.63	88.35	49.04	16.11	13.21	11.91	63.34	13.74	-2.2044	1.85E-11	6.31E-11	yhfN
AB5991_05750	32022.1	49694.91	25336.67	354200.82	323064.24	328515.54	35684.56	335260.20	3.2319	4.12E-26	4.31E-25	aprN
AB5991_05755	2.81	4.43	3.44	1.31	0.41	1.74	3.56	1.15	-1.6261	0.021363968	0.030660035	yhfO
AB5991_05760	23.85	31.08	23.36	45.53	41.87	39.41	26.10	42.27	0.6958	0.009342759	0.014047746	yhfP
AB5991_05765	24.92	28.68	21.08	42.27	55.07	47.71	24.89	48.35	0.9578	0.000465717	0.000819753	yhfQ
AB5991_05770	16.46	25.09	16.61	42.9	37.93	30.26	19.39	37.03	0.9336	0.002574596	0.004178063	phoE
AB5991_05775	5.94	9.4	8.12	36.26	35.28	21.08	7.82	30.87	1.9811	3.30E-09	9.28E-09	yhfS
AB5991_05780	5.15	4.84	4.97	33.31	35.64	18.89	4.99	29.28	2.5538	6.92E-14	2.86E-13	yhfT
AB5991_05785	0.64	1.18	0.69	7.36	9.18	2.79	0.84	6.44	2.9451	1.82E-05	3.67E-05	bioY
AB5991_05790	6.54	10.48	10.56	1.21	1.84	1.36	9.19	1.47	-2.6448	1.22E-10	3.86E-10	hemAT

AB5991_05800	167.27	268.84	205.68	16.27	19.94	23.94	213.93	20.05	-3.4155	2.36E-27	2.73E-26	yhxC
AB5991_05805	455.74	612.72	505.54	246.99	208.33	205.7	524.67	220.34	-1.2517	3.00E-06	6.55E-06	yhzC
AB5991_05810	331.22	431.06	344.52	7.13	8.26	5.74	368.93	7.04	-5.7110	3.22E-61	4.13E-59	comK
AB5991_05815	18.48	25.81	24.7	87.59	96.48	77.61	23.00	87.23	1.9233	3.01E-11	1.01E-10	yhxD
AB5991_05820	80.33	92.19	55.84	233.71	219.39	212.33	76.12	221.81	1.5430	8.69E-08	2.17E-07	yhjA
AB5991_05825	27.05	16.48	36.42	36.24	34.54	29.02	26.65	33.27	0.3199	0.320213063	0.375203916	yhjB
AB5991_05830	12.59	3.3	19.23	13.69	17.39	7.79	11.71	12.96	0.1464	0.884980874	0.92431853	yhjC
AB5991_05835	11.66	18.74	28.06	0.53	0	0	19.49	0.18	-6.7853	1.54E-14	6.71E-14	yhjD
AB5991_05840	8.4	10.11	8.98	18.27	12.97	8.78	9.16	13.34	0.5418	0.144085671	0.181754828	yhjE
AB5991_05845	11.41	10.47	11.82	28.3	25.76	25.47	11.23	26.51	1.2388	6.08E-05	0.00011633	sipV
AB5991_05850	5.54	5.31	5.15	5.11	4.54	5.09	5.33	4.91	-0.1183	0.732071468	0.781886982	yhjG
AB5991_05855	2.74	5.03	3.29	4.1	1.05	2.22	3.69	2.46	-0.5856	0.435809125	0.493469642	yhjH
AB5991_05860	0	0	0.98	0	0.93	0	0.33	0.31	-0.0756	1	1	--
AB5991_05865	17.47	27.45	18.36	1.77	4.52	2.88	21.09	3.06	-2.7868	6.46E-13	2.50E-12	glcP
AB5991_05870	7.21	5.67	6.79	22.03	32.84	27.87	6.56	27.58	2.0726	6.04E-11	1.97E-10	ntdC
AB5991_05875	1.7	2.35	2.5	3.94	4.33	2.54	2.18	3.60	0.7228	0.140765255	0.178132195	ntdB
AB5991_05880	0.82	1.5	1.75	0.74	1.11	0.44	1.36	0.76	-0.8297	0.159282948	0.198778312	ntdA
AB5991_05885	10.77	17.77	17.38	33.16	25.09	11.27	15.31	23.17	0.5983	0.127075826	0.163043007	ntdR
AB5991_05890	39.75	20.98	42.51	42.21	64.98	32.53	34.41	46.57	0.4365	0.215692632	0.263381132	yhjN
AB5991_05895	1.2	1.1	1.44	0.16	0.15	0	1.25	0.10	-3.5927	0.000182464	0.000332369	yhjO
AB5991_05900	12.66	12.29	15.21	15.24	14.05	14.38	13.39	14.56	0.1209	0.67185026	0.72564515	yhjP
AB5991_05905	0	0	0.03	0.07	0.07	0	0.01	0.05	2.2224	1	1	--
AB5991_05910	0	0	0	0	0	0	0.00	0.00	0.0000	1	1	--
AB5991_05915	10.5	15.22	14.19	1.2	3.38	5.98	13.30	3.52	-1.9181	0.000182339	0.000332294	yhjQ
AB5991_05920	6.6	8.34	12.8	0	3.78	5.81	9.25	3.20	-1.5324	0.019677401	0.028403838	yhjR
AB5991_05925	4.65	3.89	3.48	48.46	50.13	44.88	4.01	47.82	3.5772	1.03E-34	1.97E-33	addB

AB5991_05930	14.41	10.5	9.09	86.41	103.09	104.84	11.33	98.11	3.1139	2.33E-25	2.34E-24	addA
AB5991_05935	2	3.1	0.82	19.22	15.96	17.64	1.97	17.61	3.1574	3.99E-16	1.96E-15	sbcD
AB5991_05940	3.68	4.21	2.68	26.88	39.9	31.89	3.52	32.89	3.2226	1.19E-23	1.03E-22	sbcC
AB5991_05945	4.18	5.48	2.55	25.95	43.11	38.1	4.07	35.72	3.1336	6.55E-12	2.32E-11	yisB
AB5991_05950	255.03	130.33	293.91	49.37	56.28	106.32	226.42	70.66	-1.6801	9.05E-06	1.88E-05	gerPF
AB5991_05955	243.25	118.89	274.55	33.74	45.3	79.82	212.23	52.95	-2.0028	3.86E-07	9.16E-07	gerPE
AB5991_05960	60.25	13.13	61.16	15.55	11.43	16.58	44.85	14.52	-1.6270	0.001862298	0.003062189	gerPD
AB5991_05965	139.51	81.09	175.47	31.81	46.44	63.32	132.02	47.19	-1.4842	3.15E-05	6.23E-05	gerPC
AB5991_05970	49.44	29.79	57.82	7.56	14.94	27.59	45.68	16.70	-1.4521	0.001194926	0.002021751	gerPB
AB5991_05975	23.61	7.48	29.6	7.08	5.8	2.64	20.23	5.17	-1.9673	0.000602462	0.001049749	gerPA
AB5991_05980	1.06	5.82	1.13	0	0	0	2.67	0.00	-11.3826	0.043578572	0.059508254	yisI
AB5991_05985	4.11	3.95	6.48	0.43	1	0	4.85	0.48	-3.3459	4.20E-08	1.08E-07	yisJ
AB5991_05990	2.59	5.86	5.55	72.25	60.3	31.75	4.67	54.77	3.5528	7.03E-18	3.98E-17	yisK
AB5991_05995	0.51	3.72	1.62	26.98	19.07	13.15	1.95	19.73	3.3391	4.82E-09	1.34E-08	yisL
AB5991_06000	20.6	31.77	15.98	511.7	671.5	579.04	22.78	587.41	4.6883	3.50E-41	1.04E-39	wprA
AB5991_06005	2.17	6.27	5.98	3.38	5.69	3.03	4.81	4.03	-0.2531	0.69999259	0.752890188	yisN
AB5991_06010	53	67.28	69.04	8.1	12.06	18.24	63.11	12.80	-2.3016	2.85E-12	1.04E-11	asnO
AB5991_06020	28.54	22.32	30.1	3.88	7.4	5.01	26.99	5.43	-2.3132	9.67E-13	3.69E-12	yisQ
AB5991_06025	48.74	55.7	44.74	17.75	22.78	23.1	49.73	21.21	-1.2293	5.81E-06	1.23E-05	yisR
AB5991_06030	159.18	209.8	159.17	52.73	54.97	49.4	176.05	52.37	-1.7493	6.71E-11	2.18E-10	degA
AB5991_06035	75.88	150.62	53.16	137.93	135.33	130.06	93.22	134.44	0.5283	0.126973566	0.162964457	iolX
AB5991_06040	17.72	15.62	17.06	10.02	6.49	8.06	16.80	8.19	-1.0365	0.002886205	0.004662783	yisT
AB5991_06045	11.72	8.01	13.99	1.48	0.55	1.48	11.24	1.17	-3.2641	5.92E-11	1.93E-10	yisU
AB5991_06050	12.17	13	11.29	5.27	5.18	6.72	12.15	5.72	-1.0864	0.000236228	0.000427362	yisV
AB5991_06055	6.51	7.79	10.89	5.23	5.18	3.37	8.40	4.59	-0.8703	0.03369138	0.046779186	yisX
AB5991_06060	4.26	5.76	4.31	1.71	2.74	1.7	4.78	2.05	-1.2204	0.007808253	0.011848136	yisY

AB5991_06070	10.04	18.44	11.17	13.54	8.18	6.52	13.22	9.41	-0.4896	0.283933998	0.336567733	yitI
AB5991_06075	22.61	26.17	21.13	24.91	19.81	21.6	23.30	22.11	-0.0761	0.786820219	0.833412401	yitJ
AB5991_06080	26.82	51.27	24.36	45.15	62.44	18.69	34.15	42.09	0.3017	0.480730868	0.540176083	yitK
AB5991_06090	4.35	5.7	4.65	3.38	5.06	8.07	4.90	5.50	0.1675	0.802995328	0.848057034	yitR
AB5991_06095	144.26	130.5	129.09	106.6	100.62	72.57	134.62	93.26	-0.5295	0.046559537	0.0632377	yitS
AB5991_06100	262.87	325.98	240.76	981.27	1047.23	1007.32	276.54	1011.94	1.8716	1.24E-12	4.69E-12	yitT
AB5991_06105	217.61	262.75	158.96	791.93	743.9	680.09	213.11	738.64	1.7933	2.73E-10	8.40E-10	ipi
AB5991_06110	123.24	90.52	100.55	29.78	17.65	16.8	104.77	21.41	-2.2909	2.41E-11	8.14E-11	yizC
AB5991_06115	301.25	216.92	288.05	23.13	15.63	21.74	268.74	20.17	-3.7362	1.62E-27	1.89E-26	--
AB5991_06120	1.11	6.12	3.33	12.57	9.5	3.85	3.52	8.64	1.2955	0.023298205	0.033267591	yitU
AB5991_06125	84.49	162.92	74.75	6.66	7.43	9.68	107.39	7.92	-3.7606	5.75E-22	4.50E-21	yitV
AB5991_06130	31.59	61.22	23.15	38.17	40.48	22.8	38.65	33.82	-0.1929	0.664891227	0.719815447	yitW
AB5991_06135	1.06	0	1.13	1.15	2.15	0	0.73	1.10	0.5915	1	1	--
AB5991_06140	7.52	8.42	7.76	1.25	2.2	2.48	7.90	1.98	-1.9988	4.90E-08	1.25E-07	yitY
AB5991_06150	4.35	7.35	3.54	44.13	12.94	5.28	5.08	20.78	2.0325	0.001005038	0.001713613	argC
AB5991_06155	10.36	10.87	9.97	68.42	16.87	8.17	10.40	31.15	1.5828	0.005887981	0.009080067	argJ
AB5991_06160	3.26	3.42	3.73	64.77	11.36	8.06	3.47	28.06	3.0157	9.74E-06	2.01E-05	argB
AB5991_06165	4.99	4.87	5.84	98.46	15.25	10.31	5.23	41.34	2.9817	9.92E-06	2.05E-05	argD
AB5991_06170	0.68	0.94	1.46	152.71	22	15.29	1.03	63.33	5.9469	1.29E-12	4.85E-12	carA
AB5991_06175	5.49	6.87	4.75	162.26	26.17	22.27	5.70	70.23	3.6223	1.17E-08	3.14E-08	carB
AB5991_06180	11.49	11.06	6.85	158.7	17.44	16.71	9.80	64.28	2.7136	0.000113815	0.000212087	argF
AB5991_06185	0.45	0.62	0.61	1.39	0.97	0.74	0.56	1.03	0.8838	0.223195532	0.270880578	insK
AB5991_06190	3.01	7.38	1.07	0	2.04	0	3.82	0.68	-2.4900	0.106349983	0.138192337	yjzC
AB5991_06195	9.72	3.57	5.2	6.34	1.98	0	6.16	2.77	-1.1521	0.149537071	0.188093668	yjzD
AB5991_06200	24670.91	34035.22	24808.14	541.6	783.14	1127.12	27838.09	817.29	-5.0901	2.40E-47	1.08E-45	yjaU
AB5991_06205	235.82	261.71	215.12	4.23	8.9	10.87	237.55	8.00	-4.8921	2.07E-42	6.63E-41	yjaV

AB5991_06210	429.9	518.36	367.34	22.67	30.47	31.79	438.53	28.31	-3.9533	7.12E-37	1.60E-35	med
AB5991_06215	62.13	67.42	79.53	10.24	5.75	4.08	69.69	6.69	-3.3809	4.03E-16	1.97E-15	comZ
AB5991_06220	209.29	71.33	116.99	16.38	29.05	42.05	132.54	29.16	-2.1843	6.85E-07	1.58E-06	yjzB
AB5991_06225	149.18	192.28	152.33	80.6	99.13	68.97	164.60	82.90	-0.9895	0.000265667	0.000477144	fabHA
AB5991_06230	271.71	303.03	231.73	342.67	358.59	268.13	268.82	323.13	0.2655	0.31257409	0.367336996	fabF
AB5991_06235	36.56	24.91	29.02	2	4.68	4.73	30.16	3.80	-2.9875	1.86E-15	8.64E-15	yjaZ
AB5991_06240	165.73	211.51	150.6	46.21	21.81	26.56	175.95	31.53	-2.4805	6.95E-14	2.87E-13	appD
AB5991_06245	165.6	193.77	156.98	57.78	29.73	30.63	172.12	39.38	-2.1279	2.05E-11	6.97E-11	appF
AB5991_06250	274.34	290.61	268.74	254.28	334.67	348.04	277.90	312.33	0.1685	0.513789571	0.569904577	appA
AB5991_06255	121.07	91.44	111.38	42.79	49.13	56.58	107.96	49.50	-1.1250	3.03E-05	6.01E-05	appB
AB5991_06260	186.89	143.02	182.48	76.09	94.6	113.92	170.80	94.87	-0.8483	0.001859999	0.003059675	appC
AB5991_06265	45.61	76.25	58.27	15.66	6.84	7.28	60.04	9.93	-2.5966	3.09E-12	1.13E-11	yjbA
AB5991_06270	13.11	24.73	13.24	57.01	61.32	52.02	17.03	56.78	1.7377	2.48E-08	6.49E-08	trpS
AB5991_06275	123.26	127.04	111.04	491.33	574.56	513.88	120.45	526.59	2.1283	2.51E-16	1.25E-15	oppA
AB5991_06280	2.32	0.35	2.48	56.92	45.99	42.23	1.72	48.38	4.8167	7.16E-33	1.17E-31	oppB
AB5991_06285	6.89	4.7	4	75.81	89.37	66.93	5.20	77.37	3.8961	1.38E-30	1.98E-29	oppC
AB5991_06290	32.39	24.04	18.13	233.63	276.88	262.88	24.85	257.80	3.3747	7.98E-28	9.49E-27	oppD
AB5991_06295	98.64	83.51	63.8	656.98	747.86	757.28	81.98	720.71	3.1360	2.46E-27	2.84E-26	oppF
AB5991_06300	3.41	2.72	1.43	7.59	7.55	7.87	2.52	7.67	1.6058	3.04E-05	6.02E-05	yjbB
AB5991_06305	110.51	309.53	113.84	83.86	52.74	121.32	177.96	85.97	-1.0496	0.012509883	0.018508474	yjbC
AB5991_06310	149.71	378.82	154.24	417.98	286.62	323.63	227.59	342.74	0.5907	0.100257681	0.130904485	spx
AB5991_06315	537.85	460.2	509.86	7.18	7.28	6.55	502.64	7.00	-6.1653	2.83E-73	1.25E-70	yjbE
AB5991_06320	160.24	200.08	196.04	33.46	14.35	12.49	185.45	20.10	-3.2058	1.03E-16	5.32E-16	--
AB5991_06325	408.27	508.19	386	276.77	262.64	295.73	434.15	278.38	-0.6411	0.014265357	0.020965112	mecA
AB5991_06330	0	0	0	0	0	0	0.00	0.00	0.0000	1	1	--
AB5991_06335	2.51	8.07	3.86	18.09	8.62	15.63	4.81	14.11	1.5520	0.00037595	0.000669161	coiA

AB5991_06340	212.85	317.38	187.7	578.36	771.32	722.98	239.31	690.89	1.5296	1.71E-07	4.16E-07	yjbG
AB5991_06345	11.83	5.93	12.66	2.34	2.19	1.16	10.14	1.90	-2.4185	0.000729837	0.001259076	yizD
AB5991_06350	59.04	84.08	58.85	15.51	15.53	13.48	67.32	14.84	-2.1816	8.19E-14	3.36E-13	spxH
AB5991_06355	21.74	39.93	24.22	16.75	9.68	12.75	28.63	13.06	-1.1324	0.003144858	0.005035577	yjbI
AB5991_06360	40.82	38.83	48.39	12.04	12.26	5.32	42.68	9.87	-2.1120	7.62E-10	2.25E-09	yjbJ
AB5991_06365	26.5	28.96	32.72	5.49	1.93	0	29.39	2.47	-3.5710	3.86E-12	1.40E-11	yjbK
AB5991_06370	0	5.4	2.62	1.6	1.5	1.06	2.67	1.39	-0.9470	0.395948824	0.452985532	yjbL
AB5991_06375	24.44	35.49	21.27	18.24	15.62	11.08	27.07	14.98	-0.8535	0.013042204	0.019274505	yjbM
AB5991_06380	48.52	43.92	46.09	39.02	28.48	23.45	46.18	30.32	-0.6071	0.030423965	0.042615014	ppnKA
AB5991_06385	45.59	42.41	52.62	55.7	49.46	37.83	46.87	47.66	0.0241	0.945483525	0.981572569	yjbO
AB5991_06390	7.13	9.48	6.31	2.14	2.5	3.46	7.64	2.70	-1.5006	0.000406494	0.000719656	prpE
AB5991_06395	15.38	19.07	16.76	1.6	2.19	1.59	17.07	1.79	-3.2507	6.40E-22	4.97E-21	yjbQ
AB5991_06400	1.53	0.47	1.63	4.42	6.21	3.85	1.21	4.83	1.9960	0.000265516	0.000477107	tenA
AB5991_06405	6.73	11.28	7.19	9.86	17.86	16.46	8.40	14.73	0.8100	0.030136973	0.042227918	tenI
AB5991_06410	12.54	9.87	12.71	13.64	17.07	20.8	11.71	17.17	0.5526	0.071386238	0.095093844	thiO
AB5991_06415	2.7	4.95	3.85	1.96	5.49	7.79	3.83	5.08	0.4062	0.621435707	0.677387096	thiS
AB5991_06420	27.19	24.54	21.81	30.09	38.41	51.52	24.51	40.01	0.7067	0.018540139	0.026869669	thiG
AB5991_06425	41.12	30.53	32.12	32.47	44.94	58.45	34.59	45.29	0.3887	0.199302978	0.244873801	thiF
AB5991_06430	28.57	33.76	26.06	29.87	56.13	67.14	29.46	51.05	0.7929	0.015167971	0.022234039	thiD
AB5991_06435	4.19	11.11	7.71	127.76	157.92	91.16	7.67	125.61	4.0336	4.64E-25	4.48E-24	fabI
AB5991_06440	115.48	326.07	129.71	16.96	15.33	12.87	190.42	15.05	-3.6610	5.52E-18	3.16E-17	cotO
AB5991_06445	7905.97	2700.85	6444.84	736.09	1576.32	2570.99	5683.89	1627.80	-1.8040	5.12E-05	9.88E-05	cotZ
AB5991_06450	7851.68	2299.48	6788.21	741.7	1559.7	2460.37	5646.46	1587.26	-1.8308	7.28E-05	0.000138186	cotY
AB5991_06455	11712.4	3939.39	9444.58	1660.56	3975.87	5843.1	8365.46	3826.51	-1.1284	0.010365781	0.015451395	cotX
AB5991_06460	342.92	154.05	346.24	226.59	552.45	707.67	281.07	495.57	0.8182	0.044406009	0.060555035	--
AB5991_06465	19871.2	11499.52	18973.76	1137.83	2342.5	3276.7	16781.49	2252.34	-2.8974	5.40E-14	2.25E-13	--

AB5991_06470	27.85	40.91	28.15	6.61	1.03	1.1	32.30	2.91	-3.4709	2.80E-11	9.41E-11	yjcA
AB5991_06475	345.78	288.6	370.71	11.4	31.99	44.43	335.03	29.27	-3.5166	1.01E-19	6.69E-19	yjzK
AB5991_06480	9626.7	3568.93	9034.55	648.72	962.72	1176.58	7410.06	929.34	-2.9952	3.39E-14	1.43E-13	--
AB5991_06485	4216.06	2897.21	3777.19	402.41	628.35	913.85	3630.15	648.20	-2.4855	1.33E-13	5.36E-13	spoVIF
AB5991_06490	4.2	4.95	4.24	5.26	4.03	3.35	4.46	4.21	-0.0832	0.81994942	0.863207621	yjcD
AB5991_06495	87.18	93.71	67.46	175.77	174.58	186.45	82.78	178.93	1.1120	3.90E-05	7.65E-05	yjzE
AB5991_06500	228.61	305.21	194.66	379.68	355.31	291.87	242.83	342.29	0.4953	0.071632414	0.0953578	yjcF
AB5991_06505	360.45	471.46	412.43	378.3	408.56	301.45	414.78	362.77	-0.1933	0.468218955	0.527759714	yjcG
AB5991_06510	311.75	468.23	372.69	166.13	146.81	107.17	384.22	140.04	-1.4561	4.47E-07	1.05E-06	yjcH
AB5991_06515	3.22	8.28	4.13	11.91	10.33	7.85	5.21	10.03	0.9450	0.015032519	0.022051766	metI
AB5991_06520	12.64	14.15	10.38	32.01	38.74	30.86	12.39	33.87	1.4508	1.69E-07	4.12E-07	metC
AB5991_06525	36.41	21.88	41.42	5.04	4.04	2.87	33.24	3.98	-3.0607	1.82E-14	7.86E-14	yjcK
AB5991_06530	30.35	20.9	40.25	0.99	1.24	0.82	30.50	1.02	-4.9069	1.88E-29	2.48E-28	yjcL
AB5991_06550	87.26	105.86	121.09	19.77	19.56	17.99	104.74	19.11	-2.4546	8.86E-16	4.20E-15	--
AB5991_06555	114.61	116.35	111.83	28.24	25.37	26.24	114.26	26.62	-2.1020	6.79E-15	3.04E-14	--
AB5991_06560	0	1.78	0.52	1.59	0.99	0.53	0.77	1.04	0.4353	0.775187161	0.822626461	xre
AB5991_06565	18.16	22.73	19.42	0	0	0	20.10	0.00	-14.2951	2.20E-14	9.44E-14	--
AB5991_06580	4.86	8.92	8.31	0	0	0	7.36	0.00	-12.8461	2.67E-05	5.33E-05	--
AB5991_06590	44.49	39.43	53.2	1.68	3.46	1.67	45.71	2.27	-4.3316	2.65E-33	4.50E-32	--
AB5991_06600	112.53	144.94	97.58	77.81	62.79	40.14	118.35	60.25	-0.9741	0.001406073	0.002349964	gtfA
AB5991_06605	0.84	1.54	0	0	0	0	0.79	0.00	-9.6318	0.509838307	0.568309168	--
AB5991_06610	0	0	0.73	0	0	0	0.24	0.00	-7.9268	1	1	--
AB5991_06615	3.97	7.29	7.08	0	0	0	6.11	0.00	-12.5777	3.53E-06	7.64E-06	--
AB5991_06625	0	3.95	3.62	52.15	38.79	29.95	2.52	40.30	3.9973	1.87E-16	9.45E-16	--
AB5991_06630	4.74	68.37	20.27	384.33	288	322.44	31.13	331.59	3.4132	1.82E-08	4.81E-08	--
AB5991_06635	8.72	14.56	9.33	29.31	29.85	22.31	10.87	27.16	1.3210	0.000827445	0.001418732	--

AB5991_06640	7.09	8.68	5.05	19.27	12.02	19.18	6.94	16.82	1.2775	0.015521245	0.022709972	--
AB5991_06645	17.98	20.52	16.63	51.96	68.74	73.99	18.38	64.90	1.8203	4.93E-10	1.48E-09	rapK
AB5991_06650	9.87	11.4	6.97	2.1	3	1.76	9.41	2.29	-2.0415	1.63E-08	4.32E-08	yobL
AB5991_06655	28.35	48.45	45.48	6	6.01	0.43	40.76	4.15	-3.2971	1.70E-11	5.83E-11	yobK
AB5991_06660	59.31	62.66	52.41	44.95	47.02	43.51	58.13	45.16	-0.3642	0.155211833	0.194553721	NGR_a01970
AB5991_06665	75.02	121.48	85.91	161.89	153.3	160.49	94.14	158.56	0.7522	0.007695675	0.011690714	--
AB5991_06670	64.64	74.91	67.12	9.56	14.05	5.44	68.89	9.68	-2.8307	6.10E-15	2.75E-14	ywkF
AB5991_06680	4.52	4.15	5.64	0.82	0	0.82	4.77	0.55	-3.1253	0.003331123	0.005316675	--
AB5991_06685	4.5	3.1	4.22	0	0	1.22	3.94	0.41	-3.2763	0.001447537	0.002415197	--
AB5991_06690	1.04	0	1.04	0	0.23	0	0.69	0.08	-3.1769	0.139054385	0.176307472	--
AB5991_06695	41.99	59.78	45.23	5.3	7.74	9.88	49.00	7.64	-2.6811	6.48E-15	2.91E-14	--
AB5991_06700	24.1	42.78	22.34	1.75	0.82	0.87	29.74	1.15	-4.6969	4.87E-13	1.90E-12	--
AB5991_06710	818.98	723.6	609.1	2449.64	2329.79	2186.4	717.23	2321.94	1.6948	1.45E-10	4.56E-10	--
AB5991_06715	26.98	41.28	37.5	29.34	17.39	19.47	35.25	22.07	-0.6759	0.083041994	0.109481992	--
AB5991_06725	2.27	0	3.04	13.6	6.36	6.15	1.77	8.70	2.2978	0.000730001	0.001259076	yjcS
AB5991_06730	10.8	21.92	13.98	3.09	0.58	1.23	15.57	1.63	-3.2526	6.83E-08	1.72E-07	yjcN
AB5991_06735	1.58	3.41	0.79	1.11	0.57	0.1	1.93	0.59	-1.6992	0.018568813	0.026901407	manR
AB5991_06740	1.2	1.36	1.58	1.71	1.22	1.3	1.38	1.41	0.0310	1	1	manP
AB5991_06745	12.01	14	6.32	2.07	1.94	2.27	10.78	2.09	-2.3640	8.15E-08	2.04E-07	manA
AB5991_06750	30.86	43.17	23.18	8.39	6.36	7.56	32.40	7.44	-2.1234	9.17E-09	2.49E-08	yjdF
AB5991_06760	115.15	120.45	118.57	4.27	3.27	3.09	118.06	3.54	-5.0582	2.95E-47	1.31E-45	yjdG
AB5991_06765	82.62	98.06	100.06	2.48	6.5	3.46	93.58	4.15	-4.4962	1.23E-31	1.88E-30	yjdH
AB5991_06770	28.24	33.88	26.58	9.42	14.56	8.56	29.57	10.85	-1.4467	1.61E-05	3.26E-05	yjdI
AB5991_06775	16.68	15.32	20.82	3.02	11.32	5.02	17.61	6.45	-1.4480	0.005812531	0.008972615	yjzH
AB5991_06780	15.34	11.06	17.57	0.6	2.79	3.56	14.66	2.32	-2.6614	5.51E-07	1.29E-06	yjdJ
AB5991_06785	73.7	86.27	67.19	15.63	18.75	13.56	75.72	15.98	-2.2444	1.20E-14	5.28E-14	ctaB1

AB5991_06790	578.55	209.91	582.36	36.96	99.05	131.28	456.94	89.10	-2.3586	3.86E-07	9.15E-07	cotT
AB5991_06795	12.1	18.91	11.43	1.96	1.05	0.28	14.15	1.10	-3.6893	9.45E-16	4.46E-15	pdaC
AB5991_06800	1.53	1.4	2.04	2.49	0.39	2.06	1.66	1.65	-0.0087	1	1	yjfA
AB5991_06805	5.38	11.85	16.11	109.99	100.74	76.87	11.11	95.87	3.1087	2.54E-14	1.08E-13	yjfB
AB5991_06810	1	0.74	0.43	0	0	0	0.72	0.00	-9.4985	0.006515572	0.010005157	yjfC
AB5991_06815	8.15	7.49	5.81	12.32	9.22	3.43	7.15	8.32	0.2192	0.690757169	0.744368173	yjgA
AB5991_06820	7.53	21.32	11.07	72.35	93.58	73.37	13.31	79.77	2.5836	6.06E-13	2.35E-12	yjgB
AB5991_06830	29	40.82	32.39	56.07	46.89	54.06	34.07	52.34	0.6194	0.02496192	0.035388713	yjgD
AB5991_06835	2.24	2.57	3.3	1.83	0.57	0.3	2.70	0.90	-1.5867	0.018683069	0.027047199	yjhA
AB5991_06840	40.65	48.7	34.22	178.4	146.7	155.41	41.19	160.17	1.9592	2.50E-12	9.19E-12	yjhB
AB5991_06845	4.53	24.98	11.78	21.14	19.12	11.92	13.76	17.39	0.3377	0.501748686	0.561726538	yjiA
AB5991_06850	14.72	51.83	20.12	1043.64	1108.08	911.11	28.89	1020.94	5.1432	1.65E-29	2.18E-28	yjiB
AB5991_06855	29.59	76.57	30	1617.17	1557.18	1330.63	45.39	1501.66	5.0481	5.08E-34	9.21E-33	yjiC
AB5991_06860	3.83	5.27	5.11	0	1.95	1.04	4.74	1.00	-2.2487	0.035545693	0.049233327	yjzI
AB5991_06865	4.67	9.39	2.85	42.8	52.5	40.19	5.64	45.16	3.0022	1.92E-15	8.90E-15	yjjA
AB5991_06870	1.68	1.32	2.31	8.35	9.28	6.76	1.77	8.13	2.1995	7.05E-07	1.63E-06	yjkA
AB5991_06875	0.48	0.88	2.31	8.09	7.82	5.72	1.22	7.21	2.5592	1.03E-06	2.36E-06	yjkB
AB5991_06880	23.91	21.45	25.38	20.57	11.7	11.04	23.58	14.44	-0.7078	0.026289974	0.037138914	yjlA
AB5991_06890	180.75	156.92	178.67	62.27	69.15	45.33	172.11	58.92	-1.5466	7.28E-08	1.83E-07	yjlC
AB5991_06895	355.37	329.92	377.08	268.94	350.75	319.46	354.12	313.05	-0.1779	0.486720755	0.546539119	yjlD
AB5991_06900	51.73	139.1	46.08	34.15	37.26	38.25	78.97	36.55	-1.1113	0.004005519	0.006309368	uxaC
AB5991_06905	23.84	46.66	22.97	4.13	2.53	1.28	31.16	2.65	-3.5573	5.39E-16	2.62E-15	yjmB
AB5991_06910	20.86	43.86	19.44	5.62	5.99	4.82	28.05	5.48	-2.3568	1.85E-09	5.30E-09	yjmC
AB5991_06915	27.64	60.52	21.22	8.48	14.97	14.39	36.46	12.61	-1.5314	0.000170639	0.000311687	yjmD
AB5991_06920	40.33	107.56	38.66	20.75	28.28	33.52	62.18	27.52	-1.1762	0.003408145	0.005428697	uxuA
AB5991_06925	33.9	78.12	37.41	24.19	29.45	33.66	49.81	29.10	-0.7754	0.026752389	0.037725048	uxuB

AB5991_06930	39.6	59.63	35.64	16.27	23.48	27.75	44.96	22.50	-0.9986	0.001575214	0.002611765	exuT
AB5991_06935	89.47	116.59	84.69	46.89	48.1	41.98	96.92	45.66	-1.0859	5.49E-05	0.000105643	exuR
AB5991_06940	24.8	22.08	28.67	7.9	8.54	5.42	25.18	7.29	-1.7891	2.17E-09	6.18E-09	uxaB
AB5991_06945	34	38.65	29.76	11.32	12.31	12.18	34.14	11.94	-1.5159	2.39E-08	6.24E-08	uxaA
AB5991_06950	26.23	9.98	25.01	38.55	29.58	31.97	20.41	33.37	0.7094	0.045268111	0.061624844	yjnA
AB5991_06955	77.35	112.77	65.26	328.06	350.51	302.12	85.13	326.90	1.9412	2.08E-11	7.07E-11	yjoA
AB5991_06965	0.45	0.62	0.61	1.39	0.97	0.74	0.56	1.03	0.8838	0.22305204	0.270880578	insK
AB5991_06975	6991.83	6367.5	5074.6	77271.15	77789.7	86615.67	6144.64	80558.84	3.7126	2.47E-42	7.85E-41	rapA
AB5991_06980	409.7	324.51	376.56	4722.31	5464.26	4190.05	370.26	4792.21	3.6941	7.00E-37	1.59E-35	phrA
AB5991_06990	23.11	38.27	26.14	3.71	2.51	3.08	29.17	3.10	-3.2343	5.84E-18	3.33E-17	xlyB
AB5991_06995	11.48	14.05	12.78	87.37	92.47	88	12.77	89.28	2.8056	1.25E-19	8.22E-19	yjqA
AB5991_07000	2.7	3.85	1.28	6.19	5.49	0.65	2.61	4.11	0.6551	0.341452252	0.39797678	yjqB
AB5991_07005	247.69	120.94	215.93	35.46	68.79	106.83	194.85	70.36	-1.4696	0.000158954	0.000291281	yjqC
AB5991_07010	3.33	1.11	4.53	1.65	0.92	2.95	2.99	1.84	-0.7004	0.308526697	0.362902699	xkdA
AB5991_07015	91.43	134.89	92.69	4.6	4.3	2.86	106.34	3.92	-4.7616	4.14E-32	6.38E-31	xre
AB5991_07020	0	0	0	0	0	0	0.00	0.00	0.0000	1	1	yjzJ
AB5991_07025	0.86	0	0.69	0.94	0.44	0.94	0.52	0.77	0.5819	0.826540896	0.869225079	xkdB
AB5991_07030	8.57	8.7	8.93	0.49	1.61	0.49	8.73	0.86	-3.3385	4.36E-12	1.58E-11	xkdC
AB5991_07035	1.08	0	2.3	0	0	0	1.13	0.00	-10.1378	0.26476157	0.316391271	ykzK
AB5991_07040	19.05	19.86	12.12	0	0	2.23	17.01	0.74	-4.5162	3.50E-12	1.27E-11	xkdD
AB5991_07045	11.35	9.62	14.94	0	0.89	0	11.97	0.30	-5.3344	6.50E-08	1.64E-07	xtrA
AB5991_07050	13.82	25.38	15.16	0	0	0.38	18.12	0.13	-7.1604	4.63E-20	3.13E-19	xpf
AB5991_07055	7.25	4.99	6.78	0.74	0.46	0.74	6.34	0.65	-3.2934	9.36E-10	2.74E-09	xtmA
AB5991_07060	5.97	5.61	6.68	0.91	0.57	0.6	6.09	0.69	-3.1340	1.04E-12	3.94E-12	xtmB
AB5991_07065	22.59	35.91	36.89	1.32	1.61	2.1	31.80	1.68	-4.2452	2.00E-29	2.61E-28	xkdE
AB5991_07070	11.57	12.03	5.14	1.42	2.89	2.13	9.58	2.15	-2.1579	8.14E-06	1.71E-05	xkdF

AB5991_07075	15.64	14.54	11.77	2.52	2.75	3.55	13.98	2.94	-2.2498	2.69E-10	8.27E-10	xkdG
AB5991_07080	11.77	6.91	6.04	1.02	0	0.51	8.24	0.51	-4.0141	2.07E-07	5.00E-07	ykzL
AB5991_07085	9.62	4.65	2.71	1.65	2.06	0.55	5.66	1.42	-1.9949	0.00730694	0.011138525	xkdH
AB5991_07090	10.04	6.15	7.16	3.64	1.51	2.42	7.78	2.52	-1.6251	0.000727009	0.001255743	xkdI
AB5991_07095	10.25	0.75	5.26	0.89	0.42	0.89	5.42	0.73	-2.8858	0.000740177	0.001275712	xkdJ
AB5991_07100	0	0	0.88	0.9	0	0	0.29	0.30	0.0324	1	1	ykzM
AB5991_07105	7.1	8.53	6.07	3.23	2.49	3.07	7.23	2.93	-1.3038	0.00015203	0.000279237	xkdK
AB5991_07110	8.14	9.72	13.5	2.66	2.9	0.44	10.45	2.00	-2.3859	7.25E-06	1.53E-05	xkdM
AB5991_07115	5.66	8.17	5.19	2.64	2.47	2.19	6.34	2.43	-1.3815	0.006474603	0.009949941	xkdN
AB5991_07120	0	0	0	0	2.61	0	0.00	0.87	9.7649	0.512628816	0.569081258	xkzB
AB5991_07125	5.35	5.44	4.83	6.84	7.46	8.02	5.21	7.44	0.5149	0.046738765	0.063452867	xkdO
AB5991_07130	3.83	3.52	2.05	1.79	4.46	2.08	3.13	2.78	-0.1743	0.767699546	0.815988998	xkdP
AB5991_07135	4.07	8.82	4.74	4.22	3.95	3.4	5.88	3.86	-0.6076	0.165425302	0.205732501	xkdQ
AB5991_07140	1.35	2.49	2.9	0	1.38	0	2.25	0.46	-2.2881	0.1387574	0.1760964	xkdR
AB5991_07145	1.7	2.34	0.91	1.38	0.86	0	1.65	0.75	-1.1439	0.356863094	0.413632033	xkdS
AB5991_07150	1.9	4.12	2.4	1.69	0.7	0.75	2.81	1.05	-1.4231	0.016971424	0.024731568	xkdT
AB5991_07155	2.81	2.29	3	1.7	1.27	1.35	2.70	1.44	-0.9069	0.136292221	0.173689476	xkdU
AB5991_07160	1.32	0	0	1.44	0.67	0	0.44	0.70	0.6767	1	1	xkzA
AB5991_07165	2.8	2.89	2.34	0.48	0.8	0.19	2.68	0.49	-2.4496	4.14E-07	9.78E-07	xkdV
AB5991_07170	1.1	2.01	1.17	0	0	0.59	1.43	0.20	-2.8588	0.144625176	0.182319547	xkdW
AB5991_07175	0	0	0	0	0	0	0.00	0.00	0.0000	1	1	xkdX
AB5991_07180	2.8	5.53	2.3	2.34	2.41	3.49	3.54	2.75	-0.3674	0.563575477	0.620104799	xepA
AB5991_07185	4.02	2.46	5.73	1.46	4.09	0.72	4.07	2.09	-0.9615	0.22819723	0.276524626	xhlA
AB5991_07190	4.11	5.03	4.39	0	3.48	2.96	4.51	2.15	-1.0710	0.169406082	0.210419629	xhlB
AB5991_07195	17.99	12.62	11.89	7.92	6.17	6.13	14.17	6.74	-1.0717	0.001185205	0.002007014	xlyA
AB5991_07200	416.51	562.77	249.32	5.7	1.33	5.67	409.53	4.23	-6.5960	3.30E-38	8.03E-37	--

AB5991_07205	897.4	1348.91	1022.99	18.39	22.59	19.45	1089.77	20.14	-5.7576	1.96E-57	1.73E-55	spoIISB
AB5991_07210	2299.41	4624.69	2941.33	79.74	83.98	90.37	3288.48	84.70	-5.2790	3.28E-51	2.03E-49	spoIISA
AB5991_07215	6.13	5.63	6.75	53.36	26.25	19.53	6.17	33.05	2.4212	2.32E-09	6.59E-09	pit
AB5991_07220	0.88	2.15	5.32	18.77	17.56	5.7	2.78	14.01	2.3316	0.000137675	0.000254519	ykaA
AB5991_07225	4.25	4.54	6.6	11.79	6.01	8.47	5.13	8.76	0.7714	0.03719932	0.05134463	steT
AB5991_07230	16.35	27.92	18.9	40.1	47.59	33.95	21.06	40.55	0.9453	0.00131871	0.002218897	mhqA
AB5991_07240	0.34	0.62	0.72	0.82	0.86	0.73	0.56	0.80	0.5206	0.359686241	0.416661045	ykcB
AB5991_07245	1.3	1.37	0.6	2.02	3.6	2.21	1.09	2.61	1.2597	0.01975784	0.028509587	ykcC
AB5991_07250	41.51	42.78	39.8	55.04	51.51	52.03	41.36	52.86	0.3538	0.162332945	0.202266748	htrA
AB5991_07255	7.72	8.51	6.37	11.76	8.76	6.45	7.53	8.99	0.2550	0.502722579	0.56249968	proG
AB5991_07260	10.95	14.48	9.14	76.96	65.33	69.49	11.52	70.59	2.6150	2.43E-18	1.43E-17	dppA
AB5991_07265	7.02	7.52	5.21	64.89	53.38	70.5	6.58	62.92	3.2567	3.38E-25	3.31E-24	dppB
AB5991_07270	31.34	26.88	23.08	70.22	72.78	72.74	27.10	71.91	1.4080	3.97E-07	9.40E-07	dppC
AB5991_07275	83.92	94.5	70.95	103.75	116.62	129.47	83.12	116.61	0.4884	0.060516606	0.081131479	dppD
AB5991_07280	173.41	162.73	122.65	219.69	292.33	316.97	152.93	276.33	0.8535	0.001944876	0.003192684	dppE
AB5991_07285	7.72	11.41	7.05	24.98	33.53	38.11	8.73	32.21	1.8839	4.40E-09	1.23E-08	ykfA
AB5991_07290	24.1	26.43	19.15	55.88	60.81	71.02	23.23	62.57	1.4297	5.35E-07	1.25E-06	ykfB
AB5991_07295	27.18	28.31	13.67	50.3	64.62	75.98	23.05	63.63	1.4648	9.56E-06	1.98E-05	ykfC
AB5991_07300	45.1	58.33	39.05	104.05	129.7	140.72	47.49	124.82	1.3941	7.24E-07	1.67E-06	ykfD
AB5991_07305	31.85	46.15	31.3	149.59	182.91	177.4	36.43	169.97	2.2219	4.32E-15	1.96E-14	pgl
AB5991_07315	3.13	2.56	3.35	2.65	2.48	3.02	3.01	2.72	-0.1495	0.792492275	0.838525666	ykhA
AB5991_07320	11.93	18.35	12.6	1.95	3.04	1.45	14.29	2.15	-2.7352	1.76E-12	6.56E-12	hmp
AB5991_07325	3.21	4.43	17.18	0.87	1.64	1.74	8.27	1.42	-2.5460	0.003620388	0.005743732	ykzH
AB5991_07330	17.04	22.22	17.16	0.81	0.5	1.34	18.81	0.88	-4.4121	1.16E-21	8.74E-21	ykjA
AB5991_07335	5.82	3.14	5.13	13.03	7.32	8.52	4.70	9.62	1.0349	0.023597371	0.033646378	ykkA
AB5991_07340	5.92	5.12	2.61	8.33	6.38	2.26	4.55	5.66	0.3141	0.618543699	0.674419832	ykkB

AB5991_07345	0	0.98	0.57	1.74	4.34	1.15	0.52	2.41	2.2217	0.06842276	0.091299322	gdnC
AB5991_07350	0.57	0	0.61	0	1.74	1.85	0.39	1.20	1.6052	0.328062548	0.383722055	gdnD
AB5991_07355	34.23	54.03	37.46	21.77	20.58	10.18	41.91	17.51	-1.2590	0.000209491	0.000380554	purU
AB5991_07360	26.17	30.53	24.82	22.38	22.62	17.82	27.17	20.94	-0.3759	0.169892598	0.210957945	proB
AB5991_07365	79.66	119.14	76.82	108.37	116.3	110.06	91.87	111.58	0.2803	0.300320693	0.353984408	proA
AB5991_07370	1.27	5.45	1.81	11.07	9.93	4.59	2.84	8.53	1.5850	0.005431585	0.008405622	ohrA
AB5991_07375	38.67	52.33	32.22	5.31	7.04	5.73	41.07	6.03	-2.7688	9.99E-14	4.06E-13	ohrR
AB5991_07380	208.02	420.72	209.28	2135.6	2124.7	2081.34	279.34	2113.88	2.9198	1.13E-18	6.83E-18	ohrB
AB5991_07390	27.25	28.89	31.19	1.25	1.56	1.25	29.11	1.35	-4.4269	4.52E-23	3.77E-22	guaD
AB5991_07395	91.36	77.86	94.66	54.71	52.88	50.43	87.96	52.67	-0.7398	0.00342141	0.005443276	metE
AB5991_07400	48955.53	42983.23	49780.2	19253.66	18190.66	20984.88	47239.65	19476.40	-1.2783	9.73E-08	2.42E-07	isp
AB5991_07405	4.55	12.34	9.27	29.93	33.31	19.94	8.72	27.73	1.6689	1.14E-05	2.33E-05	rsbRB
AB5991_07410	4.02	3.47	4.3	4.37	6.49	7.42	3.93	6.09	0.6327	0.137842176	0.17515881	ykoC
AB5991_07415	5.5	4.04	4.7	3.71	6.94	8.57	4.75	6.41	0.4327	0.252842986	0.304070108	ykoD
AB5991_07420	3.61	2.21	5.48	4.26	7.36	6.2	3.77	5.94	0.6572	0.190911501	0.235510894	ykoE
AB5991_07425	1.5	0	0.64	3.59	6.71	5.52	0.71	5.27	2.8861	2.03E-05	4.09E-05	ykoF
AB5991_07430	0.53	1.93	1.13	0.86	1.61	0.57	1.20	1.01	-0.2399	1	1	ykoG
AB5991_07435	3.71	5.84	4.25	5.04	7.14	3.3	4.60	5.16	0.1657	0.664783442	0.719815447	ykoH
AB5991_07440	36.1	37.53	33.21	3.75	4.86	4.31	35.61	4.31	-3.0478	1.63E-19	1.06E-18	ykoI
AB5991_07445	68.71	66.64	58.4	29.89	25.1	20.6	64.58	25.20	-1.3579	1.82E-06	4.03E-06	ykoJ
AB5991_07450	1.31	0	0	0	1.33	0	0.44	0.44	0.0219	1	1	ykzD
AB5991_07455	0	0	0	0	0	0	0.00	0.00	0.0000	1	1	--
AB5991_07460	20.26	20.56	19.67	9.28	6.92	7.79	20.16	8.00	-1.3343	2.47E-06	5.43E-06	mgtE
AB5991_07465	94.45	294.02	106.8	5.31	4.42	2.94	165.09	4.22	-5.2887	7.68E-23	6.33E-22	tnrA
AB5991_07470	1.16	2.13	1.24	0	0	0	1.51	0.00	-10.5603	0.262023071	0.3141587	ykzB
AB5991_07475	0	0	1.06	0	0	0	0.35	0.00	-8.4649	1	1	ykoL

AB5991_07480	42.76	87.08	49.47	145	136.88	138.44	59.77	140.11	1.2290	0.000108461	0.000202587	ykoM
AB5991_07485	5.96	3.85	5	0.35	0.33	1.4	4.94	0.69	-2.8319	1.07E-07	2.64E-07	ykoN
AB5991_07490	71.06	87.78	67.58	4.99	4.67	8.86	75.47	6.17	-3.6118	4.33E-25	4.22E-24	ykoP
AB5991_07495	102.94	126.14	103.66	3.39	2.04	2.89	110.91	2.77	-5.3217	9.47E-48	4.59E-46	ykoQ
AB5991_07500	25.38	18.6	20.87	1.04	0.87	1.39	21.62	1.10	-4.2966	6.61E-31	9.75E-30	ykoS
AB5991_07505	51.69	42.22	37.74	2.71	2.36	1.16	43.88	2.08	-4.4013	2.51E-32	3.95E-31	ykoT
AB5991_07510	31.21	30.73	33.79	30.19	34.67	38.79	31.91	34.55	0.1147	0.670731709	0.72475527	ligd
AB5991_07515	328.28	342.17	280.65	1433.4	1258.43	1191.32	317.03	1294.38	2.0296	1.99E-14	8.58E-14	ku
AB5991_07520	13.61	13.67	12.15	0.25	0.54	0.16	13.14	0.32	-5.3752	2.41E-36	5.23E-35	ykoW
AB5991_07525	26.05	25.91	23.8	0.59	2.76	1.47	25.25	1.61	-3.9743	1.09E-21	8.28E-21	ykoX
AB5991_07530	496.09	735.26	562.44	19.76	17.92	28.3	597.93	21.99	-4.7648	8.66E-45	3.24E-43	ykoY
AB5991_07535	173.1	209.41	151.87	2.34	3.65	3.88	178.13	3.29	-5.7587	6.37E-55	4.86E-53	sigI
AB5991_07540	30.28	39.97	24.96	2.06	3.53	2.73	31.74	2.77	-3.5165	3.11E-22	2.49E-21	rsgI
AB5991_07545	190.02	134.46	182.39	7.06	9.43	21.07	168.96	12.52	-3.7543	1.29E-21	9.70E-21	sspD
AB5991_07550	5.66	7.56	7.43	8.96	9.43	2.23	6.88	6.87	-0.0021	1	1	ykrK
AB5991_07555	72.54	85.1	66.16	27.83	15.59	17.45	74.60	20.29	-1.8784	1.12E-09	3.26E-09	htpX
AB5991_07560	15.53	11.31	20.33	13.69	12.81	9.57	15.72	12.02	-0.3871	0.210561118	0.257431712	ktrD
AB5991_07565	1.46	2.49	2.28	9.07	7.18	5.16	2.08	7.14	1.7810	8.86E-06	1.84E-05	--
AB5991_07570	376.56	255.3	317.2	5.04	5.9	13.8	316.35	8.25	-5.2616	2.90E-38	7.15E-37	ykzP
AB5991_07575	180.75	176.26	171.45	6.66	9.35	6.63	176.15	7.55	-4.5448	4.65E-33	7.72E-32	ykzE
AB5991_07580	58.13	54.18	71.99	4.61	3.6	3.83	61.43	4.01	-3.9361	2.20E-33	3.77E-32	ykrP
AB5991_07585	5.05	4.34	4.45	18	16.76	15.97	4.61	16.91	1.8740	2.61E-11	8.78E-11	kinE
AB5991_07590	6.53	17.33	10.87	66.32	68.71	70.72	11.58	68.58	2.5666	9.05E-14	3.70E-13	ogt
AB5991_07595	11.48	21.07	26.08	1.56	2.92	9.32	19.54	4.60	-2.0870	0.00197923	0.003247737	--
AB5991_07600	18.55	13.13	19.29	6.66	5.2	4.61	16.99	5.49	-1.6298	4.24E-07	1.00E-06	mtnA
AB5991_07605	10.75	10.28	13.76	3.29	0.92	0.82	11.60	1.68	-2.7900	6.39E-10	1.90E-09	mtnK

AB5991_07610	22.48	20	20.32	15.37	11.32	5.77	20.93	10.82	-0.9521	0.007442459	0.011327714	mtnU
AB5991_07615	16.46	12.2	14.37	18.56	16.14	13.08	14.34	15.93	0.1511	0.632790956	0.689197171	mtnE
AB5991_07620	8.9	3.27	6.67	36.64	24.01	16.06	6.28	25.57	2.0256	5.54E-07	1.30E-06	mtnW
AB5991_07625	8.42	8.91	6.55	57.47	38.19	33.72	7.96	43.13	2.4377	1.33E-12	5.00E-12	mtnX
AB5991_07630	15.78	5.79	9.2	83.31	64.53	49.07	10.26	65.64	2.6779	9.87E-13	3.76E-12	mtnB
AB5991_07635	22.89	19.16	18.72	209.39	181.56	130.08	20.26	173.68	3.0999	6.12E-22	4.77E-21	mtnD
AB5991_07640	44.48	118.9	53.59	53.28	36.68	43.28	72.32	44.41	-0.7035	0.072427582	0.09631866	ykvA
AB5991_07645	3.5	6.43	1.5	28.95	16.4	17.44	3.81	20.93	2.4577	2.83E-06	6.17E-06	spo0E
AB5991_07655	38.74	41.9	44.86	48.73	39.91	27.53	41.83	38.72	-0.1114	0.704121698	0.756102559	kinD
AB5991_07660	114.72	120.48	135.92	8.53	13.44	7.59	123.71	9.85	-3.6502	1.41E-29	1.88E-28	mhqR
AB5991_07665	5.54	5.93	5.43	10.8	11.28	9.75	5.63	10.61	0.9134	0.006884965	0.010531662	motB
AB5991_07670	0.89	0	2.39	3.64	1.59	1.93	1.09	2.39	1.1263	0.14575374	0.183683942	motA
AB5991_07675	534.5	678.32	622.87	58.6	60.18	59.72	611.90	59.50	-3.3623	6.47E-34	1.15E-32	clpE
AB5991_07680	20.26	19.07	18.89	1.13	0.35	0.75	19.41	0.74	-4.7064	7.83E-29	9.88E-28	ykvI
AB5991_07685	1.1	3.02	0.88	5.36	5.02	4.45	1.67	4.94	1.5685	0.002679829	0.004341738	queC
AB5991_07690	0	0	2.58	6.99	5.72	4.78	0.86	5.83	2.7611	0.000186052	0.000338594	queD
AB5991_07695	0.99	1.36	0.79	5.37	7.79	4.81	1.05	5.99	2.5168	2.78E-06	6.08E-06	queE
AB5991_07700	0.73	5.33	2.72	21.71	23.27	13.75	2.93	19.58	2.7418	1.82E-08	4.81E-08	queF
AB5991_07705	0	1.14	0.66	0	5.69	2.69	0.60	2.79	2.2190	0.106280536	0.138147302	ykvR
AB5991_07710	0	0	0	0	0	0	0.00	0.00	0.0000	1	1	ykvS
AB5991_07715	0	0	0	2.02	2.83	3.01	0.00	2.62	11.3554	0.020622285	0.029649202	ykzS
AB5991_07720	18.45	22.23	20.65	0.63	0	1.56	20.44	0.73	-4.8076	1.34E-21	1.01E-20	ykvT
AB5991_07725	5.81	7.19	7.08	0.44	0.55	0.44	6.69	0.48	-3.8117	3.37E-16	1.66E-15	ykvU
AB5991_07730	34.84	59.98	39.2	5.92	1.85	1.96	44.67	3.24	-3.7839	3.42E-17	1.82E-16	stoA
AB5991_07735	1045.3	1354.96	980.91	43.86	62.38	46.92	1127.06	51.05	-4.4644	5.96E-48	2.96E-46	zosA
AB5991_07740	0	0	0	0	0	0	0.00	0.00	0.0000	1	1	--

AB5991_07745	2.31	1.82	4.59	52.78	36.96	20.19	2.91	36.64	3.6561	5.51E-16	2.67E-15	ykvY
AB5991_07750	6.74	10.65	6.2	23.4	15.04	11.55	7.86	16.66	1.0835	0.002954668	0.004754047	ykvZ
AB5991_07755	73.59	78.09	68.44	23.58	25.25	18.96	73.37	22.60	-1.6991	8.62E-10	2.53E-09	glcT
AB5991_07760	19.97	14.07	20.25	11.51	13.23	12.95	18.10	12.56	-0.5265	0.052152221	0.070417025	ptsG
AB5991_07765	14.89	17.4	18.1	131.79	135.73	79.14	16.80	115.55	2.7823	4.32E-15	1.96E-14	ptsH
AB5991_07770	20.89	24.02	25.61	243.06	232.18	165.51	23.51	213.58	3.1837	7.05E-27	7.91E-26	ptsI
AB5991_07775	30.12	15.21	35.44	3.28	4.6	2.45	26.92	3.44	-2.9670	1.05E-08	2.83E-08	splA
AB5991_07780	76.94	53.22	81.34	23.5	17.7	15.4	70.50	18.87	-1.9018	5.56E-10	1.66E-09	splB
AB5991_07785	52.01	54.84	71.31	10.64	6.55	3.62	59.39	6.94	-3.0978	4.92E-17	2.60E-16	ykwB
AB5991_07790	14.7	17.54	13.95	3.8	4.21	2.78	15.40	3.60	-2.0979	1.06E-11	3.68E-11	mcpC
AB5991_07795	4.38	8.04	4.9	32.42	31.83	26.63	5.77	30.29	2.3915	9.97E-13	3.80E-12	ykwC
AB5991_07805	59.31	62.66	52.41	44.95	47.02	43.51	58.13	45.16	-0.3642	0.155931919	0.195209852	NGR_a01970
AB5991_07815	56.41	61.03	60.97	105.66	68	58.58	59.47	77.41	0.3804	0.183504813	0.226937905	pbpH
AB5991_07820	4.35	6.17	4.33	38.56	35.69	28.44	4.95	34.23	2.7898	6.63E-20	4.43E-19	kinA
AB5991_07825	139.46	197.95	162.06	79.83	88.24	62.9	166.49	76.99	-1.1127	4.92E-05	9.52E-05	dapX
AB5991_07830	79.22	114.73	100.23	0	2.27	1.21	98.06	1.16	-6.4015	1.04E-28	1.30E-27	ykzT
AB5991_07835	218.41	371.19	203.68	8.41	9.88	10.73	264.43	9.67	-4.7727	3.53E-40	9.95E-39	cheV
AB5991_07840	376.66	792.25	507.14	29.59	17.8	11.36	558.68	19.58	-4.8343	1.82E-30	2.59E-29	ykyB
AB5991_07845	22.93	29.01	33.64	13.53	14.37	8.02	28.53	11.97	-1.2525	6.07E-05	0.000116233	ykuC
AB5991_07850	10.59	14.08	16.79	4.77	6.69	3.56	13.82	5.01	-1.4648	0.000298764	0.00053393	ykuD
AB5991_07855	13.23	11.18	15.94	10.5	11.11	7.5	13.45	9.70	-0.4711	0.141612084	0.179089677	ykuE
AB5991_07860	28.12	31.67	22.49	123.34	98.83	95.4	27.43	105.86	1.9485	2.36E-11	7.97E-11	fadH
AB5991_07865	135.71	132.86	128.39	87.85	88.52	62.02	132.32	79.46	-0.7357	0.005313769	0.00823935	ykuI
AB5991_07870	0	1.38	2.42	13.92	9.2	4.08	1.27	9.07	2.8395	0.000779674	0.001340878	ykuJ
AB5991_07875	3.83	9.59	7.08	31.82	42.89	10.56	6.83	28.42	2.0564	5.81E-05	0.000111521	ykuK
AB5991_07880	5.48	6.7	9.76	20.85	10.22	5.93	7.31	12.33	0.7540	0.230980883	0.279556565	ykzF

AB5991_07885	0.81	7.48	3.05	18.15	18.64	22.92	3.78	19.90	2.3966	6.20E-07	1.44E-06	ykuL
AB5991_07890	55.13	98.96	53.47	7.36	6.67	3.55	69.19	5.86	-3.5615	1.12E-20	8.00E-20	ykuM
AB5991_07895	0	0	0	1.24	0.77	0	0.00	0.67	9.3880	0.076895994	0.101920558	ykuN
AB5991_07900	2.02	1.11	1.73	0.44	1.44	1.75	1.62	1.21	-0.4210	0.594809884	0.651044667	ykuO
AB5991_07905	1.19	1.46	2.12	1.72	0	0.43	1.59	0.72	-1.1497	0.364091716	0.421272787	ykuP
AB5991_07910	3.81	12.14	3.26	26.82	21.73	12.38	6.40	20.31	1.6653	0.000466748	0.000821204	dapH
AB5991_07915	4.18	5.9	5.67	21.67	21.09	17.91	5.25	20.22	1.9456	7.47E-10	2.21E-09	ykuR
AB5991_07920	130.05	237.45	153.22	35.96	32.16	28.63	173.57	32.25	-2.4282	2.88E-13	1.14E-12	ykuS
AB5991_07925	4.72	4.95	5.29	2.45	1.83	3.16	4.99	2.48	-1.0077	0.018398818	0.026694083	ykuT
AB5991_07930	16.31	26.28	22.07	242.56	275.77	247.91	21.55	255.41	3.5669	2.94E-31	4.37E-30	ykuU
AB5991_07935	18.2	24.5	24.22	223.41	246.51	204.85	22.31	224.92	3.3339	4.62E-27	5.24E-26	ykuV
AB5991_07940	1280.62	1662.91	1427.89	172.01	139.89	89.34	1457.14	133.75	-3.4456	5.22E-27	5.89E-26	rok
AB5991_07945	0.45	0.62	0.61	1.39	0.97	0.74	0.56	1.03	0.8838	0.223060227	0.270880578	insK
AB5991_07950	33.31	27.83	34.62	3.26	3.24	4.86	31.92	3.79	-3.0755	5.11E-20	3.44E-19	cse15
AB5991_07955	1.2	1.11	5.15	3.6	3.68	4.57	2.49	3.95	0.6676	0.32369557	0.379060781	mobA
AB5991_07960	1.77	2.28	2.27	10.21	10.82	6.71	2.11	9.25	2.1340	1.39E-07	3.40E-07	moeB
AB5991_07965	7.13	6.42	5.68	23.41	27.89	20.58	6.41	23.96	1.9022	2.31E-10	7.14E-10	moeA
AB5991_07970	7.27	3.81	5.18	20.71	20.79	19.49	5.42	20.33	1.9072	1.94E-07	4.71E-07	mobB
AB5991_07975	3.81	8.4	8.56	26.54	36.09	28.9	6.92	30.51	2.1397	1.04E-08	2.80E-08	moaE
AB5991_07980	1.54	5.67	1.65	4.2	12.58	16.72	2.95	11.17	1.9188	0.006769454	0.010378959	moaD
AB5991_07990	57.56	84.3	51.44	129.1	137.84	138.85	64.43	135.26	1.0699	0.000112491	0.000209719	yknV
AB5991_07995	56.61	95.85	68.32	64.68	59.21	51.16	73.59	58.35	-0.3348	0.258412502	0.310392028	yknW
AB5991_08000	17.21	63.51	15.34	10.92	10.22	8.11	32.02	9.75	-1.7155	0.00043489	0.000767874	yknX
AB5991_08005	149.45	179.59	147.27	18.15	16.46	16.09	158.77	16.90	-3.2318	1.13E-27	1.33E-26	yknY
AB5991_08010	409.64	487.27	400.51	43.63	37.44	34.41	432.47	38.49	-3.4899	1.21E-33	2.11E-32	yknZ
AB5991_08015	100.89	108.87	129.89	54.09	47.21	58.23	113.22	53.18	-1.0902	5.11E-05	9.86E-05	fruR

AB5991_08020	111.38	100.44	137.13	50.44	41.96	50.85	116.32	47.75	-1.2845	1.99E-06	4.39E-06	fruK
AB5991_08025	123.44	94.28	119.95	40.59	39.05	50.76	112.56	43.47	-1.3727	3.02E-07	7.23E-07	fruA
AB5991_08030	33.85	112.91	49.49	73.97	78.39	53.79	65.42	68.72	0.0710	0.849181211	0.891382127	sipT
AB5991_08035	2.68	1.23	1.43	0	1.36	1.45	1.78	0.94	-0.9263	0.580864063	0.637537644	ykoA
AB5991_08040	92.88	128.22	115.88	123.18	87.73	69.56	112.33	93.49	-0.2648	0.361780881	0.418843113	ykpA
AB5991_08045	26.56	43.31	29.04	45.7	44.17	39.69	32.97	43.19	0.3894	0.168946267	0.209914151	ykpB
AB5991_08050	6.89	13.46	8.94	23.6	20.89	11.58	9.76	18.69	0.9368	0.009406978	0.014133602	ampS
AB5991_08055	4.02	17.21	2.86	2.91	2.73	1.45	8.03	2.36	-1.7646	0.083110141	0.10953547	ykpC
AB5991_08060	116.73	108.33	105.66	17.36	20.26	11.26	110.24	16.29	-2.7583	8.41E-20	5.59E-19	mreBH
AB5991_08065	24.62	84.46	13.86	69.05	37.58	39.27	40.98	48.63	0.2470	0.636908195	0.692921217	abh
AB5991_08070	3.52	5.17	3.31	18.53	14.04	16.15	4.00	16.24	2.0215	1.07E-09	3.12E-09	kinC
AB5991_08075	3.68	9.15	5.1	7.31	7.06	2.11	5.98	5.49	-0.1217	0.867103005	0.907316477	ykqA
AB5991_08080	56.72	82.72	51.37	30.11	30.66	17.04	63.60	25.94	-1.2941	5.48E-05	0.000105469	ktrC
AB5991_08085	5.11	6.7	2.79	8.62	7.96	2.93	4.87	6.50	0.4182	0.35120377	0.40790587	adeC
AB5991_08090	75.1	123.56	89.81	311.72	272.41	130.91	96.16	238.35	1.3096	0.000158553	0.000290681	rnjA
AB5991_08095	3.44	23.71	5.52	43.06	35.04	35.41	10.89	37.84	1.7968	0.000644073	0.001118326	rpoY
AB5991_08100	3.5	9.86	5.99	1.78	3.33	3.03	6.45	2.71	-1.2492	0.015236182	0.022325786	ykrA
AB5991_08105	101.94	226.64	130.95	5.67	10.28	3.17	153.18	6.37	-4.5870	2.58E-24	2.36E-23	defB
AB5991_08110	352.4	96.02	465.61	43.27	93.71	129.21	304.68	88.73	-1.7798	0.000397506	0.000705316	--
AB5991_08120	149.81	219.48	179.61	110.44	130.88	130.44	182.97	123.92	-0.5622	0.035233521	0.048835013	pdhA
AB5991_08125	259.3	260.63	260.69	117.18	149.35	166.85	260.21	144.46	-0.8490	0.001216718	0.002055994	pdhB
AB5991_08130	656.9	521.68	620.02	459.58	537.62	528.98	599.53	508.73	-0.2369	0.353788325	0.410667477	pdhC
AB5991_08135	835.31	614.45	707.38	841	1077.32	1012.09	719.05	976.80	0.4420	0.085217102	0.112089139	pdhD
AB5991_08140	38.08	111.52	60.31	1.57	2.94	0	69.97	1.50	-5.5405	2.67E-20	1.84E-19	slp
AB5991_08145	4.52	41.49	16.11	98.29	113.44	34.24	20.71	81.99	1.9854	0.001397959	0.002339358	ykzW
AB5991_08150	7.36	5.86	9.58	2.54	1.87	2.52	7.60	2.31	-1.7181	1.96E-06	4.34E-06	speA

AB5991_08155	0	2.49	1.45	2.95	6.89	0	1.31	3.28	1.3205	0.243300744	0.293751065	yktA
AB5991_08160	2.55	2.6	3.93	8.61	9.79	3.67	3.03	7.36	1.2813	0.01048462	0.015610959	yktB
AB5991_08165	170.23	321.35	185.11	52.01	37.96	48.66	225.56	46.21	-2.2873	2.65E-11	8.91E-11	ykzI
AB5991_08170	26.5	93.58	24.71	26.11	20.29	27.71	48.26	24.70	-0.9662	0.032578702	0.045361159	suhB
AB5991_08175	120.92	282.38	110.84	11.46	14.15	10.95	171.38	12.19	-3.8138	2.20E-19	1.41E-18	ykzC
AB5991_08180	11.86	10.01	7.06	2.24	3.01	0.9	9.64	2.05	-2.2339	3.21E-07	7.67E-07	--
AB5991_08185	61.23	35.68	53.52	8.54	4.59	2.97	50.14	5.37	-3.2240	1.21E-16	6.22E-16	yktD
AB5991_08190	923.71	1086.59	1137.42	11587.48	15249.32	14910.24	1049.24	13915.68	3.7293	3.70E-42	1.15E-40	nprE
AB5991_08195	0.37	0	0.6	0	0	0.2	0.32	0.07	-2.2780	0.114465337	0.148011023	ylaA
AB5991_08200	0.67	0	1.43	0	0	0	0.70	0.00	-9.4512	0.264701314	0.316391271	ylaB
AB5991_08210	2.46	1.13	1.31	0.67	1.25	0	1.63	0.64	-1.3517	0.380565037	0.438035873	ylaD
AB5991_08215	95.69	77.55	136.76	26.98	43.89	18.54	103.33	29.80	-1.7938	5.52E-07	1.29E-06	ylaE
AB5991_08220	0	1.76	1.02	8.32	6.81	6.21	0.93	7.11	2.9404	0.001713285	0.002827704	ylaF
AB5991_08225	241.69	221.44	246.27	151.26	112.84	71.71	236.47	111.94	-1.0790	0.000384211	0.000683544	bipA
AB5991_08230	67.07	70.97	94.22	92.73	82.15	60.3	77.42	78.39	0.0180	0.966799825	1	ylaH
AB5991_08235	2.58	3.16	0.92	0	0	1.86	2.22	0.62	-1.8402	0.324211909	0.379553505	ylaI
AB5991_08240	32.71	31.08	34.06	16.54	9.05	17.39	32.62	14.33	-1.1869	0.00024738	0.000446319	ylaJ
AB5991_08245	31.82	53.94	34.32	3.7	3.74	3.98	40.03	3.81	-3.3944	8.46E-24	7.45E-23	ylaK
AB5991_08250	24.55	27.32	22.67	300.53	372.08	340.6	24.85	337.74	3.7648	8.83E-37	1.96E-35	ylaL
AB5991_08255	20.41	29.62	18.91	7.4	6.13	3.37	22.98	5.63	-2.0283	3.60E-08	9.30E-08	glsA2
AB5991_08260	11.54	16.48	19.19	46.01	21.53	18.04	15.74	28.53	0.8582	0.055640833	0.074949032	ylaN
AB5991_08265	10.44	14.24	9.25	18.17	19.28	10.49	11.31	15.98	0.4987	0.134470846	0.171643758	ftsW
AB5991_08270	52.44	56.04	44.13	268.44	352.87	323.6	50.87	314.97	2.6303	1.91E-21	1.42E-20	pyc
AB5991_08275	1017.97	1264.5	1087.15	78.33	98.67	110.9	1123.21	95.97	-3.5489	2.19E-34	4.08E-33	ctaA
AB5991_08280	317	382.14	337.53	123.34	125.85	120	345.56	123.06	-1.4895	8.38E-09	2.28E-08	ctaB2
AB5991_08285	67.17	88.01	70.57	340.48	424.77	386.76	75.25	384.00	2.3514	2.31E-17	1.24E-16	ctaC

AB5991_08290	248.35	237.24	204.57	668.84	944.6	964.07	230.05	859.17	1.9010	1.47E-12	5.53E-12	ctaD
AB5991_08295	291.4	306.36	245.33	961.48	1376.74	1451.78	281.03	1263.33	2.1684	1.35E-14	5.93E-14	ctaE
AB5991_08300	227.43	314.95	200.26	954.57	1184.96	1188.66	247.55	1109.40	2.1640	7.13E-14	2.94E-13	ctaF
AB5991_08305	63.08	60.14	59.46	95.21	122.43	123.44	60.89	113.69	0.9008	0.000554557	0.000971393	ctaG
AB5991_08310	663.74	810.07	420.13	2814.41	2938.27	2531.2	631.31	2761.29	2.1289	1.65E-12	6.15E-12	ylbA
AB5991_08315	6.87	12.62	9.95	8.8	8.23	4.38	9.81	7.14	-0.4595	0.340968496	0.397529624	ylbB
AB5991_08320	33.86	50.05	43.08	26.44	28.45	20.11	42.33	25.00	-0.7598	0.007814826	0.011853581	ylbC
AB5991_08325	96.49	85.68	88.65	9.85	18.9	20.11	90.27	16.29	-2.4706	2.23E-14	9.55E-14	ylbD
AB5991_08330	45.94	51.17	87.79	6.55	10.73	19.57	61.63	12.28	-2.3270	9.99E-08	2.48E-07	ylbE
AB5991_08335	33.74	55.32	37.8	64.65	59.68	41.74	42.29	55.36	0.3886	0.204958206	0.251122813	ylbF
AB5991_08340	4.63	9.73	2.83	10.8	9.43	4.3	5.73	8.18	0.5130	0.38727239	0.444596317	ylbG
AB5991_08345	14	17.34	16.37	4.25	6.3	1.76	15.90	4.10	-1.9545	3.25E-06	7.07E-06	ylbH
AB5991_08350	33.47	50.53	24.66	10.92	14.38	6.84	36.22	10.71	-1.7574	6.16E-06	1.31E-05	coaD
AB5991_08355	6.92	14.61	11.03	0.8	1.5	0.8	10.85	1.03	-3.3928	7.43E-13	2.86E-12	ylbJ
AB5991_08360	0	2.54	0.99	1.26	3.29	1.75	1.18	2.10	0.8357	0.279486737	0.331493976	ylbK
AB5991_08365	16.56	33.32	15.07	6.51	6.63	2.67	21.65	5.27	-2.0385	2.26E-06	4.99E-06	ylbL
AB5991_08370	12.02	15.42	13.32	29.77	26.53	26.5	13.59	27.60	1.0225	0.000160318	0.000293644	tmcAL
AB5991_08375	0	0	2.08	0	0	0	0.69	0.00	-9.4374	0.51237006	0.569081258	ylzH
AB5991_08380	51.89	42.85	58.1	157.95	135.75	65.22	50.95	119.64	1.2316	0.000384375	0.000683544	ylbN
AB5991_08385	27.11	16.59	24.7	160.54	146.15	101.09	22.80	135.93	2.5757	7.51E-13	2.89E-12	rpmF
AB5991_08390	73.29	105.5	60.78	8.44	9.48	7.4	79.86	8.44	-3.2421	5.67E-21	4.12E-20	ylbO
AB5991_08395	175.14	302.34	153.27	850.63	976.92	825.18	210.25	884.24	2.0723	5.02E-11	1.64E-10	ylbP
AB5991_08400	2.02	2.22	1.29	8.55	8	5.45	1.84	7.33	1.9922	4.00E-06	8.61E-06	panE
AB5991_08405	4.57	7.58	5.73	23.42	23.73	11.96	5.96	19.70	1.7251	1.12E-06	2.55E-06	bshC
AB5991_08410	4.6	13.83	8.05	79.18	62.17	45.29	8.83	62.21	2.8173	2.53E-12	9.31E-12	mraZ
AB5991_08415	5.6	12.06	9.5	83.8	65.05	33.24	9.05	60.70	2.7451	6.20E-12	2.21E-11	rsmH

AB5991_08420	8.17	10.31	8.19	76.63	56.64	33.16	8.89	55.48	2.6416	6.62E-11	2.15E-10	ftsL
AB5991_08425	21.6	22.53	19.41	31.53	22.75	14.19	21.18	22.82	0.1078	0.735313396	0.785138343	pbpB
AB5991_08430	23.75	42.06	30.27	23.5	17.63	11.59	32.03	17.57	-0.8659	0.010204656	0.015239823	spoVD
AB5991_08435	4.75	6.03	3.9	38.65	35.8	21.61	4.89	32.02	2.7101	1.21E-15	5.68E-15	murE
AB5991_08440	21.88	17.02	20.22	47.78	48.11	39.73	19.71	45.21	1.1979	1.04E-05	2.14E-05	mraY
AB5991_08445	25.73	21.78	21.81	57.7	51.42	34.92	23.11	48.01	1.0551	0.000356698	0.000635463	murD
AB5991_08450	61.56	82.6	58.64	22.68	21.22	11.55	67.60	18.48	-1.8708	1.21E-08	3.24E-08	spoVE
AB5991_08455	196.64	311.53	158.07	93.07	74.63	63.61	222.08	77.10	-1.5262	1.15E-06	2.60E-06	murG
AB5991_08460	238.62	393.76	196.9	60.35	47	43.12	276.43	50.16	-2.4624	4.73E-14	1.98E-13	murB
AB5991_08465	7.76	7.96	6.1	14.64	16.03	4.94	7.27	11.87	0.7066	0.104803218	0.136405631	divIB
AB5991_08470	6.75	6.2	6.67	11.3	9.52	7.87	6.54	9.56	0.5482	0.127335461	0.163323358	ylxW
AB5991_08475	23.23	29.06	25.39	19.99	11.95	11.88	25.89	14.61	-0.8260	0.010811879	0.016080139	ylxX
AB5991_08480	0	0	0	0.54	4.52	0.53	0.00	1.86	10.8637	0.010308543	0.015383399	sbp
AB5991_08485	66.4	49.67	57.56	304.6	288.24	280.4	57.88	291.08	2.3304	9.64E-18	5.42E-17	ftsA
AB5991_08490	150.39	163.2	136.77	543.38	532.82	491.62	150.12	522.61	1.7996	3.26E-12	1.19E-11	ftsZ
AB5991_08495	409.19	430.33	304.41	2137.03	1984.08	1929.73	381.31	2016.95	2.4031	1.32E-19	8.62E-19	bpr
AB5991_08500	3.69	15.35	4.78	2.75	2.77	3.79	7.94	3.10	-1.3553	0.016020079	0.023405348	spoIIGA
AB5991_08505	11.05	13.37	9.93	4.91	2.81	1.09	11.45	2.94	-1.9631	1.37E-05	2.80E-05	sigE
AB5991_08510	55.17	64	71.1	6.28	5.64	4.5	63.42	5.47	-3.5345	1.06E-28	1.32E-27	sigG
AB5991_08515	0.23	0.42	1.22	4.45	3.47	0.49	0.62	2.80	2.1691	0.013316665	0.019665481	ylmA
AB5991_08520	7.62	6.74	6.79	4.6	4.6	6.57	7.05	5.26	-0.4235	0.200608582	0.246173263	ylmB
AB5991_08525	32.33	63.41	34.57	11.99	9.72	12.73	43.44	11.48	-1.9198	3.93E-06	8.48E-06	ylmC
AB5991_08530	26.78	28.95	34.18	94.65	77.58	50.03	29.97	74.09	1.3057	2.47E-05	4.95E-05	ylmD
AB5991_08535	781.94	755.73	768.15	100.42	85.21	49.97	768.61	78.53	-3.2909	1.71E-24	1.59E-23	ylmE
AB5991_08540	697.23	738.75	687.97	209.94	189.61	128.72	707.98	176.09	-2.0074	1.33E-12	5.02E-12	sepF
AB5991_08545	274.1	240.71	263.39	114.49	110.51	75.97	259.40	100.32	-1.3705	1.61E-06	3.61E-06	ylmG

AB5991_08550	2.8	2.14	3	15.49	26.14	6.07	2.65	15.90	2.5868	1.42E-06	3.20E-06	ylmH
AB5991_08555	69.01	48.95	81.61	126.29	125.24	71.54	66.52	107.69	0.6950	0.030012554	0.042068427	divIVA
AB5991_08560	1625.44	1400.63	1847.33	100.56	113.53	99.39	1624.47	104.49	-3.9585	4.72E-46	1.93E-44	ileS
AB5991_08565	46.75	64.61	61.34	1.05	0.98	0	57.57	0.68	-6.4106	1.13E-33	1.99E-32	ylyA
AB5991_08570	0.79	2.17	2.95	3.43	5.21	0	1.97	2.88	0.5479	0.592328643	0.649007111	lspA
AB5991_08575	15.86	17.83	15.47	14.23	10.49	6.87	16.39	10.53	-0.6380	0.056799753	0.076328873	ylyB
AB5991_08580	66.87	111.85	59.48	88.93	19.54	31.54	79.40	46.67	-0.7666	0.100859781	0.131531753	pyrR
AB5991_08585	17.87	44	18.07	196.58	26.22	28.94	26.65	83.91	1.6549	0.009557167	0.014342974	pyrP
AB5991_08590	3.56	11.97	3.8	140.51	11.06	6.42	6.44	52.66	3.0309	0.000695973	0.001203706	pyrB
AB5991_08595	7.86	58.02	9.46	363.68	24.3	11.25	25.11	133.08	2.4057	0.015073967	0.022104402	pyrC
AB5991_08600	21.29	107.3	28.42	505.9	32.42	22.69	52.34	187.00	1.8372	0.03937648	0.054142659	pyrAA
AB5991_08605	56.37	185.35	55.78	528.68	56.74	24.88	99.17	203.43	1.0366	0.192088782	0.236742567	pyrAB
AB5991_08610	40.79	145.07	37.86	488.78	74.68	22.59	74.57	195.35	1.3893	0.077525136	0.102685895	pyrK
AB5991_08615	50.21	143.96	27.05	484.52	71.15	21.95	73.74	192.54	1.3846	0.085056355	0.111951868	pyrD
AB5991_08620	35.4	84.36	20.13	338.56	61.32	22.01	46.63	140.63	1.5926	0.029411466	0.041269586	pyrF
AB5991_08625	40.55	109.06	21.68	427.94	78.47	24.73	57.10	177.05	1.6327	0.032329835	0.045046237	pyrE
AB5991_08630	71.06	75.64	74.62	223.18	161.42	76.37	73.77	153.66	1.0585	0.002589063	0.004199824	cysH
AB5991_08635	26.82	25.24	19.78	196.4	135.59	67.24	23.95	133.08	2.4744	4.50E-11	1.49E-10	cysP
AB5991_08640	38.23	20.51	21.36	316.86	214.86	86.51	26.70	206.08	2.9483	2.17E-11	7.36E-11	sat
AB5991_08645	45.34	45.26	22.13	283.95	207.19	113.31	37.58	201.48	2.4228	4.90E-10	1.47E-09	cysC
AB5991_08650	68.66	69.04	62.68	362.94	321.1	172.15	66.79	285.40	2.0952	4.64E-11	1.53E-10	sumT
AB5991_08655	109.69	107.67	100.83	218.84	192.15	86.88	106.06	165.96	0.6459	0.050296688	0.06807367	sirB
AB5991_08660	76.51	67.19	67.59	160	140.7	76.82	70.43	125.84	0.8373	0.009032468	0.013596638	sirC
AB5991_08665	21.21	25.96	23.92	23.41	15.57	15.88	23.70	18.29	-0.3739	0.183300582	0.226826616	rqcH
AB5991_08670	144.17	188.11	135.95	16.91	14.87	10.32	156.08	14.03	-3.4753	3.23E-28	3.89E-27	yloB
AB5991_08675	51.17	46.98	39.94	35.23	37.17	13.62	46.03	28.67	-0.6829	0.053239464	0.071811791	yloC

AB5991_08680	26.11	22.13	19.33	8.01	25.21	7.97	22.52	13.73	-0.7141	0.143484676	0.181111776	remA
AB5991_08685	76.71	66.92	72.92	49.54	50.55	28.32	72.18	42.80	-0.7539	0.010281764	0.015349206	gmk
AB5991_08690	28.35	32.54	29.37	16.38	24.35	9.59	30.09	16.77	-0.8430	0.037872784	0.052201606	rpoZ
AB5991_08695	6.51	7.88	10.92	12.07	13.11	6.25	8.44	10.48	0.3124	0.410796761	0.468218696	coaBC
AB5991_08700	12.71	13.73	10.07	23.25	20.85	17.96	12.17	20.69	0.7654	0.005383226	0.008337281	priA
AB5991_08705	3.74	6.18	1.6	4.07	11.05	3.65	3.84	6.26	0.7043	0.231649305	0.280280131	defA
AB5991_08710	12.32	15.31	10.74	24.11	24.1	21.74	12.79	23.32	0.8663	0.002941577	0.004734901	fmt
AB5991_08715	11.3	12.84	12.37	35.84	37.5	27.51	12.17	33.62	1.4658	1.27E-07	3.11E-07	rsmB
AB5991_08720	14.07	20.06	14.16	62.47	53.57	47.48	16.10	54.51	1.7597	1.58E-09	4.56E-09	rlmN
AB5991_08725	15.59	15.62	10.36	75.29	91.38	76.73	13.86	81.13	2.5497	1.22E-17	6.76E-17	prpC
AB5991_08730	15.69	13.98	17.18	101.07	107.62	97.88	15.62	102.19	2.7101	2.87E-23	2.41E-22	prkC
AB5991_08735	43.52	61.42	39.87	137.63	172.88	154.87	48.27	155.13	1.6842	1.68E-09	4.84E-09	rsgA
AB5991_08740	33.16	20.81	22.17	85.06	90.57	74.79	25.38	83.47	1.7176	3.99E-09	1.11E-08	rpe
AB5991_08745	197.28	214.57	225.36	14.63	24.53	27	212.40	22.05	-3.2677	1.54E-25	1.58E-24	thiN
AB5991_08750	0	0	2.39	0	0	0	0.80	0.00	-9.6378	1	1	spoVM
AB5991_08755	18.17	31.61	19.43	210.1	142.11	91.1	23.07	147.77	2.6793	3.23E-11	1.08E-10	rpmB
AB5991_08760	0.5	1.83	2.66	10.29	14.19	5.93	1.66	10.14	2.6074	3.07E-05	6.09E-05	yloU
AB5991_08765	35.89	49.92	32.91	80.79	63.2	43.32	39.57	62.44	0.6579	0.031728237	0.044285704	yloV
AB5991_08770	9.81	11.51	11.95	16.6	12.21	7.97	11.09	12.26	0.1447	0.714303326	0.765586642	sdaAB
AB5991_08775	23.82	22.05	22.05	61.61	60.91	49.4	22.64	57.31	1.3398	5.75E-07	1.34E-06	sdaAA
AB5991_08780	9.53	11.99	10.28	36.94	42.11	31.89	10.60	36.98	1.8027	2.55E-10	7.88E-10	recG
AB5991_08785	14.66	25.76	20.11	61.71	108.04	56.59	20.18	75.45	1.9028	3.58E-08	9.27E-08	fapR
AB5991_08790	4.51	5.96	8.1	58.27	77.48	41.01	6.19	58.92	3.2507	1.97E-19	1.27E-18	plsX
AB5991_08795	2.65	2.44	3.85	27.82	43.58	22.97	2.98	31.46	3.4000	1.28E-17	7.07E-17	fabD
AB5991_08800	2.2	2.69	3.13	59.96	53.38	39.87	2.67	51.07	4.2558	2.89E-29	3.72E-28	fabG
AB5991_08805	121.27	109.21	88.39	798.92	735.84	578.62	106.29	704.46	2.7285	4.96E-21	3.62E-20	acpA

AB5991_08810	5.78	10.62	10.57	29.09	41.45	15.65	8.99	28.73	1.6762	4.74E-05	9.20E-05	rnc
AB5991_08815	9.29	14.54	10.26	41.07	42.93	33.63	11.36	39.21	1.7868	3.41E-10	1.04E-09	smc
AB5991_08820	12.23	14.08	8.79	61.95	66.15	58.5	11.70	62.20	2.4104	1.67E-16	8.45E-16	ftsY
AB5991_08825	15.99	7.51	17.9	29.53	43.91	38.65	13.80	37.36	1.4370	6.13E-05	0.000117331	ylqB
AB5991_08830	142.21	145.51	142.79	89.73	77.89	47.59	143.50	71.74	-1.0003	0.000669086	0.001159224	ylxM
AB5991_08835	325.11	379.66	350.7	516.59	485.07	444.43	351.82	482.03	0.4543	0.069596433	0.092803034	ffh
AB5991_08840	222.46	209.1	235.07	382.36	322.1	254.43	222.21	319.63	0.5245	0.052190552	0.07042089	rpsP
AB5991_08845	122.7	140.31	128.08	233.34	233.31	178.16	130.36	214.94	0.7214	0.007486789	0.011390819	khpA
AB5991_08850	0.93	0	0	2.03	2.38	0	0.31	1.47	2.2455	0.165282832	0.205619713	ylqD
AB5991_08855	3.1	1.9	3.68	5.99	6.31	2.98	2.89	5.09	0.8159	0.118132753	0.152356337	rimM
AB5991_08860	6.17	8.61	5.55	28.74	20.36	20.85	6.78	23.32	1.7827	1.31E-07	3.21E-07	trmD
AB5991_08865	592.63	787.76	517.12	2320.56	2361.86	2147.21	632.50	2276.54	1.8477	1.31E-11	4.50E-11	rplS
AB5991_08870	8.94	8.99	9.56	24.54	25.57	21.2	9.16	23.77	1.3752	4.35E-06	9.35E-06	rbgA
AB5991_08875	19.53	25.06	15.6	1.54	1.92	1.53	20.06	1.66	-3.5924	6.03E-18	3.43E-17	rnhB
AB5991_08880	32.79	41.8	33.72	5.45	5.21	2.15	36.10	4.27	-3.0798	1.54E-18	9.17E-18	ylqG
AB5991_08885	3.2	4.71	3.43	0.7	0	0	3.78	0.23	-4.0179	0.001391187	0.002329989	ylqH
AB5991_08890	197.61	170.82	189.12	274.33	347.9	372.91	185.85	331.71	0.8358	0.001555463	0.00258225	sucC
AB5991_08895	124.7	128.64	97.18	805.7	989.26	986.97	116.84	927.31	2.9885	1.84E-26	1.99E-25	sucD
AB5991_08900	41.04	29.33	40.65	24.63	30.04	28.01	37.01	27.56	-0.4252	0.110656327	0.143506294	dprA
AB5991_08905	359.67	458.19	329.42	69.88	65.75	42.04	382.43	59.22	-2.6909	6.54E-19	4.00E-18	topA
AB5991_08910	29.02	43.64	28.96	22.39	26.16	16.6	33.87	21.72	-0.6413	0.033125798	0.046058313	trmFO
AB5991_08915	14.82	19.59	13.94	27.5	25.53	20.53	16.12	24.52	0.6054	0.03832056	0.052782151	xerC
AB5991_08920	35.75	27.96	35.76	58.33	55.93	41.21	33.16	51.82	0.6443	0.025563347	0.036176782	clpQ
AB5991_08925	49.76	55.9	48.29	179.47	213.38	180.15	51.32	191.00	1.8961	5.32E-13	2.07E-12	clpY
AB5991_08930	50.52	80.85	51.79	213.47	275.47	233.54	61.05	240.83	1.9799	1.21E-11	4.19E-11	codY
AB5991_08935	1.85	2.55	5.45	5.54	2.36	3.51	3.28	3.80	0.2121	0.903963225	0.941914975	flgB

AB5991_08940	2.39	1.47	3.41	6.51	2.44	2.59	2.42	3.85	0.6666	0.345034768	0.401563032	flgC
AB5991_08945	1.13	0	3.01	2.45	2.29	0.61	1.38	1.78	0.3699	1	1	fliE
AB5991_08955	3.55	5.87	2.28	1.35	1.45	1.73	3.90	1.51	-1.3689	0.009334615	0.014040817	fliG
AB5991_08960	2.16	8.82	2.31	0.52	0.73	0	4.43	0.42	-3.4103	9.70E-05	0.00018195	fliH
AB5991_08965	3.71	9.58	4.84	1.19	0.28	1.04	6.04	0.84	-2.8526	3.05E-07	7.30E-07	fliI
AB5991_08970	0.81	2.24	0.44	0	0	0.44	1.16	0.15	-2.9877	0.094017247	0.123134066	fliJ
AB5991_08975	4.41	3.24	1.57	0	0	0.32	3.07	0.11	-4.8486	1.43E-05	2.91E-05	ylxF
AB5991_08980	6.91	7.48	7.26	0.54	0.38	0.27	7.22	0.40	-4.1853	1.29E-19	8.44E-19	fliK
AB5991_08985	4.27	5.49	2.28	0	0	0	4.01	0.00	-11.9706	4.98E-06	1.06E-05	ylxG
AB5991_08990	10.23	11.69	12.64	1.24	2.31	2.46	11.52	2.00	-2.5237	3.56E-10	1.08E-09	flgG
AB5991_08995	0	0	0	1.82	0.85	0	0.00	0.89	9.7977	0.265222375	0.316560881	swrD
AB5991_09000	1.28	0	1.37	1.39	1.74	0.93	0.88	1.35	0.6155	0.81026625	0.853692566	fliL
AB5991_09005	0.72	1.33	0.39	0.79	2.03	2.55	0.81	1.79	1.1380	0.105707814	0.137492869	fliM
AB5991_09010	0	0	0.51	0.86	1.29	1.89	0.17	1.35	2.9858	0.001367279	0.00229188	fliY
AB5991_09015	0.5	1.83	0.53	0	0.51	1.62	0.95	0.71	-0.4252	1	1	cheY
AB5991_09020	1.37	1.51	1.46	1.49	1.39	2.67	1.45	1.85	0.3548	0.647440369	0.702455111	fliZ
AB5991_09025	0.27	0	2.03	0.3	1.1	0.88	0.77	0.76	-0.0126	1	1	fliP
AB5991_09030	0	0	0	0	1.36	0	0.00	0.45	8.8244	0.5127607	0.569081258	fliQ
AB5991_09040	1.67	1.84	1.07	0.36	0.68	0	1.53	0.35	-2.1388	0.004514051	0.007061206	flhB
AB5991_09045	0.62	0.33	0.95	0.97	0.72	1.15	0.63	0.95	0.5799	0.343838441	0.400288024	flhA
AB5991_09050	1.15	0.6	0.53	0.54	0.5	0.71	0.76	0.58	-0.3817	0.699268988	0.752315674	flhF
AB5991_09055	3.83	3.33	3.45	0.44	1.03	0.22	3.54	0.56	-2.6503	6.08E-06	1.29E-05	ylxH
AB5991_09060	1.18	1.85	1.26	1.1	0.69	0.55	1.43	0.78	-0.8745	0.228432381	0.276725133	cheB
AB5991_09065	2.33	1.15	2.97	3.6	6.1	4.46	2.15	4.72	1.1345	0.005220083	0.008109918	cheA
AB5991_09070	9.59	13.39	7.8	4.17	10.15	2.49	10.26	5.60	-0.8727	0.100395571	0.130969386	cheW
AB5991_09080	2.89	6.62	5.02	1.18	0.73	0.78	4.84	0.90	-2.4334	0.0002615	0.000470723	cheD

AB5991_09085	6.14	5.64	5.31	5.91	7.21	2.05	5.70	5.06	-0.1719	0.746393165	0.795898834	sigD
AB5991_09090	10.4	8.56	9.59	8.19	6.57	3.88	9.52	6.21	-0.6151	0.134680301	0.171855872	swrB
AB5991_09095	91.47	80.62	103.82	313.84	283.01	180.87	91.97	259.24	1.4951	2.80E-07	6.72E-07	rpsB
AB5991_09100	87.51	71.87	84.16	247.84	205.65	125.78	81.18	193.09	1.2501	3.14E-05	6.21E-05	tsf
AB5991_09105	3.75	6.43	6.15	57.64	58.01	18.67	5.44	44.77	3.0401	6.43E-11	2.09E-10	pyrH
AB5991_09110	27.86	32.71	27.37	87.72	80.11	32.61	29.31	66.81	1.1886	0.001041774	0.001773964	frr
AB5991_09115	12	12.29	17.03	85.36	77.53	40.48	13.77	67.79	2.2992	4.91E-11	1.62E-10	uppS
AB5991_09120	2.45	3.69	6.2	47.08	36.56	22.22	4.11	35.29	3.1007	2.44E-13	9.71E-13	cdsA
AB5991_09125	23.38	29.96	23.32	29.18	33.85	10.53	25.55	24.52	-0.0596	0.888654383	0.927423537	dxr
AB5991_09130	132.61	130.25	132.37	104.72	90.46	66.61	131.74	87.26	-0.5943	0.026750674	0.037725048	rasP
AB5991_09135	126.26	109.06	102.29	137.78	116.13	79.3	112.54	111.07	-0.0189	0.947529057	0.983182097	proS
AB5991_09140	69.34	62.32	66.58	64.66	69.89	60	66.08	64.85	-0.0271	0.915278252	0.952954887	polC
AB5991_09145	28.78	15.5	26.26	26.71	26.95	12.05	23.51	21.90	-0.1023	0.785651997	0.832397034	rimP
AB5991_09150	62.03	53.83	56.46	185.48	160.39	129.21	57.44	158.36	1.4631	5.01E-08	1.27E-07	nusA
AB5991_09155	26.2	19.24	16.81	34.9	37.99	23.39	20.75	32.09	0.6292	0.082223975	0.108547675	ylxR
AB5991_09160	38.18	24.1	16.59	31.14	28.53	23.25	26.29	27.64	0.0722	0.867568577	0.907564494	rplGA
AB5991_09165	1080.46	1291.46	1079.07	347.65	399.82	339.75	1150.33	362.41	-1.6664	3.18E-11	1.07E-10	infB
AB5991_09170	200.18	202.23	191.22	177.56	172.75	154.28	197.88	168.20	-0.2345	0.374755827	0.432477591	ylxP
AB5991_09175	700.02	812.85	613.74	490.34	455.22	413.98	708.87	453.18	-0.6454	0.013327111	0.01967359	rbfA
AB5991_09180	1.36	3.57	2.29	12.89	11.08	9.26	2.41	11.08	2.2024	6.25E-08	1.58E-07	truB
AB5991_09185	4.37	9.77	6.71	30.18	37.33	14.2	6.95	27.24	1.9705	2.63E-06	5.76E-06	ribC
AB5991_09190	236.98	259.37	171.82	1194.77	1105.12	831.92	222.72	1043.94	2.2287	2.38E-14	1.02E-13	rpsO
AB5991_09195	47.53	53.28	49.83	188.32	192.47	122.4	50.21	167.73	1.7400	8.30E-10	2.45E-09	pnp
AB5991_09200	1.51	2.42	3.42	4.71	4.6	2.04	2.45	3.78	0.6269	0.233975367	0.282922102	ylxY
AB5991_09205	14.7	11.6	12.41	12.63	11.96	3.34	12.90	9.31	-0.4709	0.267324823	0.318782845	ymxG
AB5991_09210	390.92	677.93	380.59	12.19	10.7	8.34	483.15	10.41	-5.5364	4.82E-43	1.58E-41	ymxH

AB5991_09215	2695.67	2210.37	2434.98	97.41	173.67	274.89	2447.01	181.99	-3.7491	8.80E-24	7.71E-23	dpaA
AB5991_09220	2400.1	2110.78	2169.83	116.38	197.08	305.66	2226.90	206.37	-3.4317	1.66E-21	1.23E-20	dpaB
AB5991_09225	4412.64	4579.83	4019.81	231.7	312.78	417.82	4337.43	320.77	-3.7572	4.06E-35	8.19E-34	asd
AB5991_09230	1463.4	1206.82	1292.6	105.65	106.14	133.18	1320.94	114.99	-3.5220	6.85E-35	1.33E-33	dapG
AB5991_09235	1792.79	1422.22	1758.91	174.74	191.97	223.45	1657.97	196.72	-3.0752	8.32E-28	9.86E-27	dapA
AB5991_09240	117.47	95.31	105.45	166.06	148.34	98.18	106.08	137.53	0.3746	0.184076245	0.227573714	rnjB
AB5991_09245	16.9	17.09	15.71	0.8	0.5	2.92	16.57	1.41	-3.5579	1.02E-14	4.50E-14	tepA
AB5991_09250	1.7	1.56	3.63	1.85	0	0.92	2.30	0.92	-1.3146	0.382237958	0.439579186	ylzJ
AB5991_09255	7.03	6.46	7.36	40.66	48.09	28.8	6.95	39.18	2.4952	1.13E-15	5.31E-15	spoIIIE
AB5991_09260	13.69	26.06	13.58	48.74	38.26	20.48	17.78	35.83	1.0110	0.00776683	0.011789787	ymfC
AB5991_09265	3.88	6.02	5.42	4.22	4.55	5.81	5.11	4.86	-0.0714	0.92440476	0.962204797	ymfD
AB5991_09270	15.1	23.84	17.81	57.39	54.43	36.66	18.92	49.49	1.3876	5.28E-06	1.13E-05	ymfF
AB5991_09275	45.08	68.34	43.55	157.63	163.23	129.53	52.32	150.13	1.5207	7.98E-08	2.00E-07	ymfH
AB5991_09280	5.7	14.57	3.71	17.26	9.34	9.66	7.99	12.09	0.5965	0.208274968	0.255029262	ymfI
AB5991_09285	641.03	753.83	654.05	61.72	83.42	47.78	682.97	64.31	-3.4088	1.46E-28	1.81E-27	ymfJ
AB5991_09290	6.39	18.44	7.57	63.29	57.37	33.1	10.80	51.25	2.2466	4.50E-08	1.15E-07	ymfK
AB5991_09295	17.51	23.35	21.63	90.69	94.21	56.64	20.83	80.51	1.9506	2.64E-10	8.14E-10	ymfM
AB5991_09300	11.8	17.11	13.95	23.64	22.44	18.15	14.29	21.41	0.5836	0.056716146	0.076242321	pgsA
AB5991_09305	37.57	35.02	33.68	71.5	65.88	58.65	35.42	65.34	0.8833	0.00068667	0.001188134	cinA
AB5991_09310	55.24	69.1	51.51	200.15	157.78	133.8	58.62	163.91	1.4835	1.09E-07	2.70E-07	recA
AB5991_09315	2	2.54	2.3	0.5	0.78	0.67	2.28	0.65	-1.8105	0.001132345	0.00192242	pbpX
AB5991_09320	509.52	621.74	479.22	1504.73	1314.31	1076.58	536.83	1298.54	1.2744	1.14E-06	2.58E-06	rny
AB5991_09325	29.1	40.91	29.66	17.31	15.27	12.31	33.22	14.96	-1.1508	0.000150845	0.00027719	ymdB
AB5991_09330	51.94	94.1	58.51	135.57	107.13	101.95	68.18	114.88	0.7527	0.014129554	0.02078861	spoVS
AB5991_09335	73.93	112.22	64.62	90.01	110.48	88.27	83.59	96.25	0.2035	0.47377841	0.533841532	tdh
AB5991_09340	108.54	134.56	103.94	131.72	155.09	135.42	115.68	140.74	0.2829	0.271703832	0.323519016	kbl

AB5991_09345	212.06	254.02	199.23	334.83	338.7	265.49	221.77	313.01	0.4971	0.057094145	0.076698528	miaB
AB5991_09350	1664.4	1562.65	1223.3	3401.03	3183.51	3086.2	1483.45	3223.58	1.1197	1.01E-05	2.08E-05	ymcA
AB5991_09355	181.74	205.46	178.78	12.6	20.89	21.14	188.66	18.21	-3.3730	5.55E-26	5.79E-25	cotE
AB5991_09360	5.82	7.21	4.13	23.34	28.7	19.51	5.72	23.85	2.0599	3.61E-11	1.20E-10	mutS
AB5991_09365	32.14	37.87	33.34	63.65	79.19	71.45	34.45	71.43	1.0520	4.34E-05	8.47E-05	mutL
AB5991_09370	0	0	0	0	0	0	0.00	0.00	0.0000	1	1	xtrA
AB5991_09380	0	1.33	0	0	1.48	0	0.44	0.49	0.1542	1	1	--
AB5991_09385	382.41	645.49	266.78	2193.8	1634.35	1461.8	431.56	1763.32	2.0307	4.27E-09	1.19E-08	yjdB
AB5991_09390	0.48	2.21	2.06	1.83	0.98	2.09	1.58	1.63	0.0449	1	1	--
AB5991_09405	0	0	0	0	0	0	0.00	0.00	0.0000	1	1	--
AB5991_09410	0	0	0	0	0	0	0.00	0.00	0.0000	1	1	--
AB5991_09415	94.35	90.8	59.57	357.93	335.52	301.49	81.57	331.65	2.0235	2.56E-12	9.40E-12	--
AB5991_09420	0	0	0	0.8	0	0	0.00	0.27	8.0589	1	1	--
AB5991_09425	2.35	5.4	3.46	0	0.6	0	3.74	0.20	-4.2237	1.13E-05	2.31E-05	--
AB5991_09430	0.8	0	1.72	0	0	0	0.84	0.00	-9.7142	0.264733846	0.316391271	--
AB5991_09435	5.78	1.77	5.15	1.05	0	0	4.23	0.35	-3.5964	0.000220719	0.000399669	--
AB5991_09440	2.62	2	2.33	0.24	0.22	0.24	2.32	0.23	-3.3116	0.002153634	0.003523724	--
AB5991_09445	9.14	2.49	6.15	0.37	0.69	0.73	5.93	0.60	-3.3122	3.05E-06	6.64E-06	--
AB5991_09450	1.08	0	0	0	0	0	0.36	0.00	-8.4919	1	1	--
AB5991_09460	451.87	435.24	496.8	4.74	5.65	10.73	461.30	7.04	-6.0340	6.39E-61	7.93E-59	ymzD
AB5991_09465	13.93	11.3	10.05	6.34	3.3	5.26	11.76	4.97	-1.2435	0.001412805	0.002360222	ymcC
AB5991_09470	24.39	39.4	32.37	11.19	18.25	16.86	32.05	15.43	-1.0544	0.001570169	0.002604487	pksA
AB5991_09475	189.86	196.16	249.06	37.44	47.92	48.23	211.69	44.53	-2.2491	1.45E-14	6.34E-14	ymzB
AB5991_09480	11.17	7.24	8.9	14.06	12.71	10.67	9.10	12.48	0.4552	0.176911303	0.219261793	ymaE
AB5991_09485	83825.72	73545.26	86863.96	1153.16	1600.26	2242.96	81411.65	1665.46	-5.6112	2.45E-61	3.24E-59	aprX
AB5991_09490	0.77	4.26	2.48	0	0	0	2.50	0.00	-11.2896	0.012925804	0.01911671	--

AB5991_09495	0.26	0.47	1.09	2.78	2.6	0.55	0.61	1.98	1.7041	0.04359343	0.059508254	ymaC
AB5991_09500	2.79	8.79	2.56	53.38	58.88	53.99	4.71	55.42	3.5555	1.40E-18	8.33E-18	ymaD
AB5991_09505	1.02	0.94	1.64	0	0.52	0.55	1.20	0.36	-1.7504	0.316298843	0.371274817	ebrB
AB5991_09510	2.84	0	1.22	0	0	0	1.35	0.00	-10.4023	0.025874345	0.03659082	ebrA
AB5991_09515	34.34	16.65	29.1	1.41	1.98	3.51	26.70	2.30	-3.5370	4.25E-11	1.40E-10	ymaG
AB5991_09520	3.06	1.6	3.27	2.37	0.44	0.47	2.64	1.09	-1.2736	0.097494489	0.127477976	ymaF
AB5991_09525	4.02	5.97	7.36	7.28	5.84	4.14	5.78	5.75	-0.0075	1	1	miaA
AB5991_09530	151.44	221.26	147.15	13.28	13.26	8.81	173.28	11.78	-3.8783	3.70E-26	3.91E-25	hfq
AB5991_09535	39.06	44.98	46.02	39.6	35.71	43	43.35	39.44	-0.1366	0.666744103	0.721624647	ymzC
AB5991_09540	39.91	79.02	43.51	26.38	42.21	16.09	54.15	28.23	-0.9398	0.027173932	0.03827871	ymzA
AB5991_09545	4.14	0.84	1.97	43.02	79.11	37.84	2.32	53.32	4.5246	8.97E-19	5.43E-18	nrdI
AB5991_09550	16.42	12.94	12.87	190.88	222.71	156.03	14.08	189.87	3.7537	8.06E-35	1.55E-33	nrdE
AB5991_09555	21.73	23.47	15.23	205.91	252.34	168.58	20.14	208.94	3.3747	2.51E-27	2.88E-26	nrdF
AB5991_09560	7.86	6.95	7.47	75.34	77.61	43.48	7.43	65.48	3.1402	1.18E-18	7.09E-18	ymaB
AB5991_09565	8435.43	9221.62	9089.34	121.33	158.57	235.41	8915.46	171.77	-5.6978	9.52E-62	1.35E-59	cwlC
AB5991_09570	31.15	38.7	36.11	7.1	5.32	3.63	35.32	5.35	-2.7229	1.50E-16	7.66E-16	spoVK
AB5991_09575	1.86	1.58	4.59	11.67	7.72	3.41	2.68	7.60	1.5056	0.004461427	0.006985933	hflX
AB5991_09580	2.14	4.72	2.75	14.29	11.48	8.65	3.20	11.47	1.8406	1.18E-06	2.68E-06	ynbB
AB5991_09585	507.25	673.54	574.2	57.82	48.7	32.13	585.00	46.22	-3.6619	1.06E-30	1.55E-29	glnR
AB5991_09590	1045.77	1255.71	1135.44	254.3	158.74	175.58	1145.64	196.21	-2.5457	2.82E-18	1.66E-17	glnA
AB5991_09595	44.44	52.87	47.53	3.74	6.05	6.61	48.28	5.47	-3.1427	6.79E-24	6.00E-23	xerC
AB5991_09600	0.68	1.26	1.65	0.93	0.7	0.74	1.20	0.79	-0.5991	0.433527829	0.491306795	--
AB5991_09605	3.74	4.29	6.49	0	0	0	4.84	0.00	-12.2408	4.58E-07	1.08E-06	--
AB5991_09610	0	0	0.88	0	0	0	0.29	0.00	-8.1964	1	1	--
AB5991_09615	2.13	1.3	0.76	0	0	0	1.40	0.00	-10.4478	0.000292181	0.000523106	yoqD
AB5991_09620	1.16	0	0.62	0	0	1.25	0.59	0.42	-0.5099	1	1	--

AB5991_09625	0	0	0	0	2.3	0	0.00	0.77	9.5825	0.274112878	0.325347926	--
AB5991_09630	0	1.2	0	0	0.67	0	0.40	0.22	-0.8408	1	1	--
AB5991_09635	1.63	1.79	2.44	1.42	1.66	4.23	1.95	2.44	0.3190	0.789645873	0.835736611	--
AB5991_09640	3.09	4.26	3.1	4.62	2.75	3.97	3.48	3.78	0.1179	0.769261949	0.817212198	--
AB5991_09645	4.02	1.05	1.84	3.74	7.59	6.83	2.30	6.05	1.3940	0.037152277	0.051315372	--
AB5991_09650	7.02	3.79	7.5	6.73	9.66	5.81	6.10	7.40	0.2779	0.588163875	0.645193024	--
AB5991_09655	1.5	1.37	2.32	2.93	3.96	3.8	1.73	3.56	1.0425	0.0049599	0.007723829	--
AB5991_09660	1.65	0	0.44	0.45	1.68	1.34	0.70	1.16	0.7314	0.799511049	0.844826604	--
AB5991_09665	0.67	0	0.36	1.09	0.68	1.45	0.34	1.07	1.6444	0.262212571	0.314291011	--
AB5991_09670	1.05	0	0.37	0	0	0.38	0.47	0.13	-1.9018	0.401436813	0.45873542	--
AB5991_09675	1.75	4.81	1.87	0	0	3.31	2.81	1.10	-1.3487	0.269369697	0.321028532	--
AB5991_09680	1.37	0	0	0	2.79	0	0.46	0.93	1.0261	1	1	--
AB5991_09685	3.22	4.22	3.44	0.5	0.47	0.5	3.63	0.49	-2.8878	0.001565053	0.002597085	--
AB5991_09690	0	0	1.81	0	0	0	0.60	0.00	-9.2368	0.512278474	0.569081258	--
AB5991_09695	0.37	0	1.99	0	0.38	0.81	0.79	0.40	-0.9878	0.566675501	0.623343051	--
AB5991_09700	0.83	1.91	0.78	0.45	0.21	0	1.17	0.22	-2.4150	0.001827349	0.003007788	--
AB5991_09705	0	0	0	0	0	0	0.00	0.00	0.0000	1	1	--
AB5991_09710	0.44	1.08	0.32	0.16	0.45	0	0.61	0.20	-1.5928	0.238927527	0.288660089	--
AB5991_09715	0	0	0	0.66	2.47	0.66	0.00	1.26	10.3030	0.003310941	0.005286589	--
AB5991_09720	0.9	0.82	0.32	0.33	0	0.49	0.68	0.27	-1.3149	0.201534565	0.247156812	--
AB5991_09725	1.41	0	0	0.51	0.96	0.51	0.47	0.66	0.4898	1	1	--
AB5991_09730	0	0	0.66	0	0	0	0.22	0.00	-7.7814	1	1	--
AB5991_09735	0	0	0	0	0	0	0.00	0.00	0.0000	1	1	--
AB5991_09740	0.92	0	0	0.5	0	0	0.31	0.17	-0.8797	1	1	--
AB5991_09745	0.44	0	0	0.96	0	0	0.15	0.32	1.1255	1	1	--
AB5991_09750	13.59	22.13	8.26	0.34	0	0.67	14.66	0.34	-5.4444	1.73E-14	7.52E-14	--

AB5991_09755	0.5	0	0	0	0	0.54	0.17	0.18	0.1110	1	1	--
AB5991_09760	0	0	0	0	0	0	0.00	0.00	0.0000	1	1	--
AB5991_09765	0.92	1.33	1.4	0.47	0.93	0.42	1.22	0.61	-1.0040	0.03307982	0.046010496	xkdO
AB5991_09770	0.43	0.8	0.7	0	0.22	0.23	0.64	0.15	-2.1006	0.207154697	0.25373575	--
AB5991_09775	0.77	0.89	0.52	0.53	0.99	0.73	0.73	0.75	0.0456	1	1	--
AB5991_09780	7.52	2.34	6.86	0.87	1.05	0.37	5.57	0.76	-2.8682	1.34E-07	3.28E-07	--
AB5991_09785	3.28	1.06	3.37	1.37	3.03	3.31	2.57	2.57	0.0000	1	1	xkdV
AB5991_09790	1.4	3.32	0.96	0	0.69	1.3	1.89	0.66	-1.5131	0.271309525	0.323146408	yomQ
AB5991_09795	0	0	0	0	0	0	0.00	0.00	0.0000	1	1	--
AB5991_09800	0	0	0	0	0.86	0	0.00	0.29	8.1632	1	1	bhlA
AB5991_09805	0.68	0	0	0	0	0	0.23	0.00	-7.8244	1	1	xhlB
AB5991_09810	2.96	8.14	4.55	1.41	2.82	0.6	5.22	1.61	-1.6961	0.003768125	0.005956698	xlyB
AB5991_09815	2.33	2.14	4.16	2.54	2.37	0.42	2.88	1.78	-0.6952	0.382361731	0.43959422	yobK
AB5991_09820	2.46	3.57	2.74	2.23	1.25	0.67	2.92	1.38	-1.0795	0.022962358	0.032811631	yobL
AB5991_09830	12.48	14.67	12.28	7.6	6.61	2.52	13.14	5.58	-1.2369	0.0013044	0.002196681	--
AB5991_09835	17.39	17.11	18.6	6.08	2.53	0	17.70	2.87	-2.6246	2.00E-06	4.42E-06	ynxB
AB5991_09850	0.72	2.63	0	0	0	0	1.12	0.00	-10.1250	0.143057405	0.180629875	ynzG
AB5991_09855	0.83	0.76	0.44	3.62	1.69	0	0.68	1.77	1.3872	0.21129175	0.258245472	ynaB
AB5991_09865	12.07	12.39	12.65	3.28	5.41	5.33	12.37	4.67	-1.4043	6.15E-05	0.000117496	tnpB
AB5991_09870	5.22	5.43	5.74	1.95	2.3	2.06	5.46	2.10	-1.3771	5.93E-05	0.000113814	tnpA
AB5991_09880	0.96	0.44	1.28	1.83	1.71	0.78	0.89	1.44	0.6888	0.397098164	0.454169588	--
AB5991_09885	0	1.74	0.51	0.52	0.48	0	0.75	0.33	-1.1699	0.706237646	0.757964782	--
AB5991_09890	0	0	1.67	0	3.19	0.85	0.56	1.35	1.2745	0.501270257	0.56134918	yopB
AB5991_09910	2.19	0	1.17	0	0	0	1.12	0.00	-10.1293	0.264723671	0.316391271	--
AB5991_09915	1.15	2.82	1.64	2.5	0.39	0.83	1.87	1.24	-0.5927	0.575793925	0.632681461	ynaI
AB5991_09925	12.86	19.31	14.16	0.85	0.93	1.12	15.44	0.97	-3.9978	2.56E-23	2.16E-22	ynaJ

AB5991_09930	109.89	83.49	118.12	2.68	2.7	3.08	103.83	2.82	-5.2024	5.01E-35	9.92E-34	--
AB5991_09935	17.91	34.55	13.2	2.47	3.7	4.28	21.89	3.48	-2.6515	1.94E-09	5.57E-09	--
AB5991_09940	15.34	15.54	8.57	4.05	6.55	4.89	13.15	5.16	-1.3487	0.000125929	0.000233457	xynB
AB5991_09945	44.91	78.16	50.37	10.89	4.78	6.44	57.81	7.37	-2.9717	5.54E-16	2.68E-15	xylR
AB5991_09950	524.15	558.11	441.48	8.67	14.02	12.72	507.91	11.80	-5.4273	3.27E-59	3.51E-57	xylA
AB5991_09955	21.81	27.88	18.43	2.62	2.21	1.57	22.71	2.13	-3.4119	8.97E-22	6.82E-21	xylB
AB5991_09965	59.31	62.66	52.41	44.95	47.02	43.51	58.13	45.16	-0.3642	0.156124494	0.195327778	NGR_a01970
AB5991_09970	0.57	1.57	0.91	2.16	2.89	2.46	1.02	2.50	1.3000	0.042613037	0.058310259	yncB
AB5991_09975	12.89	11.02	9.97	9.44	8.31	5.8	11.29	7.85	-0.5247	0.097534722	0.127488604	yncC
AB5991_09980	71.38	63.86	64.11	16.42	15.68	15.36	66.45	15.82	-2.0705	9.23E-14	3.77E-13	alr2
AB5991_09985	64.49	66.22	59.89	110.75	90.68	79	63.53	93.48	0.5571	0.038352797	0.052808238	--
AB5991_09995	0	0.76	0	3.62	1.27	1.35	0.25	2.08	3.0375	0.019647043	0.02837033	yncF
AB5991_10000	201.97	568.24	718.99	64.78	121.94	234.94	496.40	140.55	-1.8204	0.000170628	0.000311687	--
AB5991_10005	0	0	0	0	1.11	0	0.00	0.37	8.5314	1	1	--
AB5991_10010	2.58	3.16	2.76	5.38	4.38	3.03	2.83	4.26	0.5895	0.172195039	0.213616527	thyA
AB5991_10015	4.39	9.22	5.37	0	0	0	6.33	0.00	-12.6272	1.69E-06	3.77E-06	--
AB5991_10020	279.41	280.26	264.97	65.8	49.31	50	274.88	55.04	-2.3203	1.33E-17	7.37E-17	yncM
AB5991_10025	15.7	10.23	11.37	0	0	0	12.43	0.00	-13.6019	6.14E-14	2.54E-13	ynzK
AB5991_10030	4038.53	6073.09	9493.51	74.33	114.4	197.61	6535.04	128.78	-5.6652	1.35E-34	2.57E-33	--
AB5991_10035	4.78	14.05	8.18	61.37	52.56	34.16	9.00	49.36	2.4549	6.17E-08	1.56E-07	tatAc
AB5991_10045	28.09	44.09	33.43	1.48	2.77	4.9	35.20	3.05	-3.5288	1.64E-15	7.64E-15	yndA
AB5991_10050	1.46	2.49	2.28	9.07	7.18	5.16	2.08	7.14	1.7810	8.73E-06	1.82E-05	--
AB5991_10055	186.57	289.93	219.95	144.61	106.15	87.71	232.15	112.82	-1.0410	0.000484031	0.000850482	yndB
AB5991_10060	58.34	61.46	84.89	1.04	4.87	4.14	68.23	3.35	-4.3482	1.33E-19	8.64E-19	ynzB
AB5991_10075	1.57	1.65	1.2	11.45	12.99	8.97	1.47	11.14	2.9182	2.47E-11	8.34E-11	yndG
AB5991_10080	0.57	0.52	1.83	4.04	6.97	6.49	0.97	5.83	2.5833	8.65E-06	1.81E-05	yndH

AB5991_10085	2.2	2.63	2	3.71	5.38	2.38	2.28	3.82	0.7479	0.069194123	0.092297569	yndJ
AB5991_10095	13.57	27.55	11.97	1.55	1.45	1.55	17.70	1.52	-3.5445	1.09E-12	4.13E-12	yndL
AB5991_10100	3.61	3.69	2.15	0.44	0.41	0.43	3.15	0.43	-2.8842	0.001827699	0.003007788	yndM
AB5991_10105	2.58	4.74	0.92	4.68	2.19	6.99	2.75	4.62	0.7502	0.245140714	0.295407908	fosB
AB5991_10115	467.08	653.58	544.54	58.53	50.6	33.88	555.07	47.67	-3.5415	6.30E-29	7.97E-28	lexA
AB5991_10120	3.41	1.04	1.22	1.85	0.58	0	1.89	0.81	-1.2224	0.329909751	0.385655467	yneA
AB5991_10125	1.38	2.03	2.07	5.71	3.38	2.69	1.83	3.93	1.1041	0.04150507	0.056892176	yneB
AB5991_10130	13.9	35.46	21.48	3.36	0.79	1.67	23.61	1.94	-3.6055	1.79E-08	4.74E-08	ynzC
AB5991_10135	36.8	37.1	44.47	196.58	191.21	174.67	39.46	187.49	2.2484	5.48E-17	2.88E-16	tkt
AB5991_10140	0	0	0	0	2.06	1.75	0.00	1.27	10.3106	0.016059679	0.023454573	sirA
AB5991_10145	0.83	1.52	1.77	2.69	0.84	2.68	1.37	2.07	0.5919	0.578881039	0.6355368	yneF
AB5991_10150	11.43	19.07	14.44	2.26	1.06	0	14.98	1.11	-3.7587	5.40E-07	1.27E-06	ynzD
AB5991_10155	1.79	2.81	2.46	31.38	32.22	18.52	2.35	27.37	3.5400	2.79E-17	1.50E-16	ccdA
AB5991_10160	0	0.91	3.19	5.96	6.59	3.77	1.37	5.44	1.9929	0.008735639	0.013169789	ccdB
AB5991_10165	8.82	17.54	16.11	43.55	33.65	29.83	14.16	35.68	1.3335	0.00011479	0.000213804	ccdC
AB5991_10170	33.28	37.91	36.05	40.78	33.45	28.73	35.75	34.32	-0.0588	0.872485827	0.911987686	yneK
AB5991_10175	213.86	166.37	253.3	31.51	36.98	39.33	211.18	35.94	-2.5548	6.94E-17	3.64E-16	cotM
AB5991_10180	23.36	51.93	31.56	0	1.25	0	35.62	0.42	-6.4175	4.31E-12	1.56E-11	sspP
AB5991_10185	1.23	6.77	9.2	0	0	1.33	5.73	0.44	-3.6929	0.011266375	0.01671228	sspO
AB5991_10190	1397.99	811.98	1324.81	1206.85	1437.51	940.61	1178.26	1194.99	0.0203	0.943875201	0.980159107	citB
AB5991_10195	150.45	120.33	120.95	259.43	322.38	236.09	130.58	272.63	1.0621	6.96E-05	0.00013246	yneN
AB5991_10200	1.43	0	0	0	0	0	0.48	0.00	-8.8968	1	1	ynzL
AB5991_10205	118.04	108.37	118.34	5.35	15.02	19.97	114.92	13.45	-3.0953	2.01E-15	9.28E-15	sspN
AB5991_10210	205.14	179.12	157.24	18.72	24.09	36.49	180.50	26.43	-2.7716	1.42E-17	7.81E-17	tlp
AB5991_10215	0	0	0.46	0	0.44	0	0.15	0.15	-0.0641	1	1	yneP
AB5991_10220	0	2.21	0.64	0	0	0	0.95	0.00	-9.8918	0.143025205	0.180629875	yneQ

AB5991_10225	4.39	18.44	9.4	17.06	15.97	9.51	10.74	14.18	0.4004	0.431151505	0.488892811	yneR
AB5991_10230	1.55	7.98	7.64	9.8	13.28	11.77	5.72	11.62	1.0213	0.03410488	0.047320223	plsY
AB5991_10235	28.8	51.25	24.64	102.63	127.6	93.99	34.90	108.07	1.6308	3.79E-07	8.98E-07	yneT
AB5991_10240	5.24	8.6	7.86	31.56	30.19	15.21	7.23	25.65	1.8264	1.52E-07	3.70E-07	parE
AB5991_10245	23.52	17.27	20.2	51.07	49.62	32.33	20.33	44.34	1.1250	8.97E-05	0.000169181	parC
AB5991_10250	22.95	26.34	33.75	43.68	48.18	26.4	27.68	39.42	0.5101	0.229699731	0.278175551	--
AB5991_10255	137.35	170.63	155.62	185.47	151.74	167.47	154.53	168.23	0.1225	0.649131646	0.703905452	ynfC
AB5991_10260	17.2	21.84	19.63	2.81	3.03	0.98	19.56	2.27	-3.1048	2.50E-16	1.25E-15	alsT
AB5991_10265	72.54	80.76	69.2	728.92	953.52	764.91	74.17	815.78	3.4593	2.70E-34	5.02E-33	eglS
AB5991_10270	0.68	2.51	2.93	2.98	2.09	1.48	2.04	2.18	0.0980	1	1	ynfE
AB5991_10275	69.94	68	70.37	30.83	44.65	39.47	69.44	38.32	-0.8577	0.001266044	0.002137526	xynC
AB5991_10285	1.21	0	1.3	0.44	0	0.88	0.84	0.44	-0.9271	0.545208667	0.602567108	yngA
AB5991_10290	33.16	35.27	33.95	12.75	12.14	9.19	34.13	11.36	-1.5869	2.46E-08	6.43E-08	yngB
AB5991_10295	39.96	46.7	44.03	24.04	16.02	10.16	43.56	16.74	-1.3798	4.13E-05	8.08E-05	yngC
AB5991_10300	43.68	83.25	51.87	3.77	4.45	4.4	59.60	4.21	-3.8246	1.60E-26	1.74E-25	nrnB
AB5991_10305	104.97	264.69	125.97	20.22	24.91	17.2	165.21	20.78	-2.9913	2.09E-15	9.68E-15	yngE
AB5991_10310	81.95	173.79	100.23	10.54	10.57	14.99	118.66	12.03	-3.3017	6.17E-19	3.78E-18	yngF
AB5991_10315	142.59	262.93	179.76	9.17	12.26	10	195.09	10.48	-4.2189	1.95E-34	3.67E-33	yngG
AB5991_10320	43.97	110.63	65.3	3.54	2.49	5.29	73.30	3.77	-4.2799	2.06E-16	1.04E-15	yngHB
AB5991_10325	116.3	251.84	147.83	12.96	12.95	14.8	171.99	13.57	-3.6638	1.76E-25	1.78E-24	accC2
AB5991_10330	157.74	242.98	183.57	11.32	14.05	14.82	194.76	13.40	-3.8618	4.34E-35	8.70E-34	yngI
AB5991_10335	59.78	108.6	75.42	8.08	5.47	5.99	81.27	6.51	-3.6412	9.64E-25	9.16E-24	yngJ
AB5991_10340	0.59	3.25	0.63	0	0	0	1.49	0.00	-10.5411	0.043576512	0.059508254	ynzE
AB5991_10350	91.97	82.7	88.51	11.93	19.8	29.87	87.73	20.53	-2.0950	2.70E-10	8.30E-10	yngK
AB5991_10355	4.14	8.45	3.93	0.5	0.94	0.5	5.51	0.65	-3.0901	7.61E-05	0.000144184	yngL
AB5991_10370	37.59	45.42	32.35	190.99	242.54	242.19	38.45	225.24	2.5503	2.60E-19	1.65E-18	dacC

AB5991_10375	12.2	11.2	14.82	78.79	85.77	87.63	12.74	84.06	2.7221	3.37E-20	2.31E-19	galM
AB5991_10380	8.57	6.91	6.8	45.05	35.95	39.78	7.43	40.26	2.4386	4.26E-17	2.26E-16	yoeA
AB5991_10385	3253.15	3013.15	3716.83	757.16	763.46	701.3	3327.71	740.64	-2.1677	2.28E-17	1.23E-16	yoeB
AB5991_10390	0.45	0.62	0.61	1.39	0.97	0.74	0.56	1.03	0.8838	0.222963944	0.270880578	insK
AB5991_10400	48.66	119.14	52.39	4.32	1.68	7.17	73.40	4.39	-4.0634	3.73E-16	1.83E-15	yoeC
AB5991_10405	10.95	27.3	5.86	0.85	1.59	0	14.70	0.81	-4.1762	3.74E-06	8.10E-06	yoeD
AB5991_10410	1063.49	1434.05	976.11	7545.74	8215.06	8102.75	1157.88	7954.52	2.7803	2.21E-25	2.23E-24	ggt
AB5991_10415	66.15	84.71	61.73	24.06	22.08	13.23	70.86	19.79	-1.8403	3.96E-09	1.11E-08	yofA
AB5991_10420	4.93	4.36	4.69	11.91	15.05	15.61	4.66	14.19	1.6065	8.77E-07	2.01E-06	yogA
AB5991_10425	9.76	21.72	6.52	86.62	67.03	67.33	12.67	73.66	2.5398	8.70E-11	2.78E-10	gltB
AB5991_10430	25.55	58.19	18.89	131.57	68.86	68.91	34.21	89.78	1.3920	0.000702275	0.001214077	gltA
AB5991_10435	29.02	37.86	31.89	37.66	24.65	12.57	32.92	24.96	-0.3995	0.274141063	0.325347926	gltC
AB5991_10460	33.8	44.07	41.91	1.6	1.99	1.06	39.93	1.55	-4.6870	3.87E-24	3.49E-23	rtp
AB5991_10465	7.56	7.87	8.09	12.89	13.85	12.01	7.84	12.92	0.7203	0.022403708	0.032071062	yoxD
AB5991_10470	15.21	32.22	16.26	173.68	143.48	193.76	21.23	170.31	3.0040	6.86E-19	4.19E-18	yoxC
AB5991_10475	4.22	10.76	6.02	85.41	61.8	102.02	7.00	83.08	3.5690	1.19E-21	9.00E-21	yoxB
AB5991_10480	4.4	10.57	10.5	51.91	43.06	47.63	8.49	47.53	2.4851	3.81E-12	1.38E-11	yoaA
AB5991_10485	0.36	1.34	0.78	0	0.74	0	0.83	0.25	-1.7447	0.195090665	0.240144151	--
AB5991_10490	0.24	0.44	0.52	0.26	0	0.52	0.40	0.26	-0.6215	1	1	--
AB5991_10495	1.15	2.12	0.62	0.21	0.39	0.21	1.30	0.27	-2.2638	0.013482896	0.019896166	lnrL
AB5991_10500	0.33	0	0	1.07	0.33	0.71	0.11	0.70	2.6767	0.147138173	0.185193562	--
AB5991_10505	0.44	1.6	1.09	0.63	0.3	0.47	1.04	0.47	-1.1607	0.138061982	0.175325913	yoaB
AB5991_10510	59.31	62.66	52.41	44.95	47.02	43.51	58.13	45.16	-0.3642	0.156061064	0.195309954	NGR_a01970
AB5991_10515	13.39	11.17	11.06	0	0	0	11.87	0.00	-13.5354	3.37E-12	1.22E-11	--
AB5991_10520	3.65	0	1.3	0	0	0	1.65	0.00	-10.6883	0.028089043	0.039483748	--
AB5991_10525	0.45	0.62	0.61	1.39	0.97	0.74	0.56	1.03	0.8838	0.223165684	0.270880578	insK

AB5991_10530	5.27	15.21	7.25	3.28	0.77	0	9.24	1.35	-2.7755	0.000603757	0.001051543	--
AB5991_10535	11.59	38.3	29.74	6.72	7.08	4.18	26.54	5.99	-2.1469	0.000110376	0.00020597	--
AB5991_10540	59.31	62.66	52.41	44.95	47.02	43.51	58.13	45.16	-0.3642	0.155804704	0.195112103	NGR_a01970
AB5991_10545	0	0	0	1.37	0	0	0.00	0.46	8.8350	1	1	yopG
AB5991_10555	0	0	0	0	0	0	0.00	0.00	0.0000	1	1	yopE
AB5991_10560	10.13	12.32	10.69	10.05	11.98	12.33	11.05	11.45	0.0522	0.859423858	0.900230056	flp
AB5991_10565	46.95	63.22	63.59	17.02	13.54	3.39	57.92	11.32	-2.3556	4.74E-08	1.21E-07	yoyI
AB5991_10570	0.46	1.69	0.49	0.5	0.47	0.5	0.88	0.49	-0.8447	0.742987665	0.79269318	yopD
AB5991_10575	0	0	0	0	0	0	0.00	0.00	0.0000	1	1	--
AB5991_10580	0	0	0	0	0	0	0.00	0.00	0.0000	1	1	ynaF
AB5991_10585	1.4	0	1.2	1.22	1.14	1.21	0.87	1.19	0.4574	0.689485252	0.743401014	ynaE
AB5991_10590	16.76	15.96	15.09	0.48	0.23	0.39	15.94	0.37	-5.4417	1.16E-30	1.68E-29	--
AB5991_10595	14.51	15.06	11.13	5.83	3.37	1.54	13.57	3.58	-1.9220	2.55E-06	5.59E-06	ttuC
AB5991_10600	1.74	0.8	0.7	2.37	0.66	0.47	1.08	1.17	0.1114	1	1	menG
AB5991_10605	0.15	0.81	0.16	1.28	0.6	0.32	0.37	0.73	0.9740	0.260229086	0.312479499	yfmI
AB5991_10610	59.31	62.66	52.41	44.95	47.02	43.51	58.13	45.16	-0.3642	0.156896854	0.196170469	NGR_a01970
AB5991_10630	9.54	15.67	9.66	6.01	3.58	2.72	11.62	4.10	-1.5022	0.002374494	0.003867561	yolB
AB5991_10635	0	0	0	0	0	0	0.00	0.00	0.0000	1	1	--
AB5991_10640	0.68	0.62	0.36	3.31	10.68	6.23	0.55	6.74	3.6065	3.94E-06	8.49E-06	yokL
AB5991_10645	0	0	0	1.42	4.31	5.29	0.00	3.67	11.8429	1.19E-06	2.70E-06	yokK
AB5991_10650	0	1.45	0.42	3.85	3.61	5.12	0.62	4.19	2.7500	0.000216695	0.000392921	--
AB5991_10655	0	0	0.42	4.28	3.21	0.85	0.14	2.78	4.3116	0.000650379	0.001128289	yobK
AB5991_10660	0.3	1.47	0.43	5.89	4.49	3.58	0.73	4.65	2.6657	5.16E-08	1.31E-07	yobL
AB5991_10665	4.86	12.49	5.89	8.81	9.89	6.31	7.75	8.34	0.1059	0.793934711	0.839711105	yobM
AB5991_10670	55.72	45.96	49.1	1.09	1.15	0.82	50.26	1.02	-5.6228	2.82E-51	1.78E-49	yobN
AB5991_10675	24.43	24.92	17.99	13.58	13.26	10.58	22.45	12.47	-0.8477	0.015765226	0.023058458	csaA

AB5991_10680	4.23	9.14	3.19	6.23	2.28	1.89	5.52	3.47	-0.6711	0.284863452	0.33746801	yobQ
AB5991_10685	0.49	1.78	0.52	3.7	3.71	3.94	0.93	3.78	2.0244	0.000340003	0.000606265	yobR
AB5991_10690	0	0	1.34	0.34	0.32	0.68	0.45	0.45	0.0000	1	1	yobS
AB5991_10695	0	1.42	0	1.96	1.05	0	0.47	1.00	1.0839	0.34933435	0.405972112	yobT
AB5991_10700	0.37	0.69	0.8	4.88	5.33	6.08	0.62	5.43	3.1306	7.93E-06	1.66E-05	yobU
AB5991_10705	0	1.41	0.62	4.19	4.7	2.08	0.68	3.66	2.4340	0.00014244	0.000262594	yobV
AB5991_10710	6357.01	5063.45	7580.23	75.25	102.76	154.81	6333.56	110.94	-5.8352	7.94E-56	6.18E-54	csk22
AB5991_10715	35.15	53.27	34.01	30.94	18.74	6.04	40.81	18.57	-1.1357	0.015256586	0.022347437	czrA
AB5991_10720	303.11	244.27	293.93	183.53	148.14	108.51	280.44	146.73	-0.9345	0.000861682	0.001475523	yocA
AB5991_10725	27.26	61.8	32.76	28.92	31.17	32.43	40.61	30.84	-0.3969	0.263087682	0.315244776	yozB
AB5991_10735	17.42	15.99	22.9	1.18	3.32	0.79	18.77	1.76	-3.4121	8.94E-13	3.42E-12	yocC
AB5991_10740	119.76	139.81	136.77	41	24.45	16.21	132.11	27.22	-2.2790	1.94E-11	6.62E-11	yocD
AB5991_10745	48.47	62.37	48.37	348.43	93.46	42.49	53.07	161.46	1.6052	0.003460154	0.005500509	des
AB5991_10750	25.33	33.7	34.73	9.89	5.29	4.04	31.25	6.41	-2.2864	1.44E-10	4.54E-10	desK
AB5991_10755	61.76	70.25	51.22	23.92	12.57	7.5	61.08	14.66	-2.0584	6.81E-08	1.71E-07	desR
AB5991_10760	603.54	569.67	668.92	277.58	526.11	372.07	614.04	391.92	-0.6478	0.024954247	0.035388713	yocH
AB5991_10765	13.94	14.58	12.3	12.62	11.91	6.94	13.61	10.49	-0.3753	0.225716714	0.273769417	recQ
AB5991_10775	77.83	104.28	86.01	6.27	5.87	7.18	89.37	6.44	-3.7947	2.88E-32	4.51E-31	azoR1
AB5991_10780	45.92	81.62	42.04	326.04	419.52	388.14	56.53	377.90	2.7410	1.10E-17	6.16E-17	yocK
AB5991_10785	1.09	3.99	4.06	0	0.55	0	3.05	0.18	-4.0547	0.003688789	0.005845244	yocL
AB5991_10790	32.5	24.75	27.98	1.72	3.23	3.43	28.41	2.79	-3.3463	1.99E-11	6.77E-11	yoyB
AB5991_10795	3.41	8.35	2.84	0.82	0	0	4.87	0.27	-4.1542	4.17E-05	8.15E-05	yocM
AB5991_10800	366.98	691.44	530.83	75.95	66.2	65.96	529.75	69.37	-2.9329	3.08E-20	2.12E-19	yozN
AB5991_10805	372.31	694.98	499.76	45.36	57.39	47.66	522.35	50.14	-3.3811	2.67E-24	2.44E-23	yocN
AB5991_10810	60.77	143.34	73.96	14.24	11.73	6.24	92.69	10.74	-3.1099	7.20E-13	2.78E-12	yozO
AB5991_10815	20.38	48.81	54.01	4.82	0.9	2.88	41.07	2.87	-3.8405	2.75E-10	8.43E-10	yozC

AB5991_10820	2636.9	4065.88	2038.55	16008.53	16001.46	16276.18	2913.78	16095.39	2.4657	9.61E-17	4.97E-16	dhaS
AB5991_10825	84.24	133.53	100.67	140.27	156.45	184.22	106.15	160.31	0.5948	0.033517569	0.046570422	sqhC
AB5991_10830	156.78	264.08	176.9	136.46	147.04	163.64	199.25	149.05	-0.4188	0.136995428	0.174305942	sodF
AB5991_10835	19.05	18.36	16.9	2.5	2.06	3.22	18.10	2.59	-2.8034	5.57E-17	2.92E-16	yocR
AB5991_10840	59.88	58.41	67.23	10.58	7.62	6.08	61.84	8.09	-2.9337	1.31E-21	9.84E-21	yocS
AB5991_10845	286.98	323.69	227.98	1360.08	1970.3	1927.28	279.55	1752.55	2.6483	2.18E-20	1.51E-19	odhB
AB5991_10850	131.91	163.08	121.29	449.61	631.95	602.51	138.76	561.36	2.0163	2.41E-13	9.59E-13	odhA
AB5991_10855	6.98	7.27	8.07	18.66	19.38	12.66	7.44	16.90	1.1836	5.64E-05	0.000108479	yojO
AB5991_10860	1.19	2.54	2.75	7.52	6.63	1.92	2.16	5.36	1.3103	0.023699297	0.03377958	yojN
AB5991_10865	6.42	23.59	12.43	37.92	32.99	23.84	14.15	31.58	1.1587	0.005231489	0.00812446	yojM
AB5991_10870	3.19	4	2.64	61.74	77.72	34.26	3.28	57.91	4.1434	3.00E-25	2.96E-24	cwlS
AB5991_10875	9.79	9.54	8.09	56.81	91.22	56.71	9.14	68.25	2.9005	1.20E-18	7.21E-18	yojK
AB5991_10880	3.48	7.45	3.41	22.05	20.93	15.68	4.78	19.55	2.0323	1.29E-07	3.17E-07	cdaS
AB5991_10885	156.81	141.4	166.84	13.45	13.67	13.1	155.02	13.41	-3.5314	1.77E-35	3.70E-34	norM
AB5991_10890	230.35	211.88	234.96	14.36	13.22	11.75	225.73	13.11	-4.1059	1.10E-43	3.77E-42	rsbRC
AB5991_10895	1.46	2.49	2.28	9.07	7.18	5.16	2.08	7.14	1.7810	8.71E-06	1.82E-05	--
AB5991_10900	763.98	1057.46	835.87	58.44	57.45	33.79	885.77	49.89	-4.1500	2.24E-35	4.63E-34	bshB2
AB5991_10905	1.54	0	2.2	13.44	13.1	3.9	1.25	10.15	3.0249	6.43E-05	0.000122752	yojF
AB5991_10910	0	3.57	0.69	10.57	13.85	2.81	1.42	9.08	2.6763	0.002047683	0.003355901	yoyC
AB5991_10915	1.01	1.11	1.29	8.99	8.82	5.67	1.14	7.83	2.7836	2.75E-09	7.79E-09	yojE
AB5991_10920	636.05	457.92	577.43	59.31	94.7	167.59	557.13	107.20	-2.3777	6.42E-11	2.09E-10	gerT
AB5991_10925	33.56	14	58.72	5.81	11.64	10.73	35.43	9.39	-1.9151	0.000295803	0.000529114	yojB
AB5991_10935	12.98	7.66	18.34	0	0	2.01	12.99	0.67	-4.2775	6.84E-10	2.03E-09	yolI
AB5991_10940	39.46	81.26	66.71	3.48	6.51	6.93	62.48	5.64	-3.4696	5.61E-16	2.71E-15	yodB
AB5991_10945	546.7	592.93	548.14	65.53	67.66	74.86	562.59	69.35	-3.0201	2.01E-28	2.46E-27	yodC
AB5991_10950	0.45	0.62	0.61	1.39	0.97	0.74	0.56	1.03	0.8838	0.223184483	0.270880578	insK

AB5991_10955	12.29	8.81	13.14	6.52	13.12	12.65	11.41	10.76	-0.0846	0.80945212	0.853061138	mhqD
AB5991_10965	0.9	0	0.96	0	0	0	0.62	0.00	-9.2761	0.509623765	0.568309168	yoyD
AB5991_10970	44.37	42.96	51.6	9.49	7.65	7.35	46.31	8.16	-2.5041	4.11E-18	2.39E-17	yodF
AB5991_10975	16.9	21.08	16.14	68.33	63.16	39.1	18.04	56.86	1.6563	1.01E-07	2.50E-07	ctpA
AB5991_10980	252.33	234.97	291.32	15.4	32.76	50.73	259.54	32.96	-2.9770	4.57E-15	2.07E-14	yodH
AB5991_10985	832.02	1199.81	897.44	124.81	166.44	225.17	976.42	172.14	-2.5039	7.41E-16	3.53E-15	yodI
AB5991_10990	11.65	18.17	12.23	20.57	24.62	15	14.02	20.06	0.5174	0.110719335	0.143541129	yodJ
AB5991_10995	9.27	11.82	6.33	12.6	16.25	6.41	9.14	11.75	0.3628	0.378728165	0.436047997	deoD
AB5991_11000	21.52	28.97	16.87	1.56	0	0	22.45	0.52	-5.4323	2.35E-08	6.16E-08	yoyE
AB5991_11005	79.19	101.15	77.32	116.08	89.94	65.22	85.89	90.41	0.0741	0.806079648	0.850409745	yodL
AB5991_11010	23.04	36.33	32.22	11.88	12.62	9.91	30.53	11.47	-1.4124	1.08E-05	2.22E-05	pgpB
AB5991_11015	33.7	63.75	45.87	0	1.04	0	47.77	0.35	-7.1065	2.49E-19	1.58E-18	yozD
AB5991_11020	0	4.61	8.05	0	0	0	4.22	0.00	-12.0430	0.010899663	0.016186448	yoyF
AB5991_11025	625.32	847.03	598.05	16.17	16.21	18.96	690.13	17.11	-5.3337	3.20E-57	2.76E-55	yodN
AB5991_11030	132.55	187.33	175.25	34.07	41.7	16.52	165.04	30.76	-2.4236	6.87E-12	2.43E-11	yozE
AB5991_11035	154.84	206.99	166.97	12.68	11.87	7.71	176.27	10.75	-4.0349	1.27E-31	1.93E-30	yokU
AB5991_11040	174.37	268.84	205.58	19.71	20.92	24.46	216.26	21.70	-3.3172	2.38E-28	2.90E-27	kamA
AB5991_11045	65.71	110.23	67	9.73	10	10.16	80.98	9.96	-3.0229	1.85E-20	1.30E-19	yodP
AB5991_11050	62.04	114.43	58.24	9.6	11.09	8.66	78.24	9.78	-2.9994	1.49E-17	8.18E-17	yodQ
AB5991_11055	16.58	30.45	13.89	1.8	2.53	0.9	20.31	1.74	-3.5420	1.60E-12	5.97E-12	yodR
AB5991_11060	40.6	65.42	36.98	2.85	2.13	2.27	47.67	2.42	-4.3019	2.64E-25	2.61E-24	yodS
AB5991_11065	18.14	32.07	18.68	1.33	1.79	0.59	22.96	1.24	-4.2148	6.06E-22	4.74E-21	yodT
AB5991_11070	0	0	0	0	0	0	0.00	0.00	0.0000	1	1	--
AB5991_11075	31.71	8.73	52.56	0	4.84	6.87	31.00	3.90	-2.9895	8.86E-05	0.000167457	--
AB5991_11080	343.19	433.16	346.44	18.9	29.72	30.6	374.26	26.41	-3.8251	2.98E-34	5.50E-33	cgeE
AB5991_11085	495.83	563.77	492.06	24.86	34.18	46.28	517.22	35.11	-3.8810	5.05E-34	9.20E-33	cgeD

AB5991_11090	2739	3728.88	3686.48	182.45	212.82	265.36	3384.79	220.21	-3.9421	3.12E-38	7.66E-37	cgeC
AB5991_11100	100948.22	81484.6	110078.99	2145.9	3267.1	4974.6	97503.94	3462.53	-4.8156	1.13E-40	3.27E-39	cgeB
AB5991_11105	6.45	12.71	7.07	822.77	1062.77	755.06	8.74	880.20	6.6535	2.54E-67	5.61E-65	phy
AB5991_11110	13615.03	15583.31	13463.12	341.24	485.72	714.56	14220.49	513.84	-4.7905	6.33E-46	2.57E-44	MJ1061
AB5991_11115	49.45	128.18	70.65	67.33	69.35	62.97	82.76	66.55	-0.3145	0.368820868	0.426371955	msrB
AB5991_11120	41.97	82.04	44.16	51.54	54.09	48	56.06	51.21	-0.1305	0.698210404	0.751584038	msrA
AB5991_11125	1714.15	1805.14	1836.29	28.61	23.32	39.04	1785.19	30.32	-5.8795	6.94E-68	1.62E-65	ypoP
AB5991_11130	4.05	3.22	2.89	5.58	3.99	3.95	3.39	4.51	0.4122	0.288323813	0.341242534	ypnP
AB5991_11135	3.71	1.7	4.96	10.08	11.32	3.01	3.46	8.14	1.2351	0.098654214	0.128909471	ypmT
AB5991_11140	7.69	8.83	10.28	22.31	24.79	26.02	8.93	24.37	1.4480	5.80E-06	1.23E-05	ypmS
AB5991_11145	11.3	14.26	10.32	39.93	34.97	28.79	11.96	34.56	1.5310	4.66E-07	1.10E-06	ypmR
AB5991_11150	3.2	9.09	4.05	35.77	31.99	29.62	5.45	32.46	2.5752	5.38E-12	1.92E-11	ypmQ
AB5991_11155	40.17	57.95	54.46	119.35	73	50.47	50.86	80.94	0.6703	0.064844066	0.086698918	ypmP
AB5991_11160	16.52	20.92	16.76	54.37	35.52	30.07	18.07	39.99	1.1462	0.000167695	0.000306592	ilvA
AB5991_11165	203.62	189.94	251.32	58.03	31.21	46.36	214.96	45.20	-2.2497	1.27E-13	5.11E-13	yplP
AB5991_11170	120.22	116.32	154.45	163.82	67.34	54.86	130.33	95.34	-0.4510	0.232760015	0.281538233	yplQ
AB5991_11175	32.02	37.95	32.68	50.97	46.8	33.4	34.22	43.72	0.3537	0.215828785	0.263466371	ypkP
AB5991_11180	4.28	1.96	3.81	25.59	29.03	15.82	3.35	23.48	2.8092	1.18E-10	3.76E-10	dfrA
AB5991_11185	4.4	15.54	11.22	154.25	144	79.88	10.39	126.04	3.6011	1.21E-17	6.75E-17	ypjQ
AB5991_11190	5.91	11.39	13.9	106.32	74.55	55.63	10.40	78.83	2.9222	9.24E-15	4.09E-14	ypjP
AB5991_11195	16.58	39.02	19.23	42.41	39.22	26.54	24.94	36.06	0.5316	0.134919242	0.172105465	ypiP
AB5991_11200	67.31	74.01	71.54	179.41	165.35	174.97	70.95	173.24	1.2879	1.46E-06	3.28E-06	yphP
AB5991_11205	862.25	1148.85	911.49	58.61	40.04	48.77	974.20	49.14	-4.3092	8.61E-44	2.97E-42	ilvD
AB5991_11210	46.7	65.56	38.18	20.46	25.31	20.01	50.15	21.93	-1.1935	6.78E-05	0.000129259	ypgR
AB5991_11215	18.72	23.09	10.32	7	5.66	6.33	17.38	6.33	-1.4569	0.000263461	0.000474039	ypgQ
AB5991_11220	6.36	10.99	3.2	0.81	0.38	0	6.85	0.40	-4.1101	2.41E-06	5.30E-06	bsaA

AB5991_11225	149.23	315.04	124.17	9.55	6.9	8.42	196.15	8.29	-4.5644	2.59E-28	3.15E-27	metAA
AB5991_11230	469.41	716.93	458.08	43.63	46.27	22.82	548.14	37.57	-3.8668	4.26E-27	4.86E-26	ugtP
AB5991_11235	2.46	0	1.31	0	0	0	1.26	0.00	-10.2954	0.264612612	0.316391271	--
AB5991_11240	374.99	536.64	464.48	344.26	232.47	169.38	458.70	248.70	-0.8831	0.004633688	0.007238543	cspD
AB5991_11245	10.86	9.07	12.68	1.07	2.01	2.14	10.87	1.74	-2.6432	0.000100164	0.000187795	degR
AB5991_11250	10.71	18.44	16.47	8.01	2.73	4.35	15.21	5.03	-1.5961	0.001475495	0.002457714	ypzA
AB5991_11255	15.8	25.39	16.9	12.89	5.03	5.35	19.36	7.76	-1.3198	0.010384049	0.015472818	ypeQ
AB5991_11260	80.16	108.19	91.68	23.38	23.77	19.54	93.34	22.23	-2.0700	4.74E-13	1.85E-12	ypeP
AB5991_11265	0.79	0.96	1.4	1.71	1.07	1.42	1.05	1.40	0.4150	0.723009334	0.774050019	ypdP
AB5991_11270	3.62	4.99	1.94	4.93	2.77	5.39	3.52	4.36	0.3112	0.635087865	0.691319603	rnhA
AB5991_11275	0	0	3	0	0	0	1.00	0.00	-9.9658	0.512210238	0.569081258	sspL
AB5991_11280	10.75	17.51	10.2	23.83	17.96	12.74	12.82	18.18	0.5037	0.152624254	0.1914916	ypcP
AB5991_11285	239.77	277.7	373.44	14.71	33.79	38.6	296.97	29.03	-3.3545	1.32E-19	8.64E-19	ypzF
AB5991_11290	11.91	10.29	12.74	6.1	3.57	3.03	11.65	4.23	-1.4601	0.002875237	0.004646954	ypbS
AB5991_11295	4.44	5.28	4.59	6.53	9.04	3.93	4.77	6.50	0.4465	0.180504416	0.22350578	dynA
AB5991_11300	0	0	0	0	0	0	0.00	0.00	0.0000	1	1	--
AB5991_11305	1.43	0	1.53	1.16	2.54	1.54	0.99	1.75	0.8240	0.336813526	0.393031594	ypbQ
AB5991_11310	1.32	3.93	2.64	4.12	3.52	2.14	2.63	3.26	0.3098	0.509918299	0.568309168	bcsA
AB5991_11315	14.27	3.28	11.01	82.24	41.62	49.92	9.52	57.93	2.6052	3.85E-09	1.08E-08	pbuX
AB5991_11320	6.8	2.84	3.97	43.35	19.18	25.42	4.54	29.32	2.6920	1.30E-09	3.77E-09	xpt
AB5991_11325	97.46	129.36	81.5	84.45	122.39	97.05	102.77	101.30	-0.0209	0.945905742	0.981754235	ypwA
AB5991_11330	227.35	345.26	171.69	97.99	106.71	107.19	248.10	103.96	-1.2548	3.04E-05	6.02E-05	kdgT
AB5991_11335	209.19	498.12	183.81	79.16	105.52	112.56	297.04	99.08	-1.5840	2.31E-05	4.63E-05	kdgA
AB5991_11340	495.16	968.78	371.32	114.52	156.41	158.13	611.75	143.02	-2.0967	5.53E-09	1.53E-08	kdgK
AB5991_11345	580.17	1015.52	471.3	96.17	121.2	118.36	689.00	111.91	-2.6222	3.75E-15	1.71E-14	kdgR
AB5991_11350	57.19	50.51	53.93	202.99	217.51	241.27	53.88	220.59	2.0336	9.63E-15	4.25E-14	kduI

AB5991_11355	58.6	55.1	46.24	230.76	244.56	266.51	53.31	247.28	2.2136	1.93E-16	9.71E-16	kduD
AB5991_11360	15.58	12.41	16.06	39.91	28.08	18.59	14.68	28.86	0.9749	0.002531918	0.004112166	ypvA
AB5991_11365	5.65	1.73	7.05	2.05	0.96	2.04	4.81	1.68	-1.5147	0.056483902	0.075955834	yptA
AB5991_11370	542.25	429.5	497.76	15.42	36.07	28.13	489.84	26.54	-4.2061	3.80E-33	6.36E-32	ypzG
AB5991_11375	50.1	49.01	51.58	29.2	28.59	17.57	50.23	25.12	-0.9997	0.000526837	0.00092365	ypsC
AB5991_11385	73.03	93.87	70.94	154.22	176.53	150.2	79.28	160.32	1.0159	0.000160563	0.000293958	gpsB
AB5991_11390	82.43	79.63	70.8	11.88	14.82	13.98	77.62	13.56	-2.5171	6.13E-18	3.48E-17	ypsA
AB5991_11395	6356.35	1155.79	6341.39	545.77	1198.15	1582.45	4617.84	1108.79	-2.0582	0.000115255	0.000214469	cotD
AB5991_11400	0	0	3	0	1.43	0	1.00	0.48	-1.0689	1	1	--
AB5991_11405	95.9	144.83	81.55	60.62	63.25	58.29	107.43	60.72	-0.8231	0.003928436	0.006195774	yprB
AB5991_11410	66.36	85.26	60.57	16.95	16.11	13.04	70.73	15.37	-2.2025	5.30E-15	2.39E-14	yprA
AB5991_11415	72.01	115.87	59.48	78.71	96.15	49.01	82.45	74.62	-0.1440	0.687397367	0.741754061	ypqE
AB5991_11420	123.94	123.27	143.13	8.89	12.26	19.57	130.11	13.57	-3.2609	2.41E-23	2.04E-22	ypqA
AB5991_11425	511.64	496.96	544.6	8.84	7.79	16.05	517.73	10.89	-5.5707	7.22E-56	5.74E-54	yppG
AB5991_11430	11.48	33.36	33.75	22.88	41.85	21.74	26.20	28.82	0.1379	0.771678351	0.819559971	yppF
AB5991_11435	6.32	3.57	8.31	9.51	8.9	3.16	6.07	7.19	0.2451	0.695595409	0.749162456	yppE
AB5991_11440	2.2	4.05	0.79	0.8	1.5	0	2.35	0.77	-1.6139	0.273839943	0.325347926	yppD
AB5991_11445	10.33	3.16	18.41	0	0	1.86	10.63	0.62	-4.1002	0.001214139	0.00205251	sspM
AB5991_11450	4.88	4.14	5.42	0.2	0.38	0.2	4.81	0.26	-4.2105	8.57E-12	3.01E-11	yppC
AB5991_11455	362.95	393.89	333.67	139.28	99.53	82.23	363.50	107.01	-1.7642	5.85E-10	1.74E-09	recU
AB5991_11460	260.82	240.97	251.81	109.43	130.48	117.25	251.20	119.05	-1.0772	1.82E-05	3.68E-05	ponA
AB5991_11465	23.73	32.85	24.6	19.86	18.21	9.49	27.06	15.85	-0.7714	0.031372744	0.043851167	ypoC
AB5991_11470	26.02	25.65	21.09	15.79	17	7.11	24.25	13.30	-0.8668	0.014382862	0.021122169	nth
AB5991_11475	24.82	24.69	28.48	21.94	15.26	8.4	26.00	15.20	-0.7743	0.031280262	0.043737296	dnaD
AB5991_11480	49.63	49.28	48.73	95.93	86.22	47.97	49.21	76.71	0.6403	0.03260123	0.045376615	asnS
AB5991_11485	33.79	68.79	37.45	65.53	58.99	32.94	46.68	52.49	0.1692	0.627062402	0.683145349	aspB

AB5991_11490	31.24	34.83	28.64	50.16	51.1	21.74	31.57	41.00	0.3771	0.288433516	0.341242534	ypmB
AB5991_11495	1.06	3.88	0	3.45	0	0	1.65	1.15	-0.5179	1	1	ypmA
AB5991_11500	26.63	36.32	26.75	79.17	53.16	57.1	29.90	63.14	1.0785	0.000122362	0.000227269	dinG
AB5991_11505	70.13	113.22	67.45	22.01	34.97	34.65	83.60	30.54	-1.4526	1.09E-05	2.25E-05	panD
AB5991_11510	15.53	13.49	15.04	42.01	61.32	57.04	14.69	53.46	1.8639	7.85E-11	2.53E-10	panC
AB5991_11515	8.89	10.74	9.73	45.49	66.83	57.95	9.79	56.76	2.5359	3.94E-17	2.09E-16	panB
AB5991_11520	12.57	12.56	12.06	12.06	13.35	8.2	12.40	11.20	-0.1460	0.66909077	0.723375836	birA
AB5991_11525	18.32	21.68	13.6	22.72	24.03	14.09	17.87	20.28	0.1828	0.554010409	0.610934555	cca
AB5991_11530	7.97	10.83	7.16	13	11.52	6.56	8.65	10.36	0.2597	0.466923884	0.526449388	bshA
AB5991_11535	2.03	3.73	3.81	12.72	13.2	5.78	3.19	10.57	1.7279	0.00018428	0.000335523	bshB1
AB5991_11540	1.31	4.01	2.33	11.4	4.89	6.62	2.55	7.64	1.5824	0.005031176	0.007828683	mgsA
AB5991_11545	0.9	5.37	3.37	8.07	12.36	2.19	3.21	7.54	1.2305	0.058916863	0.079040157	dapB
AB5991_11550	0	0.99	0.58	1.76	0	0	0.52	0.59	0.1648	1	1	ypjD
AB5991_11555	13.04	11.41	13.95	17.11	24.02	18.83	12.80	19.99	0.6429	0.034425343	0.047748179	ypjC
AB5991_11560	75.94	109.8	80.72	3.96	5.78	3.69	88.82	4.48	-4.3104	4.50E-35	8.99E-34	ypjB
AB5991_11565	10.35	4.47	9.44	3.64	4.03	0.99	8.09	2.89	-1.4861	0.003666025	0.005813813	ypjA
AB5991_11570	115.79	148.66	97.65	636.07	881.23	858.06	120.70	791.79	2.7137	3.40E-20	2.33E-19	qcrC
AB5991_11575	49.27	47.2	48.97	281.91	317.23	294.22	48.48	297.79	2.6188	1.48E-22	1.20E-21	qcrB
AB5991_11580	88.22	131.7	116.21	337.78	305.14	294.66	112.04	312.53	1.4799	4.26E-08	1.09E-07	qcrA
AB5991_11585	19	20.05	15.13	22.87	14.82	27.58	18.06	21.76	0.2687	0.443568228	0.50196906	ypiF
AB5991_11590	370.2	494.76	280.28	1245.38	1155.19	1214.55	381.75	1205.04	1.6584	6.29E-09	1.73E-08	ypiB
AB5991_11595	33.39	46.97	41.79	17.77	20.68	15.54	40.72	18.00	-1.1779	2.30E-05	4.62E-05	ypiA
AB5991_11600	99.29	159.37	101.53	74.08	65.46	49.26	120.06	62.93	-0.9319	0.001576999	0.002613632	aroA
AB5991_11605	164.23	171.3	165.93	32.76	27.53	19.64	167.15	26.64	-2.6493	4.29E-20	2.90E-19	tyrA
AB5991_11610	599.33	861.75	629.5	111.45	95.97	82.02	696.86	96.48	-2.8526	1.31E-22	1.07E-21	hisC
AB5991_11615	366.44	529.62	365.43	81.91	80.54	74.71	420.50	79.05	-2.4112	1.53E-17	8.36E-17	trpA

AB5991_11620	130.27	104.56	113.76	58.83	48.02	36.76	116.20	47.87	-1.2794	4.12E-06	8.87E-06	trpB
AB5991_11625	120.22	92.7	130.06	26.7	21.01	22.95	114.33	23.55	-2.2792	7.88E-15	3.51E-14	trpF
AB5991_11630	66.97	54.65	85.99	24.28	20.77	19.49	69.20	21.51	-1.6856	6.89E-09	1.89E-08	trpC
AB5991_11635	146.09	130.54	138.18	27.83	22.79	25.97	138.27	25.53	-2.4372	1.64E-19	1.06E-18	trpD
AB5991_11640	150.04	126.92	157.21	11.43	7.84	9.61	144.72	9.63	-3.9101	3.92E-38	9.49E-37	trpE
AB5991_11645	209.93	243.73	188.26	34.81	35.45	46.88	213.97	39.05	-2.4542	2.09E-16	1.05E-15	aroH
AB5991_11650	86.14	80.76	76.5	62.28	47.81	39.71	81.13	49.93	-0.7003	0.010625415	0.015814664	aroB
AB5991_11655	35.29	52.34	35.43	42.73	35.44	25.52	41.02	34.56	-0.2471	0.418659154	0.476496274	aroC
AB5991_11660	0.94	2.58	2.26	3.57	4.29	1.78	1.93	3.21	0.7380	0.176262078	0.218525355	cheR
AB5991_11665	36.95	75.97	41.24	167.75	168.42	113.05	51.39	149.74	1.5430	4.72E-06	1.01E-05	ndk
AB5991_11670	3.57	10.34	5.62	19.6	30.56	9.35	6.51	19.84	1.6074	0.000614919	0.001070514	hepT
AB5991_11675	9.27	12.77	6.33	15.68	17.3	9.48	9.46	14.15	0.5817	0.119453007	0.153909114	menG
AB5991_11680	11.72	28.1	15.09	19.24	18.98	10.35	18.30	16.19	-0.1770	0.688178144	0.742193213	hepS
AB5991_11685	8.72	18.92	9.33	61.22	57.29	38.62	12.32	52.38	2.0875	1.62E-07	3.96E-07	mtrB
AB5991_11690	11.36	11.58	12.82	36.37	36.28	28.68	11.92	33.78	1.5026	1.26E-06	2.84E-06	folE
AB5991_11695	123.74	132.04	86.6	1184.42	841.33	763.71	114.13	929.82	3.0263	2.52E-22	2.02E-21	hupA
AB5991_11700	1397.35	2525.64	1414.74	116.57	85.08	71.7	1779.24	91.12	-4.2874	5.68E-34	1.02E-32	spoIVA
AB5991_11705	15.25	27.54	18.67	24.61	18.52	14.64	20.49	19.26	-0.0893	0.838090682	0.880904737	yphF
AB5991_11710	0	6.51	0.95	1.93	1.8	0	2.49	1.24	-1.0000	0.796066893	0.841635152	yphE
AB5991_11715	58.33	47.64	45.62	117.04	144.08	121.58	50.53	127.57	1.3360	4.14E-07	9.78E-07	gpsA
AB5991_11720	50.32	46.07	40.69	82.32	88.12	65.82	45.69	78.75	0.7854	0.002933205	0.004725256	der
AB5991_11725	3.71	0	0.99	1.01	0	0	1.57	0.34	-2.2183	0.25431043	0.305649733	ypzH
AB5991_11730	3.84	4.83	6.92	1.54	1.65	1.31	5.20	1.50	-1.7926	0.000172533	0.000315002	yphB
AB5991_11735	3.31	5.53	4.51	0.33	3.37	2.93	4.45	2.21	-1.0098	0.099628731	0.130140029	yphA
AB5991_11740	6.85	7.54	4.39	4.47	0	1.48	6.26	1.98	-1.6582	0.11312709	0.146375913	ypzI
AB5991_11745	34.77	70.8	36.63	106.15	98.64	39.5	47.40	81.43	0.7807	0.04576174	0.062254153	fni

AB5991_11750	200.88	393.42	200.02	586.15	530.74	341.43	264.77	486.11	0.8765	0.008669283	0.013079682	ypfD
AB5991_11755	1.07	4.92	4.58	4.95	8.72	2.03	3.52	5.23	0.5708	0.37639136	0.434112719	cmk
AB5991_11760	0	0	0	0	0	0	0.00	0.00	0.0000	1	1	ypfB
AB5991_11765	0.83	1.01	0	0	0.28	0.3	0.61	0.19	-1.6656	0.485599157	0.545492009	ypfA
AB5991_11770	31.93	32.62	31.14	10.17	13.32	9.11	31.90	10.87	-1.5535	1.22E-08	3.27E-08	ypeB
AB5991_11775	1.97	3.25	2.53	2.14	1.6	0.64	2.58	1.46	-0.8233	0.158963329	0.19856665	sleB
AB5991_11780	7.43	22.73	11.77	9.57	5.6	4.47	13.98	6.55	-1.0942	0.024745032	0.035143964	prsW
AB5991_11785	90.84	140.24	96.75	29.03	32.83	14.05	109.28	25.30	-2.1106	1.37E-09	3.97E-09	ypdA
AB5991_11790	16.73	53.62	23.2	152.95	178.62	91	31.18	140.86	2.1754	1.44E-07	3.50E-07	gudB
AB5991_11795	1061.32	1408.69	1030.9	61.83	75.16	77.26	1166.97	71.42	-4.0304	1.22E-40	3.52E-39	mecB
AB5991_11800	43.8	85.1	45.35	15.12	16.51	16.31	58.08	15.98	-1.8619	1.65E-08	4.38E-08	ypbG
AB5991_11805	37.45	55.32	33.96	0.44	1.24	1.32	42.24	1.00	-5.4007	4.09E-26	4.30E-25	ypbF
AB5991_11810	51.25	60.59	57.48	20.94	23.41	17.59	56.44	20.65	-1.4508	9.17E-08	2.28E-07	ypbE
AB5991_11815	17.98	30.27	18.91	13.37	14.95	9.09	22.39	12.47	-0.8442	0.017506362	0.025473714	ypbD
AB5991_11820	15.15	29.61	16.72	34.68	32.45	26.9	20.49	31.34	0.6130	0.05044547	0.068251775	recS
AB5991_11825	4.61	8.78	6.75	17.26	13.38	7.39	6.71	12.68	0.9171	0.017502368	0.025473714	ypbB
AB5991_11830	29.04	38.65	27.17	18.95	28.81	27.5	31.62	25.09	-0.3339	0.377947911	0.435654907	fer
AB5991_11835	6.94	25.49	15.18	40.48	32.1	16.05	15.87	29.54	0.8965	0.047636108	0.064604845	fmnP
AB5991_11840	432.29	624.24	438.28	30.27	24.6	23.56	498.27	26.14	-4.2524	5.44E-42	1.66E-40	serA
AB5991_11845	30.36	47.54	30.71	11.26	12.22	4.59	36.20	9.36	-1.9521	3.95E-07	9.35E-07	aroD
AB5991_11850	106.26	147.61	117.31	47.36	24.16	16.13	123.73	29.22	-2.0823	1.84E-08	4.86E-08	rsiX
AB5991_11855	43.87	66.95	55.51	18.82	13.52	5.02	55.44	12.45	-2.1545	8.15E-08	2.04E-07	sigX
AB5991_11860	95.79	138.57	97.85	53.42	70.26	56.6	110.74	60.09	-0.8819	0.001342386	0.00225396	resE
AB5991_11865	89.75	117.52	92.77	53.84	57.5	50.61	100.01	53.98	-0.8896	0.001150443	0.001950644	resD
AB5991_11870	21.98	29.35	22.35	41.46	42.24	35.27	24.56	39.66	0.6913	0.009653639	0.014482282	resC
AB5991_11875	11.32	21.8	19.34	37.53	42.23	33.99	17.49	37.92	1.1166	0.000273798	0.00049108	resB

AB5991_11880	4.02	7.99	8.95	9.1	11.92	11.23	6.99	10.75	0.6217	0.114846887	0.148456051	resA
AB5991_11885	17.71	15.8	18.67	46	45.55	28.75	17.39	40.10	1.2051	7.43E-05	0.000140844	rluB
AB5991_11890	38.03	66.75	38.51	3.66	2.74	3.28	47.76	3.23	-3.8878	1.38E-20	9.77E-20	spmB
AB5991_11895	32.11	47.17	33.36	2	1.25	1.32	37.55	1.52	-4.6234	1.47E-26	1.61E-25	spmA
AB5991_11900	23.12	44.77	29.94	2.4	1.44	1.02	32.61	1.62	-4.3312	1.55E-24	1.45E-23	dacB
AB5991_11905	132.55	144.43	129.58	6.55	6.81	8.33	135.52	7.23	-4.2284	8.36E-39	2.14E-37	ypuI
AB5991_11910	21	20.11	21.15	4.63	4.96	1.65	20.75	3.75	-2.4697	1.90E-10	5.92E-10	scpB
AB5991_11915	8.61	8.78	7.93	3.12	3.16	3.62	8.44	3.30	-1.3548	0.000236353	0.000427394	scpA
AB5991_11920	154.24	140.34	159.79	3.37	5.61	6.71	151.46	5.23	-4.8560	3.17E-43	1.06E-41	ypuF
AB5991_11925	3210.11	3232.17	3309.19	659.99	753.99	691.86	3250.49	701.95	-2.2112	1.81E-17	9.84E-17	ribT
AB5991_11930	3210.73	3768.56	3044.9	348.77	469.99	496.52	3341.40	438.43	-2.9300	1.52E-24	1.43E-23	ribH
AB5991_11935	47.41	36.04	48.61	329.11	453.52	383.15	44.02	388.59	3.1420	2.79E-26	2.98E-25	ribBA
AB5991_11940	28.45	20.49	35.2	242.39	325.62	265.11	28.05	277.71	3.3077	1.61E-26	1.75E-25	ribE
AB5991_11945	25.3	17.11	30.26	259.39	304.73	228.99	24.22	264.37	3.4481	3.49E-28	4.19E-27	ribD
AB5991_11950	39.82	80.81	57.71	21.08	20.8	22.69	59.45	21.52	-1.4657	2.65E-05	5.29E-05	ypuD
AB5991_11955	161.43	164.74	187.69	137.82	106.64	79.04	171.29	107.83	-0.6676	0.018275728	0.026554306	sipS
AB5991_11965	69.85	62.53	65.37	8.55	4.44	3.78	65.92	5.59	-3.5597	4.20E-18	2.44E-17	ypzJ
AB5991_11970	368.29	387.98	410.18	17.54	45.77	47.77	388.82	37.03	-3.3925	5.87E-21	4.26E-20	ypzD
AB5991_11975	10.29	10.34	8.38	0	1.25	3.71	9.67	1.65	-2.5481	1.07E-05	2.19E-05	ppiB
AB5991_11990	33.39	22.59	34.09	3.82	1.79	1.09	30.02	2.23	-3.7488	2.37E-19	1.51E-18	--
AB5991_11995	18.02	21.34	14.3	0	0.39	1.47	17.89	0.62	-4.8505	5.23E-22	4.10E-21	--
AB5991_12000	0	0	0.6	0	0	0	0.20	0.00	-7.6439	1	1	--
AB5991_12005	0.42	0	1.13	0.23	0.43	0.46	0.52	0.37	-0.4688	0.799871329	0.844982455	EURM1
AB5991_12010	6	5.05	5.35	4.35	8.4	5.14	5.47	5.96	0.1255	0.805647925	0.850406143	--
AB5991_12015	59.31	62.66	52.41	44.95	47.02	43.51	58.13	45.16	-0.3642	0.156839485	0.196160502	NGR_a01970
AB5991_12020	2.47	5.44	5.28	15.58	18.6	6.95	4.40	13.71	1.6407	0.001517933	0.002522055	--

AB5991_12025	10.59	5.39	7.92	7.64	5.2	1.52	7.97	4.79	-0.7350	0.122641748	0.15766775	--
AB5991_12030	20.67	20.61	17.69	8.35	1.8	7.03	19.66	5.73	-1.7793	0.000115701	0.000215097	--
AB5991_12035	24.1	20.74	23.36	6.55	4.6	7.34	22.73	6.16	-1.8830	1.06E-05	2.18E-05	--
AB5991_12040	16.43	13.41	18.55	1.99	1.86	0	16.13	1.28	-3.6518	2.54E-08	6.61E-08	--
AB5991_12045	107.17	103.18	118.33	0	0	0	109.56	0.00	-16.7414	4.97E-49	2.67E-47	--
AB5991_12050	19.2	16.05	24.04	0	0.23	0	19.76	0.08	-8.0100	1.70E-20	1.19E-19	--
AB5991_12055	2.9	1.33	3.88	0	0	0	2.70	0.00	-11.4005	0.003678053	0.005830559	--
AB5991_12060	0	0	0	0	0	0	0.00	0.00	0.0000	1	1	--
AB5991_12065	16.74	23.05	17	143.34	143.93	101.91	18.93	129.73	2.7767	3.71E-19	2.31E-18	ppiB
AB5991_12075	161.7	299.58	149.68	59.22	48.25	39	203.65	48.82	-2.0605	5.84E-10	1.74E-09	ypuA
AB5991_12080	106.12	155.64	117.29	47.8	48.22	31.72	126.35	42.58	-1.5692	6.21E-08	1.57E-07	lysA
AB5991_12085	46.59	38.3	54.91	18.57	24.95	16.37	46.60	19.96	-1.2230	3.44E-05	6.80E-05	spoVAF
AB5991_12090	39.58	30.91	55.9	12.85	12.02	14.71	42.13	13.19	-1.6750	2.98E-07	7.15E-07	spoVAEA
AB5991_12095	12.36	15.13	21.48	3.36	4.19	1.67	16.32	3.07	-2.4091	8.59E-07	1.97E-06	spoVAEB
AB5991_12100	17.42	17.3	16.92	5.03	4.88	5.58	17.21	5.16	-1.7372	1.19E-08	3.20E-08	spoVAD
AB5991_12105	3.99	2.2	8.53	0.87	0	1.73	4.91	0.87	-2.5012	0.000926721	0.0015828	spoVAC
AB5991_12110	4.67	2.34	7.71	1.38	1.3	0	4.91	0.89	-2.4575	0.000747622	0.001287985	spoVAB
AB5991_12115	14.84	13.36	16.81	2.85	0.59	2.21	15.00	1.88	-2.9939	8.97E-12	3.14E-11	spoVAA
AB5991_12120	540.84	808.55	456.3	712.09	696.07	759.46	601.90	722.54	0.2636	0.34745371	0.404023041	sigF
AB5991_12125	262.72	376.29	219.59	370.87	352.07	299.48	286.20	340.81	0.2519	0.369297343	0.426674352	spoIIAB
AB5991_12130	103.14	164.07	105.38	198.25	199.55	173	124.20	190.27	0.6154	0.033209598	0.046158668	spoIIAA
AB5991_12135	20.39	27.52	28.58	6.72	4.87	3.68	25.50	5.09	-2.3246	3.32E-12	1.21E-11	dacF
AB5991_12140	30.79	63.86	31.5	55.41	68.08	50.11	42.05	57.87	0.4606	0.159211219	0.198778312	punA
AB5991_12145	15.71	35.85	18.11	46.45	43.93	28.9	23.22	39.76	0.7757	0.024638748	0.035018062	drm
AB5991_12150	6.69	17.88	13.02	29.56	29.11	15.59	12.53	24.75	0.9822	0.011278293	0.016723713	xerD
AB5991_12155	4.76	14.56	4.24	12.93	15.33	4.29	7.85	10.85	0.4663	0.433127885	0.490993671	yqzK

AB5991_12160	100.42	228.64	102.66	18.35	25.75	16.52	143.91	20.21	-2.8322	3.05E-13	1.20E-12	fur
AB5991_12165	6.17	13.89	4.2	0.61	0.57	1.21	8.09	0.80	-3.3435	8.49E-07	1.95E-06	spoIIM
AB5991_12170	0	0	0	0.91	1.7	0	0.00	0.87	9.7649	0.265056366	0.316457856	yqkK
AB5991_12175	14.24	17.85	12.3	99.33	100.48	98.57	14.80	99.46	2.7488	5.02E-22	3.95E-21	mleA
AB5991_12180	11.3	3.54	9.48	24.87	29.29	23.78	8.11	25.98	1.6802	4.11E-06	8.85E-06	mleN
AB5991_12185	34.18	26.03	31.94	67.45	73.43	77.69	30.72	72.86	1.2460	2.57E-06	5.62E-06	ansB
AB5991_12190	16.61	28.83	20.5	29.39	31.96	21.15	21.98	27.50	0.3232	0.288479271	0.341242534	ansA
AB5991_12195	2.06	3.78	2.75	0	2.1	2.23	2.86	1.44	-0.9883	0.260410639	0.312602977	ansR
AB5991_12200	30.9	33.93	27.4	119.57	127.06	121.7	30.74	122.78	1.9977	4.08E-14	1.71E-13	yqxK
AB5991_12205	25.59	26.17	20.78	93.71	103.52	105.9	24.18	101.04	2.0631	1.12E-13	4.51E-13	nudF
AB5991_12210	0	0	0	0	0	0	0.00	0.00	0.0000	1	1	mciZ
AB5991_12215	5.89	5.77	8.81	45.46	48.74	23.79	6.82	39.33	2.5271	8.17E-12	2.87E-11	yqkF
AB5991_12220	7.23	2.95	1.72	1.75	0.82	0	3.97	0.86	-2.2111	0.050547176	0.068366089	yqkE
AB5991_12225	3.15	5.42	3.58	11.78	7.01	5.12	4.05	7.97	0.9767	0.02053249	0.029560452	yqkD
AB5991_12230	42.17	73.29	33.83	18.02	20.7	13.04	49.76	17.25	-1.5282	0.000139716	0.000257813	yqkC
AB5991_12235	75.31	207.94	85.31	13.95	17.6	13.29	122.85	14.95	-3.0390	2.14E-12	7.94E-12	yqkB
AB5991_12240	30.48	35.05	29.41	23.24	21.03	16.49	31.65	20.25	-0.6439	0.018523529	0.026855397	yqkA
AB5991_12245	10.48	16.35	10.08	8.55	10.13	3.97	12.30	7.55	-0.7045	0.1373174	0.17461781	yqjZ
AB5991_12250	44.9	44.39	45.96	7.93	7.03	5.4	45.08	6.79	-2.7318	7.15E-17	3.74E-16	yqjY
AB5991_12255	67.18	53.85	69.56	0.58	1.63	2.89	63.53	1.70	-5.2238	8.12E-31	1.19E-29	yqjX
AB5991_12260	53.54	49.02	52.57	3.17	1.04	1.11	51.71	1.77	-4.8659	4.99E-37	1.15E-35	dinB2
AB5991_12265	6.11	3.21	11.21	7.6	5.33	1.89	6.84	4.94	-0.4702	0.528860582	0.585640093	yqzH
AB5991_12270	58.34	59.49	52.67	99.33	128.91	118.22	56.83	115.49	1.0229	7.32E-05	0.000138925	yqjV
AB5991_12275	4.55	4.17	6.08	6.18	4.63	9.84	4.93	6.88	0.4805	0.502102258	0.561963943	yqjU
AB5991_12280	19.15	20.58	12.49	35.56	34.22	21.74	17.41	30.51	0.8095	0.02072416	0.029784885	yqjT
AB5991_12285	2.82	6.22	2.62	30.1	25.49	16.31	3.89	23.97	2.6244	7.89E-11	2.54E-10	coaA

AB5991_12290	0.81	1.97	1.58	15.47	13.93	15.69	1.45	15.03	3.3704	3.61E-19	2.25E-18	dsdA
AB5991_12295	1.85	2.98	3.97	12.6	10.38	9.78	2.93	10.92	1.8964	2.37E-06	5.20E-06	yqjQ
AB5991_12300	2.82	3.11	3.22	12.9	12.26	12.03	3.05	12.40	2.0231	3.98E-09	1.11E-08	yqjP
AB5991_12305	25.91	54.32	41.34	1.64	1.32	2.34	40.52	1.77	-4.5197	1.06E-24	1.00E-23	proI
AB5991_12310	14.18	12.52	10.11	68.76	82.69	80.57	12.27	77.34	2.6561	1.67E-20	1.18E-19	yqjN
AB5991_12315	31.64	73.75	27.56	133.57	173.29	162.57	44.32	156.48	1.8200	5.21E-07	1.22E-06	namA
AB5991_12320	0	0	0	0	0	0	0.00	0.00	0.0000	1	1	--
AB5991_12325	1.46	2.49	2.28	9.07	7.18	5.16	2.08	7.14	1.7810	8.65E-06	1.81E-05	--
AB5991_12330	151.34	595.83	148.65	205.35	197	161.25	298.61	187.87	-0.6685	0.138816288	0.176114849	yqjL
AB5991_12335	21.69	57.53	24.48	3.93	3.68	6.52	34.57	4.71	-2.8756	3.82E-06	8.25E-06	rpmG2
AB5991_12340	8.02	13.65	10.88	4.47	6.17	2.75	10.85	4.46	-1.2815	0.001295137	0.00218386	rnz
AB5991_12345	87.18	161.2	96.52	64.72	59.32	36.87	114.97	53.64	-1.0999	0.000685436	0.001186515	zwf
AB5991_12350	4.36	7.53	5.76	98.57	97.72	71.33	5.88	89.21	3.9224	1.55E-32	2.48E-31	gndA
AB5991_12355	2.61	4.27	2.79	7.42	5.91	3.93	3.22	5.75	0.8358	0.037222451	0.051358704	dinB1
AB5991_12360	15.06	24.2	20.81	107.16	93.26	103.27	20.02	101.23	2.3379	1.05E-13	4.26E-13	mifM
AB5991_12365	439.87	516.67	405.03	353.75	311.04	258.52	453.86	307.77	-0.5604	0.034822579	0.048282284	misCB
AB5991_12370	2.48	7.28	4.24	19.95	18.67	27.64	4.67	22.09	2.2427	1.27E-08	3.39E-08	yqjF
AB5991_12375	31.58	37.77	31	275.32	317.15	288.93	33.45	293.80	3.1348	2.74E-29	3.53E-28	yqjE
AB5991_12385	0	0	0	1.86	0	0.46	0.00	0.77	9.5949	0.082968244	0.109421088	yqjC
AB5991_12390	0	0	0	0	0	0	0.00	0.00	0.0000	1	1	--
AB5991_12395	6.47	15	8.01	1.11	0.35	1.47	9.83	0.98	-3.3308	8.27E-08	2.07E-07	yqjB
AB5991_12400	398.25	391.14	416.31	15.62	13.86	17.37	401.90	15.62	-4.6857	1.25E-53	9.22E-52	yqjA
AB5991_12405	12.5	16.53	14.44	51.66	30.02	18.67	14.49	33.45	1.2069	0.001494482	0.002486212	artM
AB5991_12410	8.22	13.07	8.79	19.96	9.48	7.11	10.03	12.18	0.2811	0.518829125	0.575012687	artQ
AB5991_12415	25.18	40.62	27.94	56.57	19.16	16.81	31.25	30.85	-0.0186	0.976474284	1	artP
AB5991_12420	7.43	13.64	7.94	22.89	20.58	12.06	9.67	18.51	0.9367	0.020553743	0.02957207	yqiW

AB5991_12425	62.68	117.31	57.3	191.08	237.67	304.87	79.10	244.54	1.6284	8.80E-07	2.01E-06	bmrU
AB5991_12435	12.74	21.02	12.47	6.58	3.08	6.31	15.41	5.32	-1.5335	0.000100821	0.000188938	bmrR
AB5991_12440	34.02	34.88	27.29	66.91	81.95	81.03	32.06	76.63	1.2570	2.71E-06	5.92E-06	bfmBB
AB5991_12445	21.49	22.26	15.71	34.96	46.36	41.56	19.82	40.96	1.0473	0.0001495	0.000274972	bfmBAB
AB5991_12450	14.56	15.04	5.84	26.33	34.83	34.68	11.81	31.95	1.4352	5.23E-05	0.000100704	bfmBAA
AB5991_12455	6.47	7.69	5.7	18.07	14.97	12.49	6.62	15.18	1.1970	9.56E-05	0.000179562	bfmBC
AB5991_12460	5.79	12.46	4.96	18	17.52	14.33	7.74	16.62	1.1028	0.002526551	0.004105911	buk
AB5991_12465	14.53	23.34	13.95	40.21	37.63	38.77	17.27	38.87	1.1701	5.18E-05	1.00E-04	yqiT
AB5991_12470	3.41	3.32	3.22	8.52	12.47	6.3	3.32	9.10	1.4556	0.000334875	0.000597389	yqiS
AB5991_12475	0.52	1.6	1.67	9.46	7.96	4.99	1.26	7.47	2.5639	4.91E-09	1.36E-08	yqiR
AB5991_12480	7.63	11.2	8.16	72.16	30.27	45.41	9.00	49.28	2.4535	4.52E-08	1.15E-07	yqzF
AB5991_12485	164.39	326.76	168.12	23	20.1	17.28	219.76	20.13	-3.4487	9.02E-23	7.40E-22	mmgF
AB5991_12490	174.89	391.06	158.15	19.53	14.78	16.82	241.37	17.04	-3.8239	1.45E-22	1.18E-21	mmgE
AB5991_12495	133.91	237.87	133.01	11.07	9.04	7.69	168.26	9.27	-4.1825	4.10E-32	6.33E-31	mmgD
AB5991_12500	110.99	222.13	102.07	7.76	9.36	9.1	145.06	8.74	-4.0529	8.99E-27	9.92E-26	mmgC
AB5991_12505	87.24	163.26	82.78	5.92	5.96	3.17	111.09	5.02	-4.4689	1.44E-27	1.70E-26	mmgB
AB5991_12510	91.9	156.96	70.48	5.65	4.2	3.64	106.45	4.50	-4.5651	2.12E-30	3.00E-29	mmgA
AB5991_12515	14.32	36.73	18.22	19.07	15.58	18.44	23.09	17.70	-0.3838	0.328945332	0.384641317	yqiK
AB5991_12520	2.04	1.07	0.31	0.95	1.48	0	1.14	0.81	-0.4930	0.700192726	0.75290152	yqiI
AB5991_12525	0.61	4.47	3.25	0	0.62	1.98	2.78	0.87	-1.6798	0.192558234	0.237247516	yqiH
AB5991_12530	0	0	0	0	0	0	0.00	0.00	0.0000	1	1	--
AB5991_12535	16.48	32.92	17.62	108.74	137.6	114	22.34	120.11	2.4267	6.25E-14	2.58E-13	yqiG
AB5991_12540	382.41	444.58	432.75	94.87	69.79	61.57	419.91	75.41	-2.4773	4.24E-18	2.46E-17	spo0A
AB5991_12545	33.72	40.16	31.84	3.84	4.16	6.72	35.24	4.91	-2.8444	8.94E-19	5.42E-18	spoIVB
AB5991_12550	73.41	83.02	70.91	57.12	47.93	33.8	75.78	46.28	-0.7113	0.010393093	0.015480484	recN
AB5991_12555	45.79	64.17	47.68	25.77	25.35	12.17	52.55	21.10	-1.3166	0.000146453	0.000269742	argR

AB5991_12560	20.3	25.11	24.22	179.62	175.26	147.56	23.21	167.48	2.8512	6.06E-24	5.37E-23	yqxC
AB5991_12565	18.53	27.05	21.24	147.18	130.57	100.92	22.27	126.22	2.5026	1.20E-17	6.71E-17	dxs
AB5991_12570	4.67	2.98	4.34	35.08	28.7	21.08	4.00	28.29	2.8233	1.56E-14	6.80E-14	ispA
AB5991_12575	0.71	1.3	1.52	6.94	5.05	6.14	1.18	6.04	2.3606	0.004647646	0.007257493	xseB
AB5991_12580	3.09	6.41	5.17	29.48	28.13	17.58	4.89	25.06	2.3577	3.50E-11	1.17E-10	xseA
AB5991_12585	72.55	80.25	70.33	161.28	133.65	120.8	74.38	138.58	0.8978	0.000570899	0.000997379	folD
AB5991_12590	29.67	28.5	19.52	44.18	40.88	21.74	25.90	35.60	0.4591	0.20094926	0.246515141	nusB
AB5991_12595	106.77	149.68	132.65	968.45	995.54	759.63	129.70	907.87	2.8073	3.04E-22	2.43E-21	yqhY
AB5991_12600	24.71	39.98	32.14	659.34	586.41	482.14	32.28	575.96	4.1574	4.64E-39	1.21E-37	accC1
AB5991_12605	0.75	3.46	2.42	36.45	49.44	11.41	2.21	32.43	3.8754	3.85E-10	1.17E-09	accB
AB5991_12610	1.46	2.49	2.28	9.07	7.18	5.16	2.08	7.14	1.7810	8.71E-06	1.82E-05	--
AB5991_12615	331.24	351.09	251.25	25.13	23.8	16.68	311.19	21.87	-3.8308	2.02E-34	3.78E-33	spoIIIAH
AB5991_12620	16.77	18.76	26.61	3.42	1.87	0.57	20.71	1.95	-3.4065	1.08E-13	4.36E-13	spoIIIAG
AB5991_12625	45.41	57.19	39.22	1.9	0.59	3.15	47.27	1.88	-4.6522	3.96E-27	4.53E-26	spoIIIAF
AB5991_12630	23.2	34.02	23.52	2.13	1.38	0.65	26.91	1.39	-4.2786	1.42E-25	1.46E-24	spoIIIAE
AB5991_12635	8.09	10.73	8.17	0.98	0.46	0	9.00	0.48	-4.2283	5.95E-10	1.77E-09	spoIIIAD
AB5991_12640	0.87	0	0	0	0	0	0.29	0.00	-8.1799	1	1	spoIIIAC
AB5991_12645	11.56	13.51	13.11	1.9	2.5	0.76	12.73	1.72	-2.8874	5.71E-10	1.70E-09	spoIIIAB
AB5991_12650	29.73	56.39	32.22	2.55	2.19	3.39	39.45	2.71	-3.8635	8.68E-22	6.61E-21	spoIIIAA
AB5991_12655	0.64	7.06	5.48	0	0	0	4.39	0.00	-12.1011	0.000460114	0.000810609	yqhV
AB5991_12660	17.82	16.65	21.13	145.85	172.09	110.8	18.53	142.91	2.9469	1.26E-20	9.02E-20	efp
AB5991_12665	23.66	34.06	25.12	156.97	186.04	146.29	27.61	163.10	2.5623	3.09E-19	1.95E-18	yqhT
AB5991_12670	4.45	7.42	6.05	38.26	44.03	33.27	5.97	38.52	2.6890	9.00E-14	3.68E-13	yqhS
AB5991_12675	9.09	9.89	5.04	0.37	0	0.36	8.01	0.24	-5.0402	2.85E-10	8.73E-10	yqhR
AB5991_12680	1532.31	1572.75	1594.22	112.16	124.18	132.69	1566.43	123.01	-3.6706	9.99E-40	2.75E-38	yqhQ
AB5991_12685	1883.26	2160.64	2132.09	378.27	362.81	430.85	2058.66	390.64	-2.3978	9.36E-20	6.21E-19	yqhP

AB5991_12690	12.59	12.88	14.56	2.02	2.1	2.46	13.34	2.19	-2.6049	2.51E-12	9.24E-12	yqhO
AB5991_12695	123.03	140.8	127.06	25.2	42.45	11.4	130.30	26.35	-2.3059	2.44E-09	6.94E-09	mntR
AB5991_12700	0.65	1.98	0.92	9.63	12.31	5.38	1.18	9.11	2.9441	4.07E-08	1.05E-07	lipM
AB5991_12705	2.85	3.48	5.07	10.84	9.17	6.16	3.80	8.72	1.1989	0.015323697	0.022437463	yqhL
AB5991_12710	12.2	11.31	9.36	17.42	40.88	37.21	10.96	31.84	1.5389	8.84E-06	1.84E-05	gcvPB
AB5991_12715	15.16	12.32	10.62	29.48	52.03	41.98	12.70	41.16	1.6965	4.52E-08	1.15E-07	gcvPA
AB5991_12720	7.3	6.4	6.92	18.77	28.38	28.21	6.87	25.12	1.8698	3.56E-09	9.98E-09	gcvT
AB5991_12725	3.56	6.54	4.16	1.29	0.77	0.94	4.75	1.00	-2.2489	3.32E-07	7.90E-07	yqhH
AB5991_12730	1.36	1.25	4.13	0.25	0.46	0.25	2.25	0.32	-2.8116	0.000722102	0.001247809	yqhG
AB5991_12735	1.04	3.81	0	0	1.06	1.12	1.62	0.73	-1.1537	0.706438415	0.757975398	sinI
AB5991_12740	50.57	80.01	85.72	29.25	26.28	23.29	72.10	26.27	-1.4564	6.81E-06	1.44E-05	sinR
AB5991_12745	361.04	575.95	294.86	12472.38	15154.84	13937.76	410.62	13854.99	5.0765	2.29E-51	1.47E-49	tasA
AB5991_12750	4.42	7.53	5.06	134.48	104.02	59.07	5.67	99.19	4.1288	2.25E-24	2.07E-23	sipW
AB5991_12755	3.8	10.02	7.36	54.95	51.9	20.8	7.06	42.55	2.5914	4.91E-09	1.36E-08	tapA
AB5991_12760	110	132.96	116.45	19.24	13.5	9.57	119.80	14.10	-3.0866	1.09E-20	7.80E-20	yqzG
AB5991_12765	1	1.84	3.22	0	0	0	2.02	0.00	-10.9801	0.073412963	0.097466692	yqzE
AB5991_12770	0.48	0	0	0	0.49	0	0.16	0.16	0.0297	1	1	comGG
AB5991_12775	0	0	0	2.05	0.48	1.02	0.00	1.18	10.2086	0.02256697	0.032269874	comGF
AB5991_12780	0	1.91	0	0	0.53	1.12	0.64	0.55	-0.2111	1	1	comGE
AB5991_12785	0.84	1.54	0	2.28	2.98	3.17	0.79	2.81	1.8246	0.023905534	0.034061311	comGD
AB5991_12790	0	2.23	0	0.66	0.62	0	0.74	0.43	-0.8009	1	1	comGC
AB5991_12795	2.44	1.92	2.23	0.57	0	1.88	2.20	0.82	-1.4275	0.039134341	0.053847009	comGB
AB5991_12800	4.89	3.41	1.62	4.59	5.5	4.57	3.31	4.89	0.5635	0.208546244	0.255282717	comGA
AB5991_12805	6.39	6.7	6.83	64.53	80.83	55.34	6.64	66.90	3.3328	1.34E-15	6.26E-15	yhcV
AB5991_12810	13.07	12.52	10.94	64.7	81.76	82.65	12.18	76.37	2.6489	1.93E-19	1.24E-18	corA
AB5991_12815	408.42	323.4	389.5	84.9	99.8	96.87	373.77	93.86	-1.9936	1.15E-13	4.64E-13	yqhB

AB5991_12820	47.72	70.18	45.73	33.35	16.7	25.48	54.54	25.18	-1.1153	0.000574203	0.001002269	rsbRD
AB5991_12830	81.6	233.46	92.33	885.39	689.48	828.35	135.80	801.07	2.5605	3.38E-11	1.13E-10	mgsR
AB5991_12835	1.47	6.75	4.71	41.55	50.1	37.38	4.31	43.01	3.3189	2.93E-13	1.16E-12	yqgY
AB5991_12840	4.26	15.66	10.64	32.15	23.14	18.15	10.19	24.48	1.2649	0.00271471	0.004396457	yqgX
AB5991_12845	61.29	97.28	88.87	533.25	610.02	400.32	82.48	514.53	2.6411	1.54E-16	7.85E-16	yqgW
AB5991_12850	14.92	23.18	22.7	19.97	26.86	14.29	20.27	20.37	0.0076	1	1	yqgV
AB5991_12855	11.57	18.44	13.83	33.13	31.35	34.82	14.61	33.10	1.1795	3.57E-05	7.03E-05	yqgU
AB5991_12860	16.14	21.42	15.72	5.39	6.02	4.32	17.76	5.24	-1.7601	3.87E-08	9.98E-08	yqgT
AB5991_12865	469.46	500.87	494.07	94.03	89.53	69.51	488.13	84.36	-2.5327	7.56E-21	5.47E-20	yqgS
AB5991_12870	39.67	55.66	38.22	42.53	41.32	35.04	44.52	39.63	-0.1678	0.562835513	0.619633996	glcK
AB5991_12875	7.53	10.76	5.37	5.46	0	3.62	7.89	3.03	-1.3817	0.052171551	0.07041918	yqgQ
AB5991_12880	3.68	5.66	5.71	14.83	20.4	14.38	5.02	16.54	1.7209	1.15E-07	2.83E-07	gluP
AB5991_12885	11.43	7.63	13.33	0	6.34	0	10.80	2.11	-2.3530	0.002992729	0.00480944	yqgO
AB5991_12890	0.96	0	0.34	5.93	7.5	1.73	0.43	5.05	3.5437	7.14E-05	0.000135748	yqgN
AB5991_12895	15.66	8.85	7.73	41.94	20.85	26.09	10.75	29.63	1.4630	0.004202682	0.006604215	rpmGA
AB5991_12900	3.51	2.15	3.22	7.64	5.11	2.54	2.96	5.10	0.7840	0.113623618	0.146970484	yqgM
AB5991_12905	0.77	3.52	0.82	9.18	7.42	1.25	1.70	5.95	1.8045	0.01862026	0.026966103	yqgL
AB5991_12910	5.11	13.13	7.64	29.43	41.05	27.08	8.63	32.52	1.9145	1.25E-06	2.83E-06	yqzD
AB5991_12915	39.65	51.39	37	113.3	125.8	128.76	42.68	122.62	1.5226	3.61E-08	9.33E-08	yqzC
AB5991_12920	5.31	9.33	8.89	26.61	25.84	15.49	7.84	22.65	1.5298	1.79E-05	3.62E-05	pstB1
AB5991_12925	6.92	9.01	4.53	13.59	15.22	9.66	6.82	12.82	0.9109	0.011239138	0.016678108	pstB2
AB5991_12930	1.63	4.5	2.4	10	5.61	3.32	2.84	6.31	1.1501	0.026408148	0.037292588	yqgI
AB5991_12935	1.36	1.78	2.29	2.96	2.37	2.52	1.81	2.62	0.5317	0.284199793	0.336782267	yqgH
AB5991_12940	16.21	33.08	17.98	8.27	3.67	3.9	22.42	5.28	-2.0864	6.53E-07	1.52E-06	pstS
AB5991_12945	73.19	69.28	67.58	92.76	102.63	84.6	70.02	93.33	0.4146	0.100312855	0.130904485	pbpA
AB5991_12950	7.97	6.93	10.31	4.71	4.84	2.88	8.40	4.14	-1.0202	0.003424276	0.005445656	yqgE

AB5991_12955	141.57	340.06	141.56	1657.54	1687.05	1640.15	207.73	1661.58	2.9998	2.73E-16	1.35E-15	sodA
AB5991_12960	1.5	3.44	1.6	58.2	76.55	78.99	2.18	71.25	5.0304	9.39E-33	1.52E-31	yqgC
AB5991_12965	1.42	1.31	2.54	24.51	19.07	12.33	1.76	18.64	3.4072	9.53E-14	3.88E-13	yqgB
AB5991_12970	6.02	9.96	5.8	0	0	0	7.26	0.00	-12.8258	1.10E-07	2.73E-07	yqfZ
AB5991_12975	249.77	698.61	257.04	50.1	43.96	34.68	401.81	42.91	-3.2270	9.87E-15	4.35E-14	ispG
AB5991_12980	328.13	371.89	392.04	79.14	80.66	120.41	364.02	93.40	-1.9625	4.86E-12	1.75E-11	yqfX
AB5991_12985	19.88	33.07	20.26	21.62	17.07	8.07	24.40	15.59	-0.6468	0.100298877	0.130904485	yqfW
AB5991_12990	5.36	3.03	3.09	21.99	14.7	11.61	3.83	16.10	2.0729	7.30E-06	1.54E-05	zur
AB5991_12995	17.42	18.44	18.41	8.02	6.05	6.21	18.09	6.76	-1.4201	3.56E-06	7.72E-06	yqfU
AB5991_13000	79.39	49.46	90.96	8.48	22.36	19.18	73.27	16.67	-2.1357	5.98E-08	1.51E-07	yqfT
AB5991_13005	21.03	12.25	16.43	72.12	67.7	54.72	16.57	64.85	1.9685	4.97E-11	1.63E-10	nfo
AB5991_13010	10.98	11.09	11.59	51.35	57.69	44.72	11.22	51.25	2.1916	1.03E-14	4.52E-14	cshB
AB5991_13015	261.41	200.29	236.68	13.48	34.86	53.91	232.79	34.08	-2.7719	1.19E-11	4.11E-11	--
AB5991_13020	74.98	71.65	76.7	36.2	30.76	15.74	74.44	27.57	-1.4332	1.21E-05	2.47E-05	ispH
AB5991_13025	27.06	28.99	23.08	108.98	94.93	91.03	26.38	98.31	1.8981	2.60E-12	9.54E-12	yqfO
AB5991_13030	15.55	10.2	11.88	20.23	22.61	18.03	12.54	20.29	0.6938	0.028648083	0.040241082	trmK
AB5991_13035	103.07	190.17	119.81	605.99	640.06	583.75	137.68	609.93	2.1473	7.18E-13	2.77E-12	cccA
AB5991_13040	275.33	252.49	245.43	929.18	1042.76	1042.83	257.75	1004.92	1.9630	3.31E-14	1.39E-13	sigA
AB5991_13045	115.51	106.6	117.13	435.58	488.02	473.6	113.08	465.73	2.0422	1.74E-15	8.09E-15	dnaG
AB5991_13050	69.01	66.38	77.71	75.06	71.35	67.59	71.03	71.33	0.0061	0.991138692	1	yqxD
AB5991_13055	57.36	82.46	55.63	44.01	38.01	28.88	65.15	36.97	-0.8175	0.005966754	0.009194405	yqfL
AB5991_13060	13.58	24.93	14.52	13.54	14.39	12.25	17.68	13.39	-0.4003	0.273079363	0.324808076	ccpN
AB5991_13065	58.3	59.22	52.11	56.85	79.35	77.98	56.54	71.39	0.3364	0.200403277	0.245997346	glyS
AB5991_13070	6.92	4.11	7.62	31.21	31.9	28.86	6.22	30.66	2.3020	1.17E-12	4.43E-12	glyQ
AB5991_13075	966.11	898.44	973.01	127.73	152.1	153.12	945.85	144.32	-2.7124	4.44E-24	3.97E-23	recO
AB5991_13080	126.78	78.36	153.02	13.65	26.83	20.38	119.39	20.29	-2.5570	7.54E-11	2.44E-10	yqzL

AB5991_13085	1693.38	1771.55	1715.54	293.79	326.5	314.66	1726.82	311.65	-2.4701	6.62E-22	5.13E-21	era
AB5991_13090	1193.56	1317.06	1111.79	255.89	291.38	298.01	1207.47	281.76	-2.0994	2.95E-15	1.36E-14	cdd
AB5991_13095	9.23	1.78	7.27	24.31	29.18	22.09	6.09	25.19	2.0477	3.29E-06	7.14E-06	dgkA
AB5991_13100	19.83	21.01	19.57	97.05	101.68	101.96	20.14	100.23	2.3154	2.26E-17	1.22E-16	ybeY
AB5991_13105	10.92	8.86	10.77	66.63	72.17	59.36	10.18	66.05	2.6974	3.76E-22	3.00E-21	pgpH
AB5991_13110	1021.61	1726.17	1042.18	155.63	135.29	100.07	1263.32	130.33	-3.2770	1.28E-23	1.09E-22	phoH
AB5991_13115	50.43	66.27	47.15	6.24	3.84	4.74	54.62	4.94	-3.4668	2.47E-26	2.67E-25	yqfD
AB5991_13120	3.2	11.77	4.11	0.7	0.65	0.69	6.36	0.68	-3.2254	0.001339356	0.002249824	yqfC
AB5991_13125	216.04	238.64	231.03	110.46	102.49	81.06	228.57	98.00	-1.2217	4.07E-06	8.76E-06	yqfB
AB5991_13130	218.31	225.59	203.39	114.87	85.52	84.28	215.76	94.89	-1.1851	7.02E-06	1.48E-05	floA
AB5991_13135	216.1	238.94	198.44	107.12	65.24	72.52	217.83	81.63	-1.4161	6.03E-07	1.40E-06	nfeD1
AB5991_13140	478.76	446.98	492.1	2359.85	2124.38	1801.66	472.61	2095.30	2.1484	5.46E-16	2.65E-15	yqeY
AB5991_13145	67.52	53.41	67.76	353.62	314	253.01	62.90	306.88	2.2866	7.10E-15	3.17E-14	rpsU
AB5991_13150	9.98	21.91	15.06	0.64	0.8	0.42	15.65	0.62	-4.6578	5.06E-18	2.90E-17	yqeW
AB5991_13155	107.84	115.28	104.77	81.18	70.41	59.88	109.30	70.49	-0.6328	0.014215799	0.020906747	mtaB
AB5991_13160	35.63	41.32	37.36	12.49	14.79	6.09	38.10	11.12	-1.7763	1.83E-07	4.45E-07	rsmE
AB5991_13165	22.01	30.85	21.89	22.89	20.83	19.23	24.92	20.98	-0.2479	0.405518844	0.462867297	prmA
AB5991_13170	74.51	79.74	69.4	35.03	32.29	36.43	74.55	34.58	-1.1081	2.63E-05	5.26E-05	dnaJ
AB5991_13175	738.75	808.03	803.92	235.34	232.15	254.17	783.57	240.55	-1.7037	1.05E-11	3.65E-11	dnaK
AB5991_13180	38.14	60.61	52.78	82.61	93.61	84.99	50.51	87.07	0.7856	0.005183299	0.008055922	grpE
AB5991_13185	746.99	1552.04	874.32	312.59	306.78	336.34	1057.78	318.57	-1.7314	3.82E-08	9.86E-08	hrcA
AB5991_13190	32.19	39.59	31.2	27.94	34.37	31.07	34.33	31.13	-0.1412	0.606886576	0.663166371	hemW
AB5991_13195	74.21	116.59	81.77	92.68	76.52	49.05	90.86	72.75	-0.3206	0.298226049	0.351724277	lepA
AB5991_13200	53.32	78.32	44.47	9.86	5.97	6.35	58.70	7.39	-2.9891	1.92E-14	8.26E-14	yqxA
AB5991_13205	41.07	58.07	61.71	6.03	6.41	6.81	53.62	6.42	-3.0628	6.13E-23	5.07E-22	spoIIP
AB5991_13210	25.8	30.88	35.45	7.1	5.65	4.95	30.71	5.90	-2.3799	2.00E-14	8.61E-14	gpr

AB5991_13215	172.63	208.83	162.16	1714.74	1299.42	1219.4	181.21	1411.19	2.9612	4.39E-24	3.93E-23	rpsT
AB5991_13220	144.56	104.27	130.34	27.68	25.9	27.36	126.39	26.98	-2.2279	5.53E-16	2.67E-15	yqeN
AB5991_13225	110.86	68.59	125	1.31	2.45	1.3	101.48	1.69	-5.9109	7.58E-25	7.22E-24	--
AB5991_13230	49.54	81.13	73.02	355.3	343.39	211.6	67.90	303.43	2.1600	8.31E-11	2.67E-10	yqzM
AB5991_13235	10.47	8.54	10.03	1.77	1.34	0.59	9.68	1.23	-2.9724	6.92E-16	3.32E-15	comEC
AB5991_13240	11.73	12.81	12.21	3.1	4.2	1.72	12.25	3.01	-2.0265	8.44E-07	1.94E-06	comEB
AB5991_13245	2.05	3.22	2.5	1.27	0	0	2.59	0.42	-2.6131	0.003411412	0.005431723	comEA
AB5991_13250	91.47	169.58	131.21	9.09	9.18	9.05	130.75	9.11	-3.8438	1.95E-28	2.39E-27	comER
AB5991_13255	25.99	32.12	24.42	57.6	63.54	47.86	27.51	56.33	1.0340	0.000161398	0.000295351	yqeM
AB5991_13260	19.75	13.02	19.49	39.1	40.19	39.46	17.42	39.58	1.1841	0.000182132	0.000332069	rsfS
AB5991_13265	11.6	13.02	14.82	33.64	32.14	29.65	13.15	31.81	1.2748	1.77E-05	3.58E-05	yqeK
AB5991_13270	12.68	18.05	14.24	51.73	47.44	43.94	14.99	47.70	1.6701	2.10E-08	5.54E-08	nadD
AB5991_13275	3.73	15.97	9.3	30.4	43.62	29.58	9.67	34.53	1.8369	3.90E-05	7.65E-05	yqeI
AB5991_13280	11.79	10.63	10.09	33.81	39.5	43.64	10.84	38.98	1.8469	2.46E-10	7.61E-10	aroE
AB5991_13285	3.45	1.81	2.81	16.43	10.53	7.29	2.69	11.42	2.0855	1.17E-06	2.66E-06	yqeH
AB5991_13290	2.79	2.56	2.98	4.92	0.71	2.64	2.78	2.76	-0.0104	1	1	yqeG
AB5991_13295	5.13	7.06	10.97	5.58	0	0	7.72	1.86	-2.0533	0.03719821	0.05134463	sda
AB5991_13300	8.4	12.24	11.09	17.99	11.06	16.04	10.58	15.03	0.5070	0.131262928	0.167764753	yqeF
AB5991_13305	7587.86	9186.7	8494.4	66.57	94.79	141.87	8422.99	101.08	-6.3808	9.91E-67	1.97E-64	cwlH
AB5991_13310	213.61	139.21	229.05	5.64	3.52	2.5	193.96	3.89	-5.6411	1.72E-44	6.28E-43	yqeD
AB5991_13315	4.45	4.83	5.62	0.66	0.41	0.88	4.97	0.65	-2.9338	3.10E-08	8.03E-08	yqeC
AB5991_13320	181.75	231.82	177.52	69.88	51.14	53.31	197.03	58.11	-1.7616	1.71E-10	5.37E-10	yqeB
AB5991_13325	60.69	76.71	79.01	4.78	1.34	1.9	72.14	2.67	-4.7540	2.97E-29	3.80E-28	nucB
AB5991_13330	104.63	141.59	102.61	11.33	11.1	12.35	116.28	11.59	-3.3262	5.83E-26	6.06E-25	sigK
AB5991_13335	0	0	0	0	0	0	0.00	0.00	0.0000	1	1	--
AB5991_13345	4.42	5.97	8.07	1.98	2.25	1.55	6.15	1.93	-1.6753	3.09E-05	6.11E-05	fumC

AB5991_13350	28.4	27.19	28.2	5.74	9.08	5.71	27.93	6.84	-2.0290	1.62E-10	5.09E-10	yybE
AB5991_13355	69.83	66.21	46.86	486.49	489.16	423.44	60.97	466.36	2.9354	3.36E-24	3.04E-23	--
AB5991_13360	27.72	25.3	21.08	51.76	40.77	34.69	24.70	42.41	0.7798	0.005271194	0.008179723	rapH
AB5991_13370	0	0	0.67	0	1.28	0	0.22	0.43	0.9339	1	1	--
AB5991_13375	8.61	9.03	9.2	6.24	2.09	2.22	8.95	3.52	-1.3471	0.007204339	0.01098634	--
AB5991_13380	4.79	4.4	7.25	1.3	0.81	1.73	5.48	1.28	-2.0980	0.000438046	0.00077276	--
AB5991_13385	6.53	11.06	6.98	0.55	0.51	2.17	8.19	1.08	-2.9273	8.31E-06	1.74E-05	--
AB5991_13390	3.52	5.82	6.78	0	0	0	5.37	0.00	-12.3916	3.55E-09	9.98E-09	--
AB5991_13395	0.89	1.63	5.69	0	1.8	0	2.74	0.60	-2.1894	0.163441103	0.203455994	--
AB5991_13400	0.77	1.42	3.3	0.84	0	0	1.83	0.28	-2.7083	0.150442592	0.189172746	--
AB5991_13410	1.86	1.14	0	0	1.26	0	1.00	0.42	-1.2515	0.708651572	0.759939344	yxxE
AB5991_13415	5.22	5.43	5.74	1.95	2.3	2.06	5.46	2.10	-1.3771	5.97E-05	0.000114424	tnpA
AB5991_13420	12.07	12.39	12.65	3.28	5.41	5.33	12.37	4.67	-1.4043	6.16E-05	0.000117655	tnpB
AB5991_13430	0	0	0	0	2.11	0	0.00	0.70	9.4581	1	1	--
AB5991_13445	0	0	1.9	0	0	0	0.63	0.00	-9.3068	0.272812055	0.324643893	--
AB5991_13455	22.97	29.04	29.8	8.19	8.05	12.64	27.27	9.63	-1.5022	9.84E-06	2.03E-05	--
AB5991_13460	76.5	133	66.6	396.84	315.55	318.04	92.03	343.48	1.9000	3.76E-09	1.05E-08	--
AB5991_13465	0	0	0	0	0	0	0.00	0.00	0.0000	1	1	yqaO
AB5991_13480	0	0	0	0	0	0	0.00	0.00	0.0000	1	1	--
AB5991_13490	0	0	0	0.24	0.45	0	0.00	0.23	7.8455	0.265031725	0.316457856	bltR
AB5991_13495	16.38	13.52	14.94	2.94	2.45	1.46	14.95	2.28	-2.7106	1.89E-14	8.14E-14	blt
AB5991_13500	40.56	41.22	45.9	0.43	1.6	0	42.56	0.68	-5.9749	2.81E-32	4.42E-31	bltD
AB5991_13505	12.33	10.94	11.41	2.41	1.13	2.4	11.56	1.98	-2.5456	1.55E-12	5.81E-12	yrkA
AB5991_13510	0	0	0	0	0	0	0.00	0.00	0.0000	1	1	yrzO
AB5991_13515	3.93	5.15	5.8	0.61	0.76	0.2	4.96	0.52	-3.2445	5.56E-09	1.54E-08	yrdR
AB5991_13520	42.53	42.11	39.91	7.26	9.55	6.09	41.52	7.63	-2.4433	7.85E-16	3.73E-15	czcR

AB5991_13525	0.87	2.56	1.3	1.89	1.77	0.75	1.58	1.47	-0.1011	1	1	czcO
AB5991_13530	1.54	1.06	1.86	0.42	0.79	1.25	1.49	0.82	-0.8584	0.216226069	0.263789161	czcD
AB5991_13545	36.07	39.64	37.99	3.86	3.2	3.4	37.90	3.49	-3.4423	1.25E-29	1.68E-28	brnQ
AB5991_13550	17.37	9.97	13.35	2.95	4.42	3.53	13.56	3.63	-1.9003	6.14E-05	0.000117379	azlD
AB5991_13560	7.63	4.9	5.3	0.41	0.78	0.41	5.94	0.53	-3.4782	2.84E-07	6.82E-07	azlB
AB5991_13565	0	0	0	0	0	0	0.00	0.00	0.0000	1	1	yrdF
AB5991_13570	4.69	6.73	4.86	1.12	0.45	0.48	5.43	0.68	-2.9894	6.82E-10	2.02E-09	cypA
AB5991_13575	2.32	0	0.62	0	0	0	0.98	0.00	-9.9366	0.079986768	0.105699653	--
AB5991_13580	1.28	2.34	0.34	0	0	0.35	1.32	0.12	-3.5001	0.024155222	0.03437171	yrdC
AB5991_13585	0.49	0	1.04	0	0	0	0.51	0.00	-8.9944	0.264686149	0.316391271	yrdB
AB5991_13590	0	0	1.24	0	0	0	0.41	0.00	-8.6912	1	1	--
AB5991_13600	40.88	63.88	39.12	61.63	45.62	35.33	47.96	47.53	-0.0131	1	1	yrdA
AB5991_13605	1.27	0.78	2.26	12.88	8.18	8.92	1.44	9.99	2.7982	2.11E-09	6.04E-09	aadK
AB5991_13610	12.29	16.53	10	38.41	50.04	42.92	12.94	43.79	1.7588	1.62E-09	4.67E-09	yrpB
AB5991_13615	36.01	47.41	28.82	3.94	4.38	2.7	37.41	3.67	-3.3484	1.41E-19	9.16E-19	yrpC
AB5991_13620	66.89	95.16	70.44	407.04	439.89	464.28	77.50	437.07	2.4957	4.15E-19	2.57E-18	yrpD
AB5991_13625	0	0	0	0	0	0	0.00	0.00	0.0000	1	1	--
AB5991_13630	2.23	8.19	3.58	0	0	0	4.67	0.00	-12.1882	0.004258455	0.006689211	--
AB5991_13635	13.39	25.46	12.78	6.5	8.03	5.69	17.21	6.74	-1.3524	0.000461474	0.000812645	yrpE
AB5991_13640	5.79	4.38	6.55	15.55	14.55	14.37	5.57	14.82	1.4113	0.000124273	0.000230709	sigZ
AB5991_13645	86.97	79.84	73.49	11.62	17.06	17.95	80.10	15.54	-2.3655	3.26E-15	1.49E-14	yrpG
AB5991_13650	814.48	920.83	551.46	4433.43	4946.6	4413.41	762.26	4597.81	2.5926	5.97E-20	4.01E-19	csn
AB5991_13655	100.64	111.85	119.66	406.12	303.23	287.4	110.72	332.25	1.5854	2.07E-08	5.45E-08	yraL
AB5991_13660	16.84	14.37	16.23	12.9	8.93	8.47	15.81	10.10	-0.6468	0.037825056	0.052153922	yraK
AB5991_13665	13.16	14.87	12.99	442.72	772.94	717.42	13.67	644.36	5.5584	7.92E-51	4.76E-49	yraJ
AB5991_13670	13.05	27.66	9.66	413.37	612.17	575.03	16.79	533.52	4.9899	7.63E-37	1.70E-35	yraI

AB5991_13675	0	0	0	0	0	0	0.00	0.00	0.0000	1	1	--
AB5991_13680	14.95	25.73	24.97	10.67	11.41	9.1	21.88	10.39	-1.0742	0.004410888	0.006914977	yraH
AB5991_13685	0.73	0	7.07	0	1.5	1.59	2.60	1.03	-1.3359	0.421764011	0.479343128	yraG
AB5991_13690	5.39	8.09	12.57	0.53	1.99	0.53	8.68	1.02	-3.0944	5.93E-06	1.26E-05	yraF
AB5991_13695	14.94	12.55	15.98	1.04	2.43	4.3	14.49	2.59	-2.4840	1.03E-09	3.00E-09	adhB
AB5991_13700	3.65	21.79	7.81	1.99	2.79	2.96	11.08	2.58	-2.1029	0.009906457	0.014833538	yraE
AB5991_13705	54.22	57.53	74.1	1.97	3.68	3.26	61.95	2.97	-4.3826	9.87E-25	9.36E-24	yraD
AB5991_13710	20.94	34.52	28.33	25.56	36.53	33.77	27.93	31.95	0.1942	0.527771698	0.584597325	adhR
AB5991_13715	0.93	1.7	1.98	0	1.89	0	1.54	0.63	-1.2864	0.703222189	0.755340902	--
AB5991_13720	62.83	59.11	53.57	117.01	132.45	102.49	58.50	117.32	1.0038	0.000102485	0.000191967	adhA
AB5991_13725	46.78	128.85	57.23	145.32	135.99	130.06	77.62	137.12	0.8210	0.023072532	0.032957203	yraA
AB5991_13730	9.86	9.63	7.89	75.29	116.04	128.81	9.13	106.71	3.5475	1.01E-28	1.27E-27	sacC
AB5991_13735	8.51	4.81	7.47	24.93	35.55	37.57	6.93	32.68	2.2376	4.97E-11	1.63E-10	levG
AB5991_13740	1.56	0.82	2.15	10.44	12.72	11.84	1.51	11.67	2.9498	9.46E-12	3.31E-11	levF
AB5991_13745	2.22	2.71	1.58	15.68	42.89	43.21	2.17	33.93	3.9667	8.77E-14	3.59E-13	levE
AB5991_13750	2.05	0	0.88	15.16	18.35	23.96	0.98	19.16	4.2938	3.17E-14	1.34E-13	levD
AB5991_13755	19.18	20.8	18.38	4.97	3.54	5.5	19.45	4.67	-2.0585	9.17E-13	3.51E-12	levR
AB5991_13760	0	0	0	0	0	0	0.00	0.00	0.0000	1	1	--
AB5991_13765	18.87	18.16	23.24	2.83	2.65	2.11	20.09	2.53	-2.9893	3.55E-19	2.22E-18	aapA
AB5991_13770	14.28	14.16	9.77	3.42	4.94	0.62	12.74	2.99	-2.0892	2.54E-05	5.08E-05	yrhP
AB5991_13775	25.32	27.66	23.11	2.14	1.11	1.89	25.36	1.71	-3.8879	2.04E-24	1.90E-23	yrhO
AB5991_13780	0	0.66	0.77	0.39	0	0.39	0.48	0.26	-0.8745	1	1	sigV
AB5991_13785	0.63	1.55	1.58	2.29	1.07	0.68	1.25	1.35	0.1036	0.889313325	0.927867371	rsiV
AB5991_13795	1.86	2.28	4.65	3.38	2.53	6.72	2.93	4.21	0.5229	0.562092651	0.618987775	yrhK
AB5991_13805	50.05	31.77	48.57	1.01	2.83	3.34	43.46	2.39	-4.1827	4.24E-23	3.55E-22	fatR
AB5991_13810	9.32	27.5	11.39	3.62	2.03	1.44	16.07	2.36	-2.7655	1.06E-06	2.41E-06	yrhH

AB5991_13815	2048.49	1699.11	2221.05	129.82	134.21	157.51	1989.55	140.51	-3.8237	2.52E-38	6.32E-37	--
AB5991_13820	1204.99	668.21	1306.67	96.98	94.43	120.01	1059.96	103.81	-3.3520	2.48E-23	2.10E-22	yrzI
AB5991_13825	0	0	1.69	3.44	9.65	3.66	0.56	5.58	3.3091	6.91E-05	0.000131454	yrhG
AB5991_13830	74.45	58.46	62.86	12.25	13.96	9.54	65.26	11.92	-2.4531	5.50E-14	2.29E-13	yrhF
AB5991_13835	41.27	38.91	39.67	42.08	35.13	35.44	39.95	37.55	-0.0894	0.723343993	0.774050019	yrhE
AB5991_13840	19.09	19.24	23.61	37.44	43.04	41.72	20.65	40.73	0.9803	0.000878136	0.00150111	yrhD
AB5991_13845	1.46	2.49	2.28	9.07	7.18	5.16	2.08	7.14	1.7810	8.64E-06	1.81E-05	--
AB5991_13850	72.77	56.03	78.66	20.42	19.11	7.62	69.15	15.72	-2.1375	1.52E-08	4.06E-08	yrhC
AB5991_13855	435.07	410.2	395.74	112.26	62.29	52.86	413.67	75.80	-2.4481	5.69E-14	2.36E-13	mccB
AB5991_13860	265.84	264.72	251.45	55.95	24.49	19.06	260.67	33.17	-2.9744	5.65E-15	2.55E-14	mccA
AB5991_13865	317.09	324.74	303.55	72.87	56.3	38.51	315.13	55.89	-2.4952	8.70E-17	4.52E-16	mtnN
AB5991_13870	54.9	62.04	43.66	46.24	16.05	13.11	53.53	25.13	-1.0908	0.01004649	0.015009259	yrrT
AB5991_13875	7.97	1.63	6.63	6.75	0	4.8	5.41	3.85	-0.4908	0.575802266	0.632681461	yrzA
AB5991_13880	132.09	171.62	133.27	46.76	38.78	21.74	145.66	35.76	-2.0262	3.58E-10	1.09E-09	yrrS
AB5991_13885	26.78	36.31	24.78	2.91	3.46	2.01	29.29	2.79	-3.3903	2.44E-24	2.23E-23	pbpI
AB5991_13890	12.58	48.31	18.35	71.75	44.63	30.13	26.41	48.84	0.8867	0.055602219	0.074922434	greA
AB5991_13895	34.96	41.75	33.43	70.78	96.61	87.68	36.71	85.02	1.2116	1.35E-05	2.75E-05	udk
AB5991_13900	45.58	44.2	38.38	97.75	92.05	82.8	42.72	90.87	1.0888	2.88E-05	5.73E-05	trhP1
AB5991_13905	19.44	7.85	11.64	22.41	20.57	14.94	12.98	19.31	0.5732	0.119729535	0.15421537	trhP2
AB5991_13910	8.29	12.18	5.91	3.91	5.06	2.99	8.79	3.99	-1.1412	0.00954577	0.014331287	trmR
AB5991_13915	8.85	18.69	10.71	13.25	13.08	11.2	12.75	12.51	-0.0274	0.98271539	1	mltG
AB5991_13920	30.12	25.89	26.73	138.72	162.43	89.5	27.58	130.22	2.2392	1.05E-11	3.65E-11	yrzB
AB5991_13925	12.57	15.12	13.44	47.14	69.7	52.55	13.71	56.46	2.0421	4.98E-10	1.49E-09	yrrK
AB5991_13930	10.15	11.19	20.99	51.54	79.92	46.9	14.11	59.45	2.0750	1.38E-07	3.38E-07	yrzL
AB5991_13935	15.7	12.96	12.46	45.55	61.32	49.94	13.71	52.27	1.9311	3.05E-12	1.11E-11	alaS
AB5991_13940	6.98	4.69	6.01	1.48	1.21	1.66	5.89	1.45	-2.0230	1.81E-06	4.01E-06	yrrI

AB5991_13945	90.75	143.41	108.98	2.43	3.03	1.07	114.38	2.18	-5.7156	3.59E-44	1.28E-42	glnQ
AB5991_13950	301.69	544.67	317.45	10.52	8.5	7.86	387.94	8.96	-5.4362	8.40E-48	4.12E-46	glnH
AB5991_13955	28.04	46.39	44.54	2.72	1.13	1.2	39.66	1.68	-4.5582	1.40E-24	1.31E-23	glnM
AB5991_13960	29.16	40.41	42.37	1.8	1.12	0	37.31	0.97	-5.2606	1.93E-29	2.54E-28	glnP
AB5991_13965	27.39	20.11	41	8.94	12.54	23.72	29.50	15.07	-0.9694	0.042537556	0.058227037	yrzQ
AB5991_13970	134.62	93.34	111.75	25.6	18.2	28.53	113.24	24.11	-2.2316	3.57E-11	1.19E-10	yrzR
AB5991_13975	335.33	269.94	360.08	116.45	117.73	130.07	321.78	121.42	-1.4061	8.41E-08	2.10E-07	yrrD
AB5991_13980	6.64	9.83	10.16	35.76	32.16	24.08	8.88	30.67	1.7886	5.89E-09	1.62E-08	yrrC
AB5991_13985	9.61	2.67	6.54	24.37	20.14	17.01	6.27	20.51	1.7088	2.08E-05	4.18E-05	yrrB
AB5991_13990	51.5	47.88	52.65	127.35	97.25	75.39	50.68	100.00	0.9806	0.000515689	0.000905306	mnmA
AB5991_13995	112.89	139.16	100.38	73.29	56.8	32.78	117.48	54.29	-1.1136	0.000481086	0.000845681	iscS1
AB5991_14000	98.83	124.96	105.22	14.61	12.79	7.98	109.67	11.79	-3.2171	9.70E-23	7.94E-22	cymR
AB5991_14005	22.13	54.79	24.73	13.82	8.72	6.34	33.88	9.63	-1.8155	8.15E-06	1.71E-05	yrvN
AB5991_14010	34.97	45.12	35.63	36.75	27.17	24.04	38.57	29.32	-0.3957	0.171495994	0.21281581	tcdA
AB5991_14020	30.28	40.3	28.58	68.95	69.28	65.33	33.05	67.85	1.0376	8.90E-05	0.000168026	aspS
AB5991_14025	2.13	7.29	3.94	10.48	11.69	6.14	4.45	9.44	1.0834	0.011430266	0.016942735	hisS
AB5991_14030	0	0	1.13	0	0	0	0.38	0.00	-8.5571	1	1	yrzK
AB5991_14035	4.76	5.54	5.09	37.75	43.01	28.65	5.13	36.47	2.8297	3.64E-20	2.49E-19	yrvJ
AB5991_14040	9.84	20.32	17.53	52.6	49.22	30.61	15.90	44.14	1.4735	5.12E-05	9.88E-05	dtd
AB5991_14045	46.64	65.02	43.04	79.7	74.08	51.29	51.57	68.36	0.4066	0.155293142	0.194594214	relA
AB5991_14050	11.27	11.65	10.55	27.59	24.03	12.2	11.16	21.27	0.9311	0.013614098	0.020067403	apt
AB5991_14055	11.56	13.92	13.75	28.23	28.75	21.3	13.08	26.09	0.9967	0.000394818	0.000701173	recJ
AB5991_14060	0	5.12	1.79	2.43	11.36	26.57	2.30	13.45	2.5462	0.005348064	0.008286055	yrvD
AB5991_14065	33.03	31.99	29.89	12.24	13.67	5.11	31.64	10.34	-1.6134	9.35E-06	1.94E-05	yrvC
AB5991_14070	11.76	18.74	13.71	54.87	53.09	36.94	14.74	48.30	1.7126	1.01E-08	2.73E-08	secDF
AB5991_14075	0.61	1.12	3.25	4.63	6.19	3.29	1.66	4.70	1.5025	0.046898663	0.063648185	comN

AB5991_14080	3.71	7.03	7.57	0.63	0.35	0.25	6.10	0.41	-3.8959	6.93E-13	2.68E-12	spoVB
AB5991_14085	2.75	1.01	5	1.5	0.56	0.89	2.92	0.98	-1.5702	0.025408258	0.03598295	yrbG
AB5991_14090	22.71	45.95	33.21	2.02	0.47	0	33.96	0.83	-5.3544	2.85E-19	1.80E-18	yrzE
AB5991_14095	12.86	19.89	13.75	110.44	97.84	79.14	15.50	95.81	2.6279	1.99E-14	8.58E-14	yrbF
AB5991_14100	21.61	22.59	20.91	42.2	37.72	21.51	21.70	33.81	0.6395	0.035913143	0.049724927	tgt
AB5991_14105	10.89	15.16	11.65	29.61	31.82	18.25	12.57	26.56	1.0797	0.000642252	0.001115652	queA
AB5991_14110	0.9	0	0	2.93	0.92	0.97	0.30	1.61	2.4210	0.243697507	0.294140669	yrzS
AB5991_14115	3.06	5.61	3.27	10.17	9.88	5.65	3.98	8.57	1.1060	0.005271125	0.008179723	ruvB
AB5991_14120	2.09	7.67	2.87	0.97	1.82	0.97	4.21	1.25	-1.7481	0.016019448	0.023405348	ruvA
AB5991_14125	11.27	14.88	19.97	52.88	64.19	74.37	15.37	63.81	2.0534	1.89E-10	5.90E-10	bofC
AB5991_14130	10.36	11.16	8.72	51.4	64.98	74.64	10.08	63.67	2.6592	4.91E-18	2.83E-17	csbX
AB5991_14135	8.46	20.7	14.88	13.22	12.01	13.92	14.68	13.05	-0.1698	0.670586106	0.72475527	yrbE
AB5991_14140	69.73	102.44	64.43	37.01	38.04	34.72	78.87	36.59	-1.1080	0.000103262	0.000193331	yrzF
AB5991_14145	1.47	8.09	1.57	0	0	0	3.71	0.00	-11.8572	0.043576397	0.059508254	--
AB5991_14155	96.01	194.28	102.67	15.98	11.47	3.18	130.99	10.21	-3.6814	3.94E-15	1.80E-14	yrzH
AB5991_14160	8.97	14.12	2.74	0	0	0	8.61	0.00	-13.0718	0.00029741	0.000531749	yrzT
AB5991_14165	50.93	57.71	49.82	14.05	8.85	9.95	52.82	10.95	-2.2702	4.57E-14	1.92E-13	yrbD
AB5991_14170	4.5	11.94	4.01	2.18	5.09	4.06	6.82	3.78	-0.8520	0.126112756	0.161911981	yrbC
AB5991_14175	801.71	782.75	843.43	50.38	89.67	103.57	809.30	81.21	-3.3170	4.80E-24	4.28E-23	coxA
AB5991_14180	811.04	1266.54	1004.33	112.48	98.3	102.37	1027.30	104.38	-3.2989	2.37E-29	3.07E-28	safA
AB5991_14185	168.5	202.07	202.37	17.58	15.45	14.85	190.98	15.96	-3.5809	2.25E-35	4.64E-34	nadA
AB5991_14190	2.08	1.53	3.55	1.58	0.85	1.12	2.39	1.18	-1.0121	0.094362281	0.123545208	nadC
AB5991_14195	5.66	2.7	4.12	1.48	1.04	1.23	4.16	1.25	-1.7347	4.53E-05	8.82E-05	nadB
AB5991_14200	12.78	10.62	15.95	7.12	6.66	1.98	13.12	5.25	-1.3201	0.001402559	0.002346067	nifS
AB5991_14205	7.32	14.06	10.32	20.27	22.02	8.29	10.57	16.86	0.6741	0.111484003	0.144438165	nadR
AB5991_14210	0.21	1.93	1.35	16.04	15.22	7.07	1.16	12.78	3.4572	3.47E-10	1.06E-09	pheA

AB5991_14215	5.29	2.24	2.61	44.72	27.76	17.63	3.38	30.04	3.1516	1.78E-10	5.58E-10	yszB
AB5991_14220	24.86	27.34	24.03	81.41	72.9	43.48	25.41	65.93	1.3755	7.48E-06	1.57E-05	obg
AB5991_14225	15.92	17.2	13.02	19.01	13.98	6.76	15.38	13.25	-0.2151	0.592051471	0.649007111	spo0B
AB5991_14230	0	0	0	0	0	0	0.00	0.00	0.0000	1	1	--
AB5991_14235	154.75	145.57	151.92	936	823.62	586.98	150.75	782.20	2.3754	1.09E-16	5.63E-16	rpmA
AB5991_14240	159.96	168.39	184.74	1103.52	932.28	783.8	171.03	939.87	2.4582	9.52E-19	5.75E-18	ysxB
AB5991_14245	98.27	125.67	130.11	644.45	527.47	438.18	118.02	536.70	2.1851	1.67E-14	7.25E-14	rplU
AB5991_14250	21.06	28.33	19.84	4.53	5.52	3.16	23.08	4.40	-2.3898	6.29E-12	2.23E-11	spoIVFB
AB5991_14255	80.03	142.36	112.09	12.86	8.56	6.65	111.49	9.36	-3.5748	1.40E-22	1.14E-21	spoIVFA
AB5991_14260	152.53	185.07	151.14	84.77	83.2	67.16	162.91	78.38	-1.0556	5.68E-05	0.000109178	minD
AB5991_14265	126.87	143.28	133.12	62.93	58.08	48.84	134.42	56.62	-1.2475	2.34E-06	5.15E-06	minC
AB5991_14270	20.9	15.35	22.72	28.03	24.81	16.59	19.66	23.14	0.2356	0.506002538	0.566010163	mreD
AB5991_14275	97.93	140.66	84.36	132.4	108.52	83.37	107.65	108.10	0.0060	0.981805885	1	mreC
AB5991_14280	47.59	70.7	43.65	70.57	56.78	38.78	53.98	55.38	0.0369	0.904444821	0.942169566	mreB
AB5991_14285	13.5	13.83	11.11	16.66	28.81	20.52	12.81	22.00	0.7796	0.016928851	0.024678585	ysxA
AB5991_14290	45.66	80.93	25.43	40.01	43.89	45.65	50.67	43.18	-0.2308	0.551617188	0.608633468	maf
AB5991_14295	11.04	12.62	7.16	3.35	4.79	1.37	10.27	3.17	-1.6963	0.000125449	0.000232676	spoIIB
AB5991_14300	0.24	0	0	0.26	0.49	0	0.08	0.25	1.6439	0.639004309	0.69482095	comC
AB5991_14305	25.44	25.67	18.84	24.63	33.01	26.33	23.32	27.99	0.2635	0.343624817	0.400156642	folC
AB5991_14310	24.69	30.14	18.94	39.12	40.23	30.28	24.59	36.54	0.5715	0.040531343	0.055634277	valS
AB5991_14315	8.47	3.46	3.02	0	0.96	2.04	4.98	1.00	-2.3171	0.031089882	0.043486411	yszA
AB5991_14320	893.18	1226.64	869.26	55.95	53.61	51.68	996.36	53.75	-4.2124	1.03E-44	3.83E-43	ysxE
AB5991_14325	1215.18	1610.11	1145.84	75.92	58.01	55.68	1323.71	63.20	-4.3884	5.85E-47	2.58E-45	spoVID
AB5991_14330	84.29	112.17	72.35	544.44	602.1	546.58	89.60	564.37	2.6550	1.08E-20	7.75E-20	hemL
AB5991_14335	21.32	23.15	20.62	131.26	146.6	104.95	21.70	127.60	2.5561	2.74E-19	1.73E-18	hemB
AB5991_14340	14.89	15.56	16.41	44.1	48.26	38.19	15.62	43.52	1.4782	1.28E-07	3.15E-07	hemD

AB5991_14345	23.53	27.39	20.86	46.8	52.17	31.47	23.93	43.48	0.8617	0.003217497	0.005139452	hemC
AB5991_14350	15.88	13.58	15.12	18.45	23.24	9.65	14.86	17.11	0.2037	0.577163301	0.634001513	hemX
AB5991_14355	258.04	404.19	260.97	112.23	120.62	113.71	307.73	115.52	-1.4135	5.54E-07	1.30E-06	hemA
AB5991_14360	7.98	8.66	7.37	8.29	2.96	3.54	8.00	4.93	-0.6990	0.145961241	0.183828762	ysxD
AB5991_14365	30.43	28.22	33.2	106.31	117.32	118.13	30.62	113.92	1.8956	5.41E-12	1.93E-11	engB
AB5991_14370	25.11	38.26	28.77	92.16	108.56	104.1	30.71	101.61	1.7261	3.56E-10	1.08E-09	lon1
AB5991_14375	108.08	151.24	131.31	13.86	12.2	8.14	130.21	11.40	-3.5137	9.19E-29	1.15E-27	lon2
AB5991_14380	58.53	115.89	71.47	128.25	90.3	60.57	81.96	93.04	0.1829	0.588834169	0.645749927	clpX
AB5991_14385	15.45	19.26	17.59	214.93	212.09	89.16	17.43	172.06	3.3030	3.49E-19	2.19E-18	tig
AB5991_14390	1.98	11.23	3.85	9.58	8.05	6.81	5.69	8.15	0.5186	0.296800592	0.350251159	ysoA
AB5991_14395	76.22	387.21	60.89	387.26	378.04	452.3	174.77	405.87	1.2155	0.02246361	0.032145223	leuD
AB5991_14400	89.29	357.85	74.24	407.98	390.6	474.19	173.79	424.26	1.2876	0.006985743	0.010677593	leuC
AB5991_14405	21.24	81.61	13.91	151.11	146.6	186.75	38.92	161.49	2.0528	4.62E-05	8.99E-05	leuB
AB5991_14410	21.13	68.85	14.52	299.86	314.4	388.81	34.83	334.36	3.2628	1.94E-11	6.61E-11	leuA
AB5991_14415	42.51	208.36	37.38	457.92	475.72	573.86	96.08	502.50	2.3868	7.83E-06	1.65E-05	ilvC
AB5991_14420	8.01	26.22	7.08	82.95	94.28	120.26	13.77	99.16	2.8483	2.56E-11	8.61E-11	ilvH
AB5991_14425	14.98	46.56	12.89	91.28	78.06	89.27	24.81	86.20	1.7968	1.73E-05	3.51E-05	ilvB
AB5991_14430	174.83	115.57	236.44	17.55	37.78	52.41	175.61	35.91	-2.2898	7.49E-09	2.05E-08	ysnD
AB5991_14440	63.97	61.95	58.11	238.04	245.72	206.33	61.34	230.03	1.9068	2.92E-13	1.15E-12	ysnF
AB5991_14445	0.45	0.62	0.61	1.39	0.97	0.74	0.56	1.03	0.8838	0.223304965	0.270926983	insK
AB5991_14455	1.23	2.26	1.76	2.08	2.51	2.82	1.75	2.47	0.4972	0.269955462	0.321630104	hsdS
AB5991_14460	2.78	3.48	2.7	4.12	2.83	4.24	2.99	3.73	0.3206	0.384061146	0.44103725	hsdM
AB5991_14465	58.05	39.12	52.74	15.39	12.79	14.95	49.97	14.38	-1.7973	7.37E-11	2.39E-10	hsdR
AB5991_14470	508.98	371.1	396.32	12.06	15.67	9.14	425.47	12.29	-5.1135	7.32E-50	4.15E-48	--
AB5991_14475	12.17	15.64	18.22	0	0	0	15.34	0.00	-13.9053	4.54E-14	1.90E-13	--
AB5991_14480	21.44	11.25	26.21	0	0	1.11	19.63	0.37	-5.7296	1.08E-09	3.16E-09	--

AB5991_14485	68.76	51.41	80.54	0.58	1.08	0.96	66.90	0.87	-6.2594	6.96E-49	3.68E-47	xerC
AB5991_14495	7.44	14.97	12.89	93.67	90.18	74.81	11.77	86.22	2.8733	2.70E-18	1.59E-17	ysnB
AB5991_14500	16.35	35.58	21.69	130.07	141.74	115.36	24.54	129.06	2.3948	6.19E-13	2.40E-12	ysnA
AB5991_14505	4.65	13.94	5.5	57	56.58	53.02	8.03	55.53	2.7899	9.45E-14	3.85E-13	rph
AB5991_14510	159.41	453.98	170.65	22.14	13.7	11.91	261.35	15.92	-4.0374	3.58E-19	2.24E-18	gerM
AB5991_14515	3590.93	4030.09	3550.56	96.73	133.2	150.99	3723.86	126.97	-4.8742	4.43E-57	3.74E-55	racE
AB5991_14520	2385.81	2527.93	2224.85	71.32	84.26	72.76	2379.53	76.11	-4.9664	1.08E-61	1.47E-59	ysmB
AB5991_14525	12040.3	9705.94	13690.35	1056.29	1627.01	2148.8	11812.20	1610.70	-2.8745	1.70E-19	1.10E-18	gerE
AB5991_14530	10.99	23.17	10.88	36.31	43.92	52	15.01	44.08	1.5538	1.69E-05	3.43E-05	ysmA
AB5991_14535	302.91	554.02	278.02	933.11	1545.3	1399.42	378.32	1292.61	1.7726	1.16E-07	2.87E-07	sdhB
AB5991_14540	159.91	273.47	138.41	384.23	614.03	514.1	190.60	504.12	1.4032	1.30E-05	2.66E-05	sdhA
AB5991_14545	5.05	10.35	9.2	40.67	45.91	23.13	8.20	36.57	2.1570	3.48E-08	9.00E-08	sdhC
AB5991_14550	36.39	90.58	40.22	17.59	16.05	12.69	55.73	15.44	-1.8515	4.69E-06	1.01E-05	yslB
AB5991_14555	12.82	17.58	13.71	12.82	15.29	7.65	14.70	11.92	-0.3028	0.375657017	0.433391637	lysC
AB5991_14560	159.34	209.65	168.44	25.17	24.18	19.97	179.14	23.11	-2.9547	2.33E-25	2.34E-24	uvrC
AB5991_14565	195.67	486.77	248.52	226.54	207.32	180.75	310.32	204.87	-0.5990	0.077975139	0.103144329	trxA
AB5991_14570	45.79	78.73	48.06	60.51	42.78	43.92	57.53	49.07	-0.2294	0.450600351	0.508767129	abf2
AB5991_14575	705.44	693.3	562.88	2668.72	2570.91	2714.45	653.87	2651.36	2.0196	1.25E-15	5.86E-15	etfA
AB5991_14580	414.51	516.7	375.1	1914.5	1978.15	1939.93	435.44	1944.19	2.1586	3.37E-16	1.66E-15	etfB
AB5991_14585	150.04	195.63	165.18	955.83	1138.79	1103.71	170.28	1066.11	2.6463	2.91E-22	2.34E-21	fadB
AB5991_14590	94.55	166.23	117.96	535.98	634.27	635.82	126.25	602.02	2.2536	2.83E-14	1.20E-13	fadR
AB5991_14595	84.41	90.12	93.37	515.57	601.39	577.8	89.30	564.92	2.6613	9.07E-24	7.94E-23	lcfA
AB5991_14600	16.51	28.68	18.61	9.71	10.45	9.18	21.27	9.78	-1.1207	0.003745351	0.005923054	yshE
AB5991_14605	54.12	56.3	45.17	80.2	84.49	63.89	51.86	76.19	0.5549	0.033800755	0.046914645	mutSB
AB5991_14610	34.08	37.01	32.5	59.67	61.75	52.31	34.53	57.91	0.7460	0.003468841	0.005512111	polX
AB5991_14615	8.8	11.19	4.34	10.68	8.27	8.79	8.11	9.25	0.1892	0.668180555	0.72278534	yshB

AB5991_14620	5.6	9	4.5	5.33	9.27	6.07	6.37	6.89	0.1140	0.817860774	0.861237107	zapA
AB5991_14625	8.85	16.97	10.91	10.05	10.38	7.92	12.24	9.45	-0.3736	0.316918821	0.371892623	rnhC
AB5991_14630	33.75	37.79	30.01	178.84	159.28	130.52	33.85	156.21	2.2063	1.94E-15	8.98E-15	pheT
AB5991_14635	12.57	25.01	15.31	42.36	38.57	17.39	17.63	32.77	0.8945	0.017196114	0.025049805	pheS
AB5991_14640	0.87	0	0.93	0	0	0	0.60	0.00	-9.2288	0.509596993	0.568309168	--
AB5991_14645	66.3	75.97	70.38	12.37	8.62	9.43	70.88	10.14	-2.8054	7.59E-22	5.83E-21	ysgA
AB5991_14650	11.72	15.37	11.63	7.28	1.7	0	12.91	2.99	-2.1083	0.001684879	0.002785452	sspI
AB5991_14655	6.69	8.39	7.51	4.26	5.82	4.42	7.53	4.83	-0.6396	0.064178199	0.085924352	ysfB
AB5991_14660	61.27	56.84	58.96	21.01	31.12	27.56	59.02	26.56	-1.1518	2.98E-05	5.91E-05	glcD
AB5991_14665	71.89	70.85	68.63	37.99	49.74	60.09	70.46	49.27	-0.5159	0.055932296	0.075316089	glcF
AB5991_14670	7.59	6.51	9.2	23.13	31.95	29.05	7.77	28.04	1.8523	6.60E-07	1.53E-06	ysfE
AB5991_14680	6.34	3.51	7.42	17.61	22.01	22.65	5.76	20.76	1.8503	1.06E-08	2.85E-08	cstA
AB5991_14685	14.91	16.34	10.67	94.96	143.45	150.88	13.97	129.76	3.2151	3.30E-24	2.99E-23	abfA
AB5991_14690	7.05	4.71	4.57	17.66	37.62	42.32	5.44	32.53	2.5794	2.03E-10	6.31E-10	araQ
AB5991_14695	9.98	7.4	9.64	30.89	48.43	48.6	9.01	42.64	2.2431	2.32E-12	8.56E-12	araP
AB5991_14700	10.41	8.92	6.68	34.58	49.03	45.83	8.67	43.15	2.3151	3.78E-14	1.59E-13	araN
AB5991_14705	5.95	6.44	4.4	35.17	40.21	43.43	5.60	39.60	2.8230	1.40E-20	9.89E-20	egsA
AB5991_14710	11.48	6.89	5.19	54.25	59.3	55.19	7.85	56.25	2.8404	9.52E-18	5.36E-17	araL
AB5991_14715	7.6	9.62	9.8	53.56	48.52	52.46	9.01	51.51	2.5159	7.31E-17	3.81E-16	araD
AB5991_14720	8.7	7.89	6.66	44.27	51.81	50.46	7.75	48.85	2.6560	3.21E-19	2.02E-18	araB
AB5991_14725	8.36	4.67	6.09	31.91	46.27	39.89	6.37	39.36	2.6265	2.23E-16	1.11E-15	araA
AB5991_14735	32.95	36.67	31.15	78.02	78.6	74.05	33.59	76.89	1.1948	6.28E-06	1.33E-05	ysdC
AB5991_14740	127.86	215.35	133.29	71.53	83.79	72.69	158.83	76.00	-1.0634	0.000424259	0.000749769	ysdB
AB5991_14745	52.89	49.17	57.27	15.29	12.26	10.87	53.11	12.81	-2.0521	7.31E-10	2.16E-09	ysdA
AB5991_14750	541.24	474.79	575.59	602.85	784.38	731.01	530.54	706.08	0.4124	0.115161738	0.148717809	rplT
AB5991_14755	168.16	84.21	169.25	67.48	102.5	67.17	140.54	79.05	-0.8301	0.013949128	0.020530759	rpmI

AB5991_14760	631.24	599.56	765.77	303.91	410.21	383.83	665.52	365.98	-0.8627	0.001357996	0.002278244	infC
AB5991_14765	77.75	57.95	63.51	4.06	4.38	6.21	66.40	4.88	-3.7653	4.66E-28	5.56E-27	yscB
AB5991_14770	6.49	8.11	6.67	5.93	7.14	5.06	7.09	6.04	-0.2304	0.57736536	0.634048076	lrgB
AB5991_14775	3.69	5.27	3.51	1.78	0.83	0.44	4.16	1.02	-2.0316	0.002734251	0.004426298	lrgA
AB5991_14780	19.42	18.74	13.84	10.29	10.64	10.24	17.33	10.39	-0.7384	0.017853653	0.025950533	lytT
AB5991_14785	20.69	20.86	22.34	13.46	12.39	9.55	21.30	11.80	-0.8518	0.00138676	0.002323555	lytS
AB5991_14790	47.78	38.15	49.62	13.31	13.16	8.25	45.18	11.57	-1.9650	1.34E-10	4.23E-10	serB
AB5991_14795	33.21	39.68	26.71	79.16	65.7	70.08	33.20	71.65	1.1097	3.53E-05	6.96E-05	thrS
AB5991_14800	11.96	19.22	15.08	22.54	22.61	9.25	15.42	18.13	0.2338	0.534866017	0.591795194	ytxC
AB5991_14805	15.2	14.99	9.94	24.19	21.78	11.89	13.38	19.29	0.5279	0.150812868	0.189485438	ytxB
AB5991_14810	17.57	16.67	12.39	15.96	15.33	9.2	15.54	13.50	-0.2037	0.555801624	0.612509453	dnaI
AB5991_14815	18.85	18.95	16.62	8.17	8.95	3.45	18.14	6.86	-1.4036	6.37E-05	0.000121669	dnaB
AB5991_14820	76	95.45	73.27	29.55	23.65	25.15	81.57	26.12	-1.6431	7.71E-09	2.11E-08	nrdR
AB5991_14825	5259.75	6709.23	5163.63	2153.62	2288.14	2355.64	5710.87	2265.80	-1.3337	1.29E-07	3.17E-07	speH
AB5991_14830	9499.14	12188.77	9544.71	2699.86	2576.86	2421.38	10410.87	2566.03	-2.0205	9.39E-16	4.44E-15	gapB
AB5991_14835	37.95	54.88	33.48	4.13	2.9	3.08	42.10	3.37	-3.6431	1.15E-17	6.40E-17	ytcD
AB5991_14840	3.19	0.56	1.62	0.66	0.46	0.99	1.79	0.70	-1.3477	0.043098137	0.05891315	ytbD
AB5991_14845	4.5	2.36	2.29	1.17	2.4	1.39	3.05	1.65	-0.8834	0.109844262	0.142588697	ytbE
AB5991_14850	111.37	59.78	102.5	23.83	53.27	49.08	91.22	42.06	-1.1168	0.001619267	0.002681446	coaE
AB5991_14860	33.93	34.75	30.01	69.55	68.85	72.52	32.90	70.31	1.0957	3.45E-05	6.81E-05	mutM
AB5991_14865	17.78	18.33	16.46	35.4	35.98	31.46	17.52	34.28	0.9681	0.000163306	0.000298705	polA
AB5991_14870	1811.65	2154.62	1929.27	96.71	109.53	99.85	1965.18	102.03	-4.2676	1.98E-53	1.43E-51	phoR
AB5991_14875	1197.24	1353.27	1225	80.48	112.97	86.87	1258.50	93.44	-3.7515	1.83E-38	4.65E-37	phoP
AB5991_14880	167.47	171.42	131.95	2078.65	3046.98	2784.89	156.95	2636.84	4.0705	4.19E-42	1.29E-40	mdh
AB5991_14885	66.64	59.23	55.62	998.05	1443.61	1345.94	60.50	1262.53	4.3833	2.14E-46	9.14E-45	icd
AB5991_14890	51.69	72.37	50.61	1533.47	1756.73	1668.28	58.22	1652.83	4.8272	3.35E-56	2.72E-54	citZ

AB5991_14895	118.17	103.49	115.14	14.37	30.46	42.5	112.27	29.11	-1.9473	3.02E-08	7.84E-08	ytwI
AB5991_14900	79.52	75.24	92.32	1.23	0.49	0.53	82.36	0.75	-6.7789	1.20E-56	9.90E-55	ytvI
AB5991_14905	16	21.61	11.58	76.79	69.94	60.63	16.40	69.12	2.0757	2.19E-10	6.81E-10	ytzA
AB5991_14910	52.02	53.05	52.23	479.38	544.97	503.96	52.43	509.44	3.2803	4.38E-34	8.01E-33	pyk
AB5991_14915	6.4	11.75	8.66	133.51	135.67	113.32	8.94	127.50	3.8346	1.95E-32	3.09E-31	pfkA
AB5991_14920	38.81	34.95	28.66	71.56	70.54	42.01	34.14	61.37	0.8461	0.005813511	0.008972615	accA
AB5991_14925	27.95	33.46	35.2	87.82	81.97	49.53	32.20	73.11	1.1828	0.000128543	0.000238191	accD
AB5991_14930	255.8	352.89	264.78	62.02	46.55	36.66	291.16	48.41	-2.5884	2.30E-17	1.24E-16	ytsJ
AB5991_14935	42.76	60.67	46.65	17.56	20.6	16.13	50.03	18.10	-1.4670	7.31E-08	1.84E-07	dnaE
AB5991_14940	28.54	41.73	37.3	0	0.54	2.29	35.86	0.94	-5.2483	2.98E-21	2.18E-20	ytrH
AB5991_14945	48.41	30.95	51.39	2.73	1.82	1.55	43.58	2.03	-4.4219	2.19E-24	2.02E-23	ytrI
AB5991_14950	96.02	69.14	150	259.04	224.2	190.57	105.05	224.60	1.0963	0.000650669	0.0011283	ytzJ
AB5991_14955	46.63	87.73	64.84	18.16	13.87	7.89	66.40	13.31	-2.3190	1.16E-10	3.70E-10	nrnA
AB5991_14960	10126.13	7006.94	11463.02	72.01	103.82	133.67	9532.03	103.17	-6.5297	4.57E-67	9.56E-65	ytpI
AB5991_14965	15.61	29.17	24.75	23.98	22.72	14.38	23.18	20.36	-0.1869	0.57164258	0.628458661	ytoI
AB5991_14970	1.46	2.49	2.28	9.07	7.18	5.16	2.08	7.14	1.7810	8.63E-06	1.81E-05	--
AB5991_14975	25.63	58.23	25.43	307.23	306.33	261.17	36.43	291.58	3.0007	1.88E-17	1.02E-16	ytkL
AB5991_14980	1.88	12.1	6.04	61.18	74.01	44.58	6.67	59.92	3.1666	9.49E-12	3.32E-11	ytkK
AB5991_14985	0	0	1.43	0	6.81	0	0.48	2.27	2.2516	0.290286643	0.343228972	ytzD
AB5991_14990	35.34	32.57	30.4	397.84	270.5	237.59	32.77	301.98	3.2040	3.46E-25	3.39E-24	argH
AB5991_14995	24.46	27.66	25.04	366.4	218.57	192.27	25.72	259.08	3.3324	3.08E-24	2.80E-23	argG
AB5991_15000	4.23	9.06	4.9	10.35	6.1	4.96	6.06	7.14	0.2351	0.605290103	0.661786068	moaB
AB5991_15005	34.23	40.79	38.72	39.71	35.61	21.9	37.91	32.41	-0.2264	0.448819275	0.507301347	ackA
AB5991_15010	0	0	0	0	0	0	0.00	0.00	0.0000	1	1	--
AB5991_15015	12.23	15.09	15.81	47.46	36.79	21.74	14.38	35.33	1.2972	0.000109025	0.000203544	ytxK
AB5991_15020	16.14	22.39	13.81	64.75	69.35	49.69	17.45	61.26	1.8121	5.79E-09	1.60E-08	tpx

AB5991_15025	49.15	89.52	71.21	11.21	11.7	7.72	69.96	10.21	-2.7765	8.19E-15	3.64E-14	ytfJ
AB5991_15030	4.51	8.29	6.53	2.31	2.43	0.57	6.44	1.77	-1.8641	0.00041947	0.000741966	ytfI
AB5991_15035	31.77	44.25	31.24	15.09	16.35	9.09	35.75	13.51	-1.4041	3.33E-05	6.59E-05	yteJ
AB5991_15040	8.79	13.83	7.29	3.32	3.83	3.11	9.97	3.42	-1.5436	7.56E-05	0.00014328	sppA
AB5991_15045	37.99	51.19	28.13	192.18	216.68	167.92	39.10	192.26	2.2977	1.81E-14	7.83E-14	nadK2
AB5991_15050	10.34	11.69	14.47	51.8	74.74	57.84	12.17	61.46	2.3367	8.71E-15	3.85E-14	ytcJ
AB5991_15055	2.5	2.92	2.67	18.17	14.23	10.09	2.70	14.16	2.3929	3.07E-11	1.03E-10	ytcI
AB5991_15060	2317.03	1966.05	2847.86	145.1	140.16	150.01	2376.98	145.09	-4.0341	6.08E-40	1.70E-38	sspA
AB5991_15065	95.77	115.31	113.96	13.37	12.97	10.55	108.35	12.30	-3.1393	2.23E-27	2.59E-26	thiI
AB5991_15070	56.15	76.17	55.32	4.63	5.62	3.41	62.55	4.55	-3.7799	2.42E-30	3.40E-29	iscS2
AB5991_15075	12.29	7.94	11.99	0.73	1.1	0.44	10.74	0.76	-3.8272	5.03E-18	2.89E-17	braB
AB5991_15080	12.2	19.26	11.79	41.32	37.03	25.95	14.42	34.77	1.2700	4.92E-05	9.52E-05	ezrA
AB5991_15085	42.56	55.93	53.41	11.69	13.22	6.06	50.63	10.32	-2.2942	5.02E-12	1.80E-11	hisK
AB5991_15090	223.91	318.06	230.16	176.1	131.78	130.44	257.38	146.11	-0.8169	0.003100696	0.004970877	yttP
AB5991_15095	93.68	138.96	75.43	12.79	14.58	4.77	102.69	10.71	-3.2608	2.64E-16	1.31E-15	ytsP
AB5991_15100	60.25	77.25	66.32	44.17	46.31	45.99	67.94	45.49	-0.5787	0.023453085	0.033452658	ytrP
AB5991_15105	90.82	85.86	93.28	314.27	344.43	243.04	89.99	300.58	1.7400	1.18E-10	3.76E-10	rpsD
AB5991_15110	4.2	10.29	4.5	13.71	17.11	13.65	6.33	14.82	1.2276	0.042226289	0.057840839	--
AB5991_15115	7.41	8.11	5.79	26.8	28.41	21.89	7.10	25.70	1.8552	4.94E-10	1.48E-09	tyrS1
AB5991_15120	62.88	114.68	53.52	218.31	287.23	276.25	77.03	260.60	1.7584	7.75E-08	1.94E-07	acsA
AB5991_15125	79.67	81.27	68.71	14.29	22.96	23.49	76.55	20.25	-1.9187	4.85E-11	1.60E-10	acuA
AB5991_15130	165.06	175.46	153.74	33.22	41.36	37.92	164.75	37.50	-2.1353	3.94E-15	1.80E-14	acuB
AB5991_15135	34.16	29.65	28.23	107.24	94.03	97.66	30.68	99.64	1.6995	1.34E-10	4.23E-10	acuC
AB5991_15140	3.47	15.02	6.36	1.35	0.25	0.54	8.28	0.71	-3.5376	1.39E-06	3.12E-06	ytxE
AB5991_15145	1.99	5.67	3.07	7.2	3.59	2.87	3.58	4.55	0.3483	0.474230038	0.534080398	ytxD
AB5991_15150	25.72	23.45	25.58	52.81	34.05	19.08	24.92	35.31	0.5031	0.147608402	0.185726542	ccpA

AB5991_15155	1070.9	1314.31	1129.07	97.83	79.25	54.68	1171.43	77.25	-3.9225	3.07E-35	6.28E-34	aroA
AB5991_15160	824.15	824.14	601.75	3617.21	3988.59	3642.17	750.01	3749.32	2.3216	2.11E-17	1.14E-16	ytxJ
AB5991_15165	1032.57	1098.29	869.82	5807.33	6698.36	5782.31	1000.23	6096.00	2.6075	1.50E-23	1.28E-22	ytxH
AB5991_15170	147.85	215.77	149.88	2322.72	2828.53	2538.97	171.17	2563.41	3.9046	2.01E-38	5.08E-37	ytxG
AB5991_15175	36.87	35.51	44.79	99.12	86.1	51.97	39.06	79.06	1.0174	0.000729183	0.001258951	murC
AB5991_15180	15.79	14.56	16.28	33.59	33.5	22.86	15.54	29.98	0.9479	0.00057998	0.001011907	sftA
AB5991_15185	28.93	33.96	23.28	48.98	64.05	37.78	28.72	50.27	0.8075	0.008324558	0.012593075	ytpR
AB5991_15190	24.32	26.22	23.62	84.94	81.08	69.33	24.72	78.45	1.6661	1.08E-09	3.16E-09	ytpQ
AB5991_15195	5.02	4.1	4.77	31.55	26.69	21.14	4.63	26.46	2.5147	3.41E-09	9.59E-09	ytpP
AB5991_15200	0	0	0	0	0	0	0.00	0.00	0.0000	1	1	--
AB5991_15205	5.26	14.85	6.05	100.27	100.83	103.3	8.72	101.47	3.5405	4.59E-22	3.63E-21	ytoQ
AB5991_15210	8.25	15.45	9.72	30.2	21.07	15.85	11.14	22.37	1.0060	0.004059555	0.006389414	ytoP
AB5991_15215	46.04	57.4	65.04	97.67	98.34	52.3	56.16	82.77	0.5596	0.083733437	0.110320331	ytzB
AB5991_15220	68.96	79.61	67.39	448.75	532.95	528.44	71.99	503.38	2.8058	2.47E-25	2.45E-24	malS
AB5991_15225	35.89	38.05	25.13	285.34	354.44	327.49	33.02	322.42	3.2874	1.50E-28	1.85E-27	ytnP
AB5991_15230	45.61	84.26	57.81	18.98	13.18	9.14	62.56	13.77	-2.1841	8.91E-10	2.62E-09	trmB
AB5991_15235	7.77	4.76	11.08	2.11	0	1.4	7.87	1.17	-2.7499	9.39E-05	0.000176575	ytzH
AB5991_15240	6.57	13.31	11.63	35.72	21.21	12.5	10.50	23.14	1.1397	0.006838054	0.010472007	ytmP
AB5991_15245	68.29	72.01	68.73	36.18	39.66	45.99	69.68	40.61	-0.7788	0.002178715	0.003561828	amyX
AB5991_15250	47.23	46.04	39.91	63.84	90.79	78.9	44.39	77.84	0.8102	0.0034076	0.005428697	ytlR
AB5991_15255	1.58	4.35	1.9	10.53	15.28	9.41	2.61	11.74	2.1693	5.21E-07	1.22E-06	ytlQ
AB5991_15260	2.29	7.22	5.25	15.31	16.66	13.82	4.92	15.26	1.6333	5.21E-05	0.000100372	ytlP
AB5991_15265	19.7	26.24	16.31	19.74	18.47	7.32	20.75	15.18	-0.4513	0.244958756	0.295327667	ytkP
AB5991_15270	23.5	34.1	25.41	220.02	233.25	248.79	27.67	234.02	3.0802	8.91E-27	9.86E-26	ytjP
AB5991_15275	0.83	0	0.3	2.72	1.98	0.6	0.38	1.77	2.2297	0.006485246	0.009962442	pbuO
AB5991_15280	3.28	7.17	4.84	5.43	3.65	4.22	5.10	4.43	-0.2012	0.705754908	0.757651457	ythQ

AB5991_15285	6.61	7.94	5.98	9.12	6.47	6.05	6.84	7.21	0.0760	0.866552272	0.906979197	ythP
AB5991_15290	7.33	26.91	15.67	100.95	76.24	44.95	16.64	74.05	2.1541	2.21E-06	4.87E-06	ytzE
AB5991_15295	1	0	1.07	6.55	7.41	4.62	0.69	6.19	3.1660	1.05E-07	2.61E-07	ytzG
AB5991_15300	1.99	3.25	1.89	9.5	7.88	4.67	2.38	7.35	1.6288	3.49E-05	6.89E-05	murJ
AB5991_15305	15.74	18.66	14.39	40	34.23	25.87	16.26	33.37	1.0368	0.000270262	0.000484957	ytfP
AB5991_15310	7.75	12.29	8.92	52.12	48.53	59.5	9.65	53.38	2.4673	4.97E-16	2.42E-15	opuD
AB5991_15315	8.89	3.63	2.11	0	0	0	4.88	0.00	-12.2517	0.001145441	0.001942993	cse60
AB5991_15330	2.51	2.63	0	6.63	12.41	6.99	1.71	8.68	2.3403	0.000149164	0.000274481	yteS
AB5991_15335	2.26	1.48	2.76	8.41	12.95	12.9	2.17	11.42	2.3980	8.01E-10	2.36E-09	yteR
AB5991_15340	0.19	0	0.4	0.61	1.14	1.42	0.20	1.06	2.4257	0.017738408	0.025792463	yteP
AB5991_15350	10.14	18.62	10.07	27.84	29.86	30.98	12.94	29.56	1.1914	9.45E-05	0.000177601	ytcQ
AB5991_15355	3.57	1.54	3.37	5.02	6.41	8.18	2.83	6.54	1.2095	0.005955351	0.009180395	ytcP
AB5991_15360	327.58	366.73	301.61	479.33	661.72	570.04	331.97	570.36	0.7808	0.002919518	0.004710851	ytbQ
AB5991_15365	113.04	124.6	112.1	170.6	253.33	205.71	116.58	209.88	0.8482	0.001492993	0.002484777	bioI
AB5991_15370	40.35	67.83	36.43	376.98	511.91	350.95	48.20	413.28	3.0999	1.30E-20	9.25E-20	bioB
AB5991_15375	20.52	20.03	22.5	264.08	345.72	241.48	21.02	283.76	3.7551	3.17E-35	6.46E-34	bioD
AB5991_15380	108.35	122.82	93.18	383.3	480.5	353.98	108.12	405.93	1.9086	5.15E-12	1.84E-11	bioF
AB5991_15385	132.44	256.25	149.96	327.49	396.6	251.15	179.55	325.08	0.8564	0.006610442	0.010146913	bioK
AB5991_15395	3.21	2.58	2.79	59.63	67.45	69.35	2.86	65.48	4.5169	8.04E-40	2.23E-38	ytaP
AB5991_15400	32.48	70.87	33.62	1.71	4.44	6.62	45.66	4.26	-3.4230	1.40E-13	5.61E-13	melR
AB5991_15405	53.05	70.99	62.17	46.19	56.29	74.23	62.07	58.90	-0.0755	0.794035558	0.839711105	melE
AB5991_15410	34.73	43.02	23.49	20.02	36.2	44.16	33.75	33.46	-0.0123	0.983706205	1	melD
AB5991_15415	35.67	63.9	37.68	11.83	16.82	27.55	45.75	18.73	-1.2882	0.000404721	0.000717156	melC
AB5991_15420	96.84	162.5	100.29	46.31	83.84	86.91	119.88	72.35	-0.7284	0.024158006	0.03437171	melA
AB5991_15425	5.21	20.21	4.96	10.08	7.08	2.51	10.13	6.56	-0.6271	0.388502052	0.44575026	ytwF
AB5991_15430	22.98	31.75	21.61	121.69	145.49	102.33	25.45	123.17	2.2751	4.34E-15	1.97E-14	leuS

AB5991_15435	798.31	820.84	904.34	503.15	405.7	248.07	841.16	385.64	-1.1251	0.000179307	0.00032722	ytvB
AB5991_15440	57.03	119.5	58.53	24.01	20.13	15.18	78.35	19.77	-1.9864	2.98E-08	7.74E-08	pfyP
AB5991_15445	1.21	3.61	2.1	0.49	1.54	0.49	2.31	0.84	-1.4573	0.026968105	0.038002251	yttB
AB5991_15455	4.84	7.52	6.97	3.65	4.74	1.31	6.44	3.23	-0.9948	0.019223312	0.027788778	bceB
AB5991_15460	0.95	0.44	1.27	1.55	2.41	1.54	0.89	1.83	1.0480	0.152611778	0.1914916	bceA
AB5991_15465	19.24	17.5	19.62	16.63	19.4	17.91	18.79	17.98	-0.0633	0.825948401	0.86883208	bceS
AB5991_15470	9.61	11.92	10.28	13.56	13.22	9.84	10.60	12.21	0.2032	0.549872448	0.607214541	bceR
AB5991_15475	7.45	6.84	6.34	53.38	51.64	30.3	6.88	45.11	2.7136	1.12E-16	5.78E-16	ytrF
AB5991_15480	0.52	0	0.56	22.31	20.09	10.96	0.36	17.79	5.6267	4.43E-19	2.74E-18	ytrE
AB5991_15485	3.88	4.41	4.94	15.08	11.1	6.8	4.41	10.99	1.3178	0.000792833	0.001362328	ytrD
AB5991_15490	1.83	3.03	2.55	18.52	12.49	4.76	2.47	11.92	2.2712	1.10E-05	2.25E-05	ytrC
AB5991_15495	2.47	4.15	1.76	10.73	7.95	2.23	2.79	6.97	1.3192	0.02035924	0.02933474	ytrB
AB5991_15500	0	0	0	0.5	3.74	1.49	0.00	1.91	10.8994	0.003733765	0.005907083	ytrA
AB5991_15505	0	0	0	0	0	0	0.00	0.00	0.0000	1	1	--
AB5991_15510	300.59	350.13	374.55	165.62	161.72	215.01	341.76	180.78	-0.9187	0.000628066	0.001091488	ytzC
AB5991_15515	187.65	221.95	206.46	16.23	17.47	15.55	205.35	16.42	-3.6449	3.17E-37	7.40E-36	ytqA
AB5991_15520	202.07	226.37	190.98	23.19	20.13	19.06	206.47	20.79	-3.3118	6.21E-30	8.57E-29	ytqB
AB5991_15525	4.75	8.12	9.28	47.54	39.49	28.53	7.38	38.52	2.3833	4.45E-12	1.60E-11	ytpB
AB5991_15530	3.94	8.51	7.19	42.84	34.67	32.11	6.55	36.54	2.4806	1.08E-12	4.12E-12	ytpA
AB5991_15535	53.59	57.89	52.82	40.38	39.22	42.47	54.77	40.69	-0.4286	0.103872222	0.135326966	ytoA
AB5991_15540	19.35	16.45	15.97	49.85	62.77	41.61	17.26	51.41	1.5749	7.39E-08	1.85E-07	ytnA
AB5991_15545	49.78	52.96	52.52	98.14	106.07	71.51	51.75	91.91	0.8285	0.001877738	0.003086299	asnB
AB5991_15550	1724.4	2004.31	1707.99	412.61	433.98	417.83	1812.23	421.47	-2.1043	9.09E-17	4.71E-16	metK
AB5991_15555	4913.32	4832.94	5259.81	2862.34	2551.62	2117.69	5002.02	2510.55	-0.9945	4.87E-05	9.44E-05	pckA
AB5991_15560	0.74	4.1	2.39	8.09	12.87	7.25	2.41	9.40	1.9641	0.001706406	0.002817522	ytmB
AB5991_15565	1.63	1.29	2.75	2.03	4.04	2.53	1.89	2.87	0.6010	0.343145744	0.399715972	ytmA

AB5991_15570	315.64	241.07	291.38	6.85	5.13	6.81	282.70	6.26	-5.4962	1.86E-60	2.11E-58	ytlA
AB5991_15575	109.88	83.08	110.1	2.76	2.58	4	101.02	3.11	-5.0200	2.58E-43	8.77E-42	ytlC
AB5991_15580	157.63	113.49	158.82	7.25	11.09	12.51	143.31	10.28	-3.8008	5.97E-30	8.26E-29	ytlD
AB5991_15585	2638.1	3188.79	2571.58	46.57	43.97	66.86	2799.49	52.47	-5.7376	1.53E-65	2.89E-63	ytkD
AB5991_15590	14388.53	14243.42	15689.25	88.34	123.55	185.03	14773.73	132.31	-6.8030	7.42E-75	3.69E-72	ytkC
AB5991_15595	67.27	151.55	71.93	2352.69	2157.53	2373.4	96.92	2294.54	4.5653	6.13E-35	1.20E-33	dps
AB5991_15600	11.54	2.35	5.48	19.52	13.05	20.82	6.46	17.80	1.4627	0.018336562	0.026623213	ytzI
AB5991_15605	7.02	15.15	13.24	58.79	66.78	33.06	11.80	52.88	2.1634	3.88E-08	1.00E-07	ytkA
AB5991_15610	54.53	183.45	57.09	138.93	74.13	40.04	98.36	84.37	-0.2213	0.662308722	0.717410784	luxS
AB5991_15615	0	2.91	0	0.86	0.81	0.86	0.97	0.84	-0.2019	1	1	ytjA
AB5991_15620	46.15	111.81	58.95	16.73	12.39	13.18	72.30	14.10	-2.3584	4.92E-10	1.48E-09	ytiB
AB5991_15625	82.75	274.58	111.78	73.42	57.63	107.65	156.37	79.57	-0.9747	0.020732142	0.029785577	rpmE2
AB5991_15630	12.48	22.18	14.51	2.36	2.62	0.88	16.39	1.95	-3.0688	1.39E-13	5.57E-13	ythA
AB5991_15635	10.24	16.58	15.97	1.89	1.24	2.82	14.26	1.98	-2.8463	2.71E-12	9.90E-12	ythB
AB5991_15640	68.21	114.8	106.98	25.96	4.63	9.84	96.66	13.48	-2.8425	3.05E-09	8.60E-09	ytzL
AB5991_15645	384.91	752.36	302.35	3837.98	4272.28	4563.89	479.87	4224.72	3.1381	6.48E-19	3.96E-18	mntD
AB5991_15650	286.05	490.98	225.51	3359.61	4086.9	4265.21	334.18	3903.91	3.5462	6.45E-26	6.69E-25	mntC
AB5991_15655	291.17	552.71	246.94	3696.92	4445.55	4520.48	363.61	4220.98	3.5371	1.04E-24	9.85E-24	mntB
AB5991_15660	164.85	272.43	137.26	2717.13	3319.25	3306.49	191.51	3114.29	4.0234	3.98E-34	7.32E-33	mntA
AB5991_15665	19.76	21.71	24.42	96	108.3	102.39	21.96	102.23	2.2186	3.33E-16	1.64E-15	menC
AB5991_15670	15.34	19.08	12.97	139.4	163.06	166.73	15.80	156.40	3.3075	1.21E-29	1.63E-28	menE
AB5991_15675	19.27	21.56	19.42	198.03	257.9	235.7	20.08	230.54	3.5210	1.22E-33	2.11E-32	menB
AB5991_15680	6.35	6.44	8.43	34.55	35.68	23.48	7.07	31.24	2.1428	1.25E-10	3.95E-10	menH
AB5991_15685	16.83	15.83	24.88	22.93	28.02	33.96	19.18	28.30	0.5614	0.061729123	0.082701197	menD
AB5991_15690	3.96	5.16	7.1	13.88	11.69	7.6	5.41	11.06	1.0321	0.003727721	0.005899873	menF
AB5991_15695	31.63	40.56	47.25	3	2.81	5.71	39.81	3.84	-3.3741	3.87E-19	2.41E-18	yteA

AB5991_15700	31.78	39.31	28.56	4.08	8.31	11.23	33.22	7.87	-2.0769	1.23E-08	3.30E-08	ytdA
AB5991_15705	129.49	111.4	129.91	9.32	19.58	36.49	123.60	21.80	-2.5035	4.99E-10	1.49E-09	ytcA
AB5991_15710	145.21	103.65	159.15	12.2	25.92	55.55	136.00	31.22	-2.1229	1.23E-06	2.77E-06	ytcB
AB5991_15715	225.75	158.12	236.46	18.21	37.58	60.2	206.78	38.66	-2.4190	4.72E-10	1.42E-09	ytcC
AB5991_15720	111.23	88.97	77.05	21.54	53.6	67.5	92.42	47.55	-0.9588	0.009412591	0.014136686	ytxO
AB5991_15725	69.32	48.09	62.23	15.45	49.47	64.29	59.88	43.07	-0.4754	0.253864769	0.305206478	cotS
AB5991_15730	42.4	35.12	39.2	5.55	13.79	20.53	38.91	13.29	-1.5497	4.81E-05	9.32E-05	cotSA
AB5991_15735	193.37	385.97	193.29	15.56	25.69	45.36	257.54	28.87	-3.1572	5.80E-14	2.41E-13	cotI
AB5991_15740	32.06	84.39	33.45	5.46	3.93	18.4	49.97	9.26	-2.4314	1.37E-05	2.80E-05	ytaB
AB5991_15745	84.76	131.81	84.11	5.9	4.37	3.75	100.23	4.67	-4.4227	4.05E-38	9.74E-37	glgP
AB5991_15750	43.85	62.73	41.32	7.57	9.1	9.14	49.30	8.60	-2.5186	2.00E-16	1.01E-15	glgA
AB5991_15755	62.35	112.56	56.75	9.71	10.87	11.19	77.22	10.59	-2.8663	1.78E-16	8.97E-16	glgD
AB5991_15760	38.9	64.46	46.67	9.29	5.31	4.79	50.01	6.46	-2.9519	2.80E-17	1.50E-16	glgC
AB5991_15765	25.9	41.22	28.21	3.86	2.83	4.05	31.78	3.58	-3.1499	1.06E-21	8.03E-21	glgB
AB5991_15890	0.94	0.57	2	0.68	0.32	0.34	1.17	0.45	-1.3892	0.226424884	0.274544493	thiT
AB5991_15895	72.02	97.91	66.65	21.09	7.05	14.24	78.86	14.13	-2.4809	3.10E-11	1.04E-10	yuaI
AB5991_15900	104.67	118.01	90.46	43.04	21.88	31.84	104.38	32.25	-1.6943	2.20E-08	5.78E-08	floT
AB5991_15905	34.43	59.42	37.19	11.61	4.2	5.96	43.68	7.26	-2.5896	3.12E-10	9.55E-10	nfeD2
AB5991_15910	19.59	28.51	20.16	59.09	68.09	52.42	22.75	59.87	1.3957	2.52E-06	5.52E-06	yuaE
AB5991_15915	7.8	9.74	7.01	82.84	91.5	85.5	8.18	86.61	3.4038	2.98E-28	3.60E-27	yuaD
AB5991_15920	4.49	4.67	2.4	15.12	13.39	13.27	3.85	13.93	1.8537	4.93E-08	1.25E-07	gbsB
AB5991_15925	2.45	2.48	2.1	2.4	3.5	2.79	2.34	2.90	0.3058	0.448214316	0.506937924	gbsA
AB5991_15930	3.66	2.44	3.56	0.72	0.68	0	3.22	0.47	-2.7866	0.000202162	0.000367745	gbsR
AB5991_15935	412.48	352.56	305.87	6868.09	6691.93	6639.58	356.97	6733.20	4.2374	7.73E-51	4.72E-49	bslA
AB5991_15940	4.86	8.43	4.33	9.7	4.67	6.73	5.87	7.03	0.2600	0.545671601	0.602910943	ktrA
AB5991_15945	1.62	2.98	3.32	5	4.81	2.92	2.64	4.24	0.6847	0.105138194	0.136796778	ktrB

AB5991_15950	18.49	13.83	10.25	2.23	6.27	6.67	14.19	5.06	-1.4886	0.003370384	0.005372861	yubF
AB5991_15955	36.83	48.47	33.24	2.08	0.65	0.69	39.51	1.14	-5.1152	1.34E-31	2.01E-30	lytG
AB5991_15960	0.94	2.38	1.64	0.9	1.08	1.4	1.65	1.13	-0.5533	0.378564308	0.435985751	yubD
AB5991_15965	34.22	32.1	44.15	6.88	9.84	19.73	36.82	12.15	-1.5997	2.95E-05	5.87E-05	cdoA
AB5991_15975	9.57	9.19	6.75	44	31.66	25.9	8.50	33.85	1.9932	2.63E-09	7.45E-09	uppP
AB5991_15980	1.08	6.83	3.64	16.17	11.35	13.25	3.85	13.59	1.8196	9.00E-05	0.000169712	yubA
AB5991_15985	6.41	14.12	8.62	20.91	20.69	8.52	9.72	16.71	0.7819	0.057788992	0.077605711	iolU
AB5991_15990	12.76	21.35	10.76	57.97	69.4	67.37	14.96	64.91	2.1177	3.88E-11	1.29E-10	rhaA
AB5991_15995	3.44	6.32	4.91	13.73	24.53	14.91	4.89	17.72	1.8577	9.44E-05	0.000177463	rhaM
AB5991_16000	11.28	32.1	13.52	27.24	38.86	24.69	18.97	30.26	0.6741	0.075514357	0.100189613	rhaB
AB5991_16005	20.7	38.44	19.9	22.77	33.38	28.46	26.35	28.20	0.0982	0.761716565	0.810280332	yulB
AB5991_16010	3.58	3.53	3.46	12.63	19.37	17.11	3.52	16.37	2.2160	1.05E-12	3.99E-12	yuxG
AB5991_16020	36.77	36.43	34.06	2.47	1.95	2.56	35.75	2.33	-3.9417	9.80E-37	2.16E-35	mcpA
AB5991_16030	45.16	65.58	46.94	0.99	2.13	2.85	52.56	1.99	-4.7231	1.35E-33	2.34E-32	mcpB
AB5991_16035	35.02	29.23	38.24	7.99	24.18	33.14	34.16	21.77	-0.6501	0.114890801	0.148464487	tgl
AB5991_16040	89.31	123.65	87.17	148.78	207.77	156.53	100.04	171.03	0.7736	0.006657865	0.010215758	yuzH
AB5991_16045	17.21	28.28	9.2	217.27	279.86	269.71	18.23	255.61	3.8096	1.06E-23	9.19E-23	yugU
AB5991_16050	0	2.46	1.43	1.46	1.36	0	1.30	0.94	-0.4641	1	1	--
AB5991_16055	11.94	19.53	13.47	1.53	1.99	1.06	14.98	1.53	-3.2946	1.86E-17	1.01E-16	yugT
AB5991_16060	4.76	2.57	2.4	2.13	2.57	1.52	3.24	2.07	-0.6455	0.142169622	0.179737526	yugS
AB5991_16065	17.6	26.92	19.96	30.44	23.06	25.4	21.49	26.30	0.2912	0.330997451	0.38681309	yugP
AB5991_16070	0.68	6.29	2.93	0	2.09	0	3.30	0.70	-2.2439	0.093848039	0.122953007	yuzI
AB5991_16075	2.71	4.98	4.64	1.18	1.66	0.59	4.11	1.14	-1.8459	0.016177865	0.023618494	mstX
AB5991_16080	4.03	9.42	7.83	4.18	3.17	4.76	7.09	4.04	-0.8133	0.057864995	0.077681506	yugO
AB5991_16085	375.33	287.64	376.09	262.11	330.67	340.11	346.35	310.96	-0.1555	0.554811301	0.611647884	yugN
AB5991_16090	389.68	269.44	422.44	229.34	276.93	242.47	360.52	249.58	-0.5306	0.050995122	0.068901541	yugM

AB5991_16095	69.47	81.44	77.86	186.7	174.17	128.13	76.26	163.00	1.0959	4.38E-05	8.54E-05	pgi
AB5991_16100	3.39	6.51	5.93	14.92	21.33	12.68	5.28	16.31	1.6281	5.54E-06	1.18E-05	yugK
AB5991_16105	11.96	11.98	14.61	110.45	153.3	118.67	12.85	127.47	3.3104	1.80E-28	2.21E-27	yugJ
AB5991_16110	88.47	96.63	149.25	29.03	29.5	26.42	111.45	28.32	-1.9767	2.52E-09	7.16E-09	yuzA
AB5991_16115	126.48	171.43	139.19	127.05	143.24	96.59	145.70	122.29	-0.2527	0.382579258	0.439716999	yugI
AB5991_16120	77.06	76.04	60.6	91.09	76.69	67.41	71.23	78.40	0.1382	0.604926236	0.661570389	yugH
AB5991_16125	50.51	60.95	49.77	24.33	17.62	14.06	53.74	18.67	-1.5254	9.78E-07	2.23E-06	yugG
AB5991_16130	8.8	6.46	9.17	1.91	2.46	1.19	8.14	1.85	-2.1355	3.55E-07	8.44E-07	yugF
AB5991_16135	21.47	5.09	22.22	0.75	2.82	0.75	16.26	1.44	-3.4972	1.66E-06	3.70E-06	yugE
AB5991_16140	5.75	5.42	3.65	2.7	2.69	1.51	4.94	2.30	-1.1029	0.007379538	0.011244875	patB
AB5991_16145	5.06	5.42	5.26	7.94	4.57	5.02	5.25	5.84	0.1554	0.683169083	0.737391799	kinB
AB5991_16150	10.74	12.86	9.99	11.68	9.03	6.57	11.20	9.09	-0.3002	0.50678177	0.566552608	kapB
AB5991_16155	2468.48	1849.56	2368.01	34.04	58.64	62.37	2228.68	51.68	-5.4303	2.88E-53	2.04E-51	kapD
AB5991_16160	246.32	204.99	313.63	5.78	5.41	1.92	254.98	4.37	-5.8666	3.10E-43	1.04E-41	--
AB5991_16165	23.76	28.43	29.35	39.02	46.96	32.86	27.18	39.61	0.5434	0.048991161	0.066351944	yuxJ
AB5991_16170	87.15	93.99	95.77	53.16	59.85	40.18	92.30	51.06	-0.8541	0.001308028	0.002201856	pbpD
AB5991_16175	32.74	30.46	39.22	33.71	35.1	19.38	34.14	29.40	-0.2158	0.511348386	0.569081258	yuxK
AB5991_16180	18.46	25.58	20.78	4.23	7.58	2.45	21.61	4.75	-2.1845	1.10E-07	2.71E-07	yufK
AB5991_16185	34.75	48.48	36.8	8.1	9.3	5.62	40.01	7.67	-2.3824	6.81E-15	3.05E-14	malK
AB5991_16190	43.91	54.85	37.95	10.55	17.15	8.84	45.57	12.18	-1.9036	9.79E-09	2.65E-08	malR
AB5991_16195	1688.12	1925.26	1604.42	31.63	28.62	48	1739.27	36.08	-5.5910	4.51E-62	6.63E-60	nupN
AB5991_16200	7.9	11.91	9.2	8.21	7.8	10.59	9.67	8.87	-0.1251	0.711571425	0.762864506	nupO
AB5991_16205	19.68	12.36	16.25	6.2	4.92	6.73	16.10	5.95	-1.4358	9.31E-06	1.93E-05	nupP
AB5991_16210	40.1	45.98	43.89	5.73	8.24	9.99	43.32	7.99	-2.4395	1.31E-15	6.16E-15	nupQ
AB5991_16215	2.82	4.19	3.01	5.25	0	0.29	3.34	1.85	-0.8549	0.399332101	0.456593082	maeN
AB5991_16220	2.51	7.68	4.47	0	0	0.91	4.89	0.30	-4.0099	0.003121886	0.005002829	yufS

AB5991_16225	3.16	2.9	4.34	10.05	9.1	7.64	3.47	8.93	1.3651	1.34E-05	2.73E-05	mrpA
AB5991_16230	2.51	3.07	4.47	7.74	5.54	4.53	3.35	5.94	0.8255	0.115492652	0.149096659	mrpB
AB5991_16235	3.7	0	1.13	2.87	3.23	4	1.61	3.37	1.0643	0.187719104	0.231788732	mrpC
AB5991_16240	6.71	3.36	5.22	10.35	9.93	8.85	5.10	9.71	0.9299	0.004964795	0.00772842	mrpD
AB5991_16245	4.93	6.96	7.29	8.24	14.27	15.18	6.39	12.56	0.9746	0.016772201	0.024459203	mrpE
AB5991_16250	4.44	5.82	4.07	11.73	9.68	15.1	4.78	12.17	1.3493	0.005574315	0.008619784	mrpF
AB5991_16255	8.68	0	5.15	6.29	12.26	7.83	4.61	8.79	0.9316	0.158060731	0.197563476	mrpG
AB5991_16260	7.59	10.45	8.62	47.98	40.08	33.89	8.89	40.65	2.1935	3.09E-10	9.47E-10	yuxO
AB5991_16265	2.8	6.69	5.09	17.98	18.54	11.83	4.86	16.12	1.7295	1.29E-05	2.64E-05	comA
AB5991_16270	13.52	17.65	17.72	13.51	15.99	11.25	16.30	13.58	-0.2627	0.352098158	0.408825084	comP
AB5991_16275	2.44	2.99	13.93	5.31	5.8	1.76	6.45	4.29	-0.5891	0.51040838	0.568536234	comX
AB5991_16280	17.36	16.9	15.66	9.33	12.77	9.06	16.64	10.39	-0.6799	0.025047986	0.035498055	comQ
AB5991_16285	0	0	1.37	27.88	22.18	12.49	0.46	20.85	5.5128	1.95E-08	5.16E-08	degQ
AB5991_16290	4.3	5.27	6.14	0	0	0	5.24	0.00	-12.3544	0.006098366	0.009389923	--
AB5991_16295	46.53	64.76	46.1	0	1	0	52.46	0.33	-7.2982	3.79E-33	6.36E-32	yuzC
AB5991_16300	1.62	1.89	1.26	15.5	12.11	9.7	1.59	12.44	2.9675	5.41E-14	2.26E-13	yuxH
AB5991_16305	20.25	20.95	19.68	27.23	34.34	28.16	20.29	29.91	0.5596	0.031941565	0.044552144	pncB
AB5991_16310	4.58	7.82	5.6	19.94	22.66	20.91	6.00	21.17	1.8190	1.66E-07	4.05E-07	pncA
AB5991_16315	10.87	7.49	10.66	6.9	9.68	4.9	9.67	7.16	-0.4341	0.3142411	0.369077613	yueI
AB5991_16320	4.36	9.33	12.42	10.26	5.17	3.14	8.70	6.19	-0.4916	0.450301656	0.508574482	yueH
AB5991_16325	310.2	304.98	316.06	19.48	24.03	14.98	310.41	19.50	-3.9929	6.78E-36	1.44E-34	yueG
AB5991_16330	231.07	229.03	233.17	19.48	22.21	18.33	231.09	20.01	-3.5299	6.53E-36	1.39E-34	yueF
AB5991_16335	45.42	38.3	46.09	2.02	4.72	4.01	43.27	3.58	-3.5940	3.59E-20	2.46E-19	yuzE
AB5991_16345	7152.36	7111.58	7204.29	193.62	203.01	215.56	7156.08	204.06	-5.1321	2.89E-72	1.15E-69	yueE
AB5991_16350	52.35	52.14	53.87	35.45	31.92	32.88	52.79	33.42	-0.6596	0.009929303	0.014845855	yueD
AB5991_16355	35.67	24.75	28.4	7.33	12.1	13.73	29.61	11.05	-1.4214	4.20E-05	8.21E-05	yueC

AB5991_16360	13.98	15.24	11.91	8.9	10.99	8.74	13.71	9.54	-0.5227	0.050640053	0.068468387	yueB
AB5991_16365	8.54	7.84	7.15	12.05	13.24	12.34	7.84	12.54	0.6774	0.01033886	0.015417054	yukB
AB5991_16370	0.53	1.22	0	2.32	2.17	1.3	0.58	1.93	1.7262	0.005884843	0.009078754	yukC
AB5991_16375	0	0	0.81	1.64	0.77	0.82	0.27	1.08	1.9955	0.395242457	0.452307723	yukD
AB5991_16380	1152.74	977.61	748.19	2750.79	2433.39	2212.84	959.51	2465.67	1.3616	6.33E-07	1.47E-06	yukE
AB5991_16385	83.04	109.32	95.05	11.46	8.99	11.41	95.80	10.62	-3.1733	7.79E-27	8.71E-26	adeR
AB5991_16390	300.61	404.28	254.32	324.18	236.7	186.71	319.74	249.20	-0.3596	0.230239191	0.27874385	ald
AB5991_16395	515.32	543.85	413.39	792.41	730.14	676.45	490.85	733.00	0.5785	0.025063981	0.035508052	yukJ
AB5991_16400	21.52	9.48	13.81	58.04	55.19	54.97	14.94	56.07	1.9083	2.55E-07	6.15E-07	ybdZ
AB5991_16405	38.29	29.99	25.73	83.68	125.63	146.56	31.34	118.62	1.9205	1.87E-10	5.84E-10	dhbF
AB5991_16410	4.62	5.3	4.94	55.9	83.07	91.06	4.95	76.68	3.9523	1.04E-29	1.40E-28	dhbB
AB5991_16415	2.68	4.92	1.91	22.33	38.95	43.48	3.17	34.92	3.4615	2.16E-18	1.28E-17	dhbE
AB5991_16420	4.53	1.94	2.91	34.49	45.8	47.89	3.13	42.73	3.7724	1.37E-26	1.50E-25	dhbC
AB5991_16425	0.46	0.84	1.23	22.26	26.45	31.86	0.84	26.86	4.9930	4.56E-27	5.19E-26	dhbA
AB5991_16430	9.14	13.73	9.11	55.81	83.31	73.09	10.66	70.74	2.7303	6.25E-18	3.54E-17	besA
AB5991_16435	8.78	12.23	7.77	16.46	14.79	12.13	9.59	14.46	0.5920	0.078323569	0.103570727	yuiH
AB5991_16440	31.47	36.33	31.09	107.26	81.15	70.09	32.96	86.17	1.3863	1.05E-06	2.38E-06	bioYB
AB5991_16445	58.89	47.2	65.16	4.73	4.43	2.94	57.08	4.03	-3.8230	1.19E-30	1.71E-29	yuiF
AB5991_16450	13.95	16.12	12.86	100.45	123.13	124.06	14.31	115.88	3.0175	2.71E-26	2.91E-25	pepA
AB5991_16455	13.26	21.57	14.99	69.65	47.44	50.45	16.61	55.85	1.7497	8.36E-08	2.09E-07	yuiD
AB5991_16460	18.98	19.2	19.12	8.98	8.4	4.47	19.10	7.28	-1.3909	4.50E-05	8.77E-05	yuiC
AB5991_16465	1157.7	1715.28	1447.6	863.49	802.31	710.72	1440.19	792.17	-0.8624	0.00127669	0.002153668	yuiB
AB5991_16470	136.82	133.68	178.53	31.4	11.5	16.31	149.68	19.74	-2.9229	1.85E-14	8.00E-14	yuiA
AB5991_16475	41.15	56.27	45.59	43.31	33.45	26.28	47.67	34.35	-0.4729	0.110058683	0.14277786	yumB
AB5991_16480	6.15	7.31	6.58	81.27	53.95	34.08	6.68	56.43	3.0786	1.36E-16	6.94E-16	yumC
AB5991_16485	0	0	0	0	0	0	0.00	0.00	0.0000	1	1	yuzG

AB5991_16490	9.4	15.9	8.28	28.05	6.38	13.96	11.19	16.13	0.5271	0.268833553	0.320485751	guaC
AB5991_16495	28.1	23.93	23.54	25.2	36.26	36.37	25.19	32.61	0.3725	0.212213492	0.259292239	paiB
AB5991_16500	0.7	1.92	1.49	4.55	4.96	2.64	1.37	4.05	1.5637	0.008762827	0.013205763	paiA
AB5991_16510	3.19	5.86	0.85	12.15	11.78	5.18	3.30	9.70	1.5560	0.005813785	0.008972615	--
AB5991_16515	4.12	4.26	3.48	1.26	1.42	1.26	3.95	1.31	-1.5898	1.30E-06	2.93E-06	TMP
AB5991_16520	0.54	4.98	0.58	0	1.1	0	2.03	0.37	-2.4713	0.139709046	0.177021257	--
AB5991_16525	9.22	11.65	8.32	0	0	0.62	9.73	0.21	-5.5571	7.03E-16	3.36E-15	--
AB5991_16530	0	0	0	0	0	0	0.00	0.00	0.0000	1	1	--
AB5991_16535	0	0	0	0	0	0	0.00	0.00	0.0000	1	1	--
AB5991_16540	0	0	0	0	0	0	0.00	0.00	0.0000	1	1	--
AB5991_16545	0	0	0	0	0	0	0.00	0.00	0.0000	1	1	--
AB5991_16550	0	0	0	0	0	0	0.00	0.00	0.0000	1	1	--
AB5991_16555	5.81	3.2	7.87	0.84	0.79	1.26	5.63	0.96	-2.5462	1.08E-06	2.46E-06	--
AB5991_16560	0	0	0.83	0	0	0	0.28	0.00	-8.1120	1	1	--
AB5991_16565	3.89	0	8.31	0	0	0	4.07	0.00	-11.9896	0.004748514	0.007409174	--
AB5991_16570	1.66	3.04	6.5	0	1.13	0.6	3.73	0.58	-2.6947	0.009383898	0.014104262	--
AB5991_16575	5.99	9.17	7.97	0	0.68	0	7.71	0.23	-5.0881	6.29E-12	2.23E-11	--
AB5991_16580	0	2.41	1.4	0	0	0	1.27	0.00	-10.3106	0.509770347	0.568309168	--
AB5991_16585	12.45	15.27	11.07	33.58	32.65	30.4	12.93	32.21	1.3168	1.47E-06	3.31E-06	--
AB5991_16590	2.51	15.37	14.32	10.92	7.66	11.78	10.73	10.12	-0.0849	0.937638772	0.973937631	--
AB5991_16595	6.46	2.96	6.33	7.61	11.5	6.29	5.25	8.47	0.6895	0.197229552	0.242626564	--
AB5991_16600	0	0	0	0	0	0	0.00	0.00	0.0000	1	1	--
AB5991_16605	2.29	0	0	2.49	2.33	0.83	0.76	1.88	1.3029	0.546318613	0.603457917	--
AB5991_16610	30.45	32.47	30.11	0	0.33	2.13	31.01	0.82	-5.2410	1.32E-26	1.45E-25	yoeC
AB5991_16615	0.5	0	0	0	0.51	0	0.17	0.17	0.0286	1	1	--
AB5991_16620	3.97	4.12	2.77	1.31	0.7	0.93	3.62	0.98	-1.8851	0.000110519	0.000206139	CA_C2066

AB5991_16625	974.45	956.36	1024.51	176	166.22	154.16	985.11	165.46	-2.5738	6.83E-22	5.26E-21	sufA
AB5991_16630	1407.73	1451	1417.94	30.81	30.12	38.22	1425.56	33.05	-5.4307	1.49E-68	4.22E-66	dapF
AB5991_16635	11.31	18.3	18.14	1.46	0.76	2.25	15.92	1.49	-3.4172	3.02E-16	1.49E-15	yutK
AB5991_16640	0.76	0	0	2.49	0.78	0.83	0.25	1.37	2.4316	0.243996497	0.29441206	yuzB
AB5991_16645	82.59	115.29	81.62	30.74	23.25	20.52	93.17	24.84	-1.9073	1.74E-10	5.46E-10	yutJ
AB5991_16650	0.55	1.01	1.77	12.62	10.69	7.78	1.11	10.36	3.2229	8.45E-07	1.94E-06	yuzD
AB5991_16655	159.23	167.92	131.74	580.38	533.26	388.99	152.96	500.88	1.7113	7.77E-10	2.29E-09	yutI
AB5991_16660	4.94	8.91	5.58	51.98	46.88	49.16	6.48	49.34	2.9294	4.04E-20	2.75E-19	yuxL
AB5991_16665	97.95	141.68	80.23	160.65	124.82	114.66	106.62	133.38	0.3230	0.265502161	0.316799603	thrB
AB5991_16670	128.18	165.16	119.55	158.53	134.97	118.8	137.63	137.43	-0.0021	0.998515307	1	thrC
AB5991_16675	33.32	69.08	38.45	87.57	57.08	52.3	46.95	65.65	0.4837	0.146922952	0.184981307	hom
AB5991_16680	16.48	22.45	15.16	4.43	6.31	5.56	18.03	5.43	-1.7305	1.47E-07	3.58E-07	yutH
AB5991_16685	56.64	66.25	61.73	2.75	2.57	5.08	61.54	3.47	-4.1499	2.69E-28	3.25E-27	yutG
AB5991_16690	5.63	10.33	4.51	31.87	41.52	27.66	6.82	33.68	2.3035	2.26E-10	7.00E-10	yutF
AB5991_16695	2.08	4.58	7.55	26.66	25.37	8.55	4.74	20.19	2.0919	0.000155048	0.000284517	yutE
AB5991_16700	3.27	2.41	4.9	11.4	17.33	11.34	3.53	13.36	1.9212	0.000259291	0.000466959	yutD
AB5991_16705	47.97	58.72	48.55	4.66	3.2	5.87	51.75	4.58	-3.4991	1.32E-23	1.13E-22	yutC
AB5991_16710	38.89	34.41	36.63	100.37	87.37	51.91	36.64	79.88	1.1243	0.000285133	0.000511181	lipA
AB5991_16715	14.74	18.61	17.14	5.41	3.94	4.59	16.83	4.65	-1.8568	9.47E-09	2.57E-08	lytH
AB5991_16720	373.97	476.23	361.42	14.59	34.01	40.51	403.87	29.70	-3.7652	1.14E-23	9.84E-23	yunB
AB5991_16725	1918.54	2044.49	2226.04	97.65	156.91	198.22	2063.02	150.93	-3.7728	1.10E-30	1.60E-29	yunC
AB5991_16730	20.17	18.16	14.89	28.31	25.56	21.41	17.74	25.09	0.5003	0.064657649	0.086478789	yunD
AB5991_16735	9.24	13.73	10.35	13.87	15.22	14.76	11.11	14.62	0.3962	0.19458719	0.239598677	yunE
AB5991_16740	2.13	4.3	3.87	13.66	16.03	13.83	3.43	14.51	2.0790	1.29E-08	3.45E-08	yunF
AB5991_16745	0	0	1.11	3.39	3.17	1.12	0.37	2.56	2.7905	0.017685441	0.025724867	yunG
AB5991_16750	45.02	38.61	40.65	7.33	15.09	20.72	41.43	14.38	-1.5265	9.75E-06	2.01E-05	allB

AB5991_16755	21.29	15.18	24.59	9.36	6.8	7.72	20.35	7.96	-1.3544	7.76E-06	1.63E-05	pucR
AB5991_16760	17.14	9.1	15.89	3.64	3.13	3.33	14.04	3.37	-2.0605	5.80E-09	1.60E-08	pucJ
AB5991_16765	13.42	5.9	14.2	2.74	2.56	2.57	11.17	2.62	-2.0906	2.01E-07	4.87E-07	pucK
AB5991_16770	24.95	19.89	23.56	7.15	5.7	6.32	22.80	6.39	-1.8351	1.43E-10	4.50E-10	pucL
AB5991_16775	18.34	8.66	12.33	2.28	4.8	5.67	13.11	4.25	-1.6251	0.000987422	0.001684301	pucM
AB5991_16780	4702.22	3502.45	5987.71	44.68	66.89	60.77	4730.79	57.45	-6.3637	6.22E-64	1.03E-61	yuzJ
AB5991_16785	9.35	4.45	8.52	7.91	12.69	6	7.44	8.87	0.2531	0.624111949	0.680117604	pucE
AB5991_16790	8.24	7.27	11.83	4.57	4.85	5.42	9.11	4.95	-0.8815	0.004511513	0.007061206	pucD
AB5991_16795	2.38	0.8	4.4	1.18	1.76	2.58	2.53	1.84	-0.4575	0.457859322	0.516669328	pucC
AB5991_16800	0.58	0.54	0.94	0.95	0.6	1.58	0.69	1.04	0.6035	0.641109465	0.696537807	pucB
AB5991_16805	4	2.34	5.84	1.39	1.3	1.58	4.06	1.42	-1.5122	0.002018342	0.003309181	pucA
AB5991_16810	1.44	1.06	1.7	4.71	6.47	7.19	1.40	6.12	2.1289	3.64E-07	8.65E-07	pucG
AB5991_16815	2.19	0.27	1.4	0.63	1.04	2.68	1.29	1.45	0.1724	0.932515481	0.970138584	pucF
AB5991_16820	66.8	38.48	68.63	0	0	0	57.97	0.00	-15.8230	1.67E-19	1.08E-18	--
AB5991_16825	507.01	199.06	488.92	47.16	50.5	37.91	398.33	45.19	-3.1399	4.25E-17	2.25E-16	bsn
AB5991_16830	24.72	16.84	22.41	8.73	9.5	8.51	21.32	8.91	-1.2584	2.63E-05	5.26E-05	yurJ
AB5991_16835	59.01	20.03	68.94	7.01	5.55	4.56	49.33	5.71	-3.1116	5.10E-12	1.83E-11	yurK
AB5991_16840	13.74	5.43	9.72	4.6	3.66	4.35	9.63	4.20	-1.1960	0.003071688	0.004928352	frlD
AB5991_16850	9.46	4.53	6.16	0.45	0.63	1.78	6.72	0.95	-2.8167	3.56E-07	8.45E-07	yurN
AB5991_16855	26.35	10.72	25.13	2.32	1.45	1.7	20.73	1.82	-3.5073	2.41E-15	1.11E-14	yurO
AB5991_16860	40.47	26.23	34.86	3.19	6.52	5.75	33.85	5.15	-2.7157	6.46E-15	2.91E-14	frlB
AB5991_16865	70.37	73.46	67.52	11.53	10.3	7.3	70.45	9.71	-2.8591	4.23E-18	2.45E-17	yurQ
AB5991_16870	55.08	72.37	58.04	12.65	14.3	10.49	61.83	12.48	-2.3087	3.16E-15	1.45E-14	yurR
AB5991_16875	2140.71	2560.3	2398.42	89.6	117.63	99.83	2366.48	102.35	-4.5311	9.93E-50	5.48E-48	--
AB5991_16880	6519.41	7438.67	6108.36	207.26	295.27	380.69	6688.81	294.41	-4.5059	5.56E-44	1.94E-42	yurS
AB5991_16885	26.83	52.72	25.17	12.29	9.1	9.17	34.91	10.19	-1.7768	1.18E-05	2.41E-05	yurT

AB5991_16890	30.45	59.48	45.03	1.41	1.98	4.21	44.99	2.53	-4.1504	1.03E-15	4.85E-15	yuzN
AB5991_16895	283.54	306.01	260.49	1028.89	1289.42	1213.58	283.35	1177.30	2.0548	1.25E-15	5.86E-15	sufB
AB5991_16900	120.91	125.58	133.65	244.84	314.47	258.24	126.71	272.52	1.1048	2.17E-05	4.36E-05	sufU
AB5991_16905	230.04	262.58	228.12	523.89	613.35	574.81	240.25	570.68	1.2482	8.57E-07	1.97E-06	sufS
AB5991_16910	344.03	443.78	371.29	524.66	568.68	479.18	386.37	524.17	0.4401	0.086247735	0.113369664	sufD
AB5991_16915	268.13	358.49	280.1	189.08	210.64	155.58	302.24	185.10	-0.7074	0.008441668	0.012750803	sufC
AB5991_16920	14.41	21.65	8.4	7.12	9.33	2.84	14.82	6.43	-1.2047	0.086301368	0.113402625	yuzK
AB5991_16930	11.61	12.07	16.17	11.91	8.03	8.78	13.28	9.57	-0.4725	0.141493681	0.178996944	metQ
AB5991_16935	2.43	1.49	3.76	1.47	2.47	2.63	2.56	2.19	-0.2252	0.723369333	0.774050019	metP
AB5991_16940	7.4	3.56	5.09	3.64	1.97	2.67	5.35	2.76	-0.9549	0.021933997	0.031444008	metN
AB5991_16945	19.38	16.35	17.37	23.36	16.53	10.78	17.70	16.89	-0.0676	0.892816557	0.931033232	yusD
AB5991_16950	10.14	15.51	18.06	9.8	6.3	7.31	14.57	7.80	-0.9008	0.038915417	0.053564341	yusE
AB5991_16955	16.43	18.29	19.7	18.95	18.75	12.94	18.14	16.88	-0.1039	0.779364947	0.826451249	yusF
AB5991_16960	221.93	222.66	209.61	593.89	631.83	603.5	218.07	609.74	1.4834	5.78E-09	1.60E-08	yusG
AB5991_16965	187.34	226.45	139.43	817.55	860.87	810.67	184.41	829.70	2.1697	1.49E-14	6.52E-14	gcvH
AB5991_16970	1.01	1.86	7.58	6.06	4.12	3.84	3.48	4.67	0.4240	0.608303978	0.664532351	yusI
AB5991_16975	1108.09	1128.61	837.39	928.06	718.11	699.12	1024.70	781.76	-0.3904	0.130981411	0.16751278	fadE
AB5991_16980	528.26	576.57	425.38	665.97	564.23	577.17	510.07	602.46	0.2402	0.354158227	0.410976716	fadA
AB5991_16985	343.96	418.57	359.51	1161.9	1157.62	1071.35	374.01	1130.29	1.5955	1.37E-10	4.32E-10	fadN
AB5991_16990	24.59	15.8	26.3	2.67	0	0	22.23	0.89	-4.6426	7.39E-09	2.02E-08	yuzL
AB5991_16995	267.25	163.57	256.87	14.71	46.34	63.07	229.23	41.37	-2.4700	1.26E-08	3.37E-08	fadM
AB5991_17000	69.8	47.22	62.07	6.39	2.99	3.98	59.70	4.45	-3.7447	1.31E-18	7.82E-18	yuzM
AB5991_17005	84.13	74.75	96.36	11.22	8.29	8.23	85.08	9.25	-3.2018	6.28E-21	4.55E-20	yusN
AB5991_17010	18.92	21.98	30.15	10.92	4.72	2.51	23.68	6.05	-1.9689	2.06E-05	4.15E-05	yusO
AB5991_17015	54.25	53.68	69.42	30.47	18.44	14.2	59.12	21.04	-1.4907	3.45E-06	7.49E-06	yusP
AB5991_17020	0.94	4.32	0.5	0	0.96	1.02	1.92	0.66	-1.5406	0.273113615	0.324808076	yusQ

AB5991_17025	1.22	0.45	1.3	1.33	2.98	1.85	0.99	2.05	1.0525	0.14590707	0.183818837	yusR
AB5991_17030	63.3	69.52	67.91	6.64	5.39	8.15	66.91	6.73	-3.3143	1.47E-27	1.73E-26	yusT
AB5991_17040	34.93	57.72	37.12	86.18	63.99	65.22	43.26	71.80	0.7310	0.015445497	0.022607471	yusV
AB5991_17045	0.83	0.76	1.77	1.8	0.42	0.45	1.12	0.89	-0.3316	1	1	yusW
AB5991_17050	68.6	93.82	71.64	91.56	96.33	94.18	78.02	94.02	0.2692	0.297873531	0.351412891	--
AB5991_17055	2.8	0	3	0	0	0	1.93	0.00	-10.9169	0.139056913	0.176307472	--
AB5991_17060	1.36	1.66	1.61	8.54	8.45	4.74	1.54	7.24	2.2306	2.06E-07	5.00E-07	--
AB5991_17065	4.98	17.37	6.66	33.31	50.68	40.7	9.67	41.56	2.1037	3.33E-07	7.93E-07	ecsA
AB5991_17070	8	9.29	8.03	60.73	66.14	60.09	8.44	62.32	2.8844	5.03E-23	4.18E-22	CVOMT1
AB5991_17075	16.44	18.44	18.05	23.58	21.77	12.29	17.64	19.21	0.1230	0.690272438	0.744047734	--
AB5991_17080	36.15	49.94	49.7	58.79	47.3	28.7	45.26	44.93	-0.0107	0.989440214	1	--
AB5991_17085	36.96	36.05	31.13	82.9	59.39	38.84	34.71	60.38	0.7985	0.010332407	0.015413218	--
AB5991_17090	35.79	31.06	31.24	42.11	35.14	23.61	32.70	33.62	0.0402	0.898055709	0.93625078	--
AB5991_17095	14.22	17.58	14.45	98.44	77.08	73.91	15.42	83.14	2.4311	2.13E-17	1.15E-16	--
AB5991_17100	24.76	30.22	28.77	243.12	144.2	112.47	27.92	166.60	2.5772	9.33E-15	4.12E-14	bioF
AB5991_17110	615.02	771.54	699.54	122.12	143.34	148.23	695.37	137.90	-2.3342	5.69E-18	3.25E-17	mrgA
AB5991_17115	220.52	182.7	218.56	63.24	60.79	60.39	207.26	61.47	-1.7534	1.38E-11	4.76E-11	htrB
AB5991_17120	38.92	42.59	51.89	15.08	14.38	20.49	44.47	16.65	-1.4172	1.69E-06	3.77E-06	cssR
AB5991_17125	126.5	91.54	122.88	14.93	15.6	15.15	113.64	15.23	-2.8998	5.78E-24	5.14E-23	cssS
AB5991_17130	109.54	54.31	79.66	13.11	6.69	4.74	81.17	8.18	-3.3108	6.85E-13	2.65E-12	spxO
AB5991_17135	106.68	339.47	127.98	8.53	7.14	10.05	191.38	8.57	-4.4804	4.47E-22	3.55E-21	yuxN
AB5991_17140	24.46	35.6	25.19	155.11	139.19	98.61	28.42	130.97	2.2044	1.55E-13	6.18E-13	fumC
AB5991_17145	0	0	0	1.06	0	0	0.00	0.35	8.4649	1	1	yvzF
AB5991_17150	5.61	7.56	6.8	0.54	0.63	0.27	6.66	0.48	-3.7937	3.72E-16	1.83E-15	gerAA
AB5991_17155	1.48	3.02	4.93	0.18	0.34	0	3.14	0.17	-4.1807	3.10E-07	7.42E-07	gerAB
AB5991_17160	9.72	19.33	17.84	1.06	0.66	1.4	15.63	1.04	-3.9097	3.26E-17	1.74E-16	gerAC

AB5991_17165	56.56	63.66	44.98	61.82	57.85	56.3	55.07	58.66	0.0911	0.729627331	0.77948618	liaR
AB5991_17170	45.23	49.34	46.58	37.76	43.99	33.06	47.05	38.27	-0.2980	0.272338931	0.324178026	liaS
AB5991_17175	30.37	42.97	34.08	17.87	21.28	14.01	35.81	17.72	-1.0148	0.00081546	0.001399391	liaF
AB5991_17180	21.33	27.37	19.93	18.69	16.23	11.88	22.88	15.60	-0.5523	0.077387428	0.102537697	liaG
AB5991_17185	40.52	95.46	25.37	34.79	35.27	35.5	53.78	35.19	-0.6121	0.135507781	0.172800705	liaH
AB5991_17190	2.85	8.71	2.03	5.16	4.35	6.16	4.53	5.22	0.2055	0.698552653	0.751748669	liaI
AB5991_17195	19.23	24.06	21.02	92.95	83.35	66.92	21.44	81.07	1.9191	7.06E-12	2.49E-11	yvqJ
AB5991_17200	6.21	12.55	3.99	12.5	19.6	17.48	7.58	16.53	1.1239	0.006805394	0.010430035	yvqK
AB5991_17205	8.16	5.74	6.98	17.45	25.61	20.91	6.96	21.32	1.6153	1.81E-07	4.39E-07	yvrA
AB5991_17210	5.45	4.06	4.55	10.37	10.39	8.47	4.69	9.74	1.0559	0.001491825	0.002483873	yvrB
AB5991_17215	21.42	30.91	21.89	63.03	67.74	55.49	24.74	62.09	1.3274	2.67E-06	5.83E-06	yvrC
AB5991_17220	5.93	5.45	3.17	18.86	26.94	22.98	4.85	22.93	2.2410	2.09E-10	6.50E-10	yvrD
AB5991_17225	11.72	31.34	12.09	103.77	118.66	131.11	18.38	117.85	2.6804	2.03E-12	7.55E-12	yvrE
AB5991_17230	2.8	5.52	3.66	14.55	12.88	7.86	3.99	11.76	1.5586	2.01E-05	4.04E-05	yvrG
AB5991_17235	0	0.93	1.08	2.48	2.06	0.55	0.67	1.70	1.3405	0.118757681	0.153062886	yvrH
AB5991_17240	0	0	0	0	0	0	0.00	0.00	0.0000	1	1	rsoA
AB5991_17250	0	0	0	0	0	0	0.00	0.00	0.0000	1	1	yvrJ
AB5991_17255	4.99	8.02	6.84	0.85	1.43	1.01	6.62	1.10	-2.5930	7.37E-09	2.02E-08	oxdC
AB5991_17260	0	0	0	0	0	0	0.00	0.00	0.0000	1	1	yvrL
AB5991_17265	28.07	24.82	24.83	109.8	101.7	90.51	25.91	100.67	1.9582	2.28E-13	9.08E-13	yvrN
AB5991_17270	19.91	17.8	15.97	75.21	72.78	67.49	17.89	71.83	2.0051	3.95E-13	1.55E-12	yvrO
AB5991_17275	13.93	13.9	10.85	46.76	37.75	30.64	12.89	38.38	1.5739	5.45E-08	1.38E-07	yvrP
AB5991_17280	23.88	28.68	24.34	15.78	12.95	18.36	25.63	15.70	-0.7076	0.016317094	0.023813002	fhuC
AB5991_17285	17.88	20.68	26	8.75	7.64	8.9	21.52	8.43	-1.3521	1.10E-05	2.26E-05	fhuG
AB5991_17290	15.19	14.9	16.06	6.01	4.57	5.61	15.38	5.40	-1.5112	5.99E-07	1.39E-06	fhuB
AB5991_17295	17.16	29.76	14.48	79.42	75.49	79.87	20.47	78.26	1.9350	1.83E-09	5.26E-09	fhuD

AB5991_17300	3.59	3.06	2.19	0.84	1.04	0.42	2.95	0.77	-1.9424	9.18E-05	0.000172829	yvsH
AB5991_17305	76.91	124.75	137.09	19.52	27.4	31.92	112.92	26.28	-2.1032	1.96E-08	5.16E-08	sspJ
AB5991_17310	1.87	1.37	0.8	3.26	2.29	2.43	1.35	2.66	0.9820	0.191536725	0.236135466	yvsG
AB5991_17315	17.74	24.88	22.94	9.65	9.62	4.85	21.85	8.04	-1.4426	7.09E-06	1.50E-05	yvgJ
AB5991_17320	25.35	17.9	15.85	85.46	86.32	76.62	19.70	82.80	2.0714	4.85E-12	1.75E-11	yvgK
AB5991_17325	6.23	9.75	11.11	27.62	14.33	15.99	9.03	19.31	1.0968	0.003199813	0.005113263	yvgL
AB5991_17330	7.04	7.66	10.88	32.62	18.58	17.22	8.53	22.81	1.4194	0.000134249	0.000248418	yvgM
AB5991_17335	8.27	12.78	12.56	190.43	239.52	177.53	11.20	202.49	4.1759	5.22E-38	1.24E-36	yvgN
AB5991_17340	1206.85	2791.7	1515.73	12730.43	12870.69	12289.62	1838.09	12630.25	2.7806	5.54E-17	2.91E-16	yvgO
AB5991_17345	38.34	43.69	33.03	81.35	99.79	93.7	38.35	91.61	1.2562	1.75E-06	3.89E-06	nhaK
AB5991_17350	14.54	9.09	11.94	139.07	153.19	133.06	11.86	141.77	3.5798	8.06E-33	1.31E-31	cysI
AB5991_17355	4.67	5.48	3.51	53.09	47.36	30.78	4.55	43.74	3.2641	1.01E-21	7.64E-21	cysJ
AB5991_17360	21.92	24.7	21.53	26.46	24.05	22.81	22.72	24.44	0.1055	0.693022752	0.746606985	helD
AB5991_17365	34.9	77.06	49.77	16.72	10.57	9.9	53.91	12.40	-2.1206	5.78E-08	1.46E-07	--
AB5991_17370	5.61	13.02	6.63	2.25	2.4	1.6	8.42	2.08	-2.0149	0.000141511	0.000261004	yvgT
AB5991_17375	7.37	6.37	5.1	24.04	17.65	10.32	6.28	17.34	1.4650	0.000750311	0.001292059	bdbC
AB5991_17380	4.59	12.4	11.56	43.49	28.87	26.03	9.52	32.80	1.7850	5.63E-06	1.20E-05	bdbD
AB5991_17385	124.27	378	114.84	17.99	13.35	15.31	205.70	15.55	-3.7256	2.14E-16	1.07E-15	cadA
AB5991_17390	5.77	3.85	4.17	8.15	8.08	7.87	4.60	8.03	0.8054	0.0078805	0.011944071	copA
AB5991_17395	0.86	0	0	2.81	5.26	0.93	0.29	3.00	3.3875	0.037114922	0.051281612	copZ
AB5991_17400	1.77	2.17	0.63	6.42	5.41	7.03	1.52	6.29	2.0451	0.002504868	0.004073231	csoR
AB5991_17405	4.7	16.95	5.2	145.84	131.69	164.78	8.95	147.44	4.0421	2.54E-20	1.76E-19	iolW
AB5991_17415	4.79	3.69	3.28	6.46	5.85	6.01	3.92	6.11	0.6395	0.042080862	0.057661526	yvaC
AB5991_17425	0.5	0.92	1.61	4.91	1.53	1.63	1.01	2.69	1.4133	0.120190251	0.154708423	yvaE
AB5991_17430	1.58	4.63	1.01	6.18	3.53	4.78	2.41	4.83	1.0050	0.06374172	0.085368759	yvaF
AB5991_17435	66.62	58.03	68.56	85.31	78.21	79	64.40	80.84	0.3279	0.214958497	0.262565424	yvaG

AB5991_17440	21.04	5.27	10.23	39.52	36.01	40.37	12.18	38.63	1.6653	0.000149956	0.000275684	--
AB5991_17445	1.48	1.05	1.83	29.04	26.01	14.21	1.45	23.09	3.9896	4.90E-22	3.86E-21	yqcG
AB5991_17450	0	0	0	27.22	31.13	12.54	0.00	23.63	14.5283	7.47E-17	3.89E-16	--
AB5991_17455	13.63	16.24	11.51	15.86	17.52	9.58	13.79	14.32	0.0541	0.864336213	0.904898261	--
AB5991_17460	23.22	26.07	29.14	0.83	0.2	0.62	26.14	0.55	-5.5709	7.80E-35	1.51E-33	xlyA
AB5991_17465	0	0	0.91	0.93	0	0.46	0.30	0.46	0.6111	1	1	yqxH
AB5991_17470	0	0	0	0	0	0	0.00	0.00	0.0000	1	1	--
AB5991_17475	0.6	0	1.42	0	0.8	0	0.67	0.27	-1.3363	0.639695257	0.695191534	yomQ
AB5991_17480	0.51	2.99	1.78	1.96	1.27	0.2	1.76	1.14	-0.6223	0.479917033	0.539822237	xkdV
AB5991_17485	1.95	1.69	1.47	0.12	0.12	0	1.70	0.08	-4.4122	1.58E-08	4.21E-08	--
AB5991_17490	1.27	0.97	2.38	0.12	0.11	0	1.54	0.08	-4.3282	1.27E-06	2.87E-06	SAOUHSC_02979
AB5991_17495	0	0	0.23	0	0	0	0.08	0.00	-6.2605	1	1	--
AB5991_17500	1.35	1.37	1.05	0.76	0.33	0.71	1.26	0.60	-1.0666	0.014461318	0.021229536	TMP
AB5991_17505	0	0	0	0	0	0	0.00	0.00	0.0000	1	1	--
AB5991_17510	0	0	0	0	1.63	0	0.00	0.54	9.0857	0.273970018	0.325347926	--
AB5991_17515	0.3	0	0.32	0.32	1.2	0.64	0.21	0.72	1.8007	0.321968552	0.377149593	--
AB5991_17520	1.42	0	0	0.52	0	0	0.47	0.17	-1.4493	0.645224572	0.700433782	--
AB5991_17525	0.94	0.86	0	0	0	0	0.60	0.00	-9.2288	0.264520322	0.316391271	--
AB5991_17530	0.49	0	0	0.53	0.49	0.53	0.16	0.52	1.6614	0.636712662	0.692898323	--
AB5991_17535	0	0	0	0	1.06	0	0.00	0.35	8.4649	0.512696027	0.569081258	--
AB5991_17540	1.1	0	0.39	0.8	0.75	0	0.50	0.52	0.0570	1	1	--
AB5991_17545	0	0	0	0	0	1.24	0.00	0.41	8.6912	0.512634938	0.569081258	--
AB5991_17550	0.15	0.55	0.64	0	0.15	0	0.45	0.05	-3.1592	0.091064768	0.119464221	--
AB5991_17555	10.67	6.35	5.24	4.39	0.59	2.81	7.42	2.60	-1.5148	0.00742438	0.01130453	--
AB5991_17560	1.45	0.53	0.93	0.16	0.59	0.16	0.97	0.30	-1.6771	0.028824041	0.040473928	--
AB5991_17565	0	0	0	0	0	0	0.00	0.00	0.0000	1	1	--

AB5991_17570	1.27	3.3	0.9	1.15	1.51	0.92	1.82	1.19	-0.6116	0.331652876	0.387351051	--
AB5991_17575	0	2.49	0	0.37	0	0	0.83	0.12	-2.7505	0.308070259	0.362473184	--
AB5991_17580	2.62	2	2.33	0.24	0.22	0.24	2.32	0.23	-3.3116	0.002154747	0.003524094	--
AB5991_17585	0	1.77	0	1.57	0.49	0.52	0.59	0.86	0.5436	0.751218176	0.800613896	--
AB5991_17590	0	2.67	1.04	0.79	0.74	0	1.24	0.51	-1.2779	0.29375584	0.346864241	--
AB5991_17595	0.47	0.43	0.25	0	0	0.51	0.38	0.17	-1.1731	0.70312164	0.755340902	--
AB5991_17600	669.92	395.99	460.22	1745.83	1933.52	1834.45	508.71	1837.93	1.8532	7.60E-11	2.46E-10	--
AB5991_17610	4.79	5.86	1.28	0	0	0.43	3.98	0.14	-4.7941	8.39E-05	0.000158751	--
AB5991_17615	0	0	0	0	0	0	0.00	0.00	0.0000	1	1	--
AB5991_17620	2.7	8.26	5.77	0	0.92	0.97	5.58	0.63	-3.1460	0.007023329	0.010730912	--
AB5991_17625	2.58	2.37	1.84	0.47	1.75	2.33	2.26	1.52	-0.5775	0.46600287	0.525709488	yopX
AB5991_17630	3.8	1.99	5.22	0	0	0	3.67	0.00	-11.8416	5.37E-05	0.00010337	--
AB5991_17635	3.85	2.53	3.24	0.3	0.84	1.79	3.21	0.98	-1.7151	0.006826272	0.010457996	--
AB5991_17640	0	0	0	0	0	0.76	0.00	0.25	7.9849	1	1	--
AB5991_17645	0.44	0	0.47	0	0.45	0	0.30	0.15	-1.0159	1	1	--
AB5991_17650	0	0	0	0	0	1.23	0.00	0.41	8.6795	0.512528468	0.569081258	--
AB5991_17655	0	0.91	0	0	0	0	0.30	0.00	-8.2448	1	1	--
AB5991_17660	0.91	0	0	0	0	0	0.30	0.00	-8.2448	1	1	xtrA
AB5991_17665	0	4.26	1.24	0	0	0	1.83	0.00	-10.8403	0.142955218	0.180615709	--
AB5991_17670	0	4.71	0	0	0	0	1.57	0.00	-10.6165	0.273743642	0.325347926	--
AB5991_17675	8.89	2.42	9.15	0.72	0	0.71	6.82	0.48	-3.8387	2.74E-07	6.59E-07	--
AB5991_17680	2.27	0	1.22	0	0	0	1.16	0.00	-10.1840	0.264667394	0.316391271	--
AB5991_17685	0	0	0	0	0	0	0.00	0.00	0.0000	1	1	--
AB5991_17690	9.6	6	9.39	0	0.42	0.88	8.33	0.43	-4.2648	7.00E-14	2.88E-13	--
AB5991_17695	3.28	4.52	4.16	0	0	0	3.99	0.00	-11.9610	8.11E-12	2.85E-11	--
AB5991_17700	0	0	1.77	0	0	0	0.59	0.00	-9.2046	0.51224665	0.569081258	--

AB5991_17705	0.94	0	0	0	0	0	0.31	0.00	-8.2916	1	1	--
AB5991_17710	0.89	0	0	0.96	0	0	0.30	0.32	0.1092	1	1	--
AB5991_17715	10.87	14.97	19.86	2.96	2.77	3.92	15.23	3.22	-2.2436	1.41E-06	3.18E-06	--
AB5991_17720	4.2	10.29	35.96	12.19	1.43	1.52	16.82	5.05	-1.7365	0.074229712	0.098518111	--
AB5991_17725	85.37	116.36	114.12	19.1	12.53	25.77	105.28	19.13	-2.4601	2.46E-14	1.05E-13	--
AB5991_17740	8.83	9.16	7.39	55.93	57.8	45.28	8.46	53.00	2.6474	2.20E-15	1.01E-14	smpB
AB5991_17745	64.11	74.75	61.21	290	305.34	304.86	66.69	300.07	2.1697	1.02E-16	5.29E-16	rnr
AB5991_17750	27.08	23.74	25.04	127.61	157.89	130.97	25.29	138.82	2.4568	5.79E-18	3.31E-17	est
AB5991_17755	28.95	44.54	31.8	164.24	168.83	116.04	35.10	149.70	2.0927	4.77E-11	1.57E-10	secG
AB5991_17760	37.13	18.87	36.96	11.68	15.69	17.95	30.99	15.11	-1.0365	0.002265041	0.00369828	yvaM
AB5991_17765	0.77	4.26	0	0.84	0.79	0.84	1.68	0.82	-1.0260	0.752112939	0.801137468	yvzC
AB5991_17770	135.56	158.62	168.66	10.6	8.57	3.36	154.28	7.51	-4.3606	4.25E-33	7.08E-32	rghR
AB5991_17775	174.72	181.75	161.08	10.3	5.26	10.71	172.52	8.76	-4.3002	3.48E-37	8.08E-36	rghRB
AB5991_17780	165.27	243.59	206.89	14.43	24.75	25.73	205.25	21.64	-3.2458	7.53E-23	6.22E-22	yvaP
AB5991_17785	13.5	11.51	11.93	0.92	0.76	0.35	12.31	0.68	-4.1856	5.65E-26	5.88E-25	yvaQ
AB5991_17790	1.86	3.41	2.55	17.9	11.62	13.79	2.61	14.44	2.4695	2.65E-09	7.51E-09	opuBD
AB5991_17795	0.79	2.16	0.84	5.34	4.39	3.82	1.26	4.52	1.8380	0.000248974	0.000448787	opuBC
AB5991_17800	0.28	1.01	1.18	6.01	4.78	4.49	0.82	5.09	2.6291	6.71E-06	1.42E-05	opuBB
AB5991_17805	0	0	0.17	5.32	1.12	0.85	0.06	2.43	5.4223	3.52E-05	6.95E-05	opuBA
AB5991_17810	25.39	42.26	24.25	2.21	2.41	3.3	30.63	2.64	-3.5365	1.93E-16	9.72E-16	yvaV
AB5991_17815	6.16	7.38	9.45	18.64	10.63	12.46	7.66	13.91	0.8601	0.021435543	0.03075164	opuCD
AB5991_17820	11.69	4.37	10.81	23.71	10.69	15.02	8.96	16.47	0.8791	0.036127264	0.050003962	opuCC
AB5991_17825	9.67	8.63	10.34	20.14	8.44	9.57	9.55	12.72	0.4137	0.310824592	0.365497321	opuCB
AB5991_17830	12.49	12.2	10.15	19.26	6.12	12.33	11.61	12.57	0.1142	0.779713167	0.826545912	opuCA
AB5991_17835	25.27	26.17	20.09	4.58	1.98	2.81	23.84	3.12	-2.9324	1.21E-13	4.87E-13	opcR
AB5991_17840	9.09	16.7	10.03	0.93	2.6	3.08	11.94	2.20	-2.4380	6.64E-07	1.54E-06	yvbG

AB5991_17845	33.21	24.82	27.03	16.3	18.25	16.86	28.35	17.14	-0.7264	0.01358752	0.020035664	yvbH
AB5991_17850	0.26	0.47	0	1.41	0.53	0.84	0.24	0.93	1.9291	0.089078955	0.116936373	yvbI
AB5991_17855	2.99	4.21	3.3	2.82	2.13	1.19	3.50	2.05	-0.7741	0.05956973	0.079889024	yvbJ
AB5991_17860	15.26	25.86	17.15	29.78	20.71	23.29	19.42	24.59	0.3405	0.2910112	0.343930201	yvbK
AB5991_17865	287.69	348.32	243.67	2008.53	2080.31	1942.84	293.23	2010.56	2.7775	5.90E-25	5.64E-24	eno
AB5991_17870	205.81	201.6	167.75	1509.42	1501.61	1547.97	191.72	1519.67	2.9867	1.32E-29	1.76E-28	gpmI
AB5991_17875	109.11	120.65	91.32	916.35	889.13	904.1	107.03	903.19	3.0771	4.93E-29	6.26E-28	tpiA
AB5991_17880	104.03	124.35	104.23	861.64	824.94	818.97	110.87	835.18	2.9132	2.22E-27	2.58E-26	pgk
AB5991_17885	513.02	553.15	412.86	2009.1	1781.19	1338.18	493.01	1709.49	1.7939	5.79E-11	1.89E-10	gapA
AB5991_17890	26.86	41.2	32.31	197.73	149.07	115.14	33.46	153.98	2.2024	8.74E-13	3.35E-12	cggR
AB5991_17895	35.24	28.55	34.09	51.29	83.34	65.64	32.63	66.76	1.0329	0.000265527	0.000477107	araE
AB5991_17900	6.14	6.7	8.16	16.07	18.92	9.34	7.00	14.78	1.0779	0.00223841	0.003657912	araR
AB5991_17905	157.69	201.89	144.92	50.94	52.77	42.19	168.17	48.63	-1.7899	5.63E-11	1.84E-10	yvbT
AB5991_17910	59.22	84.58	51.24	2.01	3.77	2.89	65.01	2.89	-4.4916	2.20E-33	3.77E-32	yvbU
AB5991_17915	12.4	13.02	11.58	1.93	1.4	1.28	12.33	1.54	-3.0047	2.54E-14	1.08E-13	yvbV
AB5991_17920	10.09	17.53	15.39	59.97	51.05	47.02	14.34	52.68	1.8775	6.09E-10	1.81E-09	yvbW
AB5991_17930	43.25	50.95	52.13	265.1	141.21	120.7	48.78	175.67	1.8486	2.93E-08	7.61E-08	lutC
AB5991_17935	27.49	40.56	37.58	247.23	134.65	133.43	35.21	171.77	2.2864	2.99E-12	1.09E-11	lutB
AB5991_17940	5.04	6.94	4.58	110.49	73.63	61.4	5.52	81.84	3.8901	3.71E-26	3.91E-25	lutA
AB5991_17945	3.6	7.71	2.88	11.08	8.24	7.79	4.73	9.04	0.9340	0.025808445	0.036510629	yvfU
AB5991_17950	2.11	2.68	3.46	7.93	7.75	5.96	2.75	7.21	1.3912	0.000245814	0.000443896	yvfT
AB5991_17955	9.06	11.24	12.57	14.12	21.69	17.23	10.96	17.68	0.6903	0.033642887	0.046728193	yvfS
AB5991_17960	126.09	129.31	125.02	32.11	43.86	34.77	126.81	36.91	-1.7804	3.93E-11	1.30E-10	yvfR
AB5991_17965	0.35	1.28	0	2.27	1.06	0.75	0.54	1.36	1.3237	0.238212275	0.287957669	--
AB5991_17970	6.29	5.45	4.67	17.66	17.06	14.18	5.47	16.30	1.5753	6.03E-07	1.40E-06	--
AB5991_17975	7.75	14.22	5.06	16.85	19.71	23.29	9.01	19.95	1.1468	0.004346642	0.006822339	--

AB5991_17980	16.07	16.39	23.15	15.29	11.36	8.94	18.54	11.86	-0.6439	0.049796892	0.067420204	rsbQ
AB5991_17985	32.51	21.91	29.5	22.22	13.81	14.04	27.97	16.69	-0.7451	0.013268836	0.019602138	rsbP
AB5991_17990	14.29	17.49	11.39	18.9	21.39	17.29	14.39	19.19	0.4155	0.144576806	0.182316449	ganB
AB5991_17995	53.42	54.03	51.6	25.91	38.59	41.62	53.02	35.37	-0.5838	0.031599892	0.04413759	ganA
AB5991_18000	40.1	27.66	35.62	7.38	13.82	12.86	34.46	11.35	-1.6018	6.94E-07	1.60E-06	ganQ
AB5991_18005	32.93	25.35	35.52	7.04	4.83	8.09	31.27	6.65	-2.2325	9.56E-13	3.65E-12	ganP
AB5991_18010	28.98	23.33	26.72	9.16	14.68	16.69	26.34	13.51	-0.9634	0.00139587	0.002336846	ganS
AB5991_18015	120.68	95.02	142.6	15.35	11.23	13.32	119.43	13.30	-3.1667	5.87E-25	5.63E-24	ganR
AB5991_18020	8.5	7.8	6.68	26.37	34.35	28.69	7.66	29.80	1.9601	9.35E-10	2.74E-09	lutR
AB5991_18025	8.97	6.67	9.71	27.77	20.98	17.58	8.45	22.11	1.3877	4.56E-06	9.79E-06	lutP
AB5991_18030	118.02	129.36	111.76	339.48	363.15	411.92	119.71	371.52	1.6338	3.80E-10	1.15E-09	sigL
AB5991_18035	14.03	12.12	5.3	41.29	33.6	23.23	10.48	32.71	1.6415	0.000240016	0.00043382	yvfG
AB5991_18040	78.72	87	67.82	207.54	264.07	287.33	77.85	252.98	1.7003	3.90E-10	1.18E-09	epsO
AB5991_18045	63.04	75.65	48.2	237.18	296.19	316.21	62.30	283.19	2.1846	3.62E-14	1.52E-13	epsN
AB5991_18050	33.87	46.39	26.13	195.07	227.19	263.89	35.46	228.72	2.6892	2.97E-18	1.74E-17	epsM
AB5991_18055	31.16	44.14	23.8	140.09	192.72	182.17	33.03	171.66	2.3776	5.61E-14	2.33E-13	epsL
AB5991_18060	22.03	22.3	15.66	104.51	138.15	146.81	20.00	129.82	2.6987	4.06E-20	2.76E-19	epsK
AB5991_18065	39.87	46.12	27.05	244.15	316.79	290.16	37.68	283.70	2.9125	6.10E-22	4.76E-21	epsJ
AB5991_18070	21.15	24.65	14.9	115.54	163.12	177.86	20.23	152.17	2.9109	1.51E-20	1.07E-19	epsI
AB5991_18075	16.07	18.28	16.25	76.54	96.87	80.72	16.87	84.71	2.3284	5.42E-16	2.63E-15	epsH
AB5991_18080	25.21	28.56	23.29	128.92	127.64	127.07	25.69	127.88	2.3157	1.10E-17	6.16E-17	epsG
AB5991_18085	17.68	14.08	11.55	109.61	106.07	94.19	14.44	103.29	2.8389	4.04E-22	3.21E-21	epsF
AB5991_18090	2.38	2.38	2.77	54.49	62.2	45.35	2.51	54.01	4.4276	5.87E-34	1.05E-32	epsE
AB5991_18095	22.87	25.2	22.1	129.34	127.13	90.32	23.39	115.60	2.3051	4.44E-16	2.16E-15	epsD
AB5991_18100	6.05	9.84	5.63	38.86	40.31	27.4	7.17	35.52	2.3081	9.62E-13	3.67E-12	epsC
AB5991_18105	122.61	152.84	102.86	681.42	535.74	517.19	126.10	578.12	2.1968	2.05E-14	8.79E-14	yveL

AB5991_18110	5.64	14.59	5.76	51.86	66.54	29.97	8.66	49.46	2.5132	3.00E-09	8.48E-09	yveK
AB5991_18115	0.39	4.34	0.42	5.57	9.62	8.1	1.72	7.76	2.1771	0.000477956	0.000840551	slrR
AB5991_18120	16.6	15.58	14.07	141.35	161.31	134.7	15.42	145.79	3.2413	1.37E-30	1.96E-29	pnbA
AB5991_18125	3.77	1.73	5.03	17.41	20.12	13.25	3.51	16.93	2.2698	5.21E-05	0.000100464	--
AB5991_18130	81.82	48.49	59.26	1458.58	1597.34	1290.32	63.19	1448.75	4.5190	6.13E-45	2.35E-43	padC
AB5991_18140	249.72	304.72	229.18	37.65	22.86	24.89	261.21	28.47	-3.1978	4.47E-25	4.34E-24	racX
AB5991_18150	1.14	3.03	3.67	11.06	12.94	5.64	2.61	9.88	1.9186	2.59E-05	5.18E-05	sacB
AB5991_18155	8.04	13.48	8.35	3.17	2.49	2.78	9.96	2.81	-1.8234	3.54E-07	8.43E-07	levB
AB5991_18160	2.78	3.82	4.95	0.25	0.71	0.75	3.85	0.57	-2.7558	4.62E-08	1.18E-07	yveA
AB5991_18165	28.73	17.02	27.76	25.54	26.1	14.38	24.50	22.01	-0.1550	0.65339514	0.708335271	yvdT
AB5991_18170	9.68	5.93	9.78	9.95	8.76	6.99	8.46	8.57	0.0175	1	1	yvdS
AB5991_18175	15.2	11.37	16.26	16.53	14.9	7.31	14.28	12.91	-0.1448	0.776596931	0.823902328	yvdR
AB5991_18185	0.77	8.51	1.65	0.84	0	0	3.64	0.28	-3.7018	0.026726145	0.037714827	--
AB5991_18190	0.63	0	0	0	0	0	0.21	0.00	-7.7142	1	1	--
AB5991_18195	2.63	1.42	2.32	0.34	1.1	1.84	2.12	1.09	-0.9576	0.089975056	0.118073677	--
AB5991_18200	3.71	0	1.22	0.93	0	0	1.64	0.31	-2.4063	0.071813445	0.095566752	--
AB5991_18205	93.66	143.49	91.22	40.22	29.14	30.35	109.46	33.24	-1.7195	6.00E-08	1.52E-07	--
AB5991_18210	0	0	1.5	0	0	0	0.50	0.00	-8.9658	0.512178454	0.569081258	--
AB5991_18215	1.14	2.8	1.22	0.21	0.58	0.21	1.72	0.33	-2.3674	0.003612375	0.00573331	xlyB
AB5991_18220	0	0	0	0	0	0	0.00	0.00	0.0000	1	1	bhlA
AB5991_18225	0	0	0	0	1.08	0	0.00	0.36	8.4919	1	1	--
AB5991_18230	2.88	0	1.52	3.34	4.12	5.99	1.47	4.48	1.6120	0.036325885	0.050261355	--
AB5991_18235	2.39	5.25	2.71	1.92	2.11	1.89	3.45	1.97	-0.8060	0.104636469	0.136233252	--
AB5991_18240	1.07	5.86	2.94	0	0	0	3.29	0.00	-11.6839	1.07E-09	3.12E-09	--
AB5991_18245	0.26	0.71	0.42	0	0	0.14	0.46	0.05	-3.3116	0.03265292	0.045432637	--
AB5991_18250	0.13	1.17	0.27	0.42	0	0	0.52	0.14	-1.9023	0.188548021	0.232667554	lytG

AB5991_18255	0.19	0.12	0.07	0.07	0.07	0.07	0.13	0.07	-0.8556	0.741330721	0.791137945	TMP
AB5991_18260	0	0	0	0	0	0	0.00	0.00	0.0000	1	1	--
AB5991_18265	0	0	0	0	0	0	0.00	0.00	0.0000	1	1	--
AB5991_18270	0.32	0	0	0	0	0	0.11	0.00	-6.7370	1	1	--
AB5991_18275	0.48	1.77	4.64	0	0	0	2.30	0.00	-11.1653	0.003050266	0.004899921	--
AB5991_18280	4.43	8.13	2.84	0.96	0	1.44	5.13	0.80	-2.6818	0.001178598	0.001996678	--
AB5991_18285	4.2	3.86	3	0	0	0	3.69	0.00	-11.8481	0.000560866	0.000981144	--
AB5991_18290	0.46	5.07	1.97	0	0	0	2.50	0.00	-11.2877	0.001994468	0.003271388	--
AB5991_18295	0	0	1.01	0	0	0	0.34	0.00	-8.3952	1	1	--
AB5991_18300	0.4	0.73	1.7	0.43	0.2	0	0.94	0.21	-2.1674	0.070862542	0.094427904	--
AB5991_18305	0	0	0	0	0	0	0.00	0.00	0.0000	1	1	--
AB5991_18310	1.78	2.86	1.9	0.97	1.13	1.2	2.18	1.10	-0.9868	0.122873108	0.157854775	--
AB5991_18315	1.41	2.18	1.5	0.82	0.22	0.12	1.70	0.39	-2.1335	0.00062504	0.001086704	--
AB5991_18320	0.42	0	0.44	0.45	0.42	0	0.29	0.29	0.0167	1	1	--
AB5991_18325	0.31	0.38	0.55	0.22	0.1	0	0.41	0.11	-1.9542	0.121730593	0.156589629	--
AB5991_18330	112.6	118.49	133.44	8.59	4.36	6.77	121.51	6.57	-4.2083	1.57E-35	3.30E-34	yoeC
AB5991_18335	2.58	0	1.84	0	3.5	0	1.47	1.17	-0.3367	1	1	--
AB5991_18345	2.32	4.26	4.34	2.52	1.18	2.51	3.64	2.07	-0.8143	0.291253447	0.344114085	--
AB5991_18350	1	0	1.07	0	0	0	0.69	0.00	-9.4305	0.509536392	0.568309168	--
AB5991_18355	2	3.06	1.42	9.05	12.2	9.37	2.16	10.21	2.2404	1.03E-06	2.34E-06	--
AB5991_18360	1.3	4.76	2.08	5.64	7.91	7.01	2.71	6.85	1.3367	0.029925784	0.041961613	--
AB5991_18365	5.93	6.29	5.61	17.37	20.9	18.03	5.94	18.77	1.6588	2.08E-07	5.02E-07	--
AB5991_18370	0.49	4.46	1.56	0	2.97	0.53	2.17	1.17	-0.8953	0.487751713	0.547445465	--
AB5991_18375	2.35	4.32	3.02	2.05	1.92	2.55	3.23	2.17	-0.5716	0.514637303	0.570685487	--
AB5991_18380	0	0	0	0	2.22	0.79	0.00	1.00	9.9706	0.142384448	0.179951828	--
AB5991_18385	2.01	1.84	1.07	0	0	0	1.64	0.00	-10.6795	0.136734283	0.174085232	--

AB5991_18390	1.06	2.91	2.83	0	0	0.57	2.27	0.19	-3.5765	0.01220636	0.018066141	--
AB5991_18395	0	0	0	0	0	0	0.00	0.00	0.0000	1	1	--
AB5991_18400	0	0	0	0	0	0	0.00	0.00	0.0000	1	1	--
AB5991_18405	2	0	1.99	1.09	0.68	0	1.33	0.59	-1.1726	0.306071277	0.36044159	--
AB5991_18410	0	2.41	1.4	0	0	0	1.27	0.00	-10.3106	0.509713011	0.568309168	--
AB5991_18415	0	4.77	1.58	3.2	5.68	3.04	2.12	3.97	0.9086	0.21930147	0.267294701	--
AB5991_18420	23.43	24.58	42.06	257.56	204.4	140.41	30.02	200.79	2.7415	2.98E-14	1.26E-13	--
AB5991_18425	0	0	0	0.59	0.55	0.7	0.00	0.61	9.2605	0.261986026	0.3141587	--
AB5991_18430	0	1.42	0	0	3.14	0	0.47	1.05	1.1449	0.426244081	0.483880859	--
AB5991_18435	34.57	21.76	23.24	82.71	64.34	51.32	26.52	66.12	1.3179	0.000235603	0.000426426	--
AB5991_18440	1.98	1.81	1.06	0.27	0	0.8	1.62	0.36	-2.1804	0.019018521	0.027522794	--
AB5991_18445	0.18	0.67	0.39	0.4	0	0	0.41	0.13	-1.6323	0.325936379	0.381459876	xerC
AB5991_18455	81.25	163.71	98.6	598.68	603.91	540.87	114.52	581.15	2.3433	8.09E-14	3.32E-13	clpP
AB5991_18460	43.92	41.79	39.8	18.64	32.7	21.45	41.84	24.26	-0.7860	0.0085051	0.012841731	yvdM
AB5991_18465	32.91	40.16	26.6	14.11	20.73	21.59	33.22	18.81	-0.8207	0.005519972	0.008539077	malL
AB5991_18470	9.38	10.22	8.08	3.28	7.44	5.94	9.23	5.55	-0.7325	0.03219609	0.044875632	mdxK
AB5991_18475	4.49	4.88	5.24	2.67	4.78	5.53	4.87	4.33	-0.1707	0.706742687	0.758097032	yvdJ
AB5991_18480	6.48	7.14	7.39	3.76	4.4	3.27	7.00	3.81	-0.8783	0.018508023	0.026842717	mdxG
AB5991_18485	8.13	8.61	6.19	4.2	6.88	6.27	7.64	5.78	-0.4023	0.248563984	0.299286713	mdxF
AB5991_18490	9.95	8.2	7.55	10.19	9.39	11.86	8.57	10.48	0.2908	0.342911039	0.399559782	mdxE
AB5991_18495	5.63	4.13	3.17	10.12	13.95	13.51	4.31	12.53	1.5392	3.74E-06	8.10E-06	bbmA
AB5991_18500	5.13	4.89	4.47	16.95	20.89	20.99	4.83	19.61	2.0215	1.77E-10	5.53E-10	yvdE
AB5991_18505	0.31	2.88	2.35	21.84	16.29	15.29	1.85	17.81	3.2694	7.21E-12	2.54E-11	yvdD
AB5991_18515	62.18	97.09	54	158.2	131.53	124.91	71.09	138.21	0.9592	0.001015848	0.001730559	yvdB
AB5991_18520	33.17	51.4	28.96	116.49	88.88	72.8	37.84	92.72	1.2929	4.57E-05	8.89E-05	yvdA
AB5991_18525	97.03	129.97	85.18	43.01	42.89	35.61	104.06	40.50	-1.3613	1.21E-06	2.73E-06	yvcT

AB5991_18530	12.66	14.53	15.04	3.34	4.93	4.64	14.08	4.30	-1.7098	1.17E-08	3.14E-08	yvcS
AB5991_18535	11.35	10.21	12.14	7.81	9.43	3.76	11.23	7.00	-0.6824	0.069902417	0.093179758	yvcR
AB5991_18540	4.22	3.72	3.07	6.79	4.98	4.2	3.67	5.32	0.5365	0.173738579	0.215464053	yvcQ
AB5991_18545	0	0.46	0.81	2.48	3.61	1.37	0.42	2.49	2.5543	0.002527033	0.004105911	yvcP
AB5991_18555	173.19	199.57	169.8	144.93	147.65	122	180.85	138.19	-0.3881	0.128533113	0.164753064	yvcN
AB5991_18560	33.63	33.45	38.21	36.57	43.49	33.37	35.10	37.81	0.1074	0.749691636	0.799201473	crh
AB5991_18565	111.57	144.83	103.25	72.14	72.35	53.7	119.88	66.06	-0.8597	0.001727821	0.002850509	whiA
AB5991_18570	178.29	211.17	150.95	113.13	92.94	90.24	180.14	98.77	-0.8669	0.001128355	0.001916466	mgfK
AB5991_18575	118.67	168.19	119.72	68.85	53.45	42.75	135.53	55.02	-1.3006	9.23E-06	1.91E-05	yvcJ
AB5991_18580	77.68	110.63	77.8	9.48	10.41	13.54	88.70	11.14	-2.9928	6.37E-20	4.27E-19	yvcI
AB5991_18585	89.01	155.6	90	60.45	72.4	47.55	111.54	60.13	-0.8913	0.003928723	0.006195774	trxB
AB5991_18590	2.44	1.41	1.37	139	186.96	65.08	1.74	130.35	6.2271	4.67E-38	1.12E-36	cwlO
AB5991_18595	133.29	137.09	124.08	32.7	27.94	15.87	131.49	25.50	-2.3662	2.53E-14	1.08E-13	yvcD
AB5991_18600	59.33	49.13	57.66	28.1	25.15	18.57	55.37	23.94	-1.2098	1.15E-05	2.36E-05	bmrA
AB5991_18605	179.73	279.39	180.19	12.77	8.83	10.5	213.10	10.70	-4.3159	3.58E-33	6.06E-32	yvzA
AB5991_18610	236.45	291.19	222.37	11.54	13.02	13.13	250.00	12.56	-4.3147	1.63E-45	6.47E-44	yvcB
AB5991_18615	53	61.05	44.38	0.82	1.02	2.16	52.81	1.33	-5.3077	6.75E-37	1.54E-35	yvcA
AB5991_18620	551.43	860.81	554.72	17.47	25.4	25.78	655.65	22.88	-4.8406	5.12E-44	1.80E-42	hisI
AB5991_18625	474.14	631.42	435.99	16.58	23.27	20.88	513.85	20.24	-4.6658	2.25E-46	9.39E-45	hisF
AB5991_18630	532.45	704.25	516.24	13.05	17.95	19.62	584.31	16.87	-5.1139	3.11E-52	2.10E-50	hisA
AB5991_18635	516.51	634.18	505.47	11.07	5.18	10.72	552.05	8.99	-5.9403	7.11E-59	7.06E-57	hisH
AB5991_18640	329.06	428.34	367.75	2.02	1.89	6.35	375.05	3.42	-6.7769	2.66E-60	2.93E-58	hisB
AB5991_18645	369.8	473.54	418.8	1.84	1.29	4.57	420.71	2.57	-7.3568	5.64E-64	9.74E-62	hisD
AB5991_18650	389.09	555.22	382.97	0.92	0.86	4.88	442.43	2.22	-7.6387	2.07E-58	1.96E-56	hisG
AB5991_18655	340.29	526.34	363.58	0.67	1.41	1.83	410.07	1.30	-8.2975	6.10E-79	4.04E-76	hisZ
AB5991_18660	150.74	181.59	142.98	1.04	1.22	1.56	158.44	1.27	-6.9592	9.50E-63	1.45E-60	yvpB

AB5991_18665	195.68	347.84	160.21	2878.46	2896.11	2896.73	234.58	2890.43	3.6232	2.49E-27	2.87E-26	pelC
AB5991_18670	0.45	0.62	0.61	1.39	0.97	0.74	0.56	1.03	0.8838	0.223198502	0.270880578	insK
AB5991_18680	103.56	117.26	126.78	17.51	31.93	20.44	115.87	23.29	-2.3145	1.42E-13	5.71E-13	ppaX
AB5991_18685	14.63	19.79	16.47	23.24	22.53	19.59	16.96	21.79	0.3610	0.20038224	0.245997346	yvoD
AB5991_18690	2.23	4.1	3.1	23.3	18.17	16.43	3.14	19.30	2.6182	2.68E-12	9.82E-12	lgt
AB5991_18695	1.55	2.13	3.31	17.7	16.56	11.53	2.33	15.26	2.7117	1.12E-11	3.87E-11	hprK
AB5991_18700	13.81	14.49	15.42	36.48	35.99	22.18	14.57	31.55	1.1143	0.000146152	0.000269313	nagA
AB5991_18705	28.02	23.67	24.92	93.84	95.64	88.57	25.54	92.68	1.8597	1.06E-11	3.68E-11	nagB
AB5991_18710	23.7	27.66	20.07	103.39	129.68	106.65	23.81	113.24	2.2497	1.65E-14	7.16E-14	nagR
AB5991_18715	169.87	129.58	146.39	1.73	2.52	3.03	148.61	2.43	-5.9364	3.29E-63	5.22E-61	yvnB
AB5991_18720	1.46	2.49	2.28	9.07	7.18	5.16	2.08	7.14	1.7810	8.69E-06	1.81E-05	--
AB5991_18725	77.82	106.02	91.28	48.05	68.98	58.15	91.71	58.39	-0.6512	0.020580364	0.029599647	yvlD
AB5991_18730	12.78	28.5	23.43	7.94	10.22	11.86	21.57	10.01	-1.1081	0.020533772	0.029560452	yvlC
AB5991_18735	125.77	210.08	147.35	64.27	66.18	64.51	161.07	64.99	-1.3094	2.98E-06	6.49E-06	yvlB
AB5991_18740	13.82	18.27	11.82	3.01	2.81	2.99	14.64	2.94	-2.3173	1.78E-06	3.96E-06	yvlA
AB5991_18745	0.78	0	2.51	0	1.59	0.85	1.10	0.81	-0.4312	1	1	yvkN
AB5991_18750	6.38	7.67	5.17	12.44	9.62	10.47	6.41	10.84	0.7592	0.024077529	0.034281774	hag
AB5991_18755	101.38	104.86	87.57	280.17	280.23	292.68	97.94	284.36	1.5378	1.51E-09	4.35E-09	uvrA
AB5991_18760	28.21	36.77	31.34	58.8	45.02	56.35	32.11	53.39	0.7337	0.006012313	0.009261014	uvrB
AB5991_18765	6.26	12.93	7.53	37.44	24.69	23.72	8.91	28.62	1.6839	9.49E-05	0.000178213	csbA
AB5991_18770	3.77	3.32	3.79	54.58	99.72	91.56	3.63	81.95	4.4981	2.53E-38	6.32E-37	yvkC
AB5991_18775	3.49	5.82	2.37	73.46	115.86	120.83	3.89	103.38	4.7309	1.25E-32	2.01E-31	yvkB
AB5991_18780	5.82	5.97	6.37	31.07	46.85	49.83	6.05	42.58	2.8145	4.75E-19	2.93E-18	yvkA
AB5991_18785	20.89	24.46	19.75	52.68	57.47	45.56	21.70	51.90	1.2581	3.85E-06	8.32E-06	minJ
AB5991_18790	14.3	7.5	16.38	12.22	22.86	4.97	12.73	13.35	0.0690	0.933305202	0.970660887	swrAA
AB5991_18795	52.86	56.58	59.48	5.59	3.44	5.02	56.31	4.68	-3.5877	4.43E-30	6.19E-29	ctpB

AB5991_18800	20.08	21.6	21.91	95.31	85.68	70.05	21.20	83.68	1.9810	6.29E-12	2.23E-11	ftsX
AB5991_18805	36.57	38.16	34.33	48.36	50.34	21.93	36.35	40.21	0.1455	0.659619614	0.714692902	ftsE
AB5991_18810	37.32	53.85	33.07	58.57	56.98	33.48	41.41	49.68	0.2625	0.423747697	0.481184474	cccB
AB5991_18815	1.28	2.75	1.83	6.51	6.09	3.47	1.95	5.36	1.4554	0.002265907	0.00369828	yvjA
AB5991_18820	73.71	65.41	59.34	424.94	330.99	315.08	66.15	357.00	2.4321	4.10E-18	2.39E-17	prfB
AB5991_18825	141.89	152.94	134.07	728.35	429.38	490.78	142.97	549.50	1.9424	2.10E-11	7.13E-11	secA
AB5991_18830	4551.07	4886.35	3212.07	19711.89	19317.62	18239.41	4216.50	19089.64	2.1787	1.43E-16	7.30E-16	yvyD
AB5991_18835	1.91	0	2.56	1.04	0	0.52	1.49	0.52	-1.5187	0.285968189	0.338675717	yvzG
AB5991_18840	20.61	17.47	9.61	2.87	5.92	5.72	15.90	4.84	-1.7166	0.000369656	0.000658254	fliT
AB5991_18845	9.44	6.6	10.1	1.47	4.58	1.95	8.71	2.67	-1.7082	0.000814935	0.001399095	fliS
AB5991_18850	7.73	5.54	5.94	1.84	1.72	1.83	6.40	1.80	-1.8335	5.39E-07	1.26E-06	fliD
AB5991_18855	0	0	0	0	0	0	0.00	0.00	0.0000	1	1	yvyC
AB5991_18860	19.69	18.08	14.95	122.06	102.8	102.31	17.57	109.06	2.6336	2.03E-19	1.30E-18	hag
AB5991_18865	7.23	8.85	5.15	4.37	0.82	0.87	7.08	2.02	-1.8087	0.014220379	0.020906747	csrA
AB5991_18870	5.86	9.99	4.92	0.91	1.28	3.62	6.92	1.94	-1.8379	0.002931692	0.004724735	fliW
AB5991_18875	2.2	2.3	2.68	1.37	3.19	2.38	2.39	2.31	-0.0490	1	1	yviE
AB5991_18880	7.46	3.7	6.68	10.52	11.07	9.6	5.95	10.40	0.8060	0.023425838	0.0334258	flgL
AB5991_18885	4.03	3.7	4.44	8.64	8.57	7.45	4.06	8.22	1.0188	0.000977602	0.001668984	flgK
AB5991_18890	18.71	21.99	29.21	5.29	4.57	4.86	23.30	4.91	-2.2477	3.07E-09	8.65E-09	yvyG
AB5991_18895	13.54	7.46	11.58	4.42	1.38	5.13	10.86	3.64	-1.5757	0.004145476	0.006516899	flgM
AB5991_18900	27.97	20.55	17.95	2.34	3.94	4.19	22.16	3.49	-2.6664	2.95E-10	9.05E-10	yvyF
AB5991_18905	17.75	37.18	17.38	5.17	8.14	6.22	24.10	6.51	-1.8885	2.76E-06	6.03E-06	comFC
AB5991_18910	1.83	3.35	2.6	0.66	0	1.98	2.59	0.88	-1.5592	0.160413072	0.200125765	comFB
AB5991_18915	5.84	6.2	5.97	6.64	5.68	6.04	6.00	6.12	0.0278	0.936899015	0.973446947	comFA
AB5991_18920	5.34	7.06	4.57	24.86	25.66	8.33	5.66	19.62	1.7941	4.76E-05	9.24E-05	degV
AB5991_18925	14.93	22.61	17.93	922.51	938.46	492.56	18.49	784.51	5.4070	1.49E-45	5.99E-44	degU

AB5991_18930	16.39	17.2	14.86	32.76	34.47	29.06	16.15	32.10	0.9909	0.000155349	0.000284938	degS
AB5991_18935	82.36	84.75	78.32	13.53	15.75	8.68	81.81	12.65	-2.6928	5.88E-19	3.61E-18	yvyE
AB5991_18940	118.68	93.58	92.66	31.67	29.09	18.32	101.64	26.36	-1.9470	8.24E-11	2.65E-10	tagV
AB5991_18945	5.21	2.29	6	43.97	49.97	50.39	4.50	48.11	3.4183	4.25E-20	2.88E-19	--
AB5991_18950	7.77	8.58	7.87	59.49	99.52	79.06	8.07	79.36	3.2971	9.45E-21	6.80E-20	--
AB5991_18955	27.93	35.17	32.43	99.38	103.55	86.38	31.84	96.44	1.5986	3.50E-09	9.82E-09	--
AB5991_18960	0	0	0	1.51	0.71	1.25	0.00	1.16	10.1758	0.000654481	0.001134414	--
AB5991_18965	0.23	0.42	0	0	0.23	0	0.22	0.08	-1.4988	1	1	--
AB5991_18970	7.39	9.55	7.03	0.89	1.11	0.3	7.99	0.77	-3.3815	8.44E-10	2.48E-09	yybJ
AB5991_18975	26.62	52.74	36.71	5.71	2.5	1.9	38.69	3.37	-3.5211	4.40E-15	1.99E-14	--
AB5991_18980	249.89	242.52	304.75	55.49	45.61	43.06	265.72	48.05	-2.4672	4.41E-19	2.73E-18	tagO
AB5991_18985	18.62	30.02	22.83	10.04	12.02	14.58	23.82	12.21	-0.9639	0.001678179	0.00277553	tuaH
AB5991_18990	3.81	2.19	2.29	9.32	6.79	4.64	2.76	6.92	1.3237	0.00434174	0.006817338	tuaG
AB5991_18995	3.21	4.43	3.44	4.95	8.72	3.19	3.69	5.62	0.6056	0.22050317	0.268676922	tuaF
AB5991_19000	1.24	1.82	2.26	0.68	1.64	1.34	1.77	1.22	-0.5396	0.292431854	0.345403597	tuaE
AB5991_19005	27.23	23.51	21.19	34.74	41.84	39.83	23.98	38.80	0.6945	0.00975145	0.014623493	tuaD
AB5991_19010	13.99	16.65	12.16	7.36	11.73	6.16	14.27	8.42	-0.7613	0.02462094	0.035005281	tuaC
AB5991_19015	24.23	33.19	24.83	18.84	22.23	17.8	27.42	19.62	-0.4825	0.080834309	0.106784112	tuaB
AB5991_19020	15.61	21.62	19.62	4.47	2.51	2.67	18.95	3.22	-2.5586	4.17E-11	1.38E-10	tuaA
AB5991_19030	2.43	1.12	1.82	4.89	4.08	3.29	1.79	4.09	1.1910	0.003934045	0.006201705	lytC
AB5991_19035	1.35	1.39	0.9	21.45	23.41	16.6	1.21	20.49	4.0776	9.35E-30	1.28E-28	lytB
AB5991_19040	0.56	0	0.6	15.31	10.32	17.07	0.39	14.23	5.2020	1.02E-11	3.56E-11	lytA
AB5991_19050	1.46	2.49	2.28	9.07	7.18	5.16	2.08	7.14	1.7810	8.68E-06	1.81E-05	--
AB5991_19055	0	0	0	0	0	0	0.00	0.00	0.0000	1	1	--
AB5991_19060	1.46	2.49	2.28	9.07	7.18	5.16	2.08	7.14	1.7810	8.77E-06	1.83E-05	--
AB5991_19065	50.6	51.4	46.67	20.47	14.97	8.22	49.56	14.55	-1.7677	8.50E-08	2.12E-07	mnaA

AB5991_19070	39.69	64.19	47.94	386.23	453.3	401.78	50.61	413.77	3.0314	1.74E-24	1.62E-23	gtaB
AB5991_19085	91.9	101.01	87.54	33.48	27.55	13.71	93.48	24.91	-1.9078	1.63E-08	4.33E-08	tagG
AB5991_19090	0.59	1.09	0	0.65	0.81	0.21	0.56	0.56	-0.0086	1	1	--
AB5991_19095	137.96	137.03	119.79	16.93	16.7	14.96	131.59	16.20	-3.0223	4.10E-28	4.90E-27	tagF
AB5991_19100	247.94	271.29	217.55	53.19	54.77	47.91	245.59	51.96	-2.2409	4.81E-17	2.54E-16	tagF
AB5991_19105	18.07	51.06	25.28	197.08	157.07	102.85	31.47	152.33	2.2752	5.54E-09	1.53E-08	tagD
AB5991_19110	44.03	56.59	44.85	109.38	97.88	62.96	48.49	90.07	0.8934	0.003080238	0.004940074	tagA
AB5991_19115	57.92	49.33	56.29	84.95	76.02	57.82	54.51	72.93	0.4199	0.12025978	0.15474776	tagB
AB5991_19120	311.99	365.79	319.45	11.31	12.04	12.14	332.41	11.83	-4.8124	4.65E-59	4.73E-57	lytD
AB5991_19125	46.88	70.43	38.33	71.01	70.98	72.14	51.88	71.38	0.4603	0.117842392	0.152031234	yvyI
AB5991_19130	3.61	5.03	5.46	0.95	0.13	0.94	4.70	0.67	-2.8033	2.77E-08	7.21E-08	gerBA
AB5991_19135	3.43	1.2	1.92	0	0.17	0	2.18	0.06	-5.2679	8.93E-07	2.04E-06	gerBB
AB5991_19140	6.43	2.36	7.04	0.35	1.47	1.04	5.28	0.95	-2.4686	1.10E-05	2.25E-05	gerBC
AB5991_19150	122.88	120.05	133.56	17.13	19.57	13.68	125.50	16.79	-2.9017	5.70E-24	5.07E-23	tagT
AB5991_19155	0.63	3.47	2.02	9.36	7.26	4.09	2.04	6.90	1.7587	0.000862412	0.001476137	ywtE
AB5991_19160	0.58	0.8	1.56	3.64	6.52	3.94	0.98	4.70	2.2618	7.32E-06	1.54E-05	pgdS
AB5991_19165	3.23	3.95	1.15	1.17	1.09	0	2.78	0.75	-1.8820	0.319314881	0.374372422	ywtC
AB5991_19170	3.48	4.65	3.55	2.41	6.12	5.14	3.89	4.56	0.2270	0.614368199	0.670051118	capA
AB5991_19175	0	2.95	1.72	0.87	1.64	0.43	1.56	0.98	-0.6676	0.671920729	0.72564515	capC
AB5991_19180	3.67	4.21	4.74	3.83	6.85	4.3	4.21	4.99	0.2473	0.552041065	0.608931964	capB
AB5991_19185	4.93	10.73	4.69	14.3	6.69	6.32	6.78	9.10	0.4244	0.335132872	0.391185371	rbsR
AB5991_19190	41.19	59.45	35.5	32.99	27.32	39.49	45.38	33.27	-0.4480	0.136217544	0.173650038	rbsK
AB5991_19195	5.02	4.19	3.9	9.93	21.37	21.25	4.37	17.52	2.0030	2.58E-05	5.15E-05	rbsD
AB5991_19200	19.15	20.16	12.78	38.47	60.2	67.46	17.36	55.38	1.6732	2.10E-07	5.08E-07	rbsA
AB5991_19205	18.28	23.63	10.97	26.17	33.41	28.67	17.63	29.42	0.7389	0.020153204	0.029048411	rbsC
AB5991_19210	28.94	32.9	23.16	16.92	25.25	31.12	28.33	24.43	-0.2138	0.506357761	0.566248006	rbsB

AB5991_19215	115.45	241.66	102.23	1208.4	1451.8	1411.53	153.11	1357.24	3.1480	1.32E-18	7.91E-18	ywsB
AB5991_19220	36.51	48.05	37.75	89.35	38.4	50.07	40.77	59.27	0.5399	0.137377555	0.174624286	ywsA
AB5991_19225	11.98	18.23	8.79	36.86	25.09	20.38	13.00	27.44	1.0779	0.003509881	0.005572865	ywrO
AB5991_19230	4.94	12.1	5.54	344.53	437.14	325.85	7.53	369.17	5.6161	1.82E-48	9.41E-47	alsD
AB5991_19235	7.07	10.66	7.45	240.3	287.48	204.91	8.39	244.23	4.8629	3.40E-48	1.71E-46	alsS
AB5991_19240	2.98	5.84	5.1	8.22	8.3	7.1	4.64	7.87	0.7628	0.041443642	0.056827591	alsR
AB5991_19245	1590.43	1336.8	1965.08	118.04	200.98	258.08	1630.77	192.37	-3.0836	3.89E-20	2.65E-19	ywrK
AB5991_19250	11.46	10.28	11.4	2.61	5.97	7.21	11.05	5.26	-1.0696	0.006940858	0.010613071	ywrJ
AB5991_19255	11.54	12.95	15.08	9.41	30.5	43.02	13.19	27.64	1.0675	0.015912029	0.023264604	cotB
AB5991_19260	5.98	3.35	7.63	2.17	3.21	4.13	5.65	3.17	-0.8346	0.041398013	0.056784632	cotH
AB5991_19265	32286.03	24440.73	61322.14	2833.1	3350.17	5300.42	39349.63	3827.90	-3.3617	2.35E-18	1.39E-17	cotG
AB5991_19270	221.11	400.09	205.8	35.94	31.26	41.48	275.67	36.23	-2.9278	2.58E-18	1.52E-17	ywrF
AB5991_19275	41.42	35.56	38.54	4.68	1.09	2.33	38.51	2.70	-3.8341	2.20E-18	1.30E-17	ywrE
AB5991_19280	2.06	2.73	1.1	0.25	0.23	0	1.96	0.16	-3.6172	1.02E-06	2.34E-06	ywrD
AB5991_19285	203.11	170.47	180.33	7.42	3.47	3.69	184.64	4.86	-5.2476	6.14E-45	2.35E-43	ywrC
AB5991_19290	233.7	195.56	252.19	6.29	5.57	6.92	227.15	6.26	-5.1813	1.72E-52	1.18E-50	ywrB
AB5991_19295	232.25	205.81	242.97	8.42	11.3	8.74	227.01	9.49	-4.5807	3.38E-45	1.31E-43	ywrA
AB5991_19300	0.81	0	0	0	0	0	0.27	0.00	-8.0768	1	1	ywqO
AB5991_19305	1.32	1.22	1.42	0.36	2.36	1.79	1.32	1.50	0.1876	0.856079494	0.897674061	ywqN
AB5991_19310	7.38	8.65	7.23	0.89	1.04	1.11	7.75	1.01	-2.9357	6.92E-11	2.25E-10	ywqM
AB5991_19320	0.51	0	0.55	1.11	0	0	0.35	0.37	0.0665	1	1	--
AB5991_19325	0	0	0	0	0	0	0.00	0.00	0.0000	1	1	--
AB5991_19335	0	3.16	0.92	0	0.88	0	1.36	0.29	-2.2130	0.402968456	0.460353205	--
AB5991_19340	13.58	8.95	10.8	3.41	3.9	3.77	11.11	3.69	-1.5889	9.15E-05	0.000172192	--
AB5991_19345	77.21	71.25	75.63	26.15	21.22	20.84	74.70	22.74	-1.7160	6.98E-11	2.26E-10	ywqJ
AB5991_19350	7.62	2.54	12.59	9.04	7.75	7.5	7.58	8.10	0.0945	1	1	ywqI

AB5991_19355	26.49	14.12	17.36	11.62	11.31	15.73	19.32	12.89	-0.5845	0.103856871	0.135326966	ywqH
AB5991_19370	25.55	52.93	27.03	15.75	17.1	11.24	35.17	14.70	-1.2589	0.000248952	0.000448787	ywqF
AB5991_19375	4.02	5.64	3.28	6.68	7.7	9.46	4.31	7.95	0.8815	0.019212525	0.027783298	ywqE
AB5991_19380	10.08	11.57	8.09	21.93	21.3	10.37	9.91	17.87	0.8498	0.020052587	0.028914987	ywqD
AB5991_19385	3.39	8.89	4.66	20.26	17.24	6.81	5.65	14.77	1.3872	0.003715509	0.005885237	ywqC
AB5991_19390	32.64	48.4	81.88	6.83	10.22	5.44	54.31	7.50	-2.8568	4.95E-08	1.26E-07	ywzD
AB5991_19395	15.26	15.66	14.76	10.49	6.97	11.05	15.23	9.50	-0.6801	0.01954079	0.028227166	ywqB
AB5991_19400	23.76	26.85	23.8	27.97	29.83	24.1	24.80	27.30	0.1384	0.592458015	0.649007111	ywqA
AB5991_19405	4.42	6.58	2.93	58.88	53.82	44.7	4.64	52.47	3.4982	7.02E-24	6.19E-23	ywpJ
AB5991_19410	4.19	2.56	1.49	14.42	13.02	14.1	2.75	13.85	2.3338	7.34E-09	2.01E-08	glcR
AB5991_19415	14.27	15.53	14.69	9.2	3.77	8.01	14.83	6.99	-1.0845	0.010830575	0.016101915	ssbB
AB5991_19425	149.96	303.63	162.25	69.35	46.55	34.28	205.28	50.06	-2.0359	3.73E-08	9.61E-08	ywpF
AB5991_19435	4.44	1.81	4.23	0	0	0.53	3.49	0.18	-4.3055	0.000256	0.000461241	--
AB5991_19440	52.69	74.15	53.35	2.58	3.3	1.17	60.06	2.35	-4.6758	6.04E-34	1.08E-32	ywpD
AB5991_19445	1.83	2.51	0.49	5.96	6.97	1.48	1.61	4.80	1.5770	0.031920699	0.044538685	mscL
AB5991_19450	1.27	1.56	1.81	3.23	2.16	0.46	1.55	1.95	0.3343	0.858230481	0.899454536	fabZ
AB5991_19455	47.18	52.04	51.73	31.75	20.04	12.86	50.32	21.55	-1.2233	0.000218404	0.000395839	rapD
AB5991_19460	24.55	27.86	25.77	20.39	10.22	8.45	26.06	13.02	-1.0011	0.004869758	0.007589407	flhP
AB5991_19465	30.68	28.58	28.53	18.62	9.05	5.05	29.26	10.91	-1.4239	0.000405486	0.000718192	flhO
AB5991_19470	20.56	41.4	31.64	107.71	77.84	36.52	31.20	74.02	1.2464	0.001543358	0.002563226	mbl
AB5991_19475	12.82	40.02	25.36	0.7	4.57	1.39	26.07	2.22	-3.5536	5.83E-08	1.48E-07	spoIIID
AB5991_19480	0	0	1.74	0	0	0	0.58	0.00	-9.1799	1	1	usd
AB5991_19485	38.42	26.46	37.82	14.72	15.11	9.92	34.23	13.25	-1.3694	3.76E-05	7.40E-05	ywoH
AB5991_19490	37.49	39.85	50.47	17.5	12.97	10.35	42.60	13.61	-1.6467	3.43E-08	8.88E-08	ywoG
AB5991_19495	27.75	28.07	29.67	25.57	18.83	12.1	28.50	18.83	-0.5975	0.051272006	0.069252087	ywoF
AB5991_19500	3.93	6.76	3.67	0.4	0.37	0.66	4.79	0.48	-3.3280	6.73E-10	2.00E-09	pucI

AB5991_19505	4.92	8.55	9.39	5.64	10.02	10.8	7.62	8.82	0.2110	0.569433347	0.626203218	ywoD
AB5991_19510	17.12	18.05	20.35	8.97	10.97	16.82	18.51	12.25	-0.5949	0.077602561	0.102719923	ywoC
AB5991_19515	0	1.43	1.66	2.54	4.35	5.89	1.03	4.26	2.0482	0.004382563	0.006873285	ywoB
AB5991_19520	84.2	59.82	78.59	7.28	1.82	1.45	74.20	3.52	-4.3992	5.14E-19	3.16E-18	nrgA
AB5991_19525	107.11	94.56	88.11	112.01	97.48	114.83	96.59	108.11	0.1625	0.538639998	0.595804856	nrgB
AB5991_19530	343.49	1102.88	323.15	5.74	5.69	13.78	589.84	8.40	-6.1332	3.25E-26	3.44E-25	bcrC
AB5991_19535	11.96	23.54	15.54	9.76	4.35	1.85	17.01	5.32	-1.6772	0.001471583	0.002452227	ywnJ
AB5991_19540	677.03	684.69	607.34	82.64	66.59	56.03	656.35	68.42	-3.2620	2.28E-29	2.97E-28	spoIIQ
AB5991_19545	16.53	22.26	20.43	39.96	35.89	19.88	19.74	31.91	0.6929	0.044444736	0.06058704	ywnH
AB5991_19550	11.84	19.18	16.76	26.51	19.49	26.01	15.93	24.00	0.5918	0.06662291	0.089017353	ywnG
AB5991_19555	1472.1	1738.37	1570.25	45.53	84.59	117.68	1593.57	82.60	-4.2700	1.32E-31	2.00E-30	pseI
AB5991_19560	230.68	211.41	207.86	20.65	29.4	28.14	216.65	26.06	-3.0553	3.96E-24	3.56E-23	ywnF
AB5991_19565	505.57	474.82	492.24	23.61	24.25	29.3	490.88	25.72	-4.2544	2.80E-48	1.42E-46	clsA
AB5991_19570	20.55	18.01	14.48	40.89	52.53	46.01	17.68	46.48	1.3944	1.14E-06	2.59E-06	mta
AB5991_19575	5.65	10.37	10.57	0	0.48	0.51	8.86	0.33	-4.7473	1.50E-09	4.33E-09	ywnC
AB5991_19580	2.25	0	1.51	8.27	11.75	4.88	1.25	8.30	2.7273	8.68E-06	1.81E-05	ywnB
AB5991_19585	4.95	0	0.96	34.23	28.37	28.72	1.97	30.44	3.9497	2.24E-14	9.60E-14	ywnA
AB5991_19590	25.16	32.8	20.8	289.58	306.28	384.46	26.25	326.77	3.6377	1.33E-31	2.01E-30	ureC
AB5991_19595	4.82	9.74	6.7	85.97	106.45	97.05	7.09	96.49	3.7672	2.82E-26	3.01E-25	ureB
AB5991_19600	1.71	3.13	3.65	50.69	59.01	61.53	2.83	57.08	4.3340	2.13E-24	1.97E-23	ureA
AB5991_19605	0	0	0	0	0	0	0.00	0.00	0.0000	1	1	--
AB5991_19615	1.91	0	3.07	10.4	8.76	7.25	1.66	8.80	2.4069	0.002479146	0.004034708	csbD
AB5991_19620	1.51	1.38	0.4	1.64	0.38	0.82	1.10	0.95	-0.2122	1	1	ywmF
AB5991_19625	87.66	87.22	76.19	2.95	2.27	2.24	83.69	2.49	-5.0728	7.19E-47	3.14E-45	rapB
AB5991_19630	12.68	23.29	16.01	91.2	75.3	72.47	17.33	79.66	2.2008	2.24E-12	8.30E-12	moaA
AB5991_19635	9.16	19.77	10.78	21.43	28.44	24.3	13.24	24.72	0.9013	0.008779599	0.01322602	fdhD

AB5991_19640	1.12	6.15	7.16	67.95	70.4	61.6	4.81	66.65	3.7925	4.19E-16	2.04E-15	ywmE
AB5991_19645	7.77	11.31	7.45	41.06	73.58	58.26	8.84	57.63	2.7042	6.87E-15	3.07E-14	ywmD
AB5991_19650	0	0	0	0	0	0	0.00	0.00	0.0000	1	1	--
AB5991_19655	25.1	30.57	21.76	149.45	196.6	135.59	25.81	160.55	2.6370	1.40E-18	8.33E-18	ywmC
AB5991_19660	602.32	728.1	501.03	16.95	16.76	14.03	610.48	15.91	-5.2616	5.17E-58	4.67E-56	spoIID
AB5991_19665	3593.89	3923.7	3387.14	247.41	247.38	314.76	3634.91	269.85	-3.7517	3.62E-42	1.13E-40	murAA
AB5991_19670	55.86	61.81	53.74	16.71	11.42	13.47	57.14	13.87	-2.0428	1.35E-12	5.08E-12	ywmB
AB5991_19675	1.56	1.44	4.18	2.55	0.8	3.39	2.39	2.25	-0.0912	1	1	ywzB
AB5991_19680	176.55	220.56	183.51	5.81	5.82	7.43	193.54	6.35	-4.9290	4.32E-44	1.53E-42	ywmA
AB5991_19685	89.24	126.43	69.28	906.05	997.71	950.36	94.98	951.37	3.3243	9.74E-27	1.07E-25	atpC
AB5991_19690	106.01	122.77	93.38	818.53	996.64	992.75	107.39	935.97	3.1237	1.54E-28	1.90E-27	atpD
AB5991_19695	25.73	24.2	32.22	279.85	254.01	230.08	27.38	254.65	3.2171	4.20E-29	5.35E-28	atpG
AB5991_19700	33.06	34.31	41.12	360.72	354.51	297.97	36.16	337.73	3.2233	1.09E-29	1.47E-28	atpA
AB5991_19705	8.28	13.37	16.99	184.7	181.6	175.24	12.88	180.51	3.8089	1.44E-29	1.92E-28	atpH
AB5991_19710	16.56	16.82	32.78	210.76	290.1	219.69	22.05	240.18	3.4451	2.39E-23	2.03E-22	atpF
AB5991_19715	11.88	7.79	17.24	48.91	34.55	29.4	12.30	37.62	1.6125	0.000131604	0.000243636	atpE
AB5991_19720	20.66	23.03	37.87	86.39	89.85	46.32	27.19	74.19	1.4483	2.77E-05	5.52E-05	atpB
AB5991_19725	8.94	16.42	11.07	29.69	20.6	13.76	12.14	21.35	0.8141	0.04649298	0.063183992	atpI
AB5991_19730	8.89	12.12	11.97	37.76	28.32	17.39	10.99	27.82	1.3397	0.000209827	0.000380989	upp
AB5991_19735	103.41	110.36	95.56	226.98	187.79	132.79	103.11	182.52	0.8239	0.003173286	0.005077002	glyA
AB5991_19740	25.63	34.84	30.97	39.82	32.18	30.99	30.48	34.33	0.1716	0.563381243	0.620062893	ywlG
AB5991_19745	28.52	32.45	31.36	33.2	39.24	28.26	30.78	33.57	0.1252	0.669030217	0.723375836	ywlF
AB5991_19750	13.17	17.58	14.51	9.11	6.09	1.73	15.09	5.64	-1.4187	0.002739572	0.004433106	ywlE
AB5991_19755	5.83	16.65	5.89	0	0.66	0.35	9.46	0.34	-4.8119	2.77E-09	7.82E-09	mntP
AB5991_19760	8.16	4.46	7.61	4.15	4.24	1.32	6.74	3.24	-1.0590	0.01745996	0.025424826	ywlC
AB5991_19765	82.64	137.54	76.19	47.82	31.07	40.54	98.79	39.81	-1.3112	4.32E-05	8.43E-05	ywlB

AB5991_19770	117.55	162.75	99.08	67.57	52.6	59.42	126.46	59.86	-1.0789	0.000215104	0.000390214	spoIIR
AB5991_19775	6.97	7.31	7.99	2.17	4.05	1.08	7.42	2.43	-1.6091	0.003058034	0.004910413	ywlA
AB5991_19780	122.38	84.12	115.44	14.33	45.35	44.84	107.31	34.84	-1.6230	2.96E-05	5.88E-05	ywkF
AB5991_19785	5.21	5.74	5.35	5.9	8.27	2.71	5.43	5.63	0.0504	0.960840816	0.995953767	prmC
AB5991_19790	70.54	80.88	55.05	69.01	57.03	48.6	68.82	58.21	-0.2415	0.390078939	0.447430233	prfA
AB5991_19795	1.87	2.57	0	1.02	1.43	0	1.48	0.82	-0.8578	0.641492303	0.696763112	ywkD
AB5991_19800	300.92	559.13	296.38	386.43	289.36	309.53	385.48	328.44	-0.2310	0.456840604	0.515666299	racA
AB5991_19805	2.82	4.49	1.81	1.02	2.49	3.26	3.04	2.26	-0.4299	0.486806563	0.546539119	ywkB
AB5991_19810	5.43	5.08	4.89	10.77	12.46	12.79	5.13	12.01	1.2259	3.71E-05	7.29E-05	maeA
AB5991_19815	7.07	5.64	5.59	15.38	6.88	6.99	6.10	9.75	0.6766	0.130031922	0.166512984	tdk
AB5991_19820	128.59	123.84	169.25	347.2	273.65	207.34	140.56	276.06	0.9738	0.001122514	0.001907362	rpmE
AB5991_19825	132.61	130.79	126.3	95.69	84.53	51.05	129.90	77.09	-0.7528	0.008887482	0.013383463	rho
AB5991_19830	12.27	18.44	15.51	2.43	2.27	1.21	15.41	1.97	-2.9673	9.65E-06	2.00E-05	--
AB5991_19835	38.17	53.6	40.82	160.15	147.21	123.96	44.20	143.77	1.7018	5.45E-10	1.63E-09	glpX
AB5991_19840	22.56	29.84	24.12	58.36	57.9	32.31	25.51	49.52	0.9572	0.00225086	0.003676744	murAB
AB5991_19845	0	0	0	0	0	4.25	0.00	1.42	10.4683	0.274013903	0.325347926	--
AB5991_19850	111.16	130.37	103.45	747.87	751.67	587.9	114.99	695.81	2.5972	2.11E-20	1.47E-19	tal
AB5991_19855	36.66	51.06	38.52	291.66	275.51	173.31	42.08	246.83	2.5523	1.26E-16	6.44E-16	fbaA
AB5991_19860	4.82	13.28	4.64	20.44	21.58	15.65	7.58	19.22	1.3426	0.001642618	0.002717848	spo0F
AB5991_19865	14.89	12.72	10	10.17	8.46	8.62	12.54	9.08	-0.4649	0.196342619	0.241610332	ywjG
AB5991_19870	16.3	18.78	16.11	110.15	70.93	50.38	17.06	77.15	2.1768	3.72E-11	1.24E-10	pyrG
AB5991_19875	171.84	229.77	174.99	38	29.39	15.05	192.20	27.48	-2.8062	3.52E-15	1.61E-14	rpoE
AB5991_19880	72.78	94.33	63.24	81.39	72.45	73.8	76.78	75.88	-0.0171	0.957734172	0.992992793	acdA
AB5991_19885	63.32	103.11	60.32	133.19	128.72	135.06	75.58	132.32	0.8079	0.004816445	0.007512216	fadF
AB5991_19890	5.29	4.16	8.07	1.48	0.61	0.98	5.84	1.02	-2.5127	1.36E-07	3.32E-07	clsB
AB5991_19895	3.75	7.24	7.63	1.02	0.96	0.41	6.21	0.80	-2.9618	8.80E-08	2.19E-07	uvsE

AB5991_19900	0	10.94	2.83	15.12	6.74	32.97	4.59	18.28	1.9934	0.013794755	0.020326149	ywjC
AB5991_19905	18.94	17.7	15.46	61.41	50.11	36.15	17.37	49.22	1.5030	2.28E-06	5.03E-06	ywjB
AB5991_19915	5.41	20.32	5.53	81.35	69.64	123.41	10.42	91.47	3.1339	3.48E-11	1.16E-10	ywiE
AB5991_19925	9.68	8.4	8.34	2.93	0.82	1.16	8.81	1.64	-2.4278	2.33E-07	5.63E-07	narI
AB5991_19930	3.58	4.19	4.18	0.71	0.33	0.35	3.98	0.46	-3.1039	1.09E-05	2.25E-05	narJ
AB5991_19935	6.05	10.65	6.34	0.94	0.5	0.13	7.68	0.52	-3.8753	4.84E-14	2.02E-13	narH
AB5991_19940	4.12	5.94	4.19	0.91	0.8	0.64	4.75	0.78	-2.6002	3.76E-13	1.48E-12	narG
AB5991_19945	0.38	2.78	0.41	0.82	0	0.41	1.19	0.41	-1.5373	0.415121205	0.472740552	arfM
AB5991_19950	0.5	0.92	0	1.64	0.77	1.63	0.47	1.35	1.5085	0.106258908	0.138147302	ywiC
AB5991_19955	11.34	12.96	18.87	7.4	3.08	1.09	14.39	3.86	-1.8996	0.000219667	0.000397946	fnr
AB5991_19960	3.96	3.63	5.86	1.32	0.77	0.49	4.48	0.86	-2.3822	1.04E-06	2.38E-06	narK
AB5991_19965	528.51	362.28	490.46	115.29	90.05	64.75	460.42	90.03	-2.3545	1.42E-14	6.22E-14	argS
AB5991_19970	16.43	19.34	13.07	5.96	5.15	2.28	16.28	4.46	-1.8669	1.32E-05	2.70E-05	ywiB
AB5991_19975	39.71	60.34	27.82	168.29	147.72	112.65	42.62	142.89	1.7452	6.64E-07	1.54E-06	sboA
AB5991_19980	15.36	8.68	6.32	16.7	13.23	12.79	10.12	14.24	0.4927	0.448204098	0.506937924	sboX
AB5991_19985	387.8	360.72	281.26	791.29	792.24	789.76	343.26	791.10	1.2046	4.36E-06	9.35E-06	albA
AB5991_19990	11.16	12.29	15.51	31.55	31.8	27.78	12.99	30.38	1.2259	0.003180611	0.005086671	albB
AB5991_19995	61.76	68.68	65.77	152.62	191.88	151.91	65.40	165.47	1.3391	3.29E-07	7.86E-07	albC
AB5991_20000	24.82	18.48	26.24	58.33	62.72	49.55	23.18	56.87	1.2947	5.19E-06	1.11E-05	albD
AB5991_20005	24.44	17.72	18.31	51.81	49.75	53.25	20.16	51.60	1.3562	9.72E-07	2.22E-06	albE
AB5991_20010	34.15	24.1	30.33	96.53	121.2	133.04	29.53	116.92	1.9855	1.52E-12	5.69E-12	albF
AB5991_20015	43	61.46	90.86	62.45	73.37	83.06	65.11	72.96	0.1643	0.612569238	0.66843894	albG
AB5991_20020	47368.23	39208.11	44670.94	494.24	666.5	892.62	43749.09	684.45	-5.9982	1.86E-71	6.71E-69	ywhL
AB5991_20025	46424.54	36990.09	42387.07	456.22	649.15	917.56	41933.90	674.31	-5.9586	2.90E-64	5.23E-62	ywhK
AB5991_20035	160.88	191.43	151.13	114.93	137.89	95.1	167.81	115.97	-0.5331	0.046318931	0.062969009	rapF
AB5991_20040	27.11	16.59	33.83	36.04	29.13	13.04	25.84	26.07	0.0126	1	1	phrF

AB5991_20050	7.04	7.22	7.09	31.3	21.91	10.98	7.12	21.40	1.5881	6.74E-05	0.000128436	speB
AB5991_20055	11.09	12.78	10.7	30.52	20.14	5.65	11.52	18.77	0.7039	0.140194799	0.177523452	speE
AB5991_20060	65.3	91.93	79.89	7.29	5.14	6.13	79.04	6.19	-3.6753	7.84E-33	1.28E-31	pbpG
AB5991_20065	2.06	3.79	4.41	0.9	2.1	0.89	3.42	1.30	-1.3992	0.056120261	0.07551798	--
AB5991_20070	117.37	134.93	112.85	24.62	29.42	21.49	121.72	25.18	-2.2734	6.97E-16	3.34E-15	ywhD
AB5991_20075	49.3	34.7	49.2	14.3	12.26	7.11	44.40	11.22	-1.9841	3.63E-09	1.02E-08	ywhC
AB5991_20080	0	3.51	1.02	2.08	0	2.07	1.51	1.38	-0.1264	1	1	ywhB
AB5991_20085	35.29	37.93	46.02	8.89	11.83	2.8	39.75	7.84	-2.3419	1.13E-08	3.03E-08	ywhA
AB5991_20090	21.97	21.47	19.46	2.77	4.13	4.59	20.97	3.83	-2.4527	7.02E-16	3.35E-15	thrZ
AB5991_20095	2.45	7.34	4.28	7.86	6.83	7.26	4.69	7.32	0.6416	0.092935673	0.121797874	mmr
AB5991_20100	0.38	0	3.28	2.5	2.73	1.66	1.22	2.30	0.9127	0.378516798	0.435985751	ywgB
AB5991_20105	1.08	3.97	2.7	27.07	35.62	19.14	2.58	27.28	3.4004	3.24E-13	1.28E-12	ywgA
AB5991_20110	2.64	4.33	3.86	31.1	26	17.43	3.61	24.84	2.7828	1.08E-14	4.73E-14	ywfO
AB5991_20115	8.84	14.75	23.2	3.49	6.54	0.87	15.60	3.63	-2.1019	0.001243121	0.002099717	ywzC
AB5991_20120	55.13	111.48	68.91	16.95	7.34	9.07	78.51	11.12	-2.8197	4.19E-13	1.64E-12	rsfA
AB5991_20125	6.9	8.57	6.29	18.09	12.39	13.83	7.25	14.77	1.0260	0.00151346	0.002515676	ywfM
AB5991_20130	2.56	1.18	4.34	7.9	5.65	5.55	2.69	6.37	1.2411	0.0083895	0.012676828	lipL
AB5991_20135	1.61	1.11	1.72	1.97	3.68	1.96	1.48	2.54	0.7773	0.191445138	0.236095852	cysL
AB5991_20140	8.37	16.05	13.52	47.73	48.83	25.56	12.65	40.71	1.6865	1.70E-06	3.79E-06	pta
AB5991_20145	39.93	68.11	56.85	165.74	136.59	95.15	54.96	132.49	1.2694	4.68E-05	9.09E-05	chdC
AB5991_20150	15.76	36.59	26.27	66.54	49.29	38.38	26.21	51.40	0.9719	0.004381546	0.006873285	bacG
AB5991_20155	42.4	46.44	40.41	41.42	46.48	40.58	43.08	42.83	-0.0086	0.982523458	1	bacF
AB5991_20160	7.47	10.92	9.13	27.7	29.65	21.3	9.17	26.22	1.5150	4.31E-07	1.02E-06	bacE
AB5991_20165	27.9	32.74	32.42	60.82	62.23	40.95	31.02	54.67	0.8175	0.003841578	0.006067982	bacD
AB5991_20170	9.73	7.84	6.34	58.56	47.08	47.25	7.97	50.96	2.6768	2.26E-17	1.22E-16	bacC
AB5991_20175	25.02	31.41	25.94	66.08	41.83	24.32	27.46	44.08	0.6829	0.056032006	0.075424778	bacB

AB5991_20180	17.93	18.89	19.8	15.02	13.76	5.73	18.87	11.50	-0.7143	0.056334075	0.075780018	bacA
AB5991_20185	24.8	35.36	28.39	27.77	20.04	16.74	29.52	21.52	-0.4561	0.122687824	0.15766775	ywfA
AB5991_20190	28.91	17.15	21.48	74.85	111.31	138.61	22.51	108.26	2.2656	2.44E-12	8.98E-12	rocC
AB5991_20195	16.05	8.78	12.5	59.63	98.95	113.19	12.44	90.59	2.8640	7.24E-17	3.78E-16	rocB
AB5991_20200	32.34	12.65	16.86	140.71	210.22	237.88	20.62	196.27	3.2510	1.25E-17	6.92E-17	rocA
AB5991_20205	0.99	0.52	1.36	11.1	18.04	21.33	0.96	16.82	4.1363	8.26E-20	5.50E-19	rocG
AB5991_20210	1468.15	1640.18	1657.75	77.36	80.7	60.59	1588.69	72.88	-4.4461	7.77E-50	4.35E-48	bslB
AB5991_20215	8528.51	8525.79	6944.59	196.58	282.39	377.16	7999.63	285.38	-4.8090	1.37E-47	6.38E-46	spsL
AB5991_20220	8766.55	8592.53	7447.94	239.96	338.55	465.96	8269.01	348.16	-4.5699	2.46E-44	8.88E-43	spsK
AB5991_20225	7462	7203.21	6459.23	259.2	338.03	434.25	7041.48	343.83	-4.3561	2.84E-46	1.17E-44	rfbB
AB5991_20230	3972.09	3779.34	3595.63	153.34	211.52	286.23	3782.35	217.03	-4.1233	1.80E-39	4.85E-38	rmlA
AB5991_20235	6637.39	6622.19	5735.9	192.34	273.23	395.54	6331.83	287.04	-4.4633	7.83E-41	2.30E-39	spsG
AB5991_20240	6260.47	5765.16	5654.44	225.4	310.41	419.47	5893.36	318.43	-4.2101	1.02E-40	2.96E-39	spsF
AB5991_20245	5683.93	5525.29	4965	234.95	312.01	458.98	5391.41	335.31	-4.0071	5.35E-36	1.15E-34	spsE
AB5991_20250	4805.23	4249.72	4309.34	169.24	206.37	307.66	4454.76	227.76	-4.2898	8.14E-42	2.45E-40	spsD
AB5991_20255	3798.82	3294.79	3175.47	127.02	154.56	224.93	3423.03	168.84	-4.3416	4.47E-43	1.48E-41	spsC
AB5991_20260	3630.58	3132.11	3347.71	91.46	115.67	154.06	3370.13	120.40	-4.8069	1.20E-53	9.03E-52	spsB
AB5991_20265	5026.51	3739.47	4528.24	105.05	123.12	160.13	4431.41	129.43	-5.0975	3.78E-59	3.95E-57	spsA
AB5991_20270	935.86	1612.04	981.34	57.61	55.93	50.17	1176.41	54.57	-4.4301	2.93E-40	8.31E-39	gerQ
AB5991_20275	5.34	4.46	3.12	11.1	10.38	4.73	4.31	8.74	1.0205	0.04656435	0.0632377	ywdK
AB5991_20280	5.6	4.52	7.16	12.48	14.88	14.05	5.76	13.80	1.2609	5.84E-05	0.000111992	ywdJ
AB5991_20285	3.41	5.22	2.43	13.6	20.83	9.84	3.69	14.76	2.0010	0.000107385	0.000200671	ywdI
AB5991_20290	3.43	7.02	3.38	1.86	1.61	1.14	4.61	1.54	-1.5850	0.000822654	0.001411127	ywdH
AB5991_20295	2.4	1.96	2.28	13.92	12.48	10.68	2.21	12.36	2.4814	1.88E-09	5.40E-09	ung
AB5991_20300	0	1.23	0.24	7.31	6.61	3.39	0.49	5.77	3.5577	9.05E-08	2.25E-07	ywdF
AB5991_20305	73.32	99.65	76.51	13.97	16.46	12.23	83.16	14.22	-2.5480	1.50E-17	8.21E-17	pdxK

AB5991_20310	9.44	14.66	13.2	3.95	7.39	1.57	12.43	4.30	-1.5307	0.00514585	0.00800085	ywdA
AB5991_20315	3.77	4.61	2.82	9.15	8.81	8.7	3.73	8.89	1.2512	0.00010399	0.000194545	sacA
AB5991_20320	0.65	1.2	1.12	3.12	2.92	2.54	0.99	2.86	1.5305	0.000876695	0.001499291	sacP
AB5991_20325	4.69	3.01	5.77	0	0	0.25	4.49	0.08	-5.7517	4.70E-10	1.42E-09	ywcJ
AB5991_20335	54.88	106.25	38.91	67.47	75.89	63.28	66.68	68.88	0.0468	0.890905732	0.92928465	ywcI
AB5991_20340	115.12	135.31	75.29	393.57	500.36	506.49	108.57	466.81	2.1042	1.97E-12	7.32E-12	vpr
AB5991_20345	20.74	27.82	15.24	32.76	40.02	36.52	21.27	36.43	0.7767	0.006866598	0.010507614	ywcH
AB5991_20350	95.68	160.19	92.01	271.28	363.75	323.75	115.96	319.59	1.4626	9.34E-07	2.14E-06	nfrA1
AB5991_20355	39.3	69.92	41.21	6.82	7	6.62	50.14	6.81	-2.8796	6.57E-18	3.72E-17	rodA
AB5991_20360	41.8	44.6	38.96	52.66	51.21	45.59	41.79	49.82	0.2537	0.337271795	0.393450734	--
AB5991_20365	493.64	577.04	664.81	1880.92	1896.73	1644.59	578.50	1807.41	1.6435	8.97E-10	2.63E-09	ywcE
AB5991_20370	53.98	28.32	50.51	68.15	64.26	56.35	44.27	62.92	0.5072	0.109731841	0.142493505	qoxD
AB5991_20375	45.26	26.44	40.86	78.31	64.91	67.77	37.52	70.33	0.9065	0.001821003	0.002999255	qoxC
AB5991_20380	37.26	20.59	49.56	52.52	41.98	33.71	35.80	42.74	0.2554	0.448831583	0.507301347	qoxB
AB5991_20385	34.99	17.52	36.22	48.43	42.66	39.09	29.58	43.39	0.5530	0.079572478	0.105187187	qoxA
AB5991_20390	181.48	228.01	195.65	357.2	342.49	349.17	201.71	349.62	0.7935	0.00211748	0.003465998	ywzA
AB5991_20395	53.45	63.71	47.88	190.21	229.77	240.58	55.01	220.19	2.0009	2.56E-13	1.02E-12	galT
AB5991_20400	6.47	9.34	6.1	28.83	34.66	34.36	7.30	32.62	2.1590	1.02E-12	3.87E-12	galK
AB5991_20405	0	0	1.01	1.02	1.44	1.02	0.34	1.16	1.7847	0.319910675	0.374960239	ywcD
AB5991_20410	1.34	0.49	0.86	2.05	1.09	2.33	0.90	1.82	1.0239	0.181980476	0.225263239	ywcC
AB5991_20415	18.19	18.79	23.1	43.27	15.04	8.61	20.03	22.31	0.1556	0.830403676	0.873056129	slrA
AB5991_20420	79.55	106.33	75.07	7	5.36	3.8	86.98	5.39	-4.0133	1.37E-24	1.29E-23	ywcB
AB5991_20425	203.61	226.64	180.13	80.83	81.6	82.86	203.46	81.76	-1.3152	3.94E-07	9.34E-07	ywcA
AB5991_20430	5.1	4.4	2.88	3.91	0.92	0	4.13	1.61	-1.3579	0.077601188	0.102719923	ywbO
AB5991_20435	32.36	65.79	35.07	19.33	21.03	16.89	44.41	19.08	-1.2185	0.000212957	0.000386496	efeN
AB5991_20440	12.33	16.34	13.02	6.79	6.2	5.74	13.90	6.24	-1.1544	0.000148409	0.000273219	efeM

AB5991_20445	11.9	23.23	12.86	2.72	2.04	2.85	16.00	2.54	-2.6568	8.05E-12	2.83E-11	efeU
AB5991_20450	16.21	23.32	19.36	86.68	102.57	98.56	19.63	95.94	2.2890	3.34E-15	1.53E-14	thiE
AB5991_20455	13.02	7.7	10.38	38.16	41.1	48.26	10.37	42.51	2.0357	3.79E-11	1.26E-10	thiM
AB5991_20460	4.39	5.86	3.84	30.38	37.36	31.1	4.70	32.95	2.8104	3.42E-17	1.82E-16	ywbI
AB5991_20465	8.41	17.15	13.99	9.14	5.23	11.12	13.18	8.50	-0.6337	0.170252566	0.211338837	cidA
AB5991_20470	31.72	20.07	21.67	11.89	10.31	10.68	24.49	10.96	-1.1597	0.000244506	0.000441735	ywbG
AB5991_20475	1.95	0.55	0.96	1.63	3.36	2.93	1.15	2.64	1.1947	0.037809017	0.052149916	ywbF
AB5991_20480	0.91	5.03	1.95	0	0	0	2.63	0.00	-11.3608	0.02339298	0.033390915	ywbE
AB5991_20485	239.02	291.38	293.36	19.86	19.51	15.15	274.59	18.17	-3.9174	2.42E-39	6.40E-38	ywbD
AB5991_20490	34.16	66.2	51.75	13.93	15.45	8.73	50.70	12.70	-1.9969	8.03E-08	2.01E-07	ywbC
AB5991_20495	10.13	18.11	13.97	4.64	2.98	2.31	14.07	3.31	-2.0877	7.71E-07	1.78E-06	ywbB
AB5991_20510	8.12	5.77	9.38	1.99	1.6	1.56	7.76	1.72	-2.1758	9.79E-09	2.65E-08	sacX
AB5991_20515	17.15	16.14	13.53	1.4	2.62	1.62	15.61	1.88	-3.0534	7.94E-15	3.53E-14	sacY
AB5991_20520	3673.34	5304.46	3652.83	1916.26	2511.54	2399.75	4210.21	2275.85	-0.8875	0.000878607	0.001501269	gspA
AB5991_20525	0.76	0.46	1.62	0	0.26	0.55	0.95	0.27	-1.8099	0.130709326	0.167242903	ywaF
AB5991_20530	2299.65	2285.28	2839.1	34.29	31.37	31.47	2474.68	32.38	-6.2561	3.46E-81	3.44E-78	ywaE
AB5991_20535	17.03	13.09	14.94	2.85	3.41	1.89	15.02	2.72	-2.4670	7.65E-13	2.94E-12	tyrS2
AB5991_20540	93.02	151.39	74.04	452.51	715.13	632.61	106.15	600.08	2.4991	5.95E-14	2.46E-13	ywaD
AB5991_20545	15.99	15.73	10.08	72.67	67.13	74.49	13.93	71.43	2.3580	1.90E-15	8.81E-15	ywaC
AB5991_20550	2.43	1.67	2.92	4.79	7.57	5.42	2.34	5.93	1.3407	0.000856609	0.00146747	HI_1721
AB5991_20555	56.77	65.6	53.49	19.32	16.51	13.17	58.62	16.33	-1.8436	8.36E-11	2.68E-10	menA
AB5991_20560	0	0	0	0	0	0	0.00	0.00	0.0000	1	1	--
AB5991_20565	32.53	33.19	19.33	0	1.23	0	28.35	0.41	-6.1116	7.33E-13	2.83E-12	ywzH
AB5991_20570	341.42	557.54	345.3	30.55	18.25	18.51	414.75	22.44	-4.2083	3.29E-32	5.10E-31	dltA
AB5991_20575	202.35	236.63	206.15	23	15.18	9.72	215.04	15.97	-3.7515	1.19E-27	1.40E-26	dltB
AB5991_20580	151.77	168.05	118.26	9.95	10.09	16.51	146.03	12.18	-3.5833	9.25E-24	8.07E-23	dltC

AB5991_20585	281.47	328.79	264.61	21.68	18.57	14.77	291.62	18.34	-3.9910	1.82E-39	4.89E-38	dltD
AB5991_20595	1898.53	3560.22	2346.96	61.75	54.58	56.26	2601.90	57.53	-5.4991	3.65E-58	3.37E-56	ilvK
AB5991_20600	36.04	46.7	32.43	43.93	54.54	53	38.39	50.49	0.3953	0.15316939	0.19211486	licH
AB5991_20605	11.94	21.93	11.61	12.4	21.54	15.28	15.16	16.41	0.1140	0.77225878	0.819957117	licA
AB5991_20610	26.07	36.39	26.17	25.46	26.8	30.23	29.54	27.50	-0.1036	0.727200102	0.777311333	licC
AB5991_20615	14.04	8.59	9.38	13.36	14.29	20.9	10.67	16.18	0.6009	0.155371329	0.194630773	licB
AB5991_20620	17.83	17.92	15.46	25.72	18.82	29.77	17.07	24.77	0.5371	0.057958495	0.077780731	licR
AB5991_20625	18.19	45.92	34.04	56.87	28.92	39.38	32.72	41.72	0.3508	0.393646207	0.45100089	yxzF
AB5991_20630	69.12	94.91	58.54	4.99	6.23	6.29	74.19	5.84	-3.6680	3.63E-25	3.54E-24	yxlJ
AB5991_20635	84.33	132.03	96.76	276.7	341.96	300.87	104.37	306.51	1.5542	2.31E-08	6.05E-08	katX
AB5991_20640	26.89	20.42	36.79	35.95	38.99	47.33	28.03	40.76	0.5399	0.072929898	0.096890139	yxlH
AB5991_20645	1057.74	899.14	991.59	40.48	52.8	52.88	982.82	48.72	-4.3343	1.22E-47	5.79E-46	yxlG
AB5991_20650	1101.8	935.87	1006.09	20.81	29.83	32.17	1014.59	27.60	-5.1999	1.17E-58	1.13E-56	yxlF
AB5991_20655	169.27	119.41	197.38	5.2	0	6.21	162.02	3.80	-5.4128	5.96E-30	8.26E-29	yxlE
AB5991_20660	416.51	333.49	487.44	7.6	14.22	3.78	412.48	8.53	-5.5951	6.08E-43	1.98E-41	yxlD
AB5991_20665	257.33	236.77	267.96	4.9	5.73	3.66	254.02	4.76	-5.7368	1.37E-52	9.55E-51	yxlC
AB5991_20670	1331.22	1176.14	1517.91	15.38	15.76	13.48	1341.76	14.87	-6.4953	7.38E-81	5.86E-78	sigY
AB5991_20675	92.61	96.86	98.76	7.3	7.5	6.12	96.08	6.97	-3.7843	2.68E-37	6.29E-36	yxlA
AB5991_20680	6.31	14.38	5.82	41.16	38.52	41.91	8.84	40.53	2.1974	4.36E-10	1.32E-09	nnrD
AB5991_20685	25.42	29.39	25.95	3.64	3.94	1.7	26.92	3.09	-3.1214	2.10E-20	1.46E-19	cydD
AB5991_20690	23.97	22.79	22.01	2.42	2.48	1.95	22.92	2.28	-3.3276	9.51E-26	9.81E-25	cydC
AB5991_20695	29.5	46.01	31.93	3.67	2.53	3.08	35.81	3.09	-3.5333	5.62E-23	4.66E-22	cydB
AB5991_20700	7.58	6.84	8.11	1.68	1.31	2.78	7.51	1.92	-1.9652	1.43E-07	3.48E-07	cydA
AB5991_20705	5.61	7.11	7.57	7.85	7.21	9.11	6.76	8.06	0.2524	0.428505384	0.486169965	cimH
AB5991_20710	1.71	2.16	2.63	7.57	7.41	3.24	2.17	6.07	1.4870	0.000437905	0.00077276	yxkI
AB5991_20715	11.69	16.51	18.27	4.89	9.15	3.89	15.49	5.98	-1.3739	0.008378448	0.012664947	yxzE

AB5991_20720	1.94	11.46	2.99	0.47	1.09	0.93	5.46	0.83	-2.7186	0.000572019	0.000998895	yxkH
AB5991_20725	46.26	92.49	39.61	112.43	108.73	92.84	59.45	104.67	0.8160	0.013040878	0.019274505	msmX
AB5991_20730	1.62	0	0.65	8.14	3.5	3.72	0.76	5.12	2.7584	4.13E-05	8.07E-05	yxkF
AB5991_20735	15.25	15.02	13.12	23.73	24.1	25.36	14.46	24.40	0.7543	0.003166413	0.005068047	aldY
AB5991_20740	9.5	5.55	9.47	0.47	3.3	5.38	8.17	3.05	-1.4221	0.009808777	0.014692815	yxkD
AB5991_20745	203.79	196.53	185.9	161.89	174.04	143.68	195.41	159.87	-0.2896	0.250310331	0.301298068	galE
AB5991_20750	1.53	1.4	0.82	0.41	0.39	0	1.25	0.27	-2.2288	0.134330428	0.171519656	yxjN
AB5991_20755	1.03	1.63	2.05	3.37	1.5	1.6	1.57	2.16	0.4580	0.435670332	0.493453191	yxjM
AB5991_20760	1.1	0	0.29	2.39	2.24	0.6	0.46	1.74	1.9117	0.055440595	0.074730008	yxjL
AB5991_20765	35.48	60.03	42.95	488.98	617.67	580.95	46.15	562.53	3.6074	2.81E-31	4.20E-30	pepT
AB5991_20770	4.2	7.72	3	4.57	2.85	0	4.97	2.47	-1.0078	0.473883506	0.533841532	--
AB5991_20775	99.94	105.42	97.78	119.49	56.99	69.06	101.05	81.85	-0.3040	0.350946996	0.407726893	yxjJ
AB5991_20780	32.16	42.76	33.2	19.7	17.3	13.6	36.04	16.87	-1.0954	0.00042269	0.000747329	yxjI
AB5991_20785	19.92	23.41	19.77	91.01	70.24	63.67	21.03	74.97	1.8337	2.65E-10	8.16E-10	yxjH
AB5991_20790	34.81	42.62	36.89	85.41	68.11	54.38	38.11	69.30	0.8628	0.002304815	0.003758693	yxjG
AB5991_20795	86.64	175.81	95.65	13.46	14.26	9.35	119.37	12.36	-3.2720	1.26E-18	7.56E-18	yxjF
AB5991_20800	86.63	163.65	82.54	12.98	8.19	9.32	110.94	10.16	-3.4483	5.64E-20	3.80E-19	scoB
AB5991_20805	35.54	76.38	41.25	2.19	4.87	3.82	51.06	3.63	-3.8154	8.03E-19	4.88E-18	scoA
AB5991_20810	17.2	33.21	19.75	1.11	2.07	2.48	23.39	1.89	-3.6318	1.24E-18	7.46E-18	yxjC
AB5991_20815	35.13	43	33.01	21.3	22.75	20.97	37.05	21.67	-0.7734	0.004516711	0.007061361	yxjB
AB5991_20820	47.08	41.14	51.32	11.69	10.48	10	46.51	10.72	-2.1169	2.75E-14	1.17E-13	nupG
AB5991_20825	39.54	43.96	39.98	53.53	56.67	51.24	41.16	53.81	0.3867	0.131118484	0.167634095	yxiT
AB5991_20830	87.58	161.95	84.36	910.62	1014.62	978.31	111.30	967.85	3.1204	3.98E-22	3.17E-21	yxiS
AB5991_20835	142.95	262.65	146.96	1945.68	2295.87	2569.51	184.19	2270.35	3.6237	3.92E-29	5.00E-28	katE
AB5991_20840	66.6	83.17	72.43	6.91	6.61	6.87	74.07	6.80	-3.4459	1.94E-29	2.54E-28	citN
AB5991_20845	117.28	231.73	102.08	2660.45	3415.98	3033.69	150.36	3036.71	4.3360	1.19E-32	1.92E-31	bglS

AB5991_20850	61.55	79.59	76.95	23.1	14.56	18.77	72.70	18.81	-1.9504	2.09E-11	7.10E-11	licT
AB5991_20855	59.72	65.79	53.93	4.04	3.78	2.01	59.81	3.28	-4.1902	1.17E-31	1.79E-30	yxiP
AB5991_20860	23.59	19.08	29.14	24.29	14.01	21.59	23.94	19.96	-0.2619	0.378263359	0.43589199	yxiO
AB5991_20865	48.83	51.17	40	17.47	11.37	8.97	46.67	12.60	-1.8886	1.79E-09	5.14E-09	dbpA
AB5991_20870	27.53	27.44	16.15	6.5	7.52	4.26	23.71	6.09	-1.9600	2.52E-08	6.57E-08	yxiM
AB5991_20880	2.39	4.4	1.28	2.17	2.03	0.86	2.69	1.69	-0.6734	0.435078485	0.492923441	yxiK
AB5991_20885	3.04	6.7	1.95	0.66	1.24	1.32	3.90	1.07	-1.8601	0.030630591	0.042889307	yxiJ
AB5991_20890	3.48	4.26	4.34	0	1.77	1.25	4.03	1.01	-2.0000	0.010839162	0.01610865	--
AB5991_20895	6.69	8.51	6.06	1.12	0.52	1.11	7.09	0.92	-2.9506	8.95E-06	1.86E-05	--
AB5991_20900	7.86	8.75	8.91	8.29	8	6.96	8.51	7.75	-0.1344	0.72847638	0.778466013	--
AB5991_20905	8.87	8.14	7.51	11.66	12.79	12.8	8.17	12.42	0.6033	0.08773812	0.11521431	yxiI
AB5991_20910	12.77	12.46	12.8	7.81	8.12	8.21	12.68	8.05	-0.6557	0.067192031	0.08974758	yxzG
AB5991_20920	0.93	2.57	0	1.52	1.9	0.51	1.17	1.31	0.1672	1	1	--
AB5991_20925	0.83	0	0	1.36	0.42	1.35	0.28	1.04	1.9150	0.215931437	0.263510675	yxiG
AB5991_20930	1.42	1.74	3.04	0.52	0	1.03	2.07	0.52	-2.0000	0.084212937	0.110915281	--
AB5991_20935	8.12	9.42	5.94	0.93	0.43	0.46	7.83	0.61	-3.6894	3.50E-08	9.05E-08	wapI
AB5991_20940	6.07	3.98	4.17	0.47	3.09	0.94	4.74	1.50	-1.6599	0.011918874	0.017660391	yxiG
AB5991_20950	0	0	0.41	0.42	0.39	0.83	0.14	0.55	2.0000	0.395135201	0.452307723	yxiF
AB5991_20955	3.58	2.39	2.44	3.19	3.31	2.47	2.80	2.99	0.0930	1	1	--
AB5991_20965	3.21	6.88	4.01	6.41	6.81	5.22	4.70	6.15	0.3871	0.345739245	0.402147201	--
AB5991_20970	14.41	8.66	12.05	10.83	10.93	8.22	11.71	9.99	-0.2283	0.497942342	0.558094564	--
AB5991_20980	0	0	0	0	0.51	0	0.00	0.17	7.4094	1	1	wapI
AB5991_20985	27.38	26.82	23.95	29.1	32.14	23.91	26.05	28.38	0.1238	0.633055411	0.689296144	wapA
AB5991_20990	4.63	4.96	4.34	8.61	3.73	2.93	4.64	5.09	0.1325	0.808367528	0.852596934	yxxF
AB5991_20995	252.66	246.69	245.51	164.61	153.3	160.42	248.29	159.44	-0.6390	0.009919896	0.014844096	bacC
AB5991_21000	20	23.58	19.8	24.98	21.86	18.71	21.13	21.85	0.0486	0.876195394	0.915383296	ido

AB5991_21005	583.09	908.06	435.02	1334.73	1197.17	1124.07	642.06	1218.66	0.9245	0.002361863	0.003848567	yxiE
AB5991_21015	9.88	7.98	6.34	5.91	6.43	6.63	8.07	6.32	-0.3513	0.256949282	0.308727867	bglP
AB5991_21020	3.2	7.83	3.42	2.32	3.8	0	4.82	2.04	-1.2395	0.150839997	0.189485438	--
AB5991_21030	5.53	6.09	5.91	0.6	1.13	2.39	5.84	1.37	-2.0891	0.001626066	0.002691583	--
AB5991_21035	5.81	5.13	5.5	3.65	3.75	2.54	5.48	3.31	-0.7259	0.031069314	0.043472955	yxiD
AB5991_21040	2.68	2.46	2.15	2.91	0.68	2.17	2.43	1.92	-0.3399	0.82208062	0.865221877	yxiC
AB5991_21045	2.47	7.25	4.75	3.76	3.52	0.53	4.82	2.60	-0.8897	0.242871363	0.293321832	yxiB
AB5991_21050	20.58	23.29	24.23	0.5	0.53	0.5	22.70	0.51	-5.4761	1.03E-50	6.08E-49	hsdR
AB5991_21055	10.66	16.17	12.89	1.51	3.3	0	13.24	1.60	-3.0458	1.30E-07	3.18E-07	nudG
AB5991_21060	1.84	3.86	3.02	1.64	1.54	0.85	2.91	1.34	-1.1135	0.009095605	0.01368649	MJ0104
AB5991_21065	27.89	30.19	25.6	1.43	1.34	1.85	27.89	1.54	-4.1789	6.50E-33	1.07E-31	abn2
AB5991_21080	55.53	110.12	46.91	86.97	131.54	146.22	70.85	121.58	0.7790	0.024721624	0.03512328	pdp
AB5991_21085	43.73	96.03	38.1	15.13	23.97	18.21	59.29	19.10	-1.6339	1.55E-05	3.16E-05	nupC
AB5991_21090	34.7	79.52	32.79	17.55	13.14	11.06	49.00	13.92	-1.8161	3.98E-06	8.57E-06	deoC
AB5991_21095	1.73	4.93	2.26	20.66	10.74	17.03	2.97	16.14	2.4408	1.50E-08	4.00E-08	deoR
AB5991_21100	11.35	10.02	7.47	61.97	61.99	51.99	9.61	58.65	2.6090	1.56E-18	9.24E-18	yxxB
AB5991_21105	21.11	26.84	16.32	25.61	30.74	26.9	21.42	27.75	0.3733	0.179505076	0.222407069	yxeR
AB5991_21110	10.67	9.43	6.07	33.35	30.93	37.14	8.72	33.81	1.9544	3.95E-11	1.31E-10	yxeQ
AB5991_21115	16.29	10.74	10.15	47.81	43.94	45.36	12.39	45.70	1.8827	1.32E-10	4.17E-10	scmP
AB5991_21120	12.05	10.62	8.25	17.56	16.43	14.09	10.31	16.03	0.6369	0.046300989	0.062966174	yxeO
AB5991_21125	8.03	12.78	11.74	23.3	19.08	17.1	10.85	19.83	0.8697	0.007566327	0.011503018	yxeN
AB5991_21130	9.55	11.27	7.05	28.44	26.15	24.12	9.29	26.24	1.4978	1.61E-06	3.61E-06	yxeM
AB5991_21135	3.63	2	3.11	15	10.71	5.5	2.91	10.40	1.8363	0.000591277	0.001030711	scmL
AB5991_21140	4.5	9.51	6.27	22.83	15.68	6.94	6.76	15.15	1.1642	0.007864298	0.011924065	scmK
AB5991_21145	14.83	23.54	14.88	31.07	38.21	32.71	17.75	34.00	0.9376	0.001348203	0.00226277	yxeI
AB5991_21150	11.78	17.96	18.31	26.6	26.02	15.4	16.02	22.67	0.5014	0.130162101	0.166625952	yxeH

AB5991_21155	0.32	1.78	1.73	11.63	11.87	5.26	1.28	9.59	2.9086	1.31E-06	2.95E-06	yxeG
AB5991_21160	0.42	1.53	1.33	13.11	17.76	4.5	1.09	11.79	3.4308	1.38E-06	3.11E-06	yxeF
AB5991_21165	1304.75	365.44	1367.84	131.59	251.31	427.68	1012.68	270.19	-1.9061	8.74E-05	0.000165241	yxeE
AB5991_21170	88.84	88.13	90.64	4.44	3.64	10.5	89.20	6.19	-3.8483	1.15E-23	9.92E-23	yxeD
AB5991_21175	0	0	0	0	0	0	0.00	0.00	0.0000	1	1	yxeC
AB5991_21180	7.67	10.99	6.2	4.88	5.71	8.91	8.29	6.50	-0.3504	0.380949901	0.438351798	yxeB
AB5991_21185	1.04	0.95	0.56	0.56	0	0	0.85	0.19	-2.1870	0.394326725	0.451650252	yxeA
AB5991_21200	2.77	5.09	2.96	11.66	12.23	8.8	3.61	10.90	1.5951	1.54E-05	3.14E-05	yxdK
AB5991_21205	3.14	2.89	1.4	8.83	7.73	7.09	2.48	7.88	1.6704	0.000115431	0.000214697	yxdJ
AB5991_21210	49.07	75.27	43.84	55.62	69.96	61.63	56.06	62.40	0.1547	0.599290446	0.65576808	iolJ
AB5991_21215	13.17	13.88	10.16	24.43	29.67	30.39	12.40	28.16	1.1831	7.37E-05	0.000139685	iolI
AB5991_21220	21.81	25.18	18.22	33.44	50.32	42.96	21.74	42.24	0.9585	0.000772719	0.001329492	iolH
AB5991_21225	18.51	17.96	14.57	24.5	31.28	35.92	17.01	30.57	0.8453	0.003062727	0.004915962	iolG
AB5991_21230	15.92	21.17	11.15	33.29	44.28	49.18	16.08	42.25	1.3937	7.85E-06	1.65E-05	iolF
AB5991_21235	17.79	15.22	13.62	44.2	45.06	59.97	15.54	49.74	1.6782	3.67E-09	1.03E-08	iolE
AB5991_21240	17.75	22.02	11.31	58.03	66.89	64.3	17.03	63.07	1.8892	3.49E-10	1.06E-09	iolD
AB5991_21245	11.27	15.61	7.71	43.22	56.05	48.82	11.53	49.36	2.0980	1.49E-11	5.12E-11	iolC
AB5991_21250	10.19	6.51	5.45	28.67	44.19	38.36	7.38	37.07	2.3280	1.71E-11	5.84E-11	iolB
AB5991_21255	8.4	8.84	5.68	10.88	15.46	13.77	7.64	13.37	0.8074	0.01000546	0.014953587	iolA
AB5991_21260	16.76	15.96	15.09	0.48	0.23	0.39	15.94	0.37	-5.4417	1.14E-30	1.65E-29	--
AB5991_21265	44.95	43.02	54.97	14.56	10.95	8.54	47.65	11.35	-2.0697	1.85E-11	6.32E-11	iolR
AB5991_21270	111.39	114.9	110.42	310.78	386.85	360.49	112.24	352.71	1.6519	2.65E-10	8.18E-10	iolS
AB5991_21275	0	1.05	0.61	0	0	0	0.55	0.00	-9.1120	0.509711395	0.568309168	--
AB5991_21280	6.14	2.82	4.51	1.67	2.34	3.74	4.49	2.58	-0.7975	0.13398165	0.171129345	--
AB5991_21290	21.07	17.65	24.51	11.66	8.65	10	21.08	10.10	-1.0608	0.001363258	0.002286105	DDB_G0273707
AB5991_21300	19.79	20.47	22.09	58.11	53.01	32.87	20.78	48.00	1.2075	3.88E-05	7.61E-05	htpG

AB5991_21305	2.87	1.32	5.37	0	1.46	0	3.19	0.49	-2.7110	0.027752347	0.039051938	yxcA
AB5991_21315	0.9	13.21	0.96	6.85	10.98	1.95	5.02	6.59	0.3924	0.701738807	0.754155562	MJ0272
AB5991_21320	0	0.83	0.96	2.93	2.29	4.38	0.60	3.20	2.4231	0.008341306	0.012613604	--
AB5991_21325	29.71	27.28	24.71	3.59	1.68	0	27.23	1.76	-3.9545	1.81E-12	6.73E-12	--
AB5991_21330	0	1.5	0	0	0	0	0.50	0.00	-8.9658	1	1	--
AB5991_21335	11.86	10.01	7.06	2.24	3.01	0.9	9.64	2.05	-2.2339	3.23E-07	7.72E-07	--
AB5991_21340	0	1.3	0	0	0	0	0.43	0.00	-8.7593	1	1	--
AB5991_21345	2.81	5.17	2.57	0.99	0.76	1.25	3.52	1.00	-1.8142	4.99E-05	9.66E-05	lagD
AB5991_21350	11.68	15.35	14.99	0.27	2	0.8	14.01	1.02	-3.7748	1.62E-17	8.87E-17	--
AB5991_21360	6.81	7.05	5.6	9.12	8.35	5.67	6.49	7.71	0.2499	0.488790988	0.548456912	qdoI
AB5991_21365	2.2	2.3	4.36	2.05	2.24	1.7	2.95	2.00	-0.5648	0.300604099	0.354213316	yxaF
AB5991_21370	39.15	34.71	41.51	201.35	217.62	227.63	38.46	215.53	2.4866	2.05E-20	1.44E-19	yxnA
AB5991_21375	20.08	17.67	12.08	3.19	2.13	2.72	16.61	2.68	-2.6317	7.97E-09	2.17E-08	yxaD
AB5991_21380	10.71	5.74	10.5	2.91	0	4.35	8.98	2.42	-1.8922	0.001698169	0.002805086	yxzK
AB5991_21385	11.74	9.1	7.25	2.84	1.06	5.65	9.36	3.18	-1.5565	0.00176002	0.002902426	yxaC
AB5991_21390	52.54	61.1	42.52	49.34	55.79	45.5	52.05	50.21	-0.0520	0.863286918	0.904038067	yxaB
AB5991_21395	12.11	16.46	11.94	20.02	26.1	17.88	13.50	21.33	0.6598	0.025478715	0.036069868	glxK
AB5991_21400	18.03	24.48	21.65	1.07	2.01	3.47	21.39	2.18	-3.2921	6.99E-16	3.34E-15	gntR
AB5991_21405	22.86	31.21	18.43	15.55	22.43	22.97	24.17	20.32	-0.2504	0.420310045	0.478101171	gntK
AB5991_21410	20.53	27.1	16.79	15.18	19.53	19.61	21.47	18.11	-0.2460	0.41337136	0.471017983	gntP
AB5991_21415	44.32	57.32	37.92	29.06	36.87	35.32	46.52	33.75	-0.4630	0.104044889	0.135507463	gntZ
AB5991_21420	289.39	307.17	346.15	826.06	1038.52	750.04	314.24	871.54	1.4717	3.37E-08	8.73E-08	ahpC
AB5991_21425	222.22	299.57	207.32	1175.12	1355.16	1151.59	243.04	1227.29	2.3362	7.88E-18	4.45E-17	ahpF
AB5991_21430	49.45	35.03	35.44	28.8	25.93	25.82	39.97	26.85	-0.5741	0.039917484	0.054848557	bglA
AB5991_21435	1.02	3.75	1.09	1.11	0	0	1.95	0.37	-2.4003	0.244219636	0.294591791	yyzE
AB5991_21445	55.93	105.96	59.54	11.61	6.47	6.6	73.81	8.23	-3.1654	1.96E-17	1.06E-16	yydK

AB5991_21450	45.25	52.79	52.4	3.26	3.82	1.89	50.15	2.99	-4.0679	9.58E-30	1.31E-28	yydJ
AB5991_21455	66.27	67.96	61.98	8.74	9.34	14.6	65.40	10.89	-2.5859	8.08E-17	4.20E-16	yydI
AB5991_21460	160.67	137	146.93	41.24	35.53	26.21	148.20	34.33	-2.1101	5.33E-14	2.23E-13	fbp
AB5991_21465	8.24	10.4	12.67	0	0	0	10.44	0.00	-13.3494	4.92E-12	1.77E-11	--
AB5991_21475	17.62	12.79	14.9	2.23	5.01	4.44	15.10	3.89	-1.9558	2.51E-06	5.51E-06	hspC2
AB5991_21485	12.07	12.39	12.65	3.28	5.41	5.33	12.37	4.67	-1.4043	6.17E-05	0.000117824	tnpB
AB5991_21490	5.22	5.43	5.74	1.95	2.3	2.06	5.46	2.10	-1.3771	5.97E-05	0.000114398	tnpA
AB5991_21495	0	0	0	0	0.48	0	0.00	0.16	7.3219	1	1	--
AB5991_21500	0.86	0.68	0.66	0.81	1.26	0.8	0.73	0.96	0.3835	0.667223945	0.721947217	--
AB5991_21505	4.32	3.88	4	3.44	5.96	3.95	4.07	4.45	0.1300	0.73808881	0.787889963	yjcD
AB5991_21510	4.89	5.06	4.28	4.64	5.7	4.62	4.74	4.99	0.0722	0.83845378	0.881053178	MJECL35
AB5991_21520	109.89	83.49	118.12	2.68	2.7	3.08	103.83	2.82	-5.2024	5.12E-35	1.01E-33	--
AB5991_21525	0.11	0.11	39.25	8.23	2.19	0.11	13.16	3.51	-1.9063	0.187913767	0.231956968	--
AB5991_21530	4.85	1.48	4.32	1.32	4.53	1.75	3.55	2.53	-0.4868	0.477592839	0.537562688	--
AB5991_21535	1.3	0	1.39	0.7	0.66	0.7	0.90	0.69	-0.3850	1	1	--
AB5991_21540	15.75	7.54	15.38	0.74	0.7	2.96	12.89	1.47	-3.1356	7.62E-07	1.76E-06	--
AB5991_21545	9.82	12.03	11.91	0.71	1.33	0	11.25	0.68	-4.0487	8.23E-09	2.24E-08	--
AB5991_21550	655.89	435.27	676.17	0	9.89	8.73	589.11	6.21	-6.5686	3.34E-17	1.78E-16	--
AB5991_21555	109.89	83.49	118.12	2.68	2.7	3.08	103.83	2.82	-5.2024	5.02E-35	9.92E-34	--
AB5991_21560	22.23	26.65	19.12	1.77	3.72	2.2	22.67	2.56	-3.1445	4.33E-20	2.93E-19	hel308
AB5991_21565	5.37	10.22	5.32	8.65	11.13	7.32	6.97	9.03	0.3741	0.307780877	0.362240031	--
AB5991_21570	4.52	19.36	8.86	5.32	1.92	2.04	10.91	3.09	-1.8189	0.004460357	0.006985933	rlmH
AB5991_21575	1.06	1.94	1.13	0	0	0	1.38	0.00	-10.4270	0.261997982	0.3141587	yyzF
AB5991_21580	34.93	12.83	33.15	16.62	16.89	8.51	26.97	14.01	-0.9452	0.024609381	0.03500138	yycS
AB5991_21585	201.52	293.75	156.59	502.75	453.83	457.34	217.29	471.31	1.1171	0.000118738	0.00022064	yycR
AB5991_21590	0	0	2.26	1.15	5.38	0	0.75	2.18	1.5308	0.512122923	0.569081258	yyzG

AB5991_21595	21.05	26.66	13.97	10.26	12.56	13.36	20.56	12.06	-0.7696	0.072750314	0.096683901	yycQ
AB5991_21600	67.12	62.19	58.26	21.39	17.95	29.06	62.52	22.80	-1.4554	5.71E-07	1.33E-06	yycP
AB5991_21605	97.97	102.54	97.96	52.74	55.59	50.11	99.49	52.81	-0.9136	0.000532805	0.000933702	yycO
AB5991_21610	47.2	70.47	56.63	9.6	6.25	9.55	58.10	8.47	-2.7787	9.37E-16	4.44E-15	yycN
AB5991_21615	61.9	68.92	61.61	121.21	138.56	122.6	64.14	127.46	0.9906	8.93E-05	0.000168627	rapG
AB5991_21620	7.72	14.18	16.52	33.6	26.73	31.77	12.81	30.70	1.2613	0.007686627	0.011681437	phrG
AB5991_21625	49.09	26.45	36.23	113.84	155.47	153.28	37.26	140.86	1.9187	3.27E-10	9.99E-10	rocF
AB5991_21630	36.3	20.33	21.2	68.61	89.23	89.33	25.94	82.39	1.6671	5.68E-08	1.44E-07	rocE
AB5991_21635	85.43	57.79	48.08	225.27	273.8	304.52	63.77	267.86	2.0706	6.43E-12	2.28E-11	rocD
AB5991_21640	24.34	39.78	19.04	280.83	335.9	349.88	27.72	322.20	3.5390	8.46E-27	9.42E-26	yitM
AB5991_21645	0	0	0.41	2.47	2.51	1.03	0.14	2.00	3.8737	4.69E-05	9.10E-05	yitO
AB5991_21650	0.65	0.6	1.04	4.96	2.98	1.76	0.76	3.23	2.0826	0.004678415	0.007302668	yitP
AB5991_21655	2.56	7.31	1.52	3.71	2.31	3.38	3.80	3.13	-0.2770	0.808837104	0.852639272	sdpI
AB5991_21660	0.63	0	0.68	0.69	0.65	2.06	0.44	1.13	1.3760	0.480208266	0.539822237	sdpR
AB5991_21665	37.56	30.65	28.59	9.93	8.89	6.92	32.27	8.58	-1.9110	2.15E-11	7.30E-11	rocR
AB5991_21680	87.9	114.77	76.8	30.07	39.3	33.5	93.16	34.29	-1.4419	2.77E-07	6.66E-07	yyxA
AB5991_21685	5.68	1.67	7.78	25.72	22.21	21.9	5.04	23.28	2.2064	2.83E-08	7.37E-08	yycJ
AB5991_21690	7.93	5.12	5.96	28.68	26.84	22.75	6.34	26.09	2.0417	3.71E-10	1.12E-09	yycI
AB5991_21695	14.4	12.13	17.24	35.64	34.56	34.47	14.59	34.89	1.2578	3.88E-06	8.37E-06	yycH
AB5991_21700	13.39	13.74	11.26	44.33	46.29	36.45	12.80	42.36	1.7268	8.94E-10	2.62E-09	walK
AB5991_21705	9.19	11.72	10.1	43.31	41.83	35.37	10.34	40.17	1.9583	7.80E-11	2.52E-10	walR
AB5991_21730	32.15	43.64	28.7	368.68	147.68	143.15	34.83	219.84	2.6580	1.12E-11	3.89E-11	purA
AB5991_21735	1.72	0	0	3.28	6.57	2.33	0.57	4.06	2.8240	0.002927327	0.004719616	yycE
AB5991_21740	24.63	25.29	20.25	23.19	17.79	12.76	23.39	17.91	-0.3849	0.193389938	0.238198339	dnaC
AB5991_21745	31.47	21.47	25.96	13.69	10.07	7.79	26.30	10.52	-1.3224	0.001422619	0.00237462	yycD
AB5991_21750	4.43	4.88	5.69	0	0	0	5.00	0.00	-12.2877	0.000307968	0.00055013	yyzB

AB5991_21755	29.48	14.12	26.05	47.4	79.59	86.04	23.22	71.01	1.6129	7.00E-05	0.000133027	yycC
AB5991_21760	201.38	159.22	152.36	488.93	559.48	612.88	170.99	553.76	1.6954	2.29E-10	7.10E-10	yycB
AB5991_21765	4.83	4.03	3.48	10.89	12.96	13.79	4.11	12.55	1.6089	1.26E-07	3.10E-07	yycA
AB5991_21770	51.41	50.15	29.64	252.93	230.97	227.4	43.73	237.10	2.4387	6.60E-16	3.17E-15	rplI
AB5991_21775	12.69	8.72	12.69	48.35	49.61	42.99	11.37	46.98	2.0473	1.51E-12	5.68E-12	gdpP
AB5991_21780	8.55	6.07	12.89	16.06	16.81	10.31	9.17	14.39	0.6504	0.076379958	0.101270388	yybS
AB5991_21785	2.41	0	1.29	9.17	8.58	13.04	1.23	10.26	3.0569	0.000762204	0.001311969	yyzH
AB5991_21795	5.74	4.39	4.6	12.48	5.84	3.11	4.91	7.14	0.5409	0.377516453	0.435283924	yybR
AB5991_21800	74.44	88.15	81.27	143.74	116.31	75.32	81.29	111.79	0.4597	0.114929971	0.14846679	ppaC
AB5991_21805	7.68	11.14	6.49	2.2	2.47	1.75	8.44	2.14	-1.9791	9.02E-05	0.000169917	yybP
AB5991_21810	6.19	3.1	1.81	0	1.72	3.05	3.70	1.59	-1.2185	0.150882135	0.189485438	--
AB5991_21815	1.38	5.06	3.54	2.1	2.81	2.24	3.33	2.38	-0.4811	0.392361616	0.449788677	yybO
AB5991_21820	2.71	5.42	3.68	8.02	8.51	8.78	3.94	8.44	1.0997	0.003368062	0.005371315	bltR
AB5991_21825	38.94	39.96	42.38	11.21	17.95	10.66	40.43	13.27	-1.6068	1.24E-07	3.05E-07	yybI
AB5991_21830	17.15	13.62	18.34	4.54	10.85	4.52	16.37	6.64	-1.3025	0.00248306	0.004039422	yybH
AB5991_21835	0.43	1.58	0.69	3.28	5.91	4.43	0.90	4.54	2.3347	3.12E-05	6.17E-05	yybG
AB5991_21850	18.75	13.19	15.36	8.25	6.5	8.21	15.77	7.65	-1.0427	0.003342116	0.005332078	yybA
AB5991_21855	14.56	14.85	12.11	3.52	1.23	1.31	13.84	2.02	-2.7764	5.20E-09	1.44E-08	yyaT
AB5991_21860	16.49	19.26	24.36	1.63	1.53	4.54	20.04	2.57	-2.9647	2.33E-11	7.88E-11	yyaS
AB5991_21865	0.35	0	0	2.64	1.06	2.25	0.12	1.98	4.0875	0.001803956	0.002973644	satA
AB5991_21870	36.96	37.19	41.15	20.37	30.4	24.66	38.43	25.14	-0.6122	0.044962737	0.061230119	yyaQ
AB5991_21875	21.04	31.02	21.14	15.6	28.55	18.63	24.40	20.93	-0.2215	0.542166305	0.599371492	yyaP
AB5991_21885	20.89	23.6	16.32	36.26	38.02	33.48	20.27	35.92	0.8254	0.005294065	0.008212006	yosT
AB5991_21890	37.9	40.08	43.33	126.78	147.88	137.62	40.44	137.43	1.7649	9.65E-12	3.37E-11	yyaL
AB5991_21895	50.41	35.03	58.63	5.24	4.5	4.57	48.02	4.77	-3.3317	2.00E-22	1.61E-21	yyaK
AB5991_21900	28.53	28.39	30.08	28.27	16.82	14.43	29.00	19.84	-0.5476	0.072208689	0.096060537	yyaJ

AB5991_21905	28.21	49.17	19.43	2.08	1.46	5.69	32.27	3.08	-3.3908	3.72E-10	1.13E-09	yyaH
AB5991_21910	1.36	5	2	16.1	7.45	7.19	2.79	10.25	1.8785	0.00020622	0.000374783	moeB
AB5991_21915	0.88	0.65	0.56	16.24	6.08	4.75	0.70	9.02	3.6951	9.11E-09	2.48E-08	clpB
AB5991_21920	2.41	2.86	3.34	4.32	4.91	3.07	2.87	4.10	0.5146	0.203502026	0.249492604	--
AB5991_21930	2.62	3.94	2.29	9.06	4.36	10.05	2.95	7.82	1.4071	0.002077437	0.00340326	exoA
AB5991_21935	141.59	95.42	124.84	596.3	561.84	489.97	120.62	549.37	2.1873	7.36E-15	3.28E-14	rpsR
AB5991_21940	356.62	265.38	240.97	1211.68	1200.16	1153.23	287.66	1188.36	2.0465	7.75E-14	3.18E-13	ssbA
AB5991_21945	166.31	107.17	127.52	494.18	515.47	457.22	133.67	488.96	1.8711	1.71E-11	5.84E-11	rpsF
AB5991_21950	16.09	16.28	16.33	31.78	32.25	20.44	16.23	28.16	0.7945	0.006284742	0.009665651	ychF
AB5991_21955	7.04	7.45	5.69	6.57	9.91	6.25	6.73	7.58	0.1717	0.592250188	0.649007111	yyaE
AB5991_21960	0	6.41	1.87	12.35	1.78	2.84	2.76	5.66	1.0353	0.313440721	0.36824648	yyzM
AB5991_21965	1.6	2.28	1.9	0	0	0	1.93	0.00	-10.9119	2.70E-07	6.49E-07	yyaD
AB5991_21970	9.07	32.22	21.89	3.18	0.89	1.58	21.06	1.88	-3.4831	1.57E-09	4.55E-09	yyaC
AB5991_21975	49.39	72.71	39.84	72.94	65.22	55.31	53.98	64.49	0.2567	0.392611234	0.449944938	spo0J
AB5991_21980	33.68	49.22	37.04	44.11	44.18	29.02	39.98	39.10	-0.0320	0.935373611	0.972347803	soj
AB5991_21985	16.8	18.06	9.64	57.5	70.91	54.57	14.83	60.99	2.0398	7.57E-10	2.24E-09	yyaB
AB5991_21990	58.34	69.73	53.77	82.37	106.66	88.64	60.61	92.56	0.6107	0.021647082	0.031043902	noc
AB5991_21995	17.32	20.74	24.97	64.16	83.55	70.66	21.01	72.79	1.7927	3.96E-10	1.20E-09	rsmG
AB5991_22000	35.44	31.48	33.29	62.09	73.31	77.77	33.40	71.06	1.0890	2.33E-05	4.66E-05	mnmG
AB5991_22005	26.46	28.62	26.61	17.66	17.33	13.47	27.23	16.15	-0.7534	0.00412275	0.006483739	mnmE
AB5991_22010	78.7	66.7	62.27	93.12	73.35	31.52	69.22	66.00	-0.0689	0.853257027	0.895187226	khpB
AB5991_22015	48.29	45.6	48.69	69.78	49.15	26.88	47.53	48.60	0.0323	0.929756778	0.967522056	misCA
AB5991_22020	0	0.95	0	7.84	14.15	8.92	0.32	10.30	5.0240	2.20E-09	6.27E-09	rnpA
AB5991_22025	37.49	71.29	60.14	164.55	208.49	130.44	56.31	167.83	1.5756	4.64E-06	9.96E-06	rpmH
