# Supplementary material for: Genomic and Transcriptomic Analysis of Mutant Bacillus subtilis with Enhanced Nattokinase Production via ARTP Mutagenesis
Source: Foods. 2025 Mar 6;14(5):898. doi: 10.3390/foods14050898 (PMC11899143; doi:10.3390/foods14050898)
Supplement: Supplementary file 1 [file foods-14-00898-s001.zip › Table S4.pdf]

Table S4. GO terms significantly enriched in up-regulated genes.

GO ID	Description	up-2 (1011)	All (3094)	ratio	class	pvalue	p.adjust
GO:0005737	cytoplasm	461	1126	0.409	Cellular Component	2.15E-17	7.87E-15
GO:0005829	cytosol	176	342	0.515	Cellular Component	1.85E-15	3.39E-13
GO:0005622	intracellular anatomical structure	505	1299	0.389	Cellular Component	6.71E-14	8.18E-12
GO:1901566	organonitrogen compound biosynthetic process	443	1075	0.412	Biological Process	1.55E-13	3.44E-10
GO:0044281	small molecule metabolic process	482	1201	0.401	Biological Process	1.56E-12	1.32E-09
GO:0040007	growth	69	105	0.657	Biological Process	1.79E-12	1.32E-09
GO:0044391	ribosomal subunit	56	83	0.675	Cellular Component	2.72E-11	2.49E-09
GO:0043603	cellular amide metabolic process	223	480	0.465	Biological Process	5.12E-12	2.83E-09
GO:1901564	organonitrogen compound metabolic process	582	1510	0.385	Biological Process	6.77E-12	3.00E-09
GO:0043604	amide biosynthetic process	189	394	0.48	Biological Process	1.00E-11	3.70E-09
GO:0022626	cytosolic ribosome	54	80	0.675	Cellular Component	6.14E-11	4.49E-09
GO:0009165	nucleotide biosynthetic process	137	267	0.513	Biological Process	3.33E-11	9.22E-09
GO:1901293	nucleoside phosphate biosynthetic process	137	267	0.513	Biological Process	3.33E-11	9.22E-09
GO:0090407	organophosphate biosynthetic process	172	358	0.48	Biological Process	9.28E-11	2.28E-08
GO:0008152	metabolic process	883	2508	0.352	Biological Process	1.23E-10	2.73E-08
GO:0009156	ribonucleoside monophosphate biosynthetic process	61	96	0.635	Biological Process	3.02E-10	6.08E-08
GO:0044283	small molecule biosynthetic process	310	737	0.421	Biological Process	5.33E-10	9.84E-08
GO:0019843	rRNA binding	55	81	0.679	Molecular Function	1.14E-10	1.84E-07
GO:0009117	nucleotide metabolic process	162	341	0.475	Biological Process	1.10E-09	1.88E-07
GO:0006753	nucleoside phosphate metabolic process	162	342	0.474	Biological Process	1.46E-09	2.19E-07
GO:0006518	peptide metabolic process	140	286	0.49	Biological Process	1.48E-09	2.19E-07
GO:0009124	nucleoside monophosphate biosynthetic process	62	101	0.614	Biological Process	1.67E-09	2.27E-07
GO:0043043	peptide biosynthetic process	131	264	0.496	Biological Process	1.78E-09	2.27E-07

GO:0009260	ribonucleotide biosynthetic process	116	227	0.511	Biological Process	1.84E-09	2.27E-07
GO:0009161	ribonucleoside monophosphate metabolic process	67	113	0.593	Biological Process	2.92E-09	3.41E-07
GO:0046390	ribose phosphate biosynthetic process	116	229	0.507	Biological Process	3.65E-09	4.04E-07
GO:1990904	ribonucleoprotein complex	62	105	0.59	Cellular Component	8.05E-09	4.91E-07
GO:0009123	nucleoside monophosphate metabolic process	70	122	0.574	Biological Process	9.03E-09	9.53E-07
GO:0019637	organophosphate metabolic process	225	520	0.433	Biological Process	1.89E-08	1.86E-06
GO:0019752	carboxylic acid metabolic process	331	816	0.406	Biological Process	1.93E-08	1.86E-06
GO:0003824	catalytic activity	801	2212	0.362	Molecular Function	5.62E-09	3.50E-06
GO:0046872	metal ion binding	267	620	0.431	Molecular Function	6.98E-09	3.50E-06
GO:0043169	cation binding	271	632	0.429	Molecular Function	8.68E-09	3.50E-06
GO:0043436	oxoacid metabolic process	333	826	0.403	Biological Process	3.95E-08	3.65E-06
GO:0019693	ribose phosphate metabolic process	138	293	0.471	Biological Process	4.76E-08	4.22E-06
GO:0006796	phosphate-containing compound metabolic process	261	626	0.417	Biological Process	7.02E-08	5.98E-06
GO:0071704	organic substance metabolic process	835	2380	0.351	Biological Process	7.33E-08	6.01E-06
GO:0044237	cellular metabolic process	816	2317	0.352	Biological Process	8.02E-08	6.34E-06
GO:0009168	purine ribonucleoside monophosphate biosynthetic process	44	69	0.638	Biological Process	8.58E-08	6.49E-06
GO:0009259	ribonucleotide metabolic process	134	285	0.47	Biological Process	8.79E-08	6.49E-06
GO:0044238	primary metabolic process	789	2230	0.354	Biological Process	1.13E-07	8.05E-06
GO:1901576	organic substance biosynthetic process	637	1746	0.365	Biological Process	1.53E-07	1.04E-05
GO:0009127	purine nucleoside monophosphate biosynthetic process	44	70	0.629	Biological Process	1.59E-07	1.04E-05
GO:0009058	biosynthetic process	642	1762	0.364	Biological Process	1.60E-07	1.04E-05
GO:0000313	organellar ribosome	41	65	0.631	Cellular Component	2.53E-07	1.32E-05
GO:0009152	purine ribonucleotide biosynthetic process	97	195	0.497	Biological Process	2.50E-07	1.58E-05
GO:0005840	ribosome	62	113	0.549	Cellular Component	3.54E-07	1.62E-05
GO:0006164	purine nucleotide biosynthetic process	97	197	0.492	Biological Process	4.64E-07	2.86E-05
GO:0009056	catabolic process	265	649	0.408	Biological Process	5.44E-07	3.17E-05

GO:0006793	phosphorus metabolic process	282	697	0.405	Biological Process	5.44E-07	3.17E-05
GO:0055086	nucleobase-containing small molecule metabolic process	192	447	0.43	Biological Process	5.60E-07	3.18E-05
GO:0019538	protein metabolic process	283	701	0.404	Biological Process	6.56E-07	3.63E-05
GO:1901575	organic substance catabolic process	254	620	0.41	Biological Process	7.46E-07	4.03E-05
GO:0006082	organic acid metabolic process	334	849	0.393	Biological Process	8.95E-07	4.72E-05
GO:1901617	organic hydroxy compound biosynthetic process	67	126	0.532	Biological Process	9.88E-07	5.09E-05
GO:0009167	purine ribonucleoside monophosphate metabolic process	47	80	0.588	Biological Process	1.08E-06	5.42E-05
GO:0072522	purine-containing compound biosynthetic process	104	218	0.477	Biological Process	1.23E-06	6.07E-05
GO:0044550	secondary metabolite biosynthetic process	28	40	0.7	Biological Process	1.28E-06	6.18E-05
GO:0009150	purine ribonucleotide metabolic process	121	262	0.462	Biological Process	1.34E-06	6.34E-05
GO:0046394	carboxylic acid biosynthetic process	228	551	0.414	Biological Process	1.38E-06	6.37E-05
GO:0009126	purine nucleoside monophosphate metabolic process	47	81	0.58	Biological Process	1.77E-06	7.99E-05
GO:0015935	small ribosomal subunit	28	41	0.683	Cellular Component	2.15E-06	8.75E-05
GO:0006412	translation	108	230	0.47	Biological Process	1.99E-06	8.83E-05
GO:0044249	cellular biosynthetic process	624	1726	0.362	Biological Process	2.04E-06	8.85E-05
GO:0006163	purine nucleotide metabolic process	123	269	0.457	Biological Process	2.09E-06	8.89E-05
GO:1901615	organic hydroxy compound metabolic process	112	241	0.465	Biological Process	2.39E-06	9.99E-05
GO:0042255	ribosome assembly	26	37	0.703	Biological Process	2.78E-06	0.00011401
GO:0015934	large ribosomal subunit	28	42	0.667	Cellular Component	4.49E-06	0.000142328
GO:0022625	cytosolic large ribosomal subunit	27	40	0.675	Cellular Component	4.67E-06	0.000142328
GO:0022627	cytosolic small ribosomal subunit	27	40	0.675	Cellular Component	4.67E-06	0.000142328
GO:0044267	cellular protein metabolic process	214	518	0.413	Biological Process	3.77E-06	0.000151979
GO:0006553	lysine metabolic process	46	81	0.568	Biological Process	5.17E-06	0.000204428
GO:0019748	secondary metabolic process	34	55	0.618	Biological Process	7.30E-06	0.000283542
GO:0006066	alcohol metabolic process	86	179	0.48	Biological Process	7.79E-06	0.000293048
GO:0016053	organic acid biosynthetic process	231	570	0.405	Biological Process	7.81E-06	0.000293048

GO:0003735	structural constituent of ribosome	61	110	0.555	Molecular Function	9.19E-07	0.000296419
GO:1901137	carbohydrate derivative biosynthetic process	195	471	0.414	Biological Process	9.84E-06	0.000363433
GO:0044272	sulfur compound biosynthetic process	132	300	0.44	Biological Process	1.06E-05	0.000386388
GO:0072528	pyrimidine-containing compound biosynthetic process	63	123	0.512	Biological Process	1.08E-05	0.000387116
GO:0006629	lipid metabolic process	154	361	0.427	Biological Process	1.52E-05	0.000534545
GO:0032787	monocarboxylic acid metabolic process	189	458	0.413	Biological Process	1.80E-05	0.000622153
GO:0006084	acetyl-CoA metabolic process	30	48	0.625	Biological Process	1.87E-05	0.000636758
GO:0044248	cellular catabolic process	205	503	0.408	Biological Process	1.94E-05	0.000652091
GO:0009085	lysine biosynthetic process	39	68	0.574	Biological Process	2.00E-05	0.000661087
GO:0035384	thioester biosynthetic process	28	44	0.636	Biological Process	2.19E-05	0.000703698
GO:0071616	acyl-CoA biosynthetic process	28	44	0.636	Biological Process	2.19E-05	0.000703698
GO:0006085	acetyl-CoA biosynthetic process	26	40	0.65	Biological Process	2.50E-05	0.000792516
GO:0072521	purine-containing compound metabolic process	133	307	0.433	Biological Process	2.56E-05	0.000798206
GO:0008610	lipid biosynthetic process	119	270	0.441	Biological Process	2.78E-05	0.00085636
GO:0072350	tricarboxylic acid metabolic process	23	34	0.676	Biological Process	2.85E-05	0.000864739
GO:0006221	pyrimidine nucleotide biosynthetic process	31	51	0.608	Biological Process	3.02E-05	0.000903525
GO:0016999	antibiotic metabolic process	42	76	0.553	Biological Process	3.31E-05	0.000977296
GO:0009237	siderophore metabolic process	14	17	0.824	Biological Process	3.45E-05	0.000991814
GO:0019290	siderophore biosynthetic process	14	17	0.824	Biological Process	3.45E-05	0.000991814
GO:0072330	monocarboxylic acid biosynthetic process	103	229	0.45	Biological Process	3.66E-05	0.001028301
GO:0009220	pyrimidine ribonucleotide biosynthetic process	29	47	0.617	Biological Process	3.67E-05	0.001028301
GO:0044271	cellular nitrogen compound biosynthetic process	474	1293	0.367	Biological Process	3.83E-05	0.001059384
GO:0044282	small molecule catabolic process	148	350	0.423	Biological Process	4.02E-05	0.001091956
GO:0046165	alcohol biosynthetic process	54	105	0.514	Biological Process	4.04E-05	0.001091956
GO:0044255	cellular lipid metabolic process	142	334	0.425	Biological Process	4.26E-05	0.001137515
GO:0006637	acyl-CoA metabolic process	32	54	0.593	Biological Process	4.62E-05	0.00120474

GO:0035383	thioester metabolic process	32	54	0.593	Biological Process	4.62E-05	0.00120474
GO:0022618	ribonucleoprotein complex assembly	26	41	0.634	Biological Process	4.74E-05	0.0012075
GO:0071826	ribonucleoprotein complex subunit organization	26	41	0.634	Biological Process	4.74E-05	0.0012075
GO:1901135	carbohydrate derivative metabolic process	244	621	0.393	Biological Process	6.06E-05	0.001526503
GO:0009218	pyrimidine ribonucleotide metabolic process	29	48	0.604	Biological Process	6.41E-05	0.001596114
GO:0016052	carbohydrate catabolic process	87	190	0.458	Biological Process	6.94E-05	0.001707494
GO:0006220	pyrimidine nucleotide metabolic process	31	53	0.585	Biological Process	8.62E-05	0.002038496
GO:0033866	nucleoside bisphosphate biosynthetic process	41	76	0.539	Biological Process	8.65E-05	0.002038496
GO:0034030	ribonucleoside bisphosphate biosynthetic process	41	76	0.539	Biological Process	8.65E-05	0.002038496
GO:0034033	purine nucleoside bisphosphate biosynthetic process	41	76	0.539	Biological Process	8.65E-05	0.002038496
GO:0019287	isopentenyl diphosphate biosynthetic process, mevalonate pathway	13	16	0.812	Biological Process	8.82E-05	0.002056288
GO:0072527	pyrimidine-containing compound metabolic process	71	151	0.47	Biological Process	0.00011979	0.002763895
GO:0043167	ion binding	467	1239	0.377	Molecular Function	1.09E-05	0.00293134
GO:0006526	arginine biosynthetic process	16	22	0.727	Biological Process	0.000133056	0.003038335
GO:0006790	sulfur compound metabolic process	195	488	0.4	Biological Process	0.000136136	0.003076941
GO:0042180	cellular ketone metabolic process	80	175	0.457	Biological Process	0.000147112	0.003291442
GO:0019427	acetyl-CoA biosynthetic process from acetate	11	13	0.846	Biological Process	0.000168519	0.003732692
GO:0042181	ketone biosynthetic process	59	122	0.484	Biological Process	0.000174925	0.00383622
GO:0034404	nucleobase-containing small molecule biosynthetic process	43	83	0.518	Biological Process	0.000200008	0.004343319
GO:0006520	cellular amino acid metabolic process	212	539	0.393	Biological Process	0.000202857	0.004362415
GO:0006807	nitrogen compound metabolic process	736	2121	0.347	Biological Process	0.00021077	0.004489
GO:0009066	aspartate family amino acid metabolic process	79	174	0.454	Biological Process	0.000213838	0.004510954
GO:0033865	nucleoside bisphosphate metabolic process	49	98	0.5	Biological Process	0.000227126	0.004658183
GO:0033875	ribonucleoside bisphosphate metabolic process	49	98	0.5	Biological Process	0.000227126	0.004658183
GO:0034032	purine nucleoside bisphosphate metabolic process	49	98	0.5	Biological Process	0.000227126	0.004658183
GO:0016874	ligase activity	98	210	0.467	Molecular Function	2.05E-05	0.004723916

GO:0032991	protein-containing complex	277	737	0.376	Cellular Component	0.000178756	0.004838119
GO:0000315	organellar large ribosomal subunit	22	35	0.629	Cellular Component	0.000185065	0.004838119
GO:0009233	menaquinone metabolic process	39	74	0.527	Biological Process	0.000247976	0.004993339
GO:0009234	menaquinone biosynthetic process	39	74	0.527	Biological Process	0.000247976	0.004993339
GO:0072525	pyridine-containing compound biosynthetic process	41	79	0.519	Biological Process	0.000267257	0.005333093
GO:0042274	ribosomal small subunit biogenesis	14	19	0.737	Biological Process	0.00028868	0.005709157
GO:0000314	organellar small ribosomal subunit	19	29	0.655	Cellular Component	0.000234821	0.005729626
GO:0072524	pyridine-containing compound metabolic process	45	89	0.506	Biological Process	0.000295324	0.005788866
GO:1901661	quinone metabolic process	57	119	0.479	Biological Process	0.000309934	0.005969601
GO:1901663	quinone biosynthetic process	57	119	0.479	Biological Process	0.000309934	0.005969601
GO:0043232	intracellular non-membrane-bounded organelle	88	201	0.438	Cellular Component	0.000264526	0.006051023
GO:0005975	carbohydrate metabolic process	182	459	0.397	Biological Process	0.000391285	0.007466769
GO:0000272	polysaccharide catabolic process	30	54	0.556	Biological Process	0.000394407	0.007466769
GO:1901605	alpha-amino acid metabolic process	164	409	0.401	Biological Process	0.000425135	0.007980296
GO:0006099	tricarboxylic acid cycle	30	55	0.545	Biological Process	0.000601164	0.011189736
GO:0070887	cellular response to chemical stimulus	52	109	0.477	Biological Process	0.000639177	0.01171612
GO:0019184	nonribosomal peptide biosynthetic process	16	24	0.667	Biological Process	0.000640023	0.01171612
GO:0006631	fatty acid metabolic process	71	158	0.449	Biological Process	0.000647316	0.011725637
GO:0019318	hexose metabolic process	46	94	0.489	Biological Process	0.00065113	0.011725637
GO:0016491	oxidoreductase activity	221	546	0.405	Molecular Function	5.88E-05	0.011851226
GO:0006091	generation of precursor metabolites and energy	104	247	0.421	Biological Process	0.000762091	0.013613163
GO:0070925	organelle assembly	33	63	0.524	Biological Process	0.000850847	0.014957345
GO:0140694	non-membrane-bounded organelle assembly	33	63	0.524	Biological Process	0.000850847	0.014957345
GO:0006633	fatty acid biosynthetic process	42	85	0.494	Biological Process	0.000864533	0.015078272
GO:0000287	magnesium ion binding	38	68	0.559	Molecular Function	8.84E-05	0.01582453
GO:0005996	monosaccharide metabolic process	55	118	0.466	Biological Process	0.000914593	0.01582674

GO:0034641	cellular nitrogen compound metabolic process	601	1715	0.35	Biological Process	0.00097418	0.016727194
GO:0043229	intracellular organelle	138	346	0.399	Cellular Component	0.000874247	0.018822032
GO:0045333	cellular respiration	50	106	0.472	Biological Process	0.00111526	0.019002316
GO:0017000	antibiotic biosynthetic process	22	38	0.579	Biological Process	0.001125212	0.019025534
GO:0033611	oxalate catabolic process	6	6	1	Biological Process	0.001205137	0.020172579
GO:0009057	macromolecule catabolic process	68	153	0.444	Biological Process	0.001211265	0.020172579
GO:0015980	energy derivation by oxidation of organic compounds	71	161	0.441	Biological Process	0.001233234	0.020385181
GO:0046040	IMP metabolic process	16	25	0.64	Biological Process	0.001243345	0.020400061
GO:0009141	nucleoside triphosphate metabolic process	35	69	0.507	Biological Process	0.001276232	0.020785687
GO:0003987	acetate-CoA ligase activity	11	13	0.846	Molecular Function	0.000202818	0.021796199
GO:0008668	(2,3-dihydroxybenzoyl)adenylate synthase activity	11	13	0.846	Molecular Function	0.000202818	0.021796199
GO:0008756	o-succinylbenzoate-CoA ligase activity	11	13	0.846	Molecular Function	0.000202818	0.021796199
GO:0016405	CoA-ligase activity	11	13	0.846	Molecular Function	0.000202818	0.021796199
GO:0030729	acetoacetate-CoA ligase activity	11	13	0.846	Molecular Function	0.000202818	0.021796199
GO:0050218	propionate-CoA ligase activity	11	13	0.846	Molecular Function	0.000202818	0.021796199
GO:0009067	aspartate family amino acid biosynthetic process	64	143	0.448	Biological Process	0.001352052	0.021859813
GO:0009199	ribonucleoside triphosphate metabolic process	32	62	0.516	Biological Process	0.001407967	0.022598896
GO:0000027	ribosomal large subunit assembly	14	21	0.667	Biological Process	0.001418464	0.022603577
GO:0008652	cellular amino acid biosynthetic process	148	373	0.397	Biological Process	0.001442576	0.022823609
GO:0000028	ribosomal small subunit assembly	11	15	0.733	Biological Process	0.00146474	0.023009917
GO:0006006	glucose metabolic process	26	48	0.542	Biological Process	0.001583007	0.02452
GO:0009089	lysine biosynthetic process via diaminopimelate	26	48	0.542	Biological Process	0.001583007	0.02452
GO:0009014	succinyl-diaminopimelate desuccinylase activity	12	15	0.8	Molecular Function	0.00027076	0.025674437
GO:0047473	D-alanine [D-alanyl carrier protein] ligase activity	12	15	0.8	Molecular Function	0.00027076	0.025674437
GO:0009119	ribonucleoside metabolic process	35	70	0.5	Biological Process	0.001767319	0.027184809
GO:0009201	ribonucleoside triphosphate biosynthetic process	27	51	0.529	Biological Process	0.0020273	0.030968757

GO:0042254	ribosome biogenesis	71	164	0.433	Biological Process	0.002249629	0.034129645
GO:0006188	IMP biosynthetic process	15	24	0.625	Biological Process	0.002499659	0.037664932
GO:1990204	oxidoreductase complex	39	81	0.481	Cellular Component	0.001908721	0.038810651
GO:0031522	cell envelope Sec protein transport complex	7	8	0.875	Cellular Component	0.002098492	0.04042359
GO:0019359	nicotinamide nucleotide biosynthetic process	25	47	0.532	Biological Process	0.002708125	0.040435374
GO:0006101	citrate metabolic process	14	22	0.636	Biological Process	0.002738287	0.040435374
GO:0006189	'de novo' IMP biosynthetic process	14	22	0.636	Biological Process	0.002738287	0.040435374
GO:0009163	nucleoside biosynthetic process	27	52	0.519	Biological Process	0.002929071	0.042683495
GO:0042455	ribonucleoside biosynthetic process	27	52	0.519	Biological Process	0.002929071	0.042683495
GO:0046496	nicotinamide nucleotide metabolic process	29	57	0.509	Biological Process	0.003086108	0.044677973
GO:0042364	water-soluble vitamin biosynthetic process	99	242	0.409	Biological Process	0.003132511	0.045055265
GO:0046132	pyrimidine ribonucleoside biosynthetic process	10	14	0.714	Biological Process	0.003356521	0.047581763
GO:0046134	pyrimidine nucleoside biosynthetic process	10	14	0.714	Biological Process	0.003356521	0.047581763
GO:0009081	branched-chain amino acid metabolic process	26	50	0.52	Biological Process	0.003392925	0.047581763
GO:0019751	polyol metabolic process	44	95	0.463	Biological Process	0.003394094	0.047581763
GO:0006083	acetate metabolic process	17	29	0.586	Biological Process	0.003439986	0.047921816
GO:0006767	water-soluble vitamin metabolic process	103	254	0.406	Biological Process	0.003610142	0.049675962
GO:0009110	vitamin biosynthetic process	99	243	0.407	Biological Process	0.003643506	0.049675962
GO:0006785	heme B biosynthetic process	5	5	1	Biological Process	0.003700467	0.049675962
GO:0046492	heme B metabolic process	5	5	1	Biological Process	0.003700467	0.049675962
GO:0070813	hydrogen sulfide metabolic process	5	5	1	Biological Process	0.003700467	0.049675962
GO:0070814	hydrogen sulfide biosynthetic process	5	5	1	Biological Process	0.003700467	0.049675962
