# Supplementary figures and images for: Genomic and Transcriptomic Analysis of Mutant Bacillus subtilis with Enhanced Nattokinase Production via ARTP Mutagenesis
Source: Foods. 2025 Mar 6;14(5):898. doi: 10.3390/foods14050898 (PMC11899143; doi:10.3390/foods14050898)

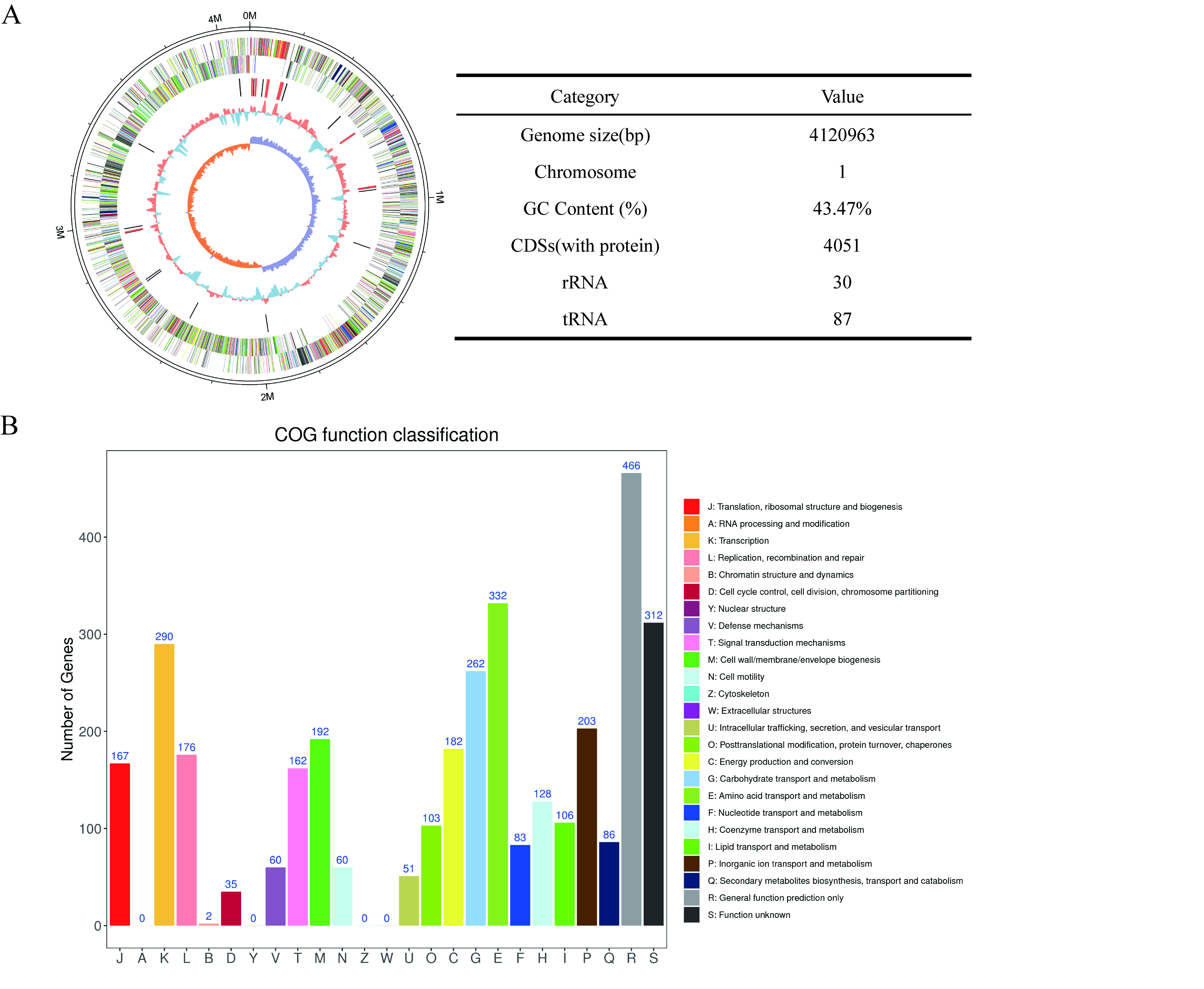

Supplement: Supplementary file 1 [file foods-14-00898-s001.zip › Figure S1.tif]
